# Assessing Causality Between Endocrine, Nutritional, and Metabolic Disease and Pulmonary Tuberculosis: A Mendelian Randomization Study

**DOI:** 10.1002/hsr2.70875

**Published:** 2025-05-29

**Authors:** Yan Gao, Yiguo Wang, Jinwen Su, Chunxia Zhang, Qiming Zhang, Zhi Chen

**Affiliations:** ^1^ ICU, Tuberculosis Department 8th Medical Center of Chinese PLA General Hospital Tuberculosis Research Institute Beijing China; ^2^ Experimental Research Center China Academy of Chinese Medical Sciences Beijing China

**Keywords:** endocrine, European ancestry, Mendelian randomization, metabolic disease, nutritional, pulmonary tuberculosis

## Abstract

**Background and Aims:**

Observational studies frequently report co‐occurrence between endocrine, nutritional, and metabolic disease (ENMD) and pulmonary tuberculosis (PTB). However, the causal properties between them remain poorly defined. Our aim in this study was to investigate the causal effect of ENMD on PTB using Mendelian randomization analysis.

**Methods:**

We obtained single nucleotide polymorphisms linked to ENMD, ENMD‐related diseases, and clinical features, as well as PTB, from the IEU OpenGWAS project. Inverse variance weighting was used as the primary analytical method, complemented by Weighted median and MR‐Egger regression to assess their causal relationship. Heterogeneity and horizontal pleiotropy were assessed using Cochran's Q test and MR regression intercepts. The robustness of the results is evaluated by sensitivity analysis leave‐one‐out and MR‐PRESSO.

**Results:**

The inverse variance weighting analyses indicated that ENMD significantly increased the risk of PTB (OR = 1.41, 95% CI: 1.18–1.68, *p* < 0.001) after removing outliers. Interestingly, at the genetic level of European ancestry, there is no evidence of increased risk of PTB with T2DM (OR = 1.05, 95% CI: 0.99–1.12, *p* = 0.10), whereas high cholesterol (OR = 0.41, 95% CI: 0.22–0.79, *p* < 0.05), BMI (OR = 0.78, 95% CI: 0.69–0.88, *p* < 0.001) was negatively correlated with the risk of PTB, and LDL‐c showed a weak inverse correlation with PTB (OR = 0.90, 95% CI: 0.81–0.99, *p* = 0.03). Sensitivity analyses confirmed the robustness of these findings.

**Conclusion:**

This MR study provides novel genetic evidence that ENMD significantly elevates PTB risk. Notably, high cholesterol, BMI, and LDL‐c exhibit protective effects against PTB at the genetic level in European ancestry, while T2DM shows no causal association. These findings highlight the complex role of metabolic factors in tuberculosis susceptibility and suggest potential biological mechanisms linking metabolic dysregulation to PTB pathogenesis.

## 1 Introduction

Pulmonary tuberculosis (PTB) is a chronic respiratory infection caused by *Mycobacterium tuberculosis* (*MTB*). Despite substantial progress in PTB control, the morbidity and mortality rates from PTB remain high. In 2021, the World Health Organization (WHO) reported approximately 10.6 million confirmed tuberculosis (TB) cases and 1.13 million deaths worldwide [1]. Current strategies for managing PTB primarily include anti‐TB drug therapy and vaccination. However, significant challenges such as the emergence of drug‐resistant strains and poor patient adherence to treatment underscore an urgent need for further research into the disease's underlying pathophysiology and contributing factors. A study highlighted that the increasing comorbidities of PTB and drug resistance significantly hinder the WHO's objective to reduce TB cases by 50% by 2025 [2]. Therefore, understanding the mechanisms of PTB and its risk factors is vital for improving public health and patient prognosis.

Endocrine, nutritional, and metabolic disease (ENMD) were the exposure factors focused on in our study. In 2022, the WHO indicated that ENMD primarily encompasses endocrine diseases (like thyroid disorders and diabetes), nutritional disorders (such as undernutrition and obesity), and metabolic diseases (such as lipoprotein metabolism disorders) [3]. These conditions significantly affect overall health and contribute to higher morbidity and mortality rates globally. Several observational studies have identified a potential link between these conditions and PTB incidence [4, 5, 6], forming a basis for further investigation into their interrelationships. Individuals with diabetes, for instance, show a significantly elevated risk of developing TB, which may be attributed to impaired immune responses and increased susceptibility to infections [7]. Similarly, malnutrition is recognized as a significant risk factor for PTB, as it compromises immune responses and increases vulnerability to infections. Consequently, investigating the link between ENMD and PTB not only reveals new pathogenic mechanisms but also provides new perspectives for both clinical prevention and treatment of PTB.

A robust methodological approach is necessary to clarify these complex interactions. Mendelian randomization (MR) is a novel approach to etiological research that simulates randomized controlled trials (RCTs) using principles from genetics, genomics, epidemiology, and statistics. This method reduces confounding factors and reverses causation by using genetic variation as an instrumental variable (IV), providing a robust causality assessment [8, 9].

We aim to elucidate the potential role of ENMD‐associated genetic variants in influencing the risk of PTB, providing insights for clinical practice and public health policy. Our study leverages data from large‐scale genome‐wide association studies (GWAS) to identify single nucleotide polymorphisms (SNPs) significantly associated with ENMD. By utilizing summary statistics from the latest and most comprehensive data sets available, we ensure the robustness and generalizability of our findings. Furthermore, we perform extensive sensitivity analyses, including MR‐PRESSO and leave‐one‐out analyses, to validate the reliability of our results.

## 2 Methods

### 2.1 Study Design

**Figure 1:**
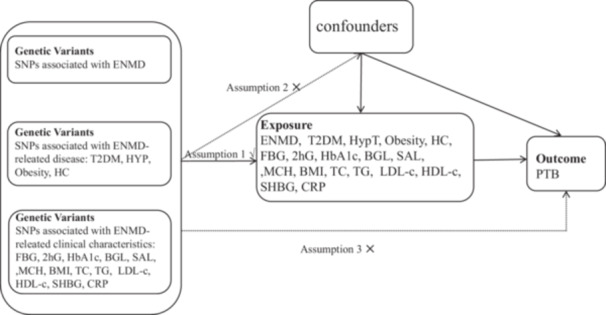
Schematic diagram of the Mendelian randomization (MR) assumptions of the association between endocrine, nutritional, and metabolic disease (ENMD) and pulmonary tuberculosis (PTB). ABL, albumin level; BGL, blood glucose levels; BMI, body mass index; CRP, C‐reactive protein levels; ENMD, endocrine, nutritional, and metabolic disease; FBG, fasting blood glucose; HbA1c, hemoglobin Alc; HC, high cholesterol; HDL‐c, HDL cholesterol; HT, hyperthyroidism; LDL‐c, low density lipoprotein cholesterol; MCH, mean corpuscular hemoglobin; OGTT, oral 2‐h glucose; SHBG, sex hormone‐binding globulin levels; T2DM, type 2 diabetes mellitus; TC, total cholesterol; TG, triglyceride.

The schematic diagram of the MR design is illustrated in Figure 1. We analyzed the impact of exposure factors on PTB through three approaches: the relationship between overall ENMD and PTB, the link between ENMD‐related diseases and PTB, and the connection between ENMD‐related clinical characteristics and PTB. ENMD is associated with several diseases, including endocrine diseases such as type 2 diabetes (T2DM) and hyperthyroidism (HT), as well as nutritional and metabolic diseases like obesity and hypercholesterolemia. ENMD is associated with several important health indicators. There are four blood glucose indicators: fasting blood glucose (FBG), the 2‐h oral glucose tolerance test (OGTT), glycated hemoglobin (HbA1c), and blood glucose levels (BGL). The nutritional indicators include obesity, serum albumin levels (SAL), and body mass index (BMI). For metabolic health, the indicators are triglycerides (TG), total cholesterol (TC), LDL cholesterol (LDL‐c), and HDL cholesterol (HDL‐c). The inflammatory indicator is C‐reactive protein (CRP), while sex hormone‐binding globulin (SHBG) levels are important in understanding both endocrinology and PTB. Genetic variants can be considered IVs only if they meet three assumptions [10]: (1) they directly affect the exposure; (2) they are not associated with any known or unknown confounders; and (3) they influence the outcome solely through the exposure, without involving other pathways.

### 2.2 Data Sources

All genetic variants were sourced from the IEU OpenGWAS project (https://gwas.mrcieu.ac.uk) [11]. Table 1 presents the characteristics of these genetic variants, including trait, GWAS ID, population, sex, sample size (case(*n*)/control(*n*)), year, and PMID. The study population was predominantly of European ancestry. Review and approval by an ethics committee were not needed for this study because all necessary ethical approvals had already been obtained for the original study.

**Table 1:** The characteristics of the GWAS.

Trait	GWAS ID	Population	Sample size(case(*n*)/control(*n*))	Sex	Year	PMID
ENMD	finn‐b‐E4_ENDONUTRMET	European	77,918/140,874	Males and females	2021	NA
PTB	ebi‐a‐GCST90018892	European	895/476,491	NA	2021	34594039
T2DM	ebi‐a‐GCST006867	European	61,714/1178	NA	2018	30054458
HT	ebi‐a‐GCST90018860	European	3557/456,942	NA	2021	34594039
Obesity	ebi‐a‐GCST001475	European	5530/8318	NA	2012	22484627
HC	ebi‐a‐GCST90038690	NA	59,853/424,745	NA	2021	33959723
FBG	ebi‐a‐GCST008032	Hispanic or Latin American	13,556	NA	2019	31217584
2hG	ebi‐a‐GCST90002227	European	63,396	NA	2021	34059833
HbA1c	ieu‐b‐4842	European	45,734	Males and females	2022	NA
BGL	ebi‐a‐GCST90025986	European	400,458	NA	2021	34226706
SAL	ebi‐a‐GCST90025992	European	400,938	NA	2021	34226706
MCH	ebi‐a‐GCST90028995	European	572,863	NA	2018	29892013
BMI	ukb‐b‐19953	European	461,460	Males and females	2018	NA
TC	ebi‐a‐GCST90025953	European	437,878	NA	2021	34226706
TG	ebi‐a‐GCST90018975	European	343,992	NA	2021	34594039
LDL‐c	ieu‐a‐300	Mixed	173,082	Males and females	2013	24097068
HDL‐c	ieu‐b‐109	European	403,943	Males and females	2020	32203549
SHBG	ebi‐a‐GCST90025958	European	397,043	NA	2021	34226706
CRP	ebi‐a‐GCST90029070	European	575,531	NA	2022	35459240

Abbreviations: 2hG, 2‐hour glucose; BGL, blood glucose levels; BMI, body mass index; CRP, C‐reactive protein levels; ENMD, endocrine, nutritional, and metabolic diseases; FBG, fasting blood glucose; HbA1c, glycated haemoglobin HbA1c levels; HC, high cholesterol; HDL‐c, HDL cholesterol; HT, hyperthyroidism; LDL‐c, LDL cholesterol; MCH, mean corpuscular hemoglobin; NA, not available; PTB, pulmonary tuberculosis; SAL, serum albumin levels; SHBG, sex hormone‐binding globulin levels; T2DM, type 2 diabetes mellitus; TC, total cholesterol levels; TG, triglycerides.

#### 2.2.1 Endocrine, Nutritional, and Metabolic Disease (ENMD)

We extracted the latest ENMD data set, which contains 77,918 cases and 140,874 controls from European ancestry, approximately 16,380,466 SNPs.

#### 2.2.2 Pulmonary Tuberculosis (PTB)

Pooled data related to PTB were obtained from a meta‐analysis of the genetic association atlas by Saori et al. for an expanded non‐European population, including approximately 477,386 individuals of European ancestry, with 895 cases and 476,491 controls. A total of 24,189,689 SNPs were included in the analysis [12].

#### 2.2.3 ENMD‐Related Diseases

We obtained pooled data on T2DM from a meta‐analysis of GWAS. With a total of 5,030,727 SNPs, the pooled data consisted of 61,714 cases and 1178 controls from individuals of European ancestry [13]. HT pooled data from the same study as PTB, including 3557 cases, 456,942 controls, and 24,189,279 SNPs [12]. Pooled data on obesity, including 13,848 samples and 2,430,514 SNPs, were obtained from a meta‐analysis of 14 studies comprising 5530 cases of European ancestry (≥ 95th percentile of BMI) and 8318 controls (< 50th percentile of BMI) by Bradfield et al. [14]. Additionally, we obtained summarized data on high cholesterol (HC) from the study by Dönertaş et al., which involved 59,853 cases, 424,745 controls, and 9,587,836 SNPs [15].

#### 2.2.4 ENMD‐Related Clinical Characteristics

The dates for FBG were derived from GWAS summary statistics for the analysis of multi‐ethnic and mixed populations [16], with 13,556 samples and 2,948,9598 SNPs. The large GWAS on OGTT from the meta‐analysis of Glucose and Insulin‐related Traits Consortium (MAGIC), published in 2021, included 281,416 individuals without diabetes (30% non‐European ancestry) [17]. Outcomes from the Within family GWAS consortium were applied to screen genetic instruments for HbA1c, including 45,734 individuals of European ancestry and 9,696,819 SNPs. Data for BGL, SAL, SHBG, and TC were all obtained from a pooled analysis by Barton et al. using the UK Biobank (49,960 individuals) and the remaining cohort (total n ≈ 500,000) [18], with 4,218,897 SNPs for BGL and 4,219,040 SNPs for SAL, 4,232,052 SNPs for TC, and 4,219,040 SNPs for SHBG. We extracted summary statistics on mean corpuscular hemoglobin (MCH) from a large study with 572,863 samples and 11,972,367 SNPs [19]. BMI aggregated data from the MRC‐IEU consortium using output from the GWAS pipeline of Phesant‐derived variables from UKBiobank, comprising 461,460 individuals of European ancestry and 9,851,867 SNPs. TG pooled data from the same study as PTB and HT, including 343,992 individuals and 19,052,580 SNPs [12]. We extracted LDL‐c summary data from the study published by Willer et al. [20] in 2013 on the discovery and refinement of genetic loci associated with lipid levels belonging to the Global Lipids Genetics Consortium, involving 173,082 mixed populations and 2,437,752 SNPs [20]. Large GWAS data for HDL‐c was from the UK Biobank Consortium, involving 403,943 European samples and 12,321,875 SNPs [21]. Summary statistics data on CRP were extracted from a published meta‐analysis of GWAS, including 575,531 individuals of European ancestry, with 10,713,245 SNPs [22].

### 2.3 SNP Selection

Firstly, we selected SNPs that were significantly associated with the exposure in GWAS, with *p* < 5 × 10^−8^ to avoid potential weak IV bias [23]. We then conducted a linkage disequilibrium (LD) test using two parameters: *r*² and kb, to exclude SNPs with *r*² < 0.001 and kb > 10,000. Here, a lower *r*² indicates greater randomness between SNPs, while kb measures the distance between genetic loci; greater distances suggest more independence among SNPs. The *F* statistics were calculated to assess the strength of the IVs by the sample size of the exposure data set, the number of IVs, and *R* ^2^, where *R* ^2^ is the proportion of the variance in the exposure variable explained by the IVs, that is, the square of the correlation coefficient between the IVs and the exposure [24] (*F* value formulae was shown in Supporting Information S1). An *F*‐statistic greater than 10 was considered to be a strong enough threshold for the IVs to significantly mitigate the effects of weak IV bias. Subsequently, harmonization of exposure and outcome through alleles for all SNPs to ensure consistency of effect, if feasible, variants in LD (*r* ^2^ > 0.8) were used to proxy for exposed SNPs that were not available in the outcome data set.

### 2.4 MR Analysis

We primarily used three methods to analyze valid IVs: inverse variance weighted (IVW), MR‐Egger, and weighted median (WM). IVW method serves as the primary approach in MR due to its ability to combine effect estimates from multiple SNPs while maximizing statistical power and providing unbiased causal estimates under the assumption of no horizontal pleiotropy. Additionally, if MR‐Egger and WM yield results consistent with IVW, it will greatly strengthen the persuasiveness of our statistical findings. We evaluated the heterogeneity of SNPs using Cochran's *Q* statistic [25], which indicates heterogeneity when the *p* value is less than 0.05. When Cochran's *Q* test indicated heterogeneity, we used random effects IVW; if not, we opted for fixed‐effects IVW. Sensitivity analyses (e.g., MR‐Egger regression, MR‐PRESSO) enhance result robustness by evaluating horizontal pleiotropy, heterogeneity, and outlier SNPs. We used intercepts from MR‐Egger regression to estimate horizontal pleiotropy among SNP effects. Deviations from zero or a *p* value less than 0.05 suggest the presence of directional pleiotropy. MR‐PRESSO analysis identified outliers and repeated all MR analyses after removing these outlier SNPs. Additionally, leave‐one‐out approach verified the robustness of the results by removing each instrumental variable to check if any single SNP influenced the outcome [26]. Data processing and analysis were performed using R (version 4.3.2), along with Zstats v1.0 (www.zstats.net), mainly using the TwoSampleMR package (version 0.6.5). A two‐tailed *p* value below 0.05 suggests that there is a statistically significant association between the exposure and the outcome.

## 3 Results

### 3.1 Acquisition of IVs

**Figure 2:**
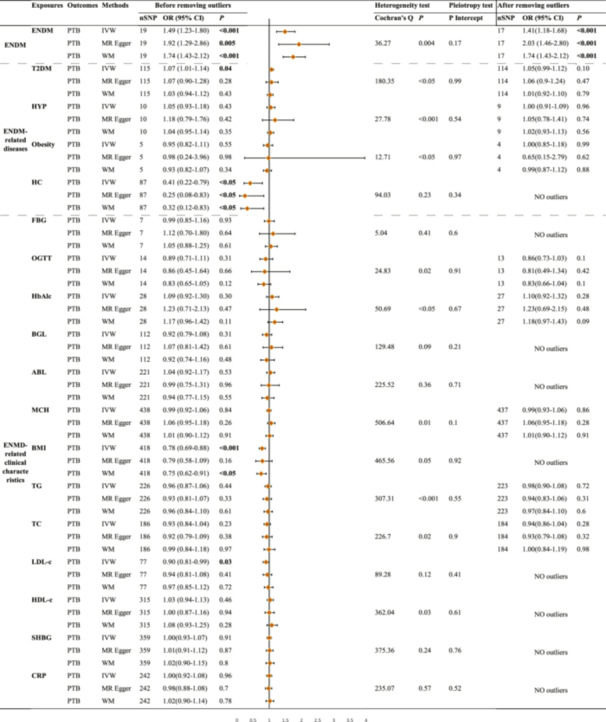
MR results of the causal effect of ENMD, ENMD‐related diseases, and ENMD‐related clinical characteristics on PTB. ABL, albumin level; BGL, blood glucose levels; BMI, body mass index; CRP, C‐reactive protein levels; ENMD, endocrine, nutritional, and metabolic disease; FBG, fasting blood glucose; HbA1c, hemoglobin Alc; HC, high cholesterol; HDL‐c, HDL cholesterol; HT, hyperthyroidism; IVW, inverse variance weighted; LDL‐c, low density lipoprotein cholesterol; MCH, mean corpuscular hemoglobin; MR, Mendelian randomization; OGTT, oral 2‐h glucose; PTB, pulmonary tuberculosis; SHBG, sex hormone‐binding globulin levels; SNP, single nucleotide polymorphisms; TC, total cholesterol; TG, triglyceride; WM, weighted median.

With the screening criteria described above, we first identified SNPs strongly associated with each exposure (Supporting Information S1: Tables S1–S18). However, before performing MR analyses, we needed to remove SNPs that were palindromic with intermediate allele frequencies or had incompatible alleles (Supporting Information S2). Palindromic SNPs are a specific kind of genetic variation. In these cases, the reference sequence is identical to its complement, meaning the SNP reads the same in both directions. For example, SNPs that contain either an A/T pair or a C/G pair are classified as palindromic. Incompatible alleles are SNP variants that may be defined differently across various databases and studies. Both palindromic SNPs and incompatible alleles may lead to errors in data analysis. Finally, Figure 2 displays the number of SNPs associated with each exposure that were included in the MR analyses.

### 3.2 The Causal Effect of ENMD on Pulmonary Tuberculosis

Our results suggested a potential causal association between ENMD and PTB. IVW analysis indicated that ENMD increases the risk of developing PTB (OR = 1.49, 95% CI: 1.23–1.80, *p* < 0.001), with no significant changes in the results when the 2 outlier SNPs were excluded (Figure 2). Other MR methods were consistent with IVW analyses (Figure 2), with no directional pleiotropy (*p* intercept = 0.17) detected, but heterogeneous (*p* = 0.004) (Figure 2, Supporting Information S1: Table S1, Supporting Information S4: Figure S1). More detailed MR‐PRESSO analyses are shown in Supporting Information S3.

### 3.3 The Causal Effect of ENMD‐Related Disease on PTB

The *F*‐statistics of the IVs for T2DM, HT, and HC were all above the threshold of 10, indicating that the IVs could reduce the bias of the estimates (Supporting Information S1: Tables S2–S3, S5). However, *F*‐statistics could not be calculated for obesity‐associated SNPs due to missing EAF values. Cochran's *Q*‐test indicated significant heterogeneity (*p* < 0.05) among SNPs associated with T2DM, HT, and obesity, necessitating the use of a random‐effects model. In contrast, no heterogeneity was observed in SNPs related to HC (*p* = 0.23), which allowed for the application of a fixed‐effects model (Figure 2). MR‐Egger analyses indicated no horizontal pleiotropy in the IVs for all the above‐mentioned four diseases (*p* for intercept > 0.05) (Figure 2).

IVW analyses revealed no significant causality between T2DM and PTB (OR = 1.05, 95% CI: 0.99–1.12, *p* = 0.10), HT and PTB (OR = 1.00, 95% CI: 0.91–1.09, *p* = 0.96), or obesity and PTB (OR = 1.00, 95% CI: 0.85–1.18, *p* = 0.99) after excluding outlier SNPs (Supporting Information S3), however, IVW results revealed a weak positive association between T2DM and PTB (OR = 1.07, 95% CI: 1.01–1.14, *p* = 0.04) before exclusion of outlier SNPs. No outlier SNPs associated with HC were identified, and IVW results indicated a causal association between higher HC levels and lower PTB risk (OR = 0.41, 95% CI: 0.22–0.79, *p* < 0.05), which was also supported by the other two methods. There is a protective causal link between HC levels and PTB. Supporting Information S4: Figures S2–S5 include forest and scatter plots that illustrate the effect of ENMD‐related diseases on PTB risk as determined by MR estimates. It also included funnel plots and leave‐one‐out methods for conducting sensitivity analyses.

### 3.4 The Causal Effect of ENMD‐Related Clinical Characteristics on PTB

Figure 2 presents all results regarding the impact of ENMD‐related clinical characteristics on PTB. First, after excluding outlier SNPs, all three MR analyses showed that four clinical features related to T2DM (FBG, OGTT, HbA1c, BGL) were not significantly causally linked to PTB at the genetic level (*p* > 0.05) (Figure 2). Second, the IVW and WM results indicated that higher BMI levels, associated with nutritional status, correlated with a lower risk of developing PTB (IVW: OR = 0.78, 95% CI: 0.69–0.88, *p* < 0.001). BMI was identified as a protective factor against PTB risk, whereas ABL (IVW: OR = 1.04, 95% CI: 0.92–1.17, *p* = 0.53) and MCH (IVW: OR = 0.99, 95% CI: 0.92–1.06, *p* = 0.84) showed no significant association with PTB risk. Third, a negative correlation between LDL‐c and the risk of PTB was observed in the IVW results (OR = 0.90, 95% CI: 0.81–0.99, *p* = 0.03). Finally, we found no evidence of a causal relationship between SHBG and CRP with PTB (Figure 2).

The *F* statistic for SNPs associated with ENMD clinical characteristics and exposure factors was greater than 10 (Supporting Information S1: Tables S6–S18). However, the *F* statistic could not be calculated for HbA1c due to missing EAF values, and for HDL‐c due to missing sample sizes. The Cochran's *Q* test results indicated no heterogeneity in the SNPs of FBG, BGL, ABL, LDL‐c, SHBG, and CRP (*p* > 0.05), while the remaining SNPs were significantly heterogeneous (Figure 2). MR‐Egger intercept indicated no horizontal pleiotropy for all SNPs (*p* for intercept > 0.05) (Figure 2). We conducted sensitivity analyses using MR‐PRESSO to eliminate outliers (Figure 2 and Supporting Information S3). Additionally, we applied the leave‐one‐out method (Supporting Information S4: Figures S6–S18). Both approaches demonstrated that the results were robust concerning ENMD‐associated clinical characteristics and PTB.

## 4 Discussion

### 4.1 Our Findings

This study examines the link between ENMD and the risk of developing PTB, using an MR approach to reduce common confounding biases in observational studies. Our findings show ENMD patients have a 41% increased risk of PTB compared to non‐ENMD, which suggests that metabolic abnormalities, inflammatory response, and altered nutritional status may contribute to the susceptibility to PTB. Notably, our findings suggest that HC levels (IVW: OR = 0.41), BMI (IVW: OR = 0.78), and LDL‐c (IVW: OR = 0.90) are associated with a lower risk of PTB, which may guide future public health interventions aimed at reducing the burden of this disease.

### 4.2 Effect of T2DM on PTB

This study focuses on the causal relationship between T2DM and PTB in a European ancestry population. We found that T2DM increased the risk of PTB before outlier removal (IVW: 95% CIs 1.01–1.14); however, this effect disappeared afterward (IVW: 95% CIs 0.99–1.12), which is contrary to our conventional knowledge. For example, two systematic reviews and meta‐analyses performed in Sub‐Saharan Africa and China, both countries with a high burden of PTB, have demonstrated that DM is highly associated with PTB [27, 28]. Two previous studies have explored the causal relationship between DM and PTB through MR analyses. One study found that T2DM increased the risk of PTB in Asian populations, which differs from our study population [29]. The second study demonstrated a clear causal relationship between T1DM and PTB in European populations and used SNPs associated with respiratory tuberculosis (probably mainly referring to PTB) [30]. In contrast, our study focused specifically on SNPs that are highly associated with PTB. We hypothesized that T2DM significantly increases the genetic risk of PTB in Asian populations, while T1DM is more likely to raise this risk in European populations. A subgroup analysis indicated that the incidence of T2DM‐PTB was significantly higher in Asia (187.20 cases per 100,000) than in Europe (105.01 cases per 100,000) [31], which supports our hypothesis. Individuals with DM may experience immune dysregulation due to hyperglycemia and insulin resistance [32]. This can weaken their immune response to *MTB* and potentially lead to PTB [32].

### 4.3 Effect of Cholesterol and BMI on PTB

It was interesting that our study found high cholesterol, BMI, and LDL‐c to be protective factors for PTB, and notably, some of the published studies were consistent with our findings. A small RCT from Mexico reported a higher sterilization rate in patients on a higher cholesterol‐rich diet (800 mg/day) than in those on a lower cholesterol‐rich diet (250 mg/day) in a routine antituberculosis program [33]. Jo et al. also found an increased risk of tuberculosis associated with lower cholesterol levels in subjects under 65 years old who were nondiabetic, nonobese, and not on statins [6].

PTB is a consumption disease that often presents with malnutrition and weight loss. Previous studies have suggested that these symptoms may result from reduced protein intake, poor nutrient absorption, or increased metabolism due to inflammation [34, 35]. However, our analysis did not reveal any SNPs that are associated with albumin (*p* = 0.53) or mean corpuscular hemoglobin (*p* = 0.86) impacting PTB at the genetic levels. In contrast, a higher BMI was identified as a protective factor against this disease. Support for higher BMI levels reducing the risk of PTB came from South Korea, however, it also concluded that a very high BMI (BMI > 30 kg/m²) does not provide any protective effect against PTB in young Korean women or individuals with diabetes [36].

Cholesterol is a fat‐soluble lipid that is transported in the blood primarily as LDL‐C and HDL‐C. Currently, there is a significant focus on cholesterol's effects on the cardiovascular system. HDL‐C is often referred to as “good” cholesterol because it facilitates the reverse transport of excess cholesterol from peripheral tissues, such as adipose tissue, back to the liver for excretion or reuse. In contrast, LDL‐C promotes atherosclerosis and is considered “bad” cholesterol. This understanding stems from recent findings that individuals with ectopic fat deposits—such as visceral fat in the liver and heart—tend to have lower levels of HDL‐C [37, 38]. It has been suggested that total cholesterol may lower PTB risk [39], which is consistent with our study.

We discovered that high cholesterol, higher BMI, and elevated LDL‐c may protect against PTB. These suggest that higher levels of peripheral or subcutaneous adipose tissue could be linked to a lower risk of the disease, as patients with PTB typically have less subcutaneous adipose tissue. Adipose tissue serves as both an essential energy reserve and an active endocrine organ, secreting various cytokines and hormones that play roles in immune responses [40]. Additionally, cholesterol is a crucial component of all cell membranes, including those of adipocytes, and is vital for maintaining the normal immune function of subcutaneous adipocytes. BMI typically indicates an individual's overall adiposity. Higher BMI is often associated with increased visceral adiposity, which is linked to various chronic diseases [40]. In contrast, elevated peripheral adiposity generally poses a lower health risk. Research suggested that visceral and subcutaneous adipose tissues contribute differently to disease risk [40]. A study found that lower BMI in PTB populations was more likely to develop drug‐resistant tuberculosis [41]. Hormones secreted by visceral adipose tissue, such as IL‐6 and plasminogen activator inhibitors, travel through the portal vein to the liver. These hormones can affect the liver's metabolic functions. In contrast, leptin, which is secreted by subcutaneous adipose tissue, indicates energy sufficiency, not metabolic excess [42]. Lowering cholesterol levels has been found to reduce T cell proliferation and function [43]. This decrease can lower the body's resistance to *MTB* since cholesterol is a vital part of T cell membranes and is essential for their immune function.

### 4.4 The Evidence for Selective Sex Hormone‐Binding Globulin and C‐Reactive Protein Effects on PTB

SHBG is a protein closely related to both ENMD and PTB. Metabolic syndrome is the major clinical characteristic of ENMD, a common metabolic disorder that is highly associated with disorders of sex hormone regulation [44]. SHBG is a serum protein that can specifically bind to androgens and estrogens [45]. SHBG levels are independently associated with the risk of metabolic syndrome such as diabetes, nonalcoholic fatty liver disease, and hypertension [46, 47, 48, 49]. One study also demonstrated that SHBG, a novel serum marker, is highly associated with PTB [50]. However, observational studies do not clearly establish a causal relationship between SHBG and TB. In contrast, our MR analysis found no evidence of such a relationship from the perspective of genetic variation.

ENMD and PTB often involve inflammatory responses. CRP, an acute‐phase reactant, is elevated in most inflammatory and infectious diseases, including PTB. Although CRP is less sensitive for identifying PTB [51], the WHO recommended it as a screening test for HIV‐infected individuals in 2021. This recommendation is due to its low cost and wide availability in clinical settings [52, 53]. An observational study from Korea showed a positive correlation between CRP concentration and metabolic syndrome, as well as significantly higher CRP levels in PTB patients compared to the general population [54]. Evidence suggests that elevated CRP levels are more common in PTB patients; however, the specificity of CRP in this context is weak, and the causal relationship remains unclear. Consequently, we investigated the potential causal relationship between CRP and PTB by examining genetic variations, and our findings indicate no definitive causal link between the two.

### 4.5 Clinical Implications

The findings of this study have significant clinical implications for the management and prevention of PTB in patients with ENMD. The demonstrated causal relationship between ENMD and increased risk of PTB suggests that clinicians should be vigilant in monitoring and managing metabolic health in patients susceptible to PTB. This may lead to targeted screening programs for early detection of PTB in populations with metabolic disorders. Additionally, improving cholesterol levels and BMI could reduce PTB incidence. These insights underscore the importance of integrated care approaches that address both metabolic health and infectious disease prevention, which could ultimately improve patient outcomes and reduce healthcare burdens. Future research should focus on validating these findings in diverse populations and exploring the underlying mechanisms to refine preventive strategies further.

### 4.6 Limitations

A key limitation of our study is that we relied on genetic instruments primarily from samples of European descent. Population stratification may restrict how applicable our findings are to other ethnic groups. While the use of MR methods helps to mitigate confounding and reverse causation, the selection of SNPs as IVs is inherently constrained by the availability and quality of GWAS data. Additionally, the heterogeneity among SNPs, weak IVs bias, and the variations of SNPs between data sets from different batches may lead to inconsistencies in effect estimates, thereby compromising the accuracy of our conclusions. The absence of clinical validation analyses is critical, as it can significantly compromise the applicability of the results in clinical practice. Moreover, advanced methods to address pleiotropy and heterogeneity should be incorporated to ensure the validity of MR analyses [55].

## 5 Conclusion

This MR study provides novel genetic evidence that ENMD significantly elevates PTB risk. Notably, high cholesterol, BMI, and LDL‐c exhibit protective effects against PTB at the genetic level in European ancestry, while T2DM shows no causal association. These findings highlight the complex role of metabolic factors in tuberculosis susceptibility and suggest potential biological mechanisms linking metabolic dysregulation to PTB pathogenesis.

## Author Contributions

**Yan Gao:** methodology, software, formal analysis, investigation, visualization, writing – original draft. **Yiguo Wang:** software, methodology, and investigation. **Jinwen Su:** investigation and supervision. **Chunxia Zhang:** investigation and supervision. **Qiming Zhang:** investigation, supervision, validation, writing – review and editing. **Zhi Chen:** conceptualization, writing – review and editing, supervision, and funding acquisition.

## Ethics Statement

Ethical approval and informed consent had been obtained in all original studies cited in our study.

## Conflicts of Interest

The authors declare no conflicts of interest.

## Transparency Statement

The lead author Zhi Chen affirms that this manuscript is an honest, accurate, and transparent account of the study being reported; that no important aspects of the study have been omitted; and that any discrepancies from the study as planned (and, if relevant, registered) have been explained.

## Supporting information

S1_File: Data on the effect of various exposure factors on PTB.

S2_File: Removing SNPs for being palindromic with intermediate allele frequencies or for incompatible alleles before MR analyses.

S3_File: MR‐PRESSO analyses for the association between ENMD‐related exposure and PTB.

S4_File: Four types of causal effect graphs of SNPs associated with ENMD on PTB: a, forest plot for the causal effects of ENMD on PTB; b, scatter plot for the causal effect of ENMD on PTB; c, funnel plot to assess heterogeneity; d, forest plot for leave‐one‐out analysis.

## Data Availability

The data set(s) supporting the conclusions of this article is(are) available in the (IEU OpenGWAS project) repository, (unique persistent identifier and hyperlink to data set(s) in http://gwas.mrcieu.ac.uk/). The authors confirm that the data supporting the findings of this study are available within the article and its Supporting Information S1.

## References

[1] J. Chakaya , E. Petersen , R. Nantanda , et al., “The WHO Global Tuberculosis 2021 Report—Not so Good News and Turning the Tide Back to End TB,” Supplement, International Journal of Infectious Diseases 124, no. S1 (2022): S26–S29, 10.1016/j.ijid.2022.03.011. 35321845 PMC8934249

[2] A. Vasiliu , L. Martinez , R. K. Gupta , et al., “Tuberculosis Prevention: Current Strategies and Future Directions,” Clinical Microbiology and Infection 30 (2024): 1123–1130, 10.1016/j.cmi.2023.10.023. 37918510 PMC11524220

[3] World Health Organization , “ICD‐11 for Mortality and Morbidity Statistics (version:2022‐02)[EB/OL].(2022‐5)[2022‐8‐22],” https://icd.who.int/browse11/l-m/en.

[4] Y. Feng , J. Guo , S. Luo , and G. Zhou , “Risk Factors for Pulmonary Tuberculosis With Tracheobronchial Tuberculosis: Propensity Score Matching Analysis,” Infection and Drug Resistance 17 (2024): 3145–3151, 10.2147/IDR.S470886. 39050839 PMC11268757

[5] I. O. Elfaky , T. H. Merghani , I. A. Elmubarak , and A. H. Ahmed , “Nutritional Status and Patterns of Anemia in Sudanese Adult Patients With Active Pulmonary Tuberculosis: A Cross‐Sectional Study,” International Journal of Mycobacteriology 12 (2023): 73–76, 10.4103/ijmy.ijmy_14_23. 36926766

[6] Y. S. Jo , K. Han , D. Kim , et al., “Relationship Between Total Cholesterol Level and Tuberculosis Risk in a Nationwide Longitudinal Cohort,” Scientific Reports 11 (2021): 16254, 10.1038/s41598-021-95704-1. 34376753 PMC8355278

[7] S. G. Smith , R. Bowness , and J. M. Cliff , “Host‐Directed Therapy in Diabetes and Tuberculosis Comorbidity Toward Global Tuberculosis Elimination,” International Journal of Infectious Diseases 155 (2025): 107877, 10.1016/j.ijid.2025.107877. 40068707

[8] D. A. Lawlor , R. M. Harbord , J. A. C. Sterne , N. Timpson , and G. Davey Smith , “Mendelian Randomization: Using Genes as Instruments for Making Causal Inferences in Epidemiology,” Statistics in Medicine 27 (2008): 1133–1163, 10.1002/sim.3034. 17886233

[9] G. Davey Smith and S. Ebrahim , “‘Mendelian Randomization’: Can Genetic Epidemiology Contribute to Understanding Environmental Determinants of Disease?,” International Journal of Epidemiology 32, no. 1 (2003): 1–22, 10.1093/ije/dyg070. 12689998

[10] F. P. Hartwig , N. M. Davies , G. Hemani , and G. Davey Smith , “Two‐Sample Mendelian Randomization: Avoiding the Downsides of a Powerful, Widely Applicable but Potentially Fallible Technique,” International Journal of Epidemiology 45 (2016): 1717–1726, 10.1093/ije/dyx028. 28338968 PMC5722032

[11] B. Elsworth , M. Lyon , T. Alexander , et al., “The MRC IEU Open GWAS Data Infrastructure,” bioRxiv 35 (2020): 99.

[12] S. Sakaue , M. Kanai , Y. Tanigawa , et al., “A Cross‐Population Atlas of Genetic Associations for 220 Human Phenotypes,” Nature Genetics 53, no. 10 (2021): 1415–1424, 10.1038/s41588-021-00931-x. 34594039 PMC12208603

[13] A. Xue , Y. Wu , Z. Zhu , et al., “Genome‐Wide Association Analyses Identify 143 Risk Variants and Putative Regulatory Mechanisms for Type 2 Diabetes,” Nature Communications 9, no. 1 (July 2018): 2941, 10.1038/s41467-018-04951-w. PMC6063971 30054458

[14] J. P. Bradfield , H. R. Taal , N. J. Timpson , et al., “A Genome‐Wide Association Meta‐Analysis Identifies New Childhood Obesity Loci,” Nature Genetics 44, no. 5 (2012): 526–531, 10.1038/ng.2247. 22484627 PMC3370100

[15] H. M. Dönertaş , D. K. Fabian , M. Fuentealba , L. Partridge , and J. M. Thornton , “Common Genetic Associations Between Age‐Related Diseases,” Nature Aging 1, no. 4 (2021): 400–412, 10.1038/s43587-021-00051-5. 33959723 PMC7610725

[16] G. L. Wojcik , M. Graff , K. K. Nishimura , et al., “Genetic Analyses of Diverse Populations Improves Discovery for Complex Traits,” Nature 570, no. 7762 (2019): 514–518, 10.1038/s41586-019-1310-4. 31217584 PMC6785182

[17] J. Chen , C. N. Spracklen , G. Marenne , et al., “The Trans‐Ancestral Genomic Architecture of Glycemic Traits,” Nature Genetics 53, no. 6 (2021): 840–860, 10.1038/s41588-021-00852-9. 34059833 PMC7610958

[18] A. R. Barton , M. A. Sherman , R. E. Mukamel , and P. R. Loh , “Whole‐Exome Imputation Within UK Biobank Powers Rare Coding Variant Association and Fine‐Mapping Analyses,” Nature Genetics 53, no. 8 (2021): 1260–1269, 10.1038/s41588-021-00892-1. 34226706 PMC8349845

[19] P. R. Loh , G. Kichaev , S. Gazal , A. P. Schoech , and A. L. Price , “Mixed‐Model Association for Biobank‐Scale Datasets,” Nature Genetics 50, no. 7 (2018): 906–908, 10.1038/s41588-018-0144-6. 29892013 PMC6309610

[20] C. J. Willer , E. M. Schmidt , S. Sengupta , et al., “Discovery and Refinement of Loci Associated With Lipid Levels,” Nature Genetics 45, no. 11 (2013): 1274–1283, 10.1038/ng.2797. 24097068 PMC3838666

[21] T. G. Richardson , E. Sanderson , T. M. Palmer , et al., “Evaluating the Relationship Between Circulating Lipoprotein Lipids and Apolipoproteins With Risk of Coronary Heart Disease: A Multivariable Mendelian Randomisation Analysis,” PLoS Medicine 17, no. 3 (2020): e1003062, 10.1371/journal.pmed.1003062. 32203549 PMC7089422

[22] S. Said , R. Pazoki , V. Karhunen , et al., “Genetic Analysis of Over Half a Million People Characterises C‐Reactive Protein Loci,” Nature Communications 13, no. 1 (2022): 2198, 10.1038/s41467-022-29650-5. PMC9033829 35459240

[23] S. Burgess and S. G. Thompson , CRP CHD Genetics Collaboration , “Avoiding Bias From Weak Instruments in Mendelian Randomization Studies,” International Journal of Epidemiology 40, no. 3 (2011): 755–764, 10.1093/ije/dyr036. 21414999

[24] N. Papadimitriou , N. Dimou , K. K. Tsilidis , et al., “Physical Activity and Risks of Breast and Colorectal Cancer: A Mendelian Randomisation Analysis,” Nature Communications 11, no. 1 (2020): 597, 10.1038/s41467-020-14389-8. PMC6992637 32001714

[25] J. F. Cohen , M. Chalumeau , R. Cohen , D. A. Korevaar , B. Khoshnood , and P. M. M. Bossuyt , “Cochran's Q Test Was Useful to Assess Heterogeneity in Likelihood Ratios in Studies of Diagnostic Accuracy,” Journal of Clinical Epidemiology 68 (2015): 299–306, 10.1016/j.jclinepi.2014.09.005. 25441698

[26] F. Wu , Y. Huang , J. Hu , and Z. Shao , “Mendelian Randomization Study of Inflammatory Bowel Disease and Bone Mineral Density,” BMC Medicine 18, no. 1 (2020): 312, 10.1186/s12916-020-01778-5. 33167994 PMC7654011

[27] I. Obels , S. Ninsiima , J. A. Critchley , and P. Huangfu , “Tuberculosis Risk Among People With Diabetes Mellitus in Sub‐Saharan Africa: A Systematic Review,” Tropical Medicine & International Health 27, no. 4 (2022): 369–386, 10.1111/tmi.13733. 35146851 PMC9303199

[28] Q. Du , L. Wang , Q. Long , Y. Zhao , and A. S. Abdullah , “Systematic Review and Meta‐Analysis: Prevalence of Diabetes Among Patients With Tuberculosis in China,” Tropical Medicine & International Health 26, no. 12 (2021): 1553–1559, 10.1111/tmi.13686. 34637179

[29] S. Chen , W. Zhang , Z. Zheng , et al., “Unraveling Genetic Causality Between Type 2 Diabetes and Pulmonary Tuberculosis on the Basis of Mendelian Randomization,” Diabetology & Metabolic Syndrome 15, no. 1 (2023): 228, 10.1186/s13098-023-01213-8. 37950319 PMC10636918

[30] Y. Jiang , W. Zhang , M. Wei , et al., “Associations Between Type 1 Diabetes and Pulmonary Tuberculosis: A Bidirectional Mendelian Randomization Study,” Diabetology & Metabolic Syndrome 16, no. 1 (2024): 60, 10.1186/s13098-024-01296-x. 38443967 PMC10913601

[31] Q. Wu , Y. Liu , Y. B. Ma , K. Liu , and S. H. Chen , “Incidence and Prevalence of Pulmonary Tuberculosis Among Patients With Type 2 Diabetes Mellitus: A Systematic Review and Meta‐Analysis,” Annals of Medicine 54, no. 1 (2022): 1657–1666, 10.1080/07853890.2022.2085318. 35703920 PMC9225779

[32] N. Martinez and H. Kornfeld , “Diabetes and Immunity to Tuberculosis,” European Journal of Immunology 44, no. 3 (2014): 617–626, 10.1002/eji.201344301. 24448841 PMC4213860

[33] C. Pérez‐Guzmán , M. H. Vargas , F. Quiñonez , N. Bazavilvazo , and A. Aguilar , “A Cholesterol‐Rich Diet Accelerates Bacteriologic Sterilization in Pulmonary Tuberculosis,” Chest 127, no. 2 (2005): 643–651, 10.1378/chest.127.2.643. 15706008

[34] N. A. Tellez‐Navarrete , L. A. Ramon‐Luing , M. Muñoz‐Torrico , I. A. Osuna‐Padilla , and L. Chavez‐Galan , “Malnutrition and Tuberculosis: The Gap Between Basic Research and Clinical Trials,” Journal of Infection in Developing Countries 15, no. 3 (2021): 310–319, 10.3855/jidc.12821. 33839703

[35] S. Kant , H. Gupta , and S. Ahluwalia , “Significance of Nutrition in Pulmonary Tuberculosis,” Critical Reviews in Food Science and Nutrition 55, no. 7 (2015): 955–963, 10.1080/10408398.2012.679500. 24915351

[36] S. J. Kim , S. Ye , E. Ha , and E. M. Chun , “Association of Body Mass Index With Incident Tuberculosis in Korea,” PLoS One 13, no. 4 (2018): e0195104, 10.1371/journal.pone.0195104. 29668698 PMC5906015

[37] K. A. Britton , J. M. Massaro , J. M. Murabito , B. E. Kreger , U. Hoffmann , and C. S. Fox , “Body Fat Distribution, Incident Cardiovascular Disease, Cancer, and All‐Cause Mortality,” Journal of the American College of Cardiology 62, no. 10 (2013): 921–925, 10.1016/j.jacc.2013.06.027. 23850922 PMC4142485

[38] A. L. Borel , J. A. Nazare , J. Smith , et al., “Visceral, Subcutaneous Abdominal Adiposity and Liver Fat Content Distribution in Normal Glucose Tolerance, Impaired Fasting Glucose and/or Impaired Glucose Tolerance,” International Journal of Obesity 39, no. 3 (2015): 495–501, 10.1038/ijo.2014.163. 25179244

[39] Z. Du , Y. Ren , J. Wang , et al., “The Potential Association Between Metabolic Disorders and Pulmonary Tuberculosis: A Mendelian Randomization Study,” European Journal of Medical Research 29, no. 1 (2024): 277, 10.1186/s40001-024-01845-0. 38725045 PMC11080151

[40] E. E. Kershaw and J. S. Flier , “Adipose Tissue as an Endocrine Organ,” Journal of Clinical Endocrinology & Metabolism 89, no. 6 (2004): 2548–2556, 10.1210/jc.2004-0395. 15181022

[41] C. Vyawahare , S. Mukhida , S. Khan , N. R. Gandham , S. Kannuri , and S. Bhaumik , “Assessment of Risk Factors Associated With Drug‐Resistant Tuberculosis in Pulmonary Tuberculosis Patients,” Indian Journal of Tuberculosis 71, no. S1 (2024): S44–S51, 10.1016/j.ijtb.2023.07.007. 39067954

[42] J. S. Flier , “Clinical Review 94: What's in a Name? In Search of Leptin's Physiologic Role,” Journal of Clinical Endocrinology and Metabolism 83, no. 5 (1998): 1407–1413, 10.1210/jcem.83.5.4779. 9589630

[43] K. Y. Chyu , W. M. Lio , P. C. Dimayuga , et al., “Cholesterol Lowering Modulates T Cell Function In Vivo and In Vitro,” PLoS One 9, no. 3 (2014): e92095, 10.1371/journal.pone.0092095. 24647529 PMC3960213

[44] J. Zhang , W. Gu , S. Zhai , et al., “Phthalate Metabolites and Sex Steroid Hormones in Relation to Obesity in US Adults: Nhanes 2013–2016,” Frontiers in Endocrinology 15 (2024): 1340664, 10.3389/fendo.2024.1340664. 38524635 PMC10957739

[45] M. A. Thaler , V. Seifert‐Klauss , and P. B. Luppa , “The Biomarker Sex Hormone‐Binding Globulin—From Established Applications to Emerging Trends in Clinical Medicine,” Best Practice & Research Clinical Endocrinology & Metabolism 29 (2015): 749–760, 10.1016/j.beem.2015.06.005. 26522459

[46] N. Li , C. Huang , B. Lan , et al., “Association of Gonadal Hormones and Sex Hormone Binding Globulin With Risk of Diabetes: A Cohort Study in Middle‐Aged and Elderly Chinese Males,” International Journal of Clinical Practice 75 (2021): e14008, 10.1111/ijcp.14008. 33400357

[47] J. Luo , Q. Chen , T. Shen , et al., “Association of Sex Hormone‐Binding Globulin With Nonalcoholic Fatty Liver Disease in Chinese Adults,” Nutrition & Metabolism 15 (2018): 79, 10.1186/s12986-018-0313-8. 30455723 PMC6225668

[48] Q. Yang , Z. Li , W. Li , et al., “Association of Total Testosterone, Free Testosterone, Bioavailable Testosterone, Sex Hormone‐Binding Globulin, and Hypertension,” Medicine 98 (2019): e15628, 10.1097/MD.0000000000015628. 31096475 PMC6531235

[49] P. I. H. G. Simons , O. Valkenburg , M. P. H. van de Waarenburg , et al., “Serum Sex Hormone‐Binding Globulin Is a Mediator of the Association Between Intrahepatic Lipid Content and Type 2 Diabetes: The Maastricht Study,” Diabetologia 66, no. 1 (2023): 213–222, 10.1007/s00125-022-05790-7. 36114428 PMC9729158

[50] C. Li , X. He , H. Li , et al., “Discovery and Verification of Serum Differential Expression Proteins for Pulmonary Tuberculosis,” Tuberculosis 95, no. 5 (2015): 547–554, 10.1016/j.tube.2015.06.001. 26276261

[51] D. Chanda , M. Kasanga , R. Chanda , and F. Cobelens , “C‐Reactive Protein: Another Addition to Our Armamentarium Against Tuberculosis?,” Lancet Global Health 11, no. 5 (2023): e636–e637, 10.1016/S2214-109X(23)00175-4. 37061298

[52] WHO , “Global Tuberculosis Report,” (World Health Organization, 2022).

[53] WHO , “WHO Consolidated Guidelines on Tuberculosis Module 2: Screening Systematic Screening for Tuberculosis Disease,” published March 22, 2021, https://www.who.int/publications/i/item/9789240022676. 33822560

[54] H. Jeong , S. Y. Baek , S. W. Kim , et al., “C Reactive Protein Level as a Marker for Dyslipidaemia, Diabetes and Metabolic Syndrome: Results From the Korea National Health and Nutrition Examination Survey,” BMJ Open 9, no. 8 (2019): e029861, 10.1136/bmjopen-2019-029861. PMC6720331 31473619

[55] N. M. Davies , M. V. Holmes , and G. Davey Smith , “Reading Mendelian Randomisation Studies: A Guide, Glossary, and Checklist for Clinicians,” British Medical Journal 362 (2018): k601, 10.1136/bmj.k601. 30002074 PMC6041728

