# Supplementary material for: Assessing Causality Between Endocrine, Nutritional, and Metabolic Disease and Pulmonary Tuberculosis: A Mendelian Randomization Study
Source: Health Sci Rep. 2025 May 29;8(6):e70875. doi: 10.1002/hsr2.70875 (PMC12122389; doi:10.1002/hsr2.70875)
Supplement: Supplementary file 1 — S1_File: Data on the effect of various exposure factors on PTB. [file HSR2-8-e70875-s004.pdf]

Calculation of F-statistics and R^2 measures

R^2=(2*EAF*(1 -EAF)*β^2)/((2*β^2*EAF*(1 - EAF)*β^2)+(2*EAF*(1-EAF)*N*se^2))

F=R^2*(N-2)/(1-R^2)

EAF: effect allele frequency of the exposure

β: the effect size of the exposure genetic variant

N: the sample size of the exposure

Se: the standard error of the effect size of the exposure genetic variant

R^2: the proportion of variability in the exposure that is explained by the genotype

Table S1. Published associations of ENMD on PTB

SNP	Exposure	EA	OA	GX	GX(SE)	EAF-exp	Pval-exp	GY	GY(SE)	EAF-out	Pval-outcome
rs11065987	**ENMD**	G	A	0.0582	0.0076	0.3828	2.08E-14	0.0661	0.0492	0.405226	0.1791
rs12555274	**ENMD**	C	G	0.0466	0.0083	0.2766	1.83E-08	0.0175	0.0167	0.28846	0.2938
rs1574285	**ENMD**	T	G	-0.0421	0.0074	0.5595	1.59E-08	-0.0216	0.0161	0.582394	0.1788
rs2237896	**ENMD**	A	G	-0.0772	0.0141	0.07531	4.25E-08	0.0167	0.0168	0.257072	0.3215
rs2395490	**ENMD**	G	A	0.0623	0.0076	0.5842	2.43E-16	0.0785	0.0161	0.554832	1.07E-06
rs34203909	**ENMD**	T	G	0.0508	0.0091	0.2091	2.51E-08	0.0066	0.0241	0.180566	0.785199
rs34811474	**ENMD**	A	G	-0.0499	0.0088	0.2299	1.56E-08	0.0093	0.0572	0.231494	0.8703
rs35239243	**ENMD**	T	C	0.0579	0.0102	0.1558	1.55E-08	0.0059	0.0196	0.175249	0.7634
rs4743130	**ENMD**	T	C	0.0788	0.0078	0.6536	3.17E-24	0.0014	0.028	0.737507	0.961
rs56348580	**ENMD**	C	G	-0.0451	0.0083	0.2827	4.76E-08	0.0232	0.0531	0.301038	0.6625
rs6679677	**ENMD**	A	C	0.16	0.0104	0.1472	2.64E-53	0.1106	0.0695	0.115101	0.1114
rs6946369	**ENMD**	T	C	0.0674	0.0109	0.1333	6.25E-10	0.0369	0.0355	0.136552	0.2979
rs72649996	**ENMD**	T	G	-0.0934	0.017	0.0506	4.19E-08	-0.0482	0.0358	0.0530596	0.1779
rs76895963	**ENMD**	G	T	-0.1287	0.0222	0.03114	6.80E-09	0.2973	0.152	0.0238735	0.0505196
rs7754251	**ENMD**	C	G	0.0521	0.0074	0.4492	2.53E-12	-0.0177	0.0167	0.489428	0.2898
rs7766070	**ENMD**	A	C	0.0543	0.0078	0.3322	4.15E-12	-0.0023	0.0161	0.321619	0.8857
rs7903146	**ENMD**	T	C	0.0914	0.0093	0.1996	6.04E-23	-0.0051	0.0333	0.204523	0.8782
rs7996350	**ENMD**	C	G	0.0447	0.0077	0.3541	6.29E-09	0.0067	0.016	0.389856	0.6742
rs9273363	**ENMD**	A	C	0.1711	0.0087	0.2794	2.06E-85	0.1009	0.0163	0.327979	6.23E-10
rs9275577	**ENMD**	T	A	0.1109	0.0087	0.5073	4.72E-37	-0.0135	0.0168	0.568935	0.4199
rs9931494	**ENMD**	G	C	0.0579	0.0075	0.4129	1.27E-14	-0.011	0.0184	0.362763	0.5509

ENMD:endocrine, nutritional and metabolic disease. EA: effect allele. OA: other allele. EAF: EA frequency. GX: beta-exposure. GX(SE): standard error of GX. GY: beta-outcome. GY(SE): standard error of GY.

Table S2. Published associations of T2DM on PTB

SNP	Exposure	EA	OA	GX	GX(SE)	Pval-exp	F	GY	GY(SE)	Pval-outcome
rs10077431	**T2DM**	A	C	-0.0487	0.0089	4.75E-08	29.94084811	0.0413	0.0307	0.1781
rs10087241	**T2DM**	A	G	-0.0475	0.008	2.80E-09	35.25278516	0.0201	0.0273	0.4628
rs10100265	**T2DM**	C	A	-0.0491	0.0079	6.29E-10	38.62735675	0.0037	0.0168	0.8278
rs10114341	**T2DM**	C	T	-0.0409	0.0072	1.15E-08	32.26768526	-0.0434	0.0206	0.0347696
rs10401969	**T2DM**	C	T	0.0921	0.0133	4.13E-12	47.95149672	0.0208	0.0269	0.4397
rs1050226	**T2DM**	G	A	-0.0491	0.0074	3.34E-11	44.02361824	0.0159	0.016	0.3198
rs1061813	**T2DM**	A	G	-0.0429	0.0073	3.37E-09	34.53464954	-0.044	0.0254	0.0833508
rs1063355	**T2DM**	G	T	0.0709	0.0079	3.72E-19	80.54238335	0.0951	0.0162	4.44E-09
rs10740322	**T2DM**	A	G	0.0477	0.0085	2.11E-08	31.49090165	0.009	0.0179	0.6158
rs10811661	**T2DM**	C	T	-0.1569	0.0098	4.13E-58	256.3184834	-0.0261	0.0163	0.1094
rs10830963	**T2DM**	G	C	0.0909	0.008	5.85E-30	129.1023006	0.0068	0.016	0.672
rs10842994	**T2DM**	T	C	-0.0755	0.0091	1.02E-16	68.83309659	0.0131	0.0203	0.5183
rs10974438	**T2DM**	C	A	0.0591	0.0075	3.01E-15	62.09242536	0.0205	0.0163	0.2081
rs11098676	**T2DM**	C	T	0.054	0.0096	2.03E-08	31.63961881	-0.0399	0.0315	0.2056
rs11107116	**T2DM**	T	G	0.0467	0.0085	3.75E-08	30.18436881	0.0379	0.0174	0.0294503
rs1111875	**T2DM**	T	C	-0.0948	0.0072	3.61E-39	173.3555981	-0.0407	0.0172	0.0180501
rs11257655	**T2DM**	T	C	0.0737	0.0087	1.97E-17	71.76003791	-0.021	0.0165	0.2036
rs1127655	**T2DM**	T	C	-0.0438	0.0079	2.47E-08	30.73832707	-8.00E-04	0.0163	0.9628
rs11708067	**T2DM**	G	A	-0.0965	0.0086	5.93E-29	125.9052713	-0.1924	0.0619	0.00187802
rs11925227	**T2DM**	A	G	-0.0534	0.0095	2.25E-08	31.59522791	-0.0092	0.0246	0.706999
rs11926707	**T2DM**	C	T	0.0463	0.0082	1.69E-08	31.88015808	-0.0064	0.018	0.7228
rs12088739	**T2DM**	G	A	-0.0884	0.013	9.79E-12	46.23852954	-0.0069	0.0291	0.8127
rs12299509	**T2DM**	G	A	0.0467	0.0073	2.09E-10	40.92363758	0.0079	0.0158	0.617401
rs12617659	**T2DM**	T	C	-0.0685	0.0103	2.83E-11	44.22755004	0.0192	0.0231	0.4062
rs12910825	**T2DM**	G	A	0.0517	0.0074	2.16E-12	48.80944121	0.0934	0.0486	0.05465
rs12945601	**T2DM**	C	T	-0.048	0.008	1.72E-09	35.99885518	0.0112	0.0222	0.615
rs12970134	**T2DM**	A	G	0.0555	0.008	5.31E-12	48.12737572	3.00E-04	0.0211	0.9874
rs13234269	**T2DM**	A	T	-0.0583	0.0078	6.98E-14	55.86426551	0.0245	0.0174	0.1579
rs13239186	**T2DM**	T	C	0.0539	0.0085	2.70E-10	40.20924031	0.0338	0.022	0.1246
rs13330951	**T2DM**	G	A	-0.0456	0.0081	1.54E-08	31.69172192	0.0105	0.0217	0.6285
rs13389219	**T2DM**	T	C	-0.0722	0.0074	2.11E-22	95.19127518	-0.062	0.0262	0.0179602
rs1359790	**T2DM**	A	G	-0.0796	0.008	2.80E-23	98.99935167	0.0095	0.0177	0.5926
rs1496653	**T2DM**	G	A	-0.0769	0.0088	2.57E-18	76.36133709	-0.0472	0.021	0.0249597
rs1552224	**T2DM**	C	A	-0.1034	0.0101	8.64E-25	104.8056073	0.0032	0.0347	0.9268
rs16988333	**T2DM**	G	A	-0.0745	0.013	9.17E-09	32.84067159	-0.0551	0.085	0.5165
rs17086692	**T2DM**	T	G	-0.0467	0.0084	2.48E-08	30.90732209	-0.0082	0.0183	0.655101
rs17168486	**T2DM**	T	C	0.0742	0.0094	2.18E-15	62.30720821	-0.0136	0.0164	0.4063
rs17405722	**T2DM**	A	G	0.087	0.0146	2.28E-09	35.507409	-0.033	0.0439	0.4524
rs17411031	**T2DM**	G	C	-0.045	0.0081	3.04E-08	30.86321603	8.00E-04	0.0199	0.966
rs1758632	**T2DM**	G	C	0.0491	0.0081	1.36E-09	36.74338264	-0.0293	0.0175	0.0951393
rs17631783	**T2DM**	T	C	-0.0487	0.0089	3.95E-08	29.94084811	0.0146	0.0574	0.7989
rs17791513	**T2DM**	G	A	-0.1027	0.0148	4.61E-12	48.15081533	-0.0596	0.0329	0.0703493

rs1801214	**T2DM**	T	C	0.0903	0.0074	5.52E-34	148.901218	-0.0397	0.0394	0.3143
rs1899951	**T2DM**	T	C	-0.1118	0.0109	1.64E-24	105.2002569	0.005	0.0388	0.8981
rs2058913	**T2DM**	T	A	-0.0491	0.0078	3.26E-10	39.6241508	-0.033	0.0274	0.2287
rs2237892	**T2DM**	T	C	-0.096	0.0157	8.75E-10	37.38775174	0.0099	0.017	0.5606
rs2246618	**T2DM**	T	C	0.0513	0.0084	1.20E-09	37.29600781	0.0589	0.0208	0.004741
rs2261181	**T2DM**	T	C	0.0985	0.0118	9.18E-17	69.67783297	-0.0019	0.0238	0.9353
rs2294120	**T2DM**	G	A	-0.0443	0.0079	1.62E-08	31.444121	-0.0284	0.0178	0.1107
rs2296173	**T2DM**	G	A	0.065	0.0087	7.66E-14	55.81801615	0.0104	0.0211	0.622499
rs2299383	**T2DM**	T	C	0.0412	0.0073	1.49E-08	31.85186753	-0.0221	0.0162	0.172
rs243019	**T2DM**	C	T	0.0566	0.0071	2.29E-15	63.54806834	-0.0119	0.017	0.4846
rs2493394	**T2DM**	G	A	0.073	0.0113	1.15E-10	41.7325596	0.1363	0.0477	0.00426904
rs2796441	**T2DM**	A	G	-0.0715	0.0073	1.96E-22	95.92958205	-0.0168	0.0162	0.2992
rs2820426	**T2DM**	G	A	0.0521	0.0073	1.30E-12	50.93495366	0.012	0.0158	0.4487
rs2867125	**T2DM**	C	T	0.0601	0.0096	4.33E-10	39.19157049	0.0123	0.0258	0.634501
rs2908282	**T2DM**	A	G	0.0552	0.0094	4.25E-09	34.48328545	-0.018	0.02	0.3698
rs2925979	**T2DM**	C	T	-0.0534	0.0078	9.06E-12	46.868332	-0.0106	0.0167	0.5266
rs2943656	**T2DM**	G	A	0.0902	0.0074	6.70E-34	148.5716083	-0.0232	0.025	0.3526
rs3217992	**T2DM**	T	C	0.0527	0.0073	7.23E-13	52.11487485	-0.0069	0.0158	0.6623
rs340874	**T2DM**	C	T	0.0626	0.0073	8.41E-18	73.5341599	0.0073	0.0162	0.653501
rs348330	**T2DM**	A	G	-0.0487	0.0081	1.86E-09	36.14715103	0.0015	0.0182	0.9329
rs3756784	**T2DM**	G	T	0.0505	0.0091	2.59E-08	30.79542206	0.0024	0.0167	0.8871
rs3802177	**T2DM**	A	G	-0.1217	0.008	2.32E-52	231.412797	-0.0169	0.0159	0.2891
rs459193	**T2DM**	G	A	0.0711	0.0083	8.81E-18	73.37856353	-0.0128	0.0159	0.421
rs4622883	**T2DM**	G	A	-0.0435	0.0078	3.02E-08	31.10108194	0.009	0.0182	0.6208
rs4686471	**T2DM**	C	T	0.0534	0.0081	4.28E-11	43.46089497	0.0424	0.0484	0.3805
rs4812829	**T2DM**	A	G	0.0532	0.0095	2.44E-08	31.35900273	0.0328	0.0163	0.0439704
rs4823182	**T2DM**	G	A	0.0482	0.0077	3.36E-10	39.18310204	-0.0126	0.0158	0.4251
rs4865796	**T2DM**	A	G	0.053	0.0078	1.33E-11	46.16881447	0.0128	0.0208	0.537
rs516946	**T2DM**	C	T	0.0824	0.0085	3.16E-22	93.97292847	0.0687	0.0222	0.00193402
rs5215	**T2DM**	T	C	-0.0678	0.0073	2.09E-20	86.25809379	-0.013	0.0163	0.4246
rs55966194	**T2DM**	G	C	-0.0526	0.0088	2.25E-09	35.72665309	-0.0089	0.056	0.874
rs576674	**T2DM**	A	G	-0.0654	0.0097	1.79E-11	45.45673274	0.0104	0.0233	0.656299
rs6059662	**T2DM**	G	A	0.0446	0.0079	1.51E-08	31.87144278	-0.0066	0.0193	0.7331
rs61953351	**T2DM**	T	G	-0.07	0.0091	1.98E-14	59.16971594	0.0729	0.0513	0.1558
rs622217	**T2DM**	C	T	-0.0485	0.0077	3.13E-10	39.67237641	0.0213	0.0174	0.221
rs6494307	**T2DM**	G	C	-0.0443	0.0078	1.67E-08	32.25554885	0.0265	0.0159	0.0965295
rs6515236	**T2DM**	C	A	-0.0504	0.0091	3.34E-08	30.67358075	0.0021	0.0169	0.9005
rs67232546	**T2DM**	T	C	0.0596	0.0096	4.66E-10	38.54217708	0.0112	0.0231	0.628801
rs6767484	**T2DM**	G	A	0.1209	0.0076	2.70E-56	253.0530675	-0.0233	0.017	0.17
rs6785040	**T2DM**	C	T	-0.0633	0.0111	1.26E-08	32.51978394	0.0103	0.0168	0.5412
rs6795735	**T2DM**	T	C	-0.0558	0.0073	1.63E-14	58.42636488	-0.0318	0.0206	0.1223
rs6878122	**T2DM**	A	G	-0.0564	0.0079	1.19E-12	50.96713417	0.0551	0.0419	0.1877
rs6960043	**T2DM**	C	T	0.064	0.0071	3.61E-19	81.25113559	-0.0179	0.0158	0.257
rs7144011	**T2DM**	T	G	0.0482	0.0085	1.64E-08	32.15454837	-0.0385	0.0565	0.495

rs7177055	**T2DM**	A	G	0.0647	0.0079	2.75E-16	67.07189361	-0.0086	0.0163	0.6
rs7240767	**T2DM**	C	T	0.0451	0.0081	2.16E-08	31.00053829	0.0265	0.0172	0.1229
rs72802358	**T2DM**	C	G	-0.1168	0.0133	1.97E-18	77.1202791	-0.0411	0.0518	0.4283
rs72892910	**T2DM**	T	G	0.0648	0.0099	6.43E-11	42.84161278	0.0029	0.0194	0.881
rs735949	**T2DM**	C	T	-0.0711	0.0106	1.95E-11	44.98975829	-0.0419	0.0777	0.589899
rs753270	**T2DM**	C	T	0.0528	0.0079	2.70E-11	44.66834394	0.0175	0.0194	0.3685
rs7561798	**T2DM**	G	A	0.04	0.0072	2.79E-08	30.86321603	-0.0042	0.0172	0.809
rs7572970	**T2DM**	G	A	0.059	0.0087	1.39E-11	45.98876076	0.0108	0.0213	0.611499
rs7607777	**T2DM**	T	G	-0.137	0.0125	9.40E-28	120.1177801	0.0243	0.0983	0.8049
rs7674212	**T2DM**	T	G	-0.0465	0.0075	6.18E-10	38.43877759	0.0222	0.0166	0.1817
rs7685296	**T2DM**	T	C	-0.0511	0.0081	2.32E-10	39.79769794	-0.008	0.016	0.6167
rs7729395	**T2DM**	T	C	0.1373	0.016	1.10E-17	73.63550984	-0.0762	0.105	0.4677
rs7756992	**T2DM**	G	A	0.1297	0.0078	6.00E-62	276.488413	0.0082	0.0159	0.6036
rs7786095	**T2DM**	G	A	-0.0743	0.0129	9.64E-09	33.17297305	0.1072	0.0963	0.2655
rs780094	**T2DM**	C	T	0.0692	0.0074	5.16E-21	87.44499121	0.0137	0.0159	0.3902
rs7845219	**T2DM**	C	T	-0.0422	0.0072	4.54E-09	34.35153102	-0.0128	0.0172	0.4571
rs7903146	**T2DM**	T	C	0.3059	0.0077	1.00E-200	1578.206009	-0.0051	0.0333	0.8782
rs7929543	**T2DM**	C	A	0.0828	0.0138	2.20E-09	35.99885518	-0.0133	0.0213	0.5332
rs7955901	**T2DM**	T	C	-0.0444	0.0072	7.16E-10	38.02656847	0.0203	0.0166	0.2212
rs8068804	**T2DM**	A	G	0.0587	0.0078	4.41E-14	56.63347181	-0.0369	0.0176	0.0363203
rs8108269	**T2DM**	G	T	0.0644	0.0079	3.11E-16	66.45133972	-0.0117	0.0165	0.4788
rs825476	**T2DM**	T	C	0.0524	0.0073	6.80E-13	51.52322543	-0.0171	0.0184	0.355
rs840967	**T2DM**	A	C	-0.0497	0.008	5.44E-10	38.5939289	-0.0299	0.0159	0.0605899
rs849135	**T2DM**	A	G	-0.0999	0.0072	1.04E-43	192.5095029	-0.0577	0.047	0.2188
rs853974	**T2DM**	C	T	-0.0601	0.0088	7.86E-12	46.64120785	0.0106	0.0163	0.5145
rs9369425	**T2DM**	A	G	-0.0546	0.0085	1.13E-10	41.26041796	-0.0272	0.0242	0.2604
rs963740	**T2DM**	T	A	-0.0479	0.0086	2.23E-08	31.02132283	-0.0098	0.0162	0.5478
rs9844972	**T2DM**	C	G	0.0956	0.0148	1.03E-10	41.72328964	0.155	0.1036	0.1347
rs9894220	**T2DM**	G	A	-0.0585	0.0079	1.52E-13	54.83321856	0.0089	0.0179	0.618399
rs9928094	**T2DM**	G	A	0.1045	0.0072	3.59E-47	210.6462718	-0.0132	0.0184	0.4731
rs993380	**T2DM**	G	A	-0.0507	0.0081	4.59E-10	39.17708058	2.00E-04	0.0163	0.9919
rs9940149	**T2DM**	A	G	-0.058	0.0095	9.29E-10	37.27305289	-0.0145	0.0173	0.3994

T2DM: type 2 diabetes mellitus. EA: effect allele. OA: other allele. GX: beta-exposure. GX(SE): standard error of GX. GY: beta-outcome. GY(SE): standard error of GY.

Table S3. Published associations of Hyperthyroidism on PTB

SNP	Exposure	EA	OA	GX	GX(SE)	Pval-exp	F	GY	GY(SE)	Pval-outcome
rs1794280	**HT**	T	A	0.5463	0.0391	2.29E-44	195.2122198	-0.1543	0.0792	0.0515502
rs2160215	**HT**	C	T	0.2483	0.0226	3.82E-28	120.7076166	0.0137	0.0163	0.4023
rs28375776	**HT**	G	C	-0.2432	0.0407	2.25E-09	35.70560832	-0.1035	0.0241	1.77E-05
rs3087243	**HT**	A	G	-0.2038	0.0224	7.94E-20	82.77714368	0.0062	0.0176	0.724499
rs385863	**HT**	G	C	0.1335	0.0214	4.09E-10	38.91643942	0.0096	0.0158	0.542
rs4338740	**HT**	C	T	0.1844	0.0264	2.87E-12	48.78789646	-0.0429	0.0354	0.2255
rs58722186	**HT**	T	C	0.1359	0.0233	5.23E-09	34.01928528	0.008	0.0176	0.6505
rs604912	**HT**	G	A	0.1198	0.0217	3.15E-08	30.47840826	-0.0038	0.0161	0.8141
rs6131010	**HT**	G	A	0.1306	0.0237	3.60E-08	30.36601314	-0.0266	0.0168	0.1133
rs6679677	**HT**	A	C	0.2936	0.0379	8.79E-15	60.01112887	0.1106	0.0695	0.1114
rs9258222	**HT**	A	G	-0.2367	0.0418	1.44E-08	32.06579901	-0.0245	0.0208	0.2386

HT: Hyperthyroidism. EA: effect allele. OA: other allele. GX: beta-exposure. GX(SE): standard error of GX. GY: beta-outcome. GY(SE): standard error of GY.

Table S4. Published associations of Obesity on PTB

SNP	Exposure	EA	OA	GX	GX(SE)	Pval-exp	GY	GY(SE)	Pval-outcome
rs1040070	**Obesity**	C	G	-0.1492	0.0269	2.77E-08	-0.0418	0.0283	0.1399
rs4854344	**Obesity**	T	G	0.2445	0.0351	3.22E-12	0.0142	0.0257	0.5798
rs571312	**Obesity**	A	C	0.1986	0.0309	1.25E-10	-0.0367	0.0192	0.0559706
rs6752378	**Obesity**	A	C	0.1695	0.0262	1.05E-10	-0.0365	0.0159	0.0216202
rs7138803	**Obesity**	A	G	0.1672	0.0271	6.50E-10	0.0341	0.0165	0.0384601
rs9941349	**Obesity**	T	C	0.1978	0.0267	1.16E-13	-0.011	0.0184	0.5512

EA: effect allele. OA: other allele. GX: beta-exposure. GX(SE): standard error of GX. GY: beta-outcome. GY(SE): standard error of GY.

Table S5. Published associations of HC on PTB

SNP	Exposure	EA	OA	GX	GX(SE)	Pval-exp	F	GY	GY(SE)	Pval-outcome
rs10085881	**HC**	C	T	0.00641021	0.000727168	3.90E-19	77.70933714	0.0072	0.0226	0.751201
rs10096633	**HC**	T	C	-0.00894032	0.000968179	7.50E-20	85.2693629	0.0012	0.024	0.9611
rs10410835	**HC**	C	T	0.00704212	0.000657094	2.90E-27	114.8550373	-0.0397	0.112	0.7229
rs1042725	**HC**	T	C	0.00365641	0.000647915	2.00E-08	31.84724836	0.0372	0.0175	0.0332997
rs10504255	**HC**	A	G	-0.00528436	0.000688655	1.10E-14	58.88159836	-0.0209	0.0207	0.3109
rs1065853	**HC**	T	G	-0.0403752	0.0011899	1.00E-200	1151.348109	-0.0403	0.205	0.844
rs1077835	**HC**	G	A	0.00507236	0.000775598	1.10E-10	42.77056475	0.0244	0.0161	0.1292

rs1081105	**HC**	C	A	0.0283942	0.00195441	1.50E-47	211.069815	-0.1691	0.1655	0.307
rs111278137	**HC**	A	G	-0.0134643	0.00233405	8.40E-09	33.27709882	-0.1261	0.2218	0.5696
rs112403212	**HC**	T	C	0.00571274	0.000934285	1.30E-09	37.38766757	-0.1432	0.1612	0.3743
rs11591147	**HC**	T	G	-0.0475535	0.00251519	5.10E-81	357.455173	0.0891	0.1332	0.5037
rs11601507	**HC**	A	C	0.00767804	0.00124753	5.20E-10	37.87886453	-0.0302	0.0344	0.3791
rs11621792	**HC**	T	C	0.00379086	0.000658759	6.80E-09	33.11468102	-0.0227	0.0299	0.4482
rs1169288	**HC**	C	A	0.00629553	0.000701132	7.10E-19	80.62379287	-8.00E-04	0.0158	0.9593
rs117733303	**HC**	G	A	0.0312162	0.00239775	6.50E-40	169.4924968	-0.0628	0.0254	0.0135301
rs11858759	**HC**	A	G	0.00588404	0.000709326	2.80E-16	68.8109704	0.0132	0.019	0.4896
rs11889765	**HC**	C	G	0.00659743	0.000972594	6.60E-12	46.01343097	0.0398	0.0314	0.2044
rs12151108	**HC**	A	G	-0.0265351	0.00100157	3.40E-156	701.9029208	-0.0266	0.0775	0.7319
rs1216743	**HC**	A	G	0.00413639	0.000726161	1.20E-08	32.44705804	0.0198	0.0161	0.2181
rs12509595	**HC**	C	T	0.0040815	0.000715494	2.60E-09	32.54061952	-0.0155	0.0172	0.3688
rs1260326	**HC**	C	T	-0.0106258	0.000664199	3.30E-57	255.9322693	0.0031	0.0159	0.8472
rs12740374	**HC**	T	G	-0.0187542	0.000776044	1.40E-130	584.0131188	0.0388	0.028	0.1661
rs12916	**HC**	C	T	0.0109365	0.000659712	6.70E-62	274.81862	5.00E-04	0.0158	0.9757
rs13173241	**HC**	A	G	0.00453483	0.000797233	1.00E-08	32.35561769	-0.0037	0.0261	0.8871
rs13379043	**HC**	C	T	-0.00444335	0.000734733	1.50E-09	36.57296433	0.0053	0.0175	0.763001
rs1367117	**HC**	A	G	0.0151916	0.000690641	4.20E-108	483.8386165	-0.0244	0.0269	0.3635
rs141783576	**HC**	C	G	0.00717336	0.0012947	4.30E-08	30.69765247	-0.0019	0.0889	0.9828
rs144018203	**HC**	C	G	0.0193282	0.00336484	8.00E-09	32.99530849	-0.7	0.3494	0.0451398
rs147233090	**HC**	T	C	0.0124289	0.00212946	8.00E-09	34.06627296	0.2777	0.2631	0.2911
rs1501908	**HC**	C	G	0.00702065	0.000670644	9.50E-26	109.5894547	-0.0191	0.0202	0.3452
rs174566	**HC**	G	A	-0.00396442	0.000681221	1.60E-09	33.86736977	0.0457	0.0162	0.00482003
rs17725246	**HC**	C	T	0.00661106	0.000830138	1.20E-15	63.42198291	0.0152	0.0162	0.3503
rs17819328	**HC**	G	T	0.0044539	0.000659494	1.80E-11	45.60972293	0.002	0.0224	0.9286
rs1883025	**HC**	T	C	-0.0064241	0.000737527	8.90E-18	75.86945164	-0.0181	0.0177	0.3052
rs1883711	**HC**	C	G	0.0240925	0.00192412	1.00E-36	156.782518	-0.0357	0.1058	0.7357
rs193084249	**HC**	G	A	0.0150976	0.00221622	1.10E-11	46.40751329	0.0349	0.1287	0.786401
rs2068888	**HC**	A	G	-0.00507614	0.000650225	3.30E-15	60.94499753	-0.0214	0.0162	0.1868
rs2169387	**HC**	G	A	0.00645434	0.00107309	1.30E-09	36.1767535	-0.0305	0.0532	0.566301
rs2519093	**HC**	T	C	0.0101238	0.000838042	1.50E-33	145.9330812	-0.0166	0.0178	0.3532
rs2618567	**HC**	T	G	-0.00497182	0.000680459	1.40E-13	53.38571658	0.0099	0.0175	0.5716
rs2738447	**HC**	C	A	0.00834437	0.000659035	1.40E-36	160.3128571	0.0078	0.0162	0.629901
rs28601761	**HC**	G	C	-0.0139751	0.00066466	1.20E-99	442.0882473	0.0162	0.0203	0.4252
rs28615248	**HC**	C	T	0.00560562	0.000816472	2.70E-12	47.13710696	0.0103	0.0196	0.600101
rs28807203	**HC**	C	A	-0.0102164	0.00144984	3.20E-12	49.65395308	0.0201	0.0318	0.5284
rs2972147	**HC**	C	T	0.00511702	0.000676784	3.70E-14	57.16527153	-0.0219	0.0256	0.3939
rs34042070	**HC**	G	C	0.00827464	0.00082818	3.30E-24	99.82675347	-0.0151	0.0166	0.3617
rs34631598	**HC**	A	G	0.00722975	0.00120156	1.10E-09	36.20377382	0.0594	0.0717	0.4078
rs34707604	**HC**	C	T	0.00581081	0.000787391	1.70E-13	54.46163375	-0.0061	0.0558	0.9125
rs35203651	**HC**	C	T	0.00567145	0.00102558	2.00E-08	30.58069399	-0.0099	0.0245	0.686501
rs360801	**HC**	G	A	-0.00387215	0.000679576	9.50E-09	32.46582829	-0.0128	0.0185	0.4901
rs3874910	**HC**	T	C	-0.00447854	0.000655522	2.30E-12	46.67631685	0.012	0.0161	0.4553

rs3918226	**HC**	T	C	0.00750006	0.0012245	5.10E-10	37.51544516	-0.0197	0.0922	0.8305
rs4299376	**HC**	T	G	-0.0112908	0.000695213	3.00E-60	263.7617817	-0.0335	0.0564	0.5521
rs456598	**HC**	A	G	0.00687697	0.000938596	1.10E-13	53.68278879	-0.0238	0.0728	0.744
rs472495	**HC**	T	G	0.00590634	0.000677384	1.20E-18	76.02654005	-0.029	0.0183	0.1138
rs4921914	**HC**	T	C	-0.00608414	0.000774849	3.40E-15	61.65416944	0.0054	0.0171	0.753701
rs556107	**HC**	T	C	0.00506757	0.000652202	1.10E-14	60.37170872	-0.011	0.019	0.5624
rs58542926	**HC**	T	C	-0.0139615	0.00123112	4.60E-30	128.6061081	0.0338	0.0298	0.2561
rs59784135	**HC**	G	A	-0.00440324	0.000727114	8.10E-10	36.67227976	0.009	0.0176	0.608199
rs6090040	**HC**	C	A	-0.00539677	0.000649902	1.70E-16	68.95571722	0.0234	0.017	0.1677
rs6093446	**HC**	A	G	0.00501433	0.000711138	8.30E-13	49.71829877	-0.0301	0.0167	0.0723902
rs62275880	**HC**	T	A	0.0058993	0.00073623	1.00E-15	64.20548805	0.2172	0.1262	0.0853002
rs622871	**HC**	G	A	0.00551676	0.000711024	2.20E-15	60.20019098	-0.0818	0.0271	0.002537
rs679582	**HC**	A	G	-0.00483667	0.000667916	6.70E-13	52.43815795	-0.0118	0.0179	0.5113
rs687339	**HC**	T	C	0.00472978	0.000768228	5.90E-10	37.90529008	0.0046	0.0206	0.823
rs6905288	**HC**	A	G	0.00529521	0.000654198	2.20E-16	65.51581682	-0.0039	0.0189	0.8372
rs7140110	**HC**	C	T	0.00434657	0.000706659	1.30E-09	37.83308664	0.0271	0.0196	0.1664
rs72631343	**HC**	G	C	-0.00585804	0.000958814	2.90E-10	37.32795001	-0.0018	0.0167	0.9126
rs72805692	**HC**	G	A	-0.00619544	0.0010311	1.50E-09	36.10280492	-0.0665	0.0934	0.4766
rs72837687	**HC**	A	G	-0.00487029	0.000816337	2.10E-09	35.59335198	8.00E-04	0.0194	0.9654
rs73352129	**HC**	G	C	-0.00480407	0.000714174	8.50E-12	45.24897967	0.0015	0.016	0.9252
rs74617384	**HC**	T	A	0.0198031	0.00122267	3.60E-59	262.3288504	0.0853	0.1087	0.4327
rs7534572	**HC**	G	C	0.00822692	0.000675295	2.20E-34	148.4176968	0.1115	0.1155	0.3347
rs7581601	**HC**	C	A	-0.00394211	0.000706276	2.70E-08	31.15349584	-0.0936	0.0376	0.0128899
rs76895963	**HC**	G	T	-0.0165229	0.00254087	2.10E-11	42.28690162	0.2973	0.152	0.0505196
rs7746081	**HC**	A	G	-0.00537028	0.00070221	2.20E-14	58.48682522	0.0418	0.0197	0.0335699
rs77542162	**HC**	G	A	0.0232886	0.0022252	1.70E-26	109.5334756	-0.3167	0.2464	0.1986
rs78058190	**HC**	A	G	0.0109303	0.0016854	9.80E-11	42.05875304	0.1158	0.0981	0.2378
rs7903146	**HC**	T	C	0.00543539	0.000711672	6.80E-15	58.33106034	-0.0051	0.0333	0.8782
rs799157	**HC**	C	T	-0.0114878	0.00159308	2.60E-13	51.99921277	-0.2819	0.1454	0.0524904
rs8090363	**HC**	G	A	0.00432555	0.00066427	8.50E-11	42.40251362	-0.0084	0.0184	0.647
rs8103315	**HC**	A	C	0.00561305	0.000990293	1.40E-08	32.12688442	0.0257	0.0633	0.684699
rs907348	**HC**	C	T	-0.00368655	0.000673327	3.70E-08	29.97687833	0.0062	0.0167	0.711001
rs9442198	**HC**	G	A	-0.00427947	0.000717698	1.00E-09	35.55451085	0.0284	0.061	0.6414
rs9534342	**HC**	T	C	-0.00357418	0.000646473	3.10E-08	30.56682044	0.0094	0.0164	0.564999
rs964184	**HC**	C	G	-0.0207869	0.000937969	1.40E-111	491.134781	0.0085	0.0183	0.640801
rs972283	**HC**	G	A	0.00382459	0.00064968	1.20E-08	34.65524576	-0.0337	0.0175	0.0535599

HC:High cholesterol. EA: effect allele. OA: other allele. GX: beta-exposure. GX(SE): standard error of GX. GY: beta-outcome. GY(SE): standard error of GY.

Table S6. Published associations of Fasting blood glucose on PTB

SNP	Exposure	EA	OA	GX	GX(SE)	Pval-exp	F	GY	GY(SE)	Pval-outcome
rs10830963	**FBG**	G	C	0.133751	0.0114092	9.71E-32	137.410402	0.0068	0.016	0.672
rs1260326	**FBG**	C	T	0.0778412	0.00983496	2.48E-15	62.6339455	0.0031	0.0159	0.8472
rs1799884	**FBG**	T	C	0.117126	0.0112502	2.21E-25	108.3732402	-0.0119	0.0209	0.5695
rs1879442	**FBG**	A	G	-0.0534129	0.00952255	2.03E-08	31.45731956	-0.0097	0.0191	0.6123
rs2389615	**FBG**	C	T	0.0844091	0.0101118	6.97E-17	69.67187818	-0.1992	0.1678	0.235
rs2908277	**FBG**	A	G	0.069616	0.0115153	1.49E-09	36.54294459	-0.0378	0.0221	0.08684
rs560887	**FBG**	C	T	0.164581	0.0133583	7.03E-35	151.7724446	0.0179	0.0376	0.632799

FBG: Fasting blood glucose. EA: effect allele. OA: other allele. GX: beta-exposure. GX(SE): standard error of GX. GY: beta-outcome. GY(SE): standard error of GY.

Table S7. Published associations of Two-hour glucose on PTB

SNP	Exposure	EA	OA	GX	GX(SE)	Pval-exp	F	GY	GY(SE)	Pval-outcome
rs11708067	**OGTT**	G	A	-0.0872	0.0093	1.98E-22	87.91305487	-0.1924	0.0619	0.00187802
rs117643180	**OGTT**	A	C	0.234	0.0327	7.31E-14	51.2061953	0.0607	0.072	0.3997
rs1260326	**OGTT**	C	T	-0.0486	0.0078	5.93E-12	38.82126045	0.0031	0.0159	0.8472
rs12692738	**OGTT**	C	T	-0.0486	0.009	2.72E-08	29.15908007	-0.0581	0.0294	0.0483905
rs17271305	**OGTT**	G	A	0.0587	0.0077	2.88E-14	58.11403772	-0.0174	0.022	0.4289
rs1800437	**OGTT**	C	G	0.1004	0.0099	4.79E-26	102.8450362	-0.0402	0.0188	0.0321203
rs2649999	**OGTT**	C	T	-0.0498	0.0082	2.01E-10	36.88223915	0.0223	0.0162	0.1681
rs4148646	**OGTT**	G	C	-0.0397	0.0078	4.39E-08	25.90467255	-0.0085	0.0162	0.6008
rs4898944	**OGTT**	G	C	-0.0579	0.01	1.38E-08	33.52304239	-0.0064	0.016	0.688101
rs550057	**OGTT**	T	C	0.0526	0.0085	3.61E-11	38.29304796	-0.0105	0.0176	0.5495
rs76453951	**OGTT**	T	C	-0.0698	0.0117	4.03E-10	35.5897896	-0.0821	0.0657	0.2112
rs7903146	**OGTT**	T	C	0.0854	0.0087	2.79E-26	96.35262145	-0.0051	0.0333	0.8782
rs878521	**OGTT**	A	G	0.099	0.0094	1.25E-28	110.917732	-0.0178	0.0166	0.2844
rs9808924	**OGTT**	A	G	0.0559	0.0082	1.56E-11	46.47102051	-0.0226	0.0167	0.1755

OGTT: Oral Two-hour glucose. EA: effect allele. OA: other allele. GX: beta-exposure. GX(SE): standard error of GX. GY: beta-outcome. GY(SE): standard error of GY.

Table S8. Published associations of HbA1c on PTB

SNP	Exposure	EA	OA	GX	GX(SE)	Pval-exp	GY	GY(SE)	Pval-outcome
rs10420309	**HbA1c**	G	A	-0.0392	0.0062	2.87E-10	-0.0027	0.0184	0.8819
rs1046917	**HbA1c**	G	A	-0.0757	0.007	1.47E-27	-0.0417	0.0161	0.00943691
rs10830963	**HbA1c**	G	C	0.0518	0.0072	5.24E-13	0.0068	0.016	0.672
rs10965248	**HbA1c**	C	T	-0.0537	0.0084	1.84E-10	-0.0267	0.0163	0.103
rs1106676	**HbA1c**	G	T	0.052	0.0067	6.19E-15	-1.00E-04	0.0171	0.9962

rs11187141	**HbA1c**	T	A	-0.0402	0.0066	1.02E-09	-0.0286	0.019	0.1315
rs11708067	**HbA1c**	G	A	-0.0584	0.0073	9.53E-16	-0.1924	0.0619	0.00187802
rs13089972	**HbA1c**	A	T	0.0362	0.0064	1.91E-08	0.0083	0.016	0.6016
rs145731180	**HbA1c**	T	G	-0.0455	0.0083	3.88E-08	0.0385	0.024	0.1082
rs17476364	**HbA1c**	C	T	-0.3044	0.0101	3.02E-199	-0.1129	0.0968	0.2433
rs17533945	**HbA1c**	C	T	0.045	0.0065	6.02E-12	0.0233	0.0162	0.1507
rs17712208	**HbA1c**	A	T	0.1112	0.0184	1.39E-09	-0.359	0.1702	0.0349301
rs1879442	**HbA1c**	A	G	-0.0479	0.0071	1.43E-11	-0.0097	0.0191	0.6123
rs2732469	**HbA1c**	A	T	-0.0563	0.0064	9.47E-19	-0.1026	0.042	0.0145201
rs2748427	**HbA1c**	G	A	0.0849	0.0076	9.16E-29	0.0322	0.02	0.1073
rs2908289	**HbA1c**	A	G	0.1141	0.0083	6.72E-43	-0.0125	0.0209	0.5508
rs2968478	**HbA1c**	G	T	0.058	0.0064	2.19E-19	-0.0046	0.0167	0.7817
rs35280834	**HbA1c**	A	C	0.055	0.0089	6.04E-10	-0.0122	0.0295	0.679499
rs3818717	**HbA1c**	C	T	-0.0472	0.0064	1.84E-13	1.00E-04	0.026	0.9977
rs3935875	**HbA1c**	G	A	0.0548	0.0073	4.31E-14	0.035	0.0228	0.1259
rs4300038	**HbA1c**	A	G	-0.0549	0.0068	8.39E-16	-0.0217	0.0164	0.1858
rs4340756	**HbA1c**	G	A	-0.0644	0.0066	3.17E-22	0.0659	0.0215	0.002169
rs4737010	**HbA1c**	A	G	0.0735	0.0076	3.59E-22	0.0127	0.017	0.4565
rs560887	**HbA1c**	C	T	0.1156	0.0068	1.48E-64	0.0179	0.0376	0.632799
rs6602909	**HbA1c**	C	T	0.0492	0.0066	1.21E-13	0.014	0.0172	0.4162
rs66593272	**HbA1c**	T	A	-0.1296	0.0178	3.85E-13	-0.0292	0.0379	0.4409
rs75431224	**HbA1c**	T	A	-0.0472	0.0078	1.47E-09	0.0037	0.0438	0.9322
rs76323047	**HbA1c**	G	A	0.0574	0.0098	4.41E-09	-0.0386	0.0233	0.0974698
rs7903146	**HbA1c**	T	C	0.0655	0.0074	5.80E-19	-0.0051	0.0333	0.8782
rs79220007	**HbA1c**	C	T	-0.1353	0.0112	1.91E-33	0.0443	0.1152	0.7008
rs855791	**HbA1c**	G	A	-0.0549	0.0064	9.41E-18	2.00E-04	0.0159	0.9918
rs857721	**HbA1c**	A	T	0.0698	0.007	2.65E-23	-0.0039	0.0163	0.8096
rs9376090	**HbA1c**	C	T	-0.0554	0.0072	1.06E-14	0.0138	0.0166	0.4067
rs9410357	**HbA1c**	G	A	-0.1112	0.0131	2.45E-17	0.2082	0.1237	0.0924592
rs9826367	**HbA1c**	G	A	-0.0402	0.0063	2.26E-10	0.0589	0.0361	0.1029
rs9937664	**HbA1c**	C	T	0.0413	0.0073	1.43E-08	-0.0146	0.0212	0.4919

HbA1c: hemoglobin Alc. EA: effect allele. OA: other allele. GX: beta-exposure. GX(SE): standard error of GX. GY: beta-outcome. GY(SE): standard error of GY.

Table S9. Published associations of BGL on PTB

SNP	Exposure	EA	OA	GX	GX(SE)	Pval-exp	F	GY	GY(SE)	Pval-outcome
rs10244051	**BGL**	G	T	0.0399066	0.00218292	1.10E-76	334.2040027	-0.0156	0.0169	0.357
rs10248619	**BGL**	C	T	-0.0216976	0.00259776	9.90E-18	69.76268346	-5.00E-04	0.0211	0.9825
rs10276674	**BGL**	C	T	0.0319109	0.00282892	2.20E-31	127.2432066	-0.0199	0.0163	0.2206
rs10492373	**BGL**	A	G	-0.020594	0.00273255	3.50E-14	56.79930387	-0.013	0.0182	0.4759
rs10501320	**BGL**	C	G	-0.0322165	0.00246962	3.00E-40	170.1744194	0.0109	0.0586	0.8518
rs1055080	**BGL**	A	G	-0.0612651	0.00589121	2.50E-27	108.1471593	0.0048	0.0204	0.8137

rs10758593	**BGL**	A	G	0.0195316	0.00221825	2.60E-19	77.5269134	0.0115	0.0161	0.4763
rs10807124	**BGL**	A	G	-0.0144984	0.00248915	1.60E-09	33.92624926	0.0096	0.0186	0.6064
rs10811661	**BGL**	C	T	-0.0351524	0.00287402	1.70E-34	149.5988386	-0.0261	0.0163	0.1094
rs10849893	**BGL**	G	C	-0.0130203	0.00221043	6.90E-09	34.69654895	-0.0365	0.0196	0.0633301
rs11257655	**BGL**	T	C	0.0273482	0.00267831	1.80E-24	104.2638218	-0.021	0.0165	0.2036
rs11418239	**BGL**	GA	G	0.0215509	0.00311241	2.40E-12	47.94408674	-0.1175	0.0703	0.0947196
rs11603334	**BGL**	A	G	-0.0257224	0.00302919	1.30E-16	72.10539779	0.0036	0.0347	0.9168
rs1169302	**BGL**	G	T	0.0126694	0.00221096	2.10E-08	32.8358419	-0.0091	0.0167	0.5856
rs11708067	**BGL**	G	A	-0.0553971	0.00253223	2.40E-108	478.5921735	-0.1924	0.0619	0.00187802
rs117710037	**BGL**	T	G	0.0366422	0.00620825	1.30E-09	34.83553769	0.1608	0.1404	0.2524
rs118115876	**BGL**	A	G	0.0497695	0.00707519	1.40E-12	49.48210566	-0.0267	0.2371	0.9104
rs1220123	**BGL**	G	A	-0.0193972	0.00320378	1.20E-09	36.65646209	0.0161	0.0681	0.8129
rs12452315	**BGL**	C	A	0.0181979	0.002202	3.30E-17	68.29764835	-0.0091	0.0172	0.596499
rs1260326	**BGL**	C	T	0.0371717	0.00223727	1.90E-60	276.0488102	0.0031	0.0159	0.8472
rs12769814	**BGL**	T	G	-0.0507895	0.00381994	1.50E-41	176.7797938	0.0186	0.0316	0.5563
rs13182927	**BGL**	G	A	0.015558	0.00293292	1.60E-08	28.13875843	-0.0084	0.0162	0.606301
rs13320382	**BGL**	A	G	-0.0194615	0.00345619	6.60E-09	31.70700591	0.1052	0.0825	0.2022
rs1359790	**BGL**	A	G	-0.017964	0.00240623	1.10E-13	55.73521099	0.0095	0.0177	0.5926
rs1627787	**BGL**	T	C	-0.0231418	0.00263095	1.70E-17	77.36898754	0.0126	0.0195	0.518
rs163177	**BGL**	C	T	0.0145791	0.00217575	8.20E-12	44.89947639	-0.0194	0.0161	0.229
rs16989483	**BGL**	C	T	-0.0210895	0.00243313	2.40E-18	75.1276442	0.0272	0.0231	0.2378
rs16989483	**BGL**	C	T	-0.0210895	0.00243313	2.40E-18	75.1276442	-0.1575	0.2388	0.5095
rs17036326	**BGL**	G	A	-0.0202428	0.0033305	3.30E-09	36.94197615	0.0066	0.0388	0.8659
rs17350833	**BGL**	A	G	-0.0142578	0.00240521	5.60E-10	35.13960426	-0.0045	0.0177	0.7995
rs174548	**BGL**	G	C	-0.0224863	0.00234211	3.50E-22	92.17629658	0.0432	0.0162	0.00779704
rs17712208	**BGL**	A	T	0.0810992	0.00590138	1.60E-45	188.8529661	-0.359	0.1702	0.0349301
rs1799884	**BGL**	T	C	0.110289	0.00283588	1.00E-200	1512.469165	-0.0119	0.0209	0.5695
rs1801214	**BGL**	T	C	0.0147594	0.00225318	2.90E-10	42.90851273	-0.0397	0.0394	0.3143
rs1882297	**BGL**	A	G	0.0159274	0.00231632	4.50E-13	47.2814178	-0.0053	0.0236	0.8216
rs221798	**BGL**	G	C	0.0258535	0.00342049	4.80E-14	57.12942746	-0.0417	0.0375	0.2658
rs2229357	**BGL**	A	G	-0.0198612	0.00255868	1.10E-15	60.25274697	-0.0576	0.027	0.0329997
rs2236418	**BGL**	G	A	-0.0212306	0.00283559	2.20E-14	56.05772836	0.0135	0.0164	0.4097
rs2302593	**BGL**	G	C	-0.0163302	0.00220081	8.40E-14	55.05740485	-0.0603	0.0481	0.2099
rs231847	**BGL**	G	A	0.0145344	0.00218365	1.20E-11	44.3022708	-0.0122	0.0247	0.6206
rs259134	**BGL**	A	G	0.0158758	0.00217457	3.00E-13	53.29939565	-0.0286	0.0183	0.1173
rs2800734	**BGL**	A	G	-0.0198928	0.00239707	7.60E-17	68.86970621	0.0037	0.0232	0.872
rs28361101	**BGL**	C	G	-0.0567038	0.00699397	9.80E-18	65.73166435	0.0624	0.1612	0.6988
rs28364870	**BGL**	T	C	0.0230524	0.00286401	4.50E-16	64.78598475	-9.00E-04	0.0221	0.9692
rs2842185	**BGL**	T	C	-0.012614	0.00225281	2.60E-08	31.35121337	-0.0122	0.0189	0.5206
rs28641468	**BGL**	C	T	0.0239879	0.00252881	5.30E-21	89.98080826	0.0368	0.0228	0.1066
rs2969962	**BGL**	T	C	-0.0183822	0.00281304	1.40E-10	42.70128945	-0.014	0.0181	0.4372
rs3217795	**BGL**	G	A	-0.025172	0.00385657	1.20E-10	42.60208357	0.0147	0.0816	0.8569
rs3217992	**BGL**	T	C	0.0136734	0.00225631	2.00E-09	36.72428357	-0.0069	0.0158	0.6623
rs34402524	**BGL**	G	T	0.0258025	0.00333057	2.90E-15	60.01838054	-0.0297	0.0389	0.4464

rs35061774	**BGL**	C	G	-0.0157471	0.00231003	1.40E-11	46.46904655	1.00E-04	0.0162	0.9965
rs35097172	**BGL**	T	C	-0.0168265	0.00257468	1.30E-10	42.71091661	-0.01	0.0254	0.6925
rs35742417	**BGL**	A	C	-0.0315027	0.00283335	4.70E-29	123.6211942	-0.0066	0.0459	0.8855
rs35802157	**BGL**	C	T	-0.0284269	0.00226539	1.60E-35	157.4602281	0.0078	0.0238	0.7413
rs3738187	**BGL**	A	G	0.0169247	0.00269532	1.80E-09	39.42930498	9.00E-04	0.023	0.9701
rs3750952	**BGL**	C	G	-0.0178018	0.00219097	1.70E-16	66.01654689	0.0216	0.0185	0.2448
rs3757972	**BGL**	T	C	0.0151913	0.00223889	1.80E-12	46.03860954	-0.0015	0.0175	0.9309
rs3802177	**BGL**	A	G	-0.0494779	0.00235278	1.40E-101	442.2396814	-0.0169	0.0159	0.2891
rs41279627	**BGL**	T	C	-0.0460663	0.00511627	3.00E-19	81.06951659	0.0906	0.1342	0.4998
rs4277405	**BGL**	T	C	-0.0122889	0.00225324	1.90E-08	29.74465574	0.0118	0.0163	0.471
rs4402960	**BGL**	T	G	0.0143666	0.00234112	9.50E-11	37.65810665	-0.023	0.017	0.1757
rs4430796	**BGL**	A	G	-0.0150243	0.00217903	2.70E-11	47.54007973	-0.0114	0.0163	0.4865
rs4581570	**BGL**	C	T	0.0157965	0.00239754	4.50E-11	43.40980665	-0.02	0.0167	0.231
rs464605	**BGL**	T	C	0.0149707	0.00249678	1.20E-09	35.95187064	-0.0135	0.0159	0.396
rs4679370	**BGL**	C	T	0.0132064	0.00217999	1.50E-09	36.69929509	-0.0119	0.0162	0.4605
rs4924455	**BGL**	T	A	-0.0232016	0.00418131	3.20E-08	30.78994884	-0.0297	0.0169	0.0797297
rs494859	**BGL**	C	A	-0.0472277	0.00289821	4.40E-60	265.5410826	0.0017	0.0262	0.9474
rs4984303	**BGL**	C	T	-0.013649	0.00220685	2.00E-09	38.25197545	0.0269	0.0274	0.3269
rs507666	**BGL**	A	G	0.0294062	0.0028087	2.10E-27	109.6137248	-0.0159	0.0178	0.3736
rs5215	**BGL**	T	C	-0.0203383	0.00228432	2.60E-19	79.27080069	-0.013	0.0163	0.4246
rs5398	**BGL**	A	G	-0.0689784	0.00240648	1.30E-179	821.598335	-0.0085	0.019	0.656999
rs560887	**BGL**	C	T	0.152884	0.00238819	1.00E-200	4098.115119	0.0179	0.0376	0.632799
rs56284809	**BGL**	A	G	-0.0347622	0.00315148	4.00E-28	121.6699216	-0.011	0.0188	0.5565
rs576674	**BGL**	A	G	-0.0365977	0.0029119	9.10E-37	157.9619563	0.0104	0.0233	0.656299
rs6004307	**BGL**	A	G	0.0154156	0.00262442	3.10E-09	34.50261556	-0.004	0.0213	0.8515
rs6006399	**BGL**	G	T	-0.0210721	0.00340748	2.20E-10	38.24254949	0.0481	0.0317	0.1289
rs60983647	**BGL**	A	C	0.0280874	0.00462892	2.00E-09	36.81811579	-0.0192	0.0469	0.6824
rs61856594	**BGL**	G	A	-0.0166964	0.00238217	4.20E-12	49.12448499	0.0216	0.0229	0.3444
rs6235	**BGL**	G	C	-0.0341143	0.00246149	1.00E-45	192.0766657	0.0076	0.0188	0.6849
rs62401198	**BGL**	T	C	0.0179143	0.00299169	2.70E-09	35.85620623	0.0292	0.0227	0.1977
rs665401	**BGL**	C	T	-0.0189493	0.00233353	1.20E-16	65.9412836	0.0033	0.0162	0.8392
rs6700065	**BGL**	G	T	-0.01719	0.00230383	6.00E-14	55.67352578	0.0334	0.0205	0.103
rs67611724	**BGL**	T	C	0.0168388	0.0030053	6.60E-09	31.39384037	-0.1375	0.0654	0.0355402
rs6770013	**BGL**	C	A	-0.0120706	0.00222361	2.10E-08	29.467162	-0.0083	0.0161	0.6076
rs6785881	**BGL**	T	C	-0.0147854	0.00219443	3.00E-12	45.39630695	-0.0084	0.0189	0.6568
rs689	**BGL**	T	A	-0.0244825	0.00243238	9.10E-25	101.3086457	-0.078	0.0357	0.0289501
rs714550	**BGL**	A	G	-0.0149353	0.00234147	1.30E-11	40.68633181	0.0121	0.0159	0.4444
rs71584073	**BGL**	C	T	-0.0294213	0.0040858	1.70E-13	51.85221817	0.0134	0.0793	0.8661
rs7177055	**BGL**	A	G	0.0194406	0.00241681	9.90E-17	64.70415078	-0.0086	0.0163	0.6
rs7178424	**BGL**	T	C	0.0165696	0.00219018	4.60E-14	57.23507579	0.0084	0.0162	0.6024
rs7209715	**BGL**	G	A	-0.0124154	0.00218584	3.20E-09	32.26134914	0.0166	0.0247	0.5029
rs72695653	**BGL**	T	C	-0.0285099	0.00312906	3.60E-19	83.01592995	-0.0423	0.0777	0.5864
rs72973028	**BGL**	C	A	0.0168602	0.00290589	3.50E-10	33.66393924	0.1128	0.0605	0.0624597
rs73174306	**BGL**	T	A	0.0494324	0.00539619	5.60E-20	83.91638337	-0.1528	0.1656	0.356

rs7343623	**BGL**	C	A	-0.019069	0.00278932	1.50E-12	46.7365862	-0.0122	0.0338	0.717399
rs74628648	**BGL**	T	C	-0.0439015	0.00467924	1.00E-21	88.02497444	0.1634	0.0892	0.0669807
rs7539775	**BGL**	A	G	0.0138831	0.00249328	9.60E-09	31.00477815	-5.00E-04	0.016	0.9763
rs76701589	**BGL**	T	G	0.0176966	0.0030304	3.60E-09	34.10182218	0.0613	0.058	0.2905
rs77059113	**BGL**	G	T	-0.0432864	0.00415179	5.20E-24	108.7001168	0.0343	0.1212	0.777301
rs7708285	**BGL**	A	G	-0.0241886	0.00235296	1.50E-25	105.6793681	0.0604	0.0422	0.1519
rs7756992	**BGL**	G	A	0.0281082	0.0024643	2.30E-29	130.0998354	0.0082	0.0159	0.6036
rs78444298	**BGL**	A	G	0.057796	0.00800015	3.90E-14	52.19118239	-0.124	0.1818	0.4952
rs7903146	**BGL**	T	C	0.0566077	0.002392	2.00E-129	560.0496106	-0.0051	0.0333	0.8782
rs79438656	**BGL**	C	G	0.0237305	0.00412658	7.30E-09	33.06976211	0.0524	0.0293	0.0737904
rs7994844	**BGL**	T	C	0.0123895	0.00223102	2.90E-08	30.83887055	-0.0211	0.0167	0.2061
rs80097396	**BGL**	G	A	-0.0306982	0.00400365	6.70E-14	58.79108087	-0.0722	0.0905	0.4251
rs8070651	**BGL**	G	A	-0.0204002	0.00273758	1.30E-13	55.53068652	-0.0074	0.0173	0.668399
rs830576	**BGL**	G	A	0.0233499	0.0030501	1.00E-14	58.60568202	-0.0383	0.0194	0.0478795
rs849134	**BGL**	G	A	-0.0183192	0.00216863	8.80E-17	71.3575477	-0.0369	0.0189	0.0503802
rs881858	**BGL**	A	G	-0.0203906	0.0023566	1.80E-18	74.86625252	-0.0241	0.0231	0.2964
rs883541	**BGL**	A	G	-0.0202133	0.00259377	6.70E-16	60.73084849	-0.0137	0.0161	0.3971
rs896854	**BGL**	C	T	-0.0180664	0.00217654	2.10E-16	68.89818291	-0.0102	0.0169	0.5463
rs9397587	**BGL**	A	G	0.0148957	0.00222662	4.40E-11	44.75354619	-0.045	0.0185	0.0150699
rs9825379	**BGL**	A	G	-0.034861	0.00463331	1.20E-14	56.6102139	0.0516	0.0191	0.00695392
rs9987289	**BGL**	G	A	-0.0526925	0.00401047	6.30E-42	172.6254785	-0.0276	0.0536	0.6062

BGL: Blood glucose levels. EA: effect allele. OA: other allele. GX: beta-exposure. GX(SE): standard error of GX. GY: beta-outcome. GY(SE): standard error of GY.

Table S10. Published associations of ABL on PTB

SNP	Exposure	EA	OA	GX	GX(SE)	Pval-exp	F	GY	GY(SE)	Pval-outcome
rs10151805	**ABL**	C	T	-0.0314275	0.00249553	2.80E-38	158.5958846	0.0719	0.0536	0.1795
rs10419198	**ABL**	T	C	-0.0729101	0.00246127	1.60E-200	877.5153154	0.0261	0.0199	0.1889
rs10468017	**ABL**	T	C	-0.017187	0.00234766	2.40E-14	53.59540866	0.0085	0.0191	0.6548
rs10517086	**ABL**	A	G	-0.0189312	0.00232501	1.40E-15	66.29861723	0.0197	0.051	0.6997
rs1052620	**ABL**	T	C	0.0196458	0.00256508	2.60E-14	58.65910625	0.1252	0.0572	0.0285102
rs10740118	**ABL**	C	G	-0.0244361	0.00217519	2.40E-29	126.202287	0.011	0.0158	0.486
rs10743939	**ABL**	A	G	-0.0149159	0.00262132	1.80E-08	32.37849644	-0.0102	0.0167	0.5438
rs10754196	**ABL**	G	A	0.0247903	0.00230337	2.10E-27	115.8334486	0.0109	0.0167	0.5114
rs1076540	**ABL**	T	C	0.017987	0.00251541	6.20E-13	51.13258355	-0.0053	0.0338	0.8748
rs10828249	**ABL**	A	G	-0.0175704	0.00225647	4.00E-15	60.6320152	-0.074	0.0377	0.04976
rs10859638	**ABL**	G	A	-0.0161591	0.00253712	5.50E-11	40.56487603	-0.026	0.032	0.4152
rs10891050	**ABL**	A	G	-0.0169472	0.00257546	2.90E-11	43.29962397	-0.0179	0.0164	0.2741
rs10899106	**ABL**	C	A	0.0173876	0.00284408	4.90E-10	37.37605829	1.00E-04	0.0164	0.9947
rs10979872	**ABL**	G	T	-0.0193373	0.00227215	1.60E-16	72.42950316	0.0332	0.0163	0.0411604
rs11057273	**ABL**	C	T	-0.0257569	0.00369198	1.60E-13	48.6705594	-0.0031	0.0184	0.8668
rs11078597	**ABL**	C	T	0.0680968	0.00274297	4.90E-141	616.324066	0.0156	0.0198	0.429

rs11136344	**ABL**	C	T	0.0158493	0.00220909	4.20E-13	51.47438731	-0.0016	0.02	0.9378
rs11217129	**ABL**	T	C	-0.0269373	0.0021288	2.50E-37	160.1165547	-0.0202	0.0197	0.3055
rs1122326	**ABL**	C	A	-0.03755	0.002562	8.80E-49	214.8124584	-0.0254	0.0207	0.2208
rs11230633	**ABL**	C	T	0.016723	0.00225657	1.80E-13	54.91975522	-0.0022	0.0514	0.9655
rs1128249	**ABL**	T	G	-0.0266546	0.00218518	4.00E-35	148.7879427	-0.0632	0.0262	0.0159599
rs1133400	**ABL**	G	A	0.0163502	0.00260122	1.60E-10	39.50843287	-0.0427	0.0176	0.0151098
rs11589479	**ABL**	A	G	0.0469787	0.00292369	1.30E-57	258.1886689	0.0498	0.0677	0.4616
rs116149723	**ABL**	A	G	-0.0482399	0.00673162	1.20E-13	51.35366541	-0.0412	0.1324	0.755499
rs116307916	**ABL**	T	C	-0.0387864	0.00592339	4.10E-11	42.8761836	-0.1262	0.1631	0.4389
rs11668319	**ABL**	G	A	0.0196544	0.00305202	1.50E-11	41.47082394	-0.0209	0.0168	0.2148
rs11759553	**ABL**	T	A	0.0198404	0.0024045	3.00E-17	68.08463571	0.0139	0.0164	0.3949
rs11781886	**ABL**	T	C	-0.0157592	0.00256173	7.90E-10	37.84421137	0.0124	0.0164	0.4509
rs11871801	**ABL**	C	A	0.0158586	0.00244081	1.90E-11	42.21429842	0.0061	0.0216	0.778901
rs11928797	**ABL**	A	C	0.0279438	0.00334061	9.30E-17	69.97085948	0.0798	0.0681	0.2413
rs12209602	**ABL**	C	T	-0.0166201	0.00299438	3.40E-08	30.80713217	3.00E-04	0.0325	0.993
rs12239046	**ABL**	C	T	-0.0136789	0.00220991	1.80E-09	38.31342778	-0.002	0.0161	0.9007
rs12243326	**ABL**	C	T	0.0154886	0.00237742	1.50E-10	42.44341676	0.0072	0.0386	0.8512
rs1229984	**ABL**	C	T	0.0461663	0.0068739	5.30E-13	45.10675166	-0.0166	0.0205	0.4184
rs12453576	**ABL**	T	C	-0.0167311	0.00279271	1.60E-09	35.89179208	0.0021	0.0197	0.9146
rs12516449	**ABL**	A	G	0.0129097	0.00227489	6.40E-09	32.20396386	2.00E-04	0.0171	0.9921
rs1260326	**ABL**	C	T	-0.0637508	0.00220577	9.29E-188	835.3118842	0.0031	0.0159	0.8472
rs12619647	**ABL**	G	T	-0.0231356	0.0023199	7.80E-24	99.45367547	0.0054	0.0165	0.741401
rs12709364	**ABL**	G	A	-0.04067	0.00663997	1.30E-09	37.51577686	-0.0137	0.1229	0.9112
rs12881869	**ABL**	T	C	-0.0525254	0.00426983	2.80E-36	151.3266444	0.0386	0.0708	0.5858
rs12897338	**ABL**	T	C	0.0130683	0.00214209	9.90E-10	37.21864839	0.0201	0.0199	0.3122
rs1292045	**ABL**	C	T	0.0190929	0.00213934	7.90E-20	79.64930794	0.0153	0.016	0.3375
rs13107325	**ABL**	T	C	-0.0499115	0.00412466	4.20E-34	146.4275404	0.0932	0.1558	0.5499
rs13235543	**ABL**	T	C	-0.0270009	0.00320448	4.30E-18	70.99686689	0.0019	0.026	0.9412
rs13390891	**ABL**	C	T	-0.0210373	0.00224673	4.60E-21	87.67505869	-0.0165	0.016	0.304
rs1378942	**ABL**	A	C	0.016424	0.00228956	1.10E-13	51.45785136	-0.0069	0.0192	0.720701
rs1414518	**ABL**	C	T	0.019372	0.0024267	2.30E-16	63.72569404	-0.0044	0.0312	0.8869
rs14347	**ABL**	G	T	0.0164327	0.00236916	1.60E-12	48.10906361	0.0035	0.016	0.8246
rs1471839	**ABL**	T	C	0.0163681	0.00216752	3.90E-14	57.02537449	-0.0498	0.0181	0.00591003
rs1497406	**ABL**	G	A	0.0270991	0.00215809	2.30E-37	157.677042	-8.00E-04	0.0195	0.9692
rs157934	**ABL**	C	T	-0.0194788	0.00232834	1.60E-17	69.98894258	0.0326	0.0161	0.0429398
rs1675382	**ABL**	G	A	-0.0123625	0.00215167	3.70E-09	33.0110329	-0.0096	0.0183	0.6002
rs17001890	**ABL**	G	A	-0.0151517	0.00245439	5.50E-10	38.10951428	-0.0151	0.0269	0.5743
rs17023530	**ABL**	A	T	-0.0406658	0.00500092	2.10E-17	66.12362588	0.0237	0.0394	0.5478
rs17036326	**ABL**	G	A	-0.0473354	0.00327887	5.50E-49	208.4114331	0.0066	0.0388	0.8659
rs17085249	**ABL**	A	G	-0.0166496	0.00248302	4.90E-12	44.96193637	0.0242	0.0173	0.1632
rs17151639	**ABL**	G	A	-0.0154962	0.00239848	4.70E-11	41.7422694	-0.0307	0.0343	0.3708
rs17580	**ABL**	A	T	0.10744	0.00509807	3.70E-101	444.1383188	0.188	0.1921	0.3278
rs17712208	**ABL**	A	T	0.0526534	0.00579316	2.20E-20	82.60752821	-0.359	0.1702	0.0349301
rs1782455	**ABL**	A	G	0.0162145	0.00294225	2.80E-08	30.37007138	-0.0096	0.0233	0.68

rs1791936	**ABL**	A	G	0.0225385	0.00218881	7.90E-26	106.0307232	-0.011	0.0191	0.5636
rs1799852	**ABL**	T	C	0.022805	0.00369533	5.10E-10	38.08478701	-0.0049	0.0203	0.8092
rs1801274	**ABL**	G	A	0.0202685	0.00216769	5.50E-21	87.42722704	0.0236	0.0192	0.2193
rs1809423	**ABL**	C	T	-0.0124474	0.0021856	9.50E-09	32.43498948	0.0343	0.0188	0.0680205
rs1836828	**ABL**	A	G	0.0187199	0.00299653	1.10E-09	39.02722053	0.0204	0.0353	0.5629
rs1837913	**ABL**	C	T	-0.0321107	0.00242355	4.60E-41	175.5469999	0.0038	0.0162	0.815
rs1865076	**ABL**	A	C	0.0159262	0.00238634	4.50E-11	44.54074964	4.00E-04	0.0213	0.9862
rs1874207	**ABL**	T	C	0.0132984	0.00215902	2.20E-10	37.93877348	-0.03	0.0199	0.1311
rs1973878	**ABL**	G	A	-0.012531	0.00224692	4.50E-08	31.10241226	-0.009	0.0213	0.6709
rs2000999	**ABL**	A	G	0.0301061	0.00292376	2.70E-25	106.0286893	-0.0166	0.0166	0.3162
rs2001945	**ABL**	C	G	-0.020521	0.002145	4.60E-23	91.52511459	0.0164	0.0159	0.3004
rs2039345	**ABL**	C	G	-0.0152179	0.00264908	1.10E-08	33.000243	0.0157	0.0319	0.6226
rs205810	**ABL**	A	G	-0.0139113	0.00226222	5.40E-10	37.81495528	1.00E-04	0.0158	0.9929
rs2072728	**ABL**	G	A	-0.0182419	0.00225695	4.20E-16	65.32720825	-0.0112	0.0171	0.5117
rs2072798	**ABL**	A	G	0.0164342	0.00237729	6.80E-13	47.78929705	0.0119	0.0158	0.452101
rs2227827	**ABL**	T	C	-0.048114	0.00504102	2.40E-22	91.09696755	0.2816	0.1166	0.0157101
rs2228213	**ABL**	A	G	0.0123197	0.002275	2.70E-08	29.32481603	0.0205	0.0175	0.2427
rs2229357	**ABL**	A	G	-0.0275934	0.002518	8.20E-30	120.0872267	-0.0576	0.027	0.0329997
rs2273007	**ABL**	C	T	-0.0258442	0.00398822	2.80E-11	41.99192712	0.0056	0.0192	0.769899
rs2274720	**ABL**	C	G	0.0164135	0.00304743	4.50E-08	29.00899983	-0.001	0.0203	0.961
rs2289790	**ABL**	C	T	0.0176802	0.00252814	3.10E-12	48.90687802	-0.0325	0.0167	0.0510199
rs2293476	**ABL**	C	G	-0.0224469	0.00254851	4.70E-19	77.57787747	-0.0272	0.0218	0.2136
rs2301029	**ABL**	T	C	-0.0148348	0.00257917	6.90E-09	33.08272597	0.0106	0.022	0.6285
rs234051	**ABL**	G	A	0.0185153	0.00236859	1.00E-14	61.10539521	-0.0026	0.0178	0.8831
rs2389858	**ABL**	A	T	0.025532	0.00214556	9.60E-34	141.6074744	-0.0053	0.0159	0.739399
rs2553808	**ABL**	T	C	-0.0184169	0.00227612	9.00E-17	65.46973511	-0.0389	0.0179	0.0298099
rs2586886	**ABL**	T	C	0.0139828	0.00218807	4.30E-11	40.83792827	0.0039	0.0188	0.8349
rs2638315	**ABL**	C	G	0.0380129	0.0027655	1.30E-43	188.9350419	0.0109	0.025	0.662799
rs2696671	**ABL**	G	A	-0.0269305	0.00256089	3.00E-26	110.5871889	0.0499	0.0799	0.5321
rs2710804	**ABL**	C	T	-0.0164542	0.00220894	2.20E-13	55.4860154	-0.0085	0.0199	0.6693
rs2736231	**ABL**	T	C	-0.0137091	0.0024287	3.20E-08	31.86162903	-0.0226	0.016	0.1568
rs27744	**ABL**	T	C	-0.0199928	0.00333403	4.60E-10	35.95887284	0.0231	0.0225	0.3039
rs28444870	**ABL**	T	G	0.0284849	0.00237557	4.00E-33	143.7777118	0.0379	0.0571	0.5071
rs28925904	**ABL**	T	C	-0.0388472	0.00697242	2.80E-08	31.04203589	-0.3173	0.29	0.2739
rs28929474	**ABL**	T	C	0.276408	0.00773625	1.00E-200	1276.550674	0.2462	0.1735	0.156
rs2909210	**ABL**	T	G	0.020406	0.00213591	2.70E-22	91.2741296	0.0066	0.0181	0.715801
rs2923089	**ABL**	T	C	0.017693	0.00214345	4.60E-17	68.13559835	-0.007	0.029	0.8094
rs296894	**ABL**	T	G	0.0174223	0.00269518	1.50E-11	41.78623656	0.1049	0.0636	0.0993505
rs2972143	**ABL**	G	A	0.0187129	0.00223133	3.10E-17	70.33191104	-0.0167	0.0253	0.5088
rs3087243	**ABL**	A	G	0.0120419	0.00214746	2.20E-09	31.44399366	0.0062	0.0176	0.724499
rs3213868	**ABL**	G	A	-0.013662	0.00255118	3.40E-08	28.67769176	-0.0088	0.0164	0.5898
rs329926	**ABL**	G	A	0.0137602	0.00216213	1.60E-10	40.50267626	-0.0151	0.0232	0.5145
rs33994242	**ABL**	G	A	0.0168494	0.00293676	4.60E-09	32.91772418	-0.008	0.0249	0.7471
rs339969	**ABL**	A	C	0.0255345	0.00219824	4.30E-32	134.9280791	-0.0184	0.0252	0.4667

rs34006994	**ABL**	T	C	-0.0177161	0.00215688	2.80E-17	67.4655574	-0.0189	0.0172	0.2712
rs34562254	**ABL**	A	G	-0.0424852	0.00358844	1.00E-32	140.1721677	-0.008	0.017	0.6369
rs34931250	**ABL**	T	C	0.0388367	0.00474938	1.90E-16	66.86650686	0.2589	0.0986	0.00867801
rs3740688	**ABL**	T	G	0.0236921	0.00217263	7.30E-30	118.9141138	0.0249	0.0164	0.1287
rs3742716	**ABL**	A	G	-0.0140938	0.00229488	2.00E-10	37.7167341	-0.0194	0.0158	0.22
rs3744274	**ABL**	A	C	-0.0186726	0.00225741	7.00E-17	68.4205455	0.0276	0.0283	0.3291
rs3751261	**ABL**	T	C	0.0144238	0.00222121	1.60E-10	42.167512	0.03	0.0186	0.1057
rs3753332	**ABL**	G	A	0.0160194	0.00228081	1.40E-12	49.33011276	0.029	0.0202	0.1516
rs3756772	**ABL**	T	C	0.0128545	0.00219087	2.70E-09	34.42508282	-0.0011	0.0171	0.9489
rs3762281	**ABL**	G	A	-0.0167158	0.00217236	1.10E-14	59.20911521	6.00E-04	0.0158	0.9719
rs3810484	**ABL**	G	A	0.0132564	0.0023149	3.50E-09	32.79325785	0.027	0.0224	0.2284
rs38249	**ABL**	G	A	-0.0162792	0.0024541	7.10E-11	44.00271162	-0.0344	0.0221	0.1195
rs4409785	**ABL**	C	T	0.0173273	0.00283287	6.00E-10	37.41160433	-0.0262	0.0283	0.3545
rs4420065	**ABL**	C	T	-0.0247334	0.00220793	3.60E-30	125.4858842	-0.0047	0.0216	0.826
rs4530754	**ABL**	A	G	0.0216388	0.00214631	1.30E-24	101.6434228	-0.0213	0.0168	0.206
rs4540292	**ABL**	G	T	0.0223457	0.00298578	4.50E-14	56.01059061	0.0031	0.0159	0.8452
rs45512696	**ABL**	T	C	0.0866554	0.00287424	1.00E-200	908.9563712	-0.0246	0.0411	0.5488
rs459193	**ABL**	G	A	0.0218864	0.00245684	6.90E-20	79.35837192	-0.0128	0.0159	0.421
rs460879	**ABL**	T	C	0.0138538	0.00218509	4.00E-10	40.197311	-0.0115	0.0158	0.4648
rs4650994	**ABL**	A	G	0.0236823	0.00213606	1.20E-28	122.9189085	-0.0219	0.0158	0.1657
rs4704045	**ABL**	G	A	-0.042906	0.00215887	2.80E-92	394.9852771	0.0031	0.0159	0.8447
rs4730221	**ABL**	C	A	-0.0126765	0.0021685	1.60E-09	34.17257475	-0.0144	0.0171	0.4005
rs4804416	**ABL**	G	T	0.0152953	0.00215934	3.70E-13	50.17319681	0.0193	0.016	0.2281
rs483809	**ABL**	G	A	-0.0191419	0.0027023	1.60E-13	50.17655002	-0.0251	0.0166	0.1305
rs4845606	**ABL**	A	G	0.0272858	0.00316793	2.20E-18	74.18567683	-0.0151	0.0215	0.4814
rs4846567	**ABL**	T	G	-0.0243319	0.00233202	1.10E-26	108.8642633	0.0162	0.0164	0.3234
rs4946811	**ABL**	C	A	0.0160802	0.00228502	2.20E-12	49.52228947	-0.0099	0.0158	0.5318
rs536841	**ABL**	T	C	-0.0147649	0.00231278	2.00E-10	40.75587366	0.0063	0.0159	0.691301
rs55707100	**ABL**	T	C	0.14896	0.00680028	6.10E-111	479.8257721	0.3604	0.257	0.1608
rs55772024	**ABL**	A	G	-0.0189768	0.00249342	3.40E-14	57.92324942	0.002	0.0159	0.9008
rs55772354	**ABL**	A	G	-0.122498	0.00778881	5.00E-58	247.3509657	0.1086	0.1747	0.5342
rs55846720	**ABL**	A	G	-0.013726	0.00216038	1.30E-10	40.36691187	0.0023	0.0162	0.8877
rs55910200	**ABL**	C	T	-0.0141747	0.00247046	2.70E-09	32.92076607	-0.0285	0.0189	0.1311
rs56094005	**ABL**	G	A	-0.0527091	0.00533253	1.70E-23	97.70176746	-0.1073	0.1158	0.354
rs56248708	**ABL**	A	G	-0.0220071	0.00335517	2.50E-11	43.02237758	-0.0379	0.0321	0.2378
rs5756813	**ABL**	T	G	-0.0195702	0.00221846	8.20E-20	77.81891234	0.011	0.0161	0.4938
rs57826934	**ABL**	T	C	0.0132974	0.00227879	2.40E-09	34.05043697	0.0061	0.0204	0.765399
rs58148580	**ABL**	T	C	0.0282666	0.00341732	3.40E-17	68.41852126	0.0479	0.0401	0.2317
rs6136885	**ABL**	A	G	-0.0143959	0.00233673	1.50E-10	37.95407465	-0.0058	0.0164	0.724499
rs61735533	**ABL**	A	G	-0.0159331	0.00274056	3.70E-09	33.80023876	-0.0186	0.0361	0.605399
rs61740705	**ABL**	G	A	0.0164297	0.00271164	3.60E-10	36.7107314	-0.0034	0.0478	0.9439
rs61754296	**ABL**	T	C	-0.0199471	0.00330748	7.80E-10	36.37164218	0.0124	0.031	0.6886
rs62226440	**ABL**	A	G	0.0161102	0.0023912	1.60E-11	45.3908059	0.0641	0.0232	0.00576103
rs62622830	**ABL**	C	T	0.0152944	0.00259602	1.80E-08	34.70936386	-0.0889	0.0593	0.1337

rs646776	**ABL**	T	C	0.0232911	0.00256759	2.70E-20	82.28610064	-0.0372	0.0281	0.1849
rs648514	**ABL**	A	G	-0.0126065	0.00214323	4.40E-10	34.59786745	0.0102	0.0167	0.5424
rs6486122	**ABL**	T	C	0.0152418	0.00231051	1.10E-11	43.51656891	-7.00E-04	0.0171	0.9692
rs653178	**ABL**	T	C	0.0238273	0.00214202	7.40E-30	123.737249	-0.0582	0.0485	0.23
rs6684353	**ABL**	G	A	-0.0141522	0.00233536	5.20E-10	36.72299333	-0.003	0.0258	0.9075
rs67199213	**ABL**	G	A	0.0161966	0.0024369	1.30E-11	44.17434627	0.0038	0.0162	0.813
rs6722472	**ABL**	C	G	-0.0206855	0.00283366	5.70E-14	53.28861097	-0.0061	0.0189	0.748201
rs6734238	**ABL**	G	A	-0.0179501	0.00217524	4.50E-17	68.09531242	0.0303	0.0302	0.3162
rs67611724	**ABL**	T	C	0.0167468	0.00296182	7.80E-09	31.97011351	-0.1375	0.0654	0.0355402
rs6831256	**ABL**	G	A	0.0348668	0.00216673	2.50E-59	258.947917	-0.0109	0.0163	0.5043
rs6840938	**ABL**	A	G	-0.016988	0.0021626	6.00E-16	61.70636176	0.0029	0.0195	0.8833
rs685791	**ABL**	T	G	0.0189526	0.00216281	1.90E-19	76.78894296	0.0192	0.0246	0.4349
rs6859219	**ABL**	A	C	0.0215866	0.00264912	5.70E-17	66.39923243	0.0283	0.0408	0.487401
rs6861681	**ABL**	A	G	0.0157253	0.00232762	3.70E-12	45.64272363	-0.0161	0.0242	0.5059
rs6873349	**ABL**	C	A	0.0223155	0.00409037	3.20E-08	29.76362943	-0.0138	0.0257	0.59
rs6897932	**ABL**	T	C	0.0153232	0.00242532	3.60E-10	39.91707201	0.0062	0.0197	0.7533
rs6935921	**ABL**	T	C	-0.0200395	0.00234095	3.30E-17	73.28031387	0.017	0.0163	0.2957
rs6963924	**ABL**	T	C	-0.0224259	0.00238749	2.30E-21	88.22963319	0.0015	0.0193	0.9384
rs6968865	**ABL**	T	A	0.016281	0.00220884	1.30E-13	54.32896975	-0.0031	0.0167	0.8524
rs7031621	**ABL**	A	G	-0.0128497	0.00220046	1.90E-09	34.10019431	-0.0233	0.0169	0.1678
rs704017	**ABL**	G	A	-0.0130029	0.00216513	2.10E-09	36.06702522	0.0032	0.0166	0.8461
rs7153110	**ABL**	G	A	-0.0118181	0.00215908	2.00E-08	29.9609573	0.004	0.0159	0.8036
rs7196578	**ABL**	T	C	0.0176617	0.00227574	2.70E-15	60.23065065	-0.0012	0.0168	0.9429
rs7219984	**ABL**	A	G	-0.0213676	0.00280757	1.50E-15	57.92261194	-0.0185	0.0183	0.312
rs725660	**ABL**	A	C	-0.0130833	0.00222071	7.30E-10	34.70953032	0.0305	0.0204	0.1338
rs7258465	**ABL**	C	T	-0.0231399	0.00224496	1.30E-24	106.2437949	7.00E-04	0.0175	0.9674
rs72631343	**ABL**	G	C	0.0235776	0.00318915	3.30E-13	54.65716877	-0.0018	0.0167	0.9126
rs72729610	**ABL**	G	A	0.0226517	0.00287934	6.10E-16	61.8890099	0.0486	0.0364	0.1821
rs7282496	**ABL**	C	A	0.0167323	0.00215051	8.30E-14	60.53773753	0.0166	0.0195	0.3954
rs72961013	**ABL**	A	G	0.0336697	0.00432219	1.40E-14	60.68322019	0.005	0.0967	0.959
rs73038384	**ABL**	T	C	-0.0555151	0.00637738	5.10E-19	75.77666638	-0.1401	0.1218	0.2499
rs731839	**ABL**	A	G	-0.0261047	0.00226587	4.40E-31	132.7288306	-0.0242	0.0158	0.1268
rs73562872	**ABL**	T	G	0.0291811	0.00417237	1.90E-12	48.91426399	-0.0172	0.0223	0.44
rs74010640	**ABL**	G	A	-0.0151751	0.00254723	5.10E-09	35.49152291	0.0054	0.0215	0.8023
rs74397112	**ABL**	T	C	0.0179051	0.00306849	5.00E-09	34.04880787	-0.0554	0.0662	0.403
rs7443182	**ABL**	A	G	-0.0121024	0.00220882	1.50E-08	30.02065663	0.0231	0.0166	0.1652
rs74438527	**ABL**	A	G	0.0516235	0.0065439	5.80E-16	62.23281083	0.1527	0.1848	0.4087
rs74758151	**ABL**	G	A	-0.0323052	0.00414905	4.10E-15	60.62410843	-0.0739	0.0789	0.3492
rs74780677	**ABL**	G	A	0.0886813	0.00898255	4.40E-23	97.46813335	0.011	0.0999	0.9124
rs75009793	**ABL**	A	G	0.033499	0.00555554	5.90E-09	36.35875147	0.038	0.0735	0.604999
rs7554117	**ABL**	C	G	0.0152256	0.00230436	7.80E-12	43.65620797	-0.0158	0.0163	0.3339
rs755731	**ABL**	T	G	0.0138681	0.0025455	2.90E-08	29.68148053	0.0167	0.0223	0.452101
rs7586970	**ABL**	C	T	0.0159311	0.00235312	9.30E-12	45.83541954	0.0081	0.0279	0.771299
rs75972158	**ABL**	T	C	0.0277533	0.00378561	1.90E-14	53.74713929	0.0755	0.0784	0.3357

rs76191003	**ABL**	T	C	0.0313296	0.00425229	2.70E-14	54.28274279	0.0242	0.034	0.4754
rs76701589	**ABL**	T	G	0.0175045	0.00298653	4.50E-09	34.35290674	0.0613	0.058	0.2905
rs7726159	**ABL**	A	C	0.0135117	0.00227957	1.60E-09	35.13273468	-0.0094	0.0173	0.587899
rs77280277	**ABL**	A	G	-0.0604643	0.00685001	2.10E-18	77.91363767	0.0105	0.0292	0.7185
rs77542162	**ABL**	G	A	-0.205393	0.00733413	2.30E-176	784.2813773	-0.3167	0.2464	0.1986
rs77704934	**ABL**	G	A	-0.018726	0.00294162	9.80E-10	40.52422635	-0.0301	0.0684	0.6595
rs77849807	**ABL**	G	A	0.110172	0.00864613	7.20E-39	162.3666305	0.094	0.1581	0.5521
rs7797854	**ABL**	C	A	0.0132507	0.00232655	1.50E-08	32.43774802	0.018	0.0201	0.3715
rs7941030	**ABL**	C	T	0.0181596	0.00219986	1.90E-17	68.14285162	0.0262	0.0162	0.1061
rs8017377	**ABL**	A	G	0.0178981	0.00220173	9.80E-16	66.08206017	-0.0601	0.0306	0.0494698
rs8045125	**ABL**	T	G	0.0220893	0.0038388	1.00E-08	33.11088343	-0.0209	0.0935	0.8232
rs8124695	**ABL**	A	C	0.0262389	0.0040658	1.90E-11	41.64827892	0.0277	0.0807	0.731799
rs8176741	**ABL**	A	G	0.0317142	0.00444696	8.10E-14	50.86029899	0.0359	0.0209	0.0861807
rs8187658	**ABL**	T	C	0.0147266	0.00269365	3.60E-08	29.88962631	-0.0053	0.0192	0.781601
rs8208	**ABL**	A	G	-0.0225682	0.00238214	4.00E-22	89.7546823	0.0225	0.0171	0.1884
rs843925	**ABL**	T	G	-0.0136285	0.00228033	2.10E-09	35.7189389	0.0156	0.0171	0.3621
rs848486	**ABL**	A	G	0.0113966	0.00217955	2.60E-08	27.34102374	0.0097	0.0186	0.6009
rs869337	**ABL**	A	G	0.012795	0.00214682	1.80E-09	35.52116074	0.0122	0.0271	0.6522
rs870526	**ABL**	T	C	-0.0116685	0.00213775	1.50E-08	29.79298819	0.0178	0.0159	0.2637
rs872071	**ABL**	G	A	-0.0140311	0.00215139	2.50E-11	42.53466161	0.0013	0.0166	0.9386
rs8978	**ABL**	T	C	0.0138798	0.0021881	3.50E-10	40.23740069	-0.0111	0.0158	0.4796
rs900400	**ABL**	C	T	0.0256245	0.00218749	1.40E-32	137.2197048	0.0175	0.0159	0.2723
rs9088	**ABL**	G	A	-0.022273	0.00226367	2.80E-24	96.81196547	0.0162	0.0205	0.4309
rs921835	**ABL**	G	A	0.0246001	0.00421998	2.90E-09	33.98214536	0.0196	0.0211	0.3546
rs9289250	**ABL**	C	G	0.0146747	0.00247804	3.20E-09	35.06870042	0.0264	0.016	0.0989897
rs930734	**ABL**	C	A	-0.0126896	0.00214032	5.90E-09	35.15088625	0.0141	0.0174	0.4168
rs9309325	**ABL**	G	A	0.0125474	0.0021459	8.40E-09	34.18899702	0.017	0.0169	0.3154
rs9368571	**ABL**	G	C	0.0242428	0.00383024	1.70E-10	40.06004346	-0.0152	0.0165	0.3563
rs9465733	**ABL**	C	A	0.0156525	0.00227352	2.50E-12	47.39883861	0.0184	0.0165	0.2662
rs9772460	**ABL**	G	A	0.0123636	0.00215776	1.40E-08	32.83079878	0.0062	0.0159	0.6959
rs9901675	**ABL**	A	G	-0.0477805	0.00496766	4.60E-23	92.51144958	0.0226	0.0336	0.5014
rs9976946	**ABL**	T	C	0.0431423	0.00569079	5.20E-15	57.47239601	0.1637	0.1676	0.3287
rs9987289	**ABL**	G	A	0.0729441	0.00394887	8.80E-79	341.218446	-0.0276	0.0536	0.6062

ABL: Albumin level. EA: effect allele. OA: other allele. GX: beta-exposure. GX(SE): standard error of GX. GY: beta-outcome. GY(SE): standard error of GY.

Table S11. Published associations of Mean corpuscular hemoglobin on PTB

SNP	**Exposure**	**EA**	**OA**	**GX**	**GX(SE)**	**Pval-exp**	**F**	**GY**	**GY(SE)**	**Pval-outcome**
rs10182296	**MCH**	A	G	-0.014177	0.00192666	1.70E-13	54.14483076	-0.0222	0.0198	0.2637
rs10213703	**MCH**	T	C	0.014983	0.00223439	1.20E-10	44.96536103	0.0091	0.0197	0.6425
rs1022688	**MCH**	A	G	-0.0108651	0.00199952	4.20E-08	29.52666757	-0.0091	0.0183	0.6181

rs10249013	**MCH**	T	A	-0.0189335	0.00358031	7.30E-10	27.96527077	-0.0072	0.0217	0.738501
rs1041070	**MCH**	T	C	-0.0153027	0.00222893	2.60E-11	47.13480917	0.009	0.0188	0.6323
rs10457234	**MCH**	C	T	-0.0331715	0.00264327	2.10E-39	157.4873412	0.0258	0.0171	0.1302
rs1046411	**MCH**	A	G	-0.0270293	0.00200602	2.20E-43	181.5505478	0.0208	0.0234	0.3738
rs1047891	**MCH**	A	C	0.020654	0.00200097	7.30E-26	106.5431847	-0.0375	0.0219	0.0874299
rs10496344	**MCH**	C	T	0.0133064	0.00193972	1.20E-12	47.05887551	0.0013	0.016	0.9339
rs10750385	**MCH**	G	A	0.0180262	0.00199895	4.50E-21	81.32105269	-0.0268	0.0201	0.1828
rs1075410	**MCH**	T	C	0.0120384	0.00204492	1.80E-08	34.65639444	-0.0328	0.0241	0.1735
rs10758656	**MCH**	G	A	-0.0806922	0.00230298	1.00E-200	1227.668926	-0.0096	0.0161	0.552
rs10793565	**MCH**	C	G	0.0433693	0.00193124	2.20E-116	504.302142	-0.0068	0.0166	0.681299
rs10799544	**MCH**	C	T	0.0150848	0.00185904	2.90E-17	65.84156241	-0.0157	0.0207	0.4471
rs10801682	**MCH**	T	A	0.0113762	0.00186735	5.50E-11	37.11431667	0.0113	0.0158	0.4751
rs10802933	**MCH**	G	A	0.0111501	0.00204249	2.00E-08	29.80136322	0.039	0.0546	0.4745
rs10811425	**MCH**	C	T	-0.0153997	0.00217511	4.60E-14	50.12571201	0.0381	0.0204	0.0618401
rs10846742	**MCH**	A	G	0.0176086	0.00266144	8.80E-11	43.77385352	0.0321	0.0174	0.0654892
rs10849020	**MCH**	G	C	0.0576932	0.00229676	6.19E-152	630.9813285	0.02	0.0175	0.2548
rs10900159	**MCH**	C	T	0.0104579	0.0019081	7.80E-09	30.03898058	-0.0635	0.0485	0.1907
rs10902843	**MCH**	G	A	-0.00998727	0.00188407	3.20E-08	28.09946149	0.0104	0.0159	0.511601
rs10916606	**MCH**	T	C	0.0150484	0.00227708	8.50E-13	43.67395873	0.0054	0.057	0.9245
rs10923397	**MCH**	T	C	0.0420112	0.00253173	6.80E-66	275.35558	-0.0248	0.0194	0.1997
rs10928378	**MCH**	G	A	0.0104939	0.00192172	1.40E-08	29.8189335	0.0237	0.0162	0.1423
rs10937101	**MCH**	G	T	-0.0156912	0.00200436	2.40E-17	61.28572744	0.0363	0.0165	0.0276299
rs10971930	**MCH**	C	T	0.0272188	0.00280686	1.50E-21	94.0361694	-0.0921	0.0707	0.1929
rs10974716	**MCH**	G	C	0.0222665	0.00223058	1.80E-24	99.64758781	0.0318	0.0269	0.2385
rs11030976	**MCH**	C	T	0.0264069	0.00190712	4.00E-49	191.7243545	0.0162	0.0158	0.3064
rs1107960	**MCH**	A	T	-0.015816	0.00200175	3.70E-14	62.42695071	-0.0046	0.0181	0.8007
rs11089093	**MCH**	T	C	-0.0152895	0.00190405	3.20E-17	64.48049354	0.008	0.0195	0.6798
rs11097297	**MCH**	T	C	0.0123092	0.0020881	9.20E-09	34.75005956	0.0036	0.0196	0.8557
rs11106996	**MCH**	T	C	0.0359573	0.00306675	1.90E-33	137.4725166	-0.0282	0.0178	0.1126
rs11119337	**MCH**	G	A	-0.0207611	0.00216606	6.40E-24	91.86672611	0.0137	0.016	0.3904
rs111283523	**MCH**	T	C	0.0124692	0.00249477	3.20E-08	24.98127727	-0.0139	0.0709	0.8449
rs111315083	**MCH**	G	T	-0.0127646	0.0018717	3.10E-12	46.5093664	0.0197	0.0179	0.2724
rs11159493	**MCH**	T	A	0.0120344	0.00188339	7.40E-11	40.82882187	-0.0183	0.0159	0.2502
rs11168353	**MCH**	T	C	-0.0162258	0.00292176	5.80E-09	30.84051649	-0.0292	0.0195	0.1333
rs112513907	**MCH**	T	C	-0.0289284	0.00312649	1.80E-22	85.61172026	-0.0345	0.0675	0.61
rs113059506	**MCH**	T	C	-0.0185646	0.00260337	4.60E-14	50.85081107	-0.0144	0.021	0.4943
rs1134634	**MCH**	C	G	0.0278193	0.0018936	6.40E-53	215.8312817	-0.0184	0.0159	0.246
rs113809617	**MCH**	G	C	0.0426022	0.00277617	6.70E-58	235.4888907	0.0112	0.0256	0.660999
rs113931125	**MCH**	A	G	-0.0168858	0.00192887	1.00E-20	76.63653294	0.0062	0.0173	0.7196
rs113938611	**MCH**	A	G	0.0178178	0.0030967	4.30E-09	33.10612688	0.0986	0.0927	0.2877
rs114754319	**MCH**	T	G	0.0471726	0.00726399	1.60E-11	42.17233387	0.1378	0.2211	0.5332
rs1151503	**MCH**	G	A	0.0232818	0.00219305	1.00E-28	112.7027531	0.0176	0.0283	0.5343
rs11521	**MCH**	T	C	-0.0295243	0.00234614	3.60E-39	158.3615905	0.0244	0.0161	0.1299
rs115360810	**MCH**	G	A	0.0392003	0.00650546	3.20E-10	36.30958151	-0.0655	0.229	0.774901

rs115768156	**MCH**	T	G	-0.0492599	0.00816583	1.20E-10	36.39023556	0.1101	0.2287	0.630099
rs11581732	**MCH**	T	G	0.0161898	0.0023054	2.00E-13	49.31611693	-0.0162	0.0184	0.379
rs11591710	**MCH**	C	A	-0.0279331	0.0027917	1.70E-24	100.1150256	0.0222	0.0702	0.751299
rs116034739	**MCH**	A	T	-0.0362893	0.00623925	1.30E-10	33.82913478	0.0309	0.1398	0.825
rs11610160	**MCH**	A	G	-0.0238165	0.00232113	4.00E-26	105.2823167	0.0277	0.0634	0.662599
rs116135446	**MCH**	T	C	0.0297059	0.00454357	7.20E-12	42.74540798	0.0596	0.12	0.6195
rs11627485	**MCH**	C	T	0.0586033	0.00189076	1.00E-200	960.6602394	-0.0821	0.0481	0.0879508
rs11635810	**MCH**	T	C	0.0160415	0.00302392	2.20E-08	28.14153873	0.0137	0.0765	0.8575
rs11650788	**MCH**	A	T	0.066144	0.00235921	5.90E-183	786.042798	0.0257	0.017	0.1302
rs116597408	**MCH**	G	A	-0.0368928	0.00521005	1.10E-13	50.14157095	-0.1668	0.1223	0.1726
rs11664929	**MCH**	A	G	0.0183369	0.00291165	5.30E-11	39.66175976	0.1247	0.1058	0.2386
rs116726424	**MCH**	T	C	0.0237034	0.00469446	1.30E-08	25.4946168	0.012	0.1228	0.9221
rs117107603	**MCH**	A	C	-0.0650828	0.0074022	2.20E-20	77.30530739	-0.0441	0.0932	0.636
rs117111916	**MCH**	T	C	0.0241731	0.00408848	1.80E-10	34.9574243	0.0021	0.1025	0.9834
rs11716021	**MCH**	C	T	0.00975937	0.00186498	1.60E-08	27.38379866	-0.0011	0.0161	0.9436
rs117246501	**MCH**	C	A	-0.032782	0.00569715	1.30E-08	33.10961017	0.2721	0.203	0.1801
rs117747069	**MCH**	C	G	-0.242072	0.00594974	1.00E-200	1655.35683	0.1491	0.1857	0.422
rs11807602	**MCH**	G	C	-0.0179458	0.00240527	1.10E-15	55.66682625	-0.1362	0.1429	0.3407
rs118108907	**MCH**	A	G	0.041293	0.0051748	4.20E-16	63.67430556	-0.0022	0.1249	0.9859
rs11816944	**MCH**	T	C	0.0188828	0.00337548	2.20E-08	31.29393615	-0.0106	0.046	0.8185
rs11894425	**MCH**	C	T	0.0111166	0.00190515	7.50E-10	34.04741088	0.0015	0.0161	0.9271
rs12000236	**MCH**	G	A	0.0169934	0.00209282	3.20E-14	65.93185429	0.0416	0.0207	0.0448301
rs12001675	**MCH**	A	G	-0.0317635	0.00226175	6.50E-50	197.226827	-0.0097	0.0284	0.7329
rs12026918	**MCH**	G	C	0.0301988	0.00232943	1.10E-40	168.0654826	-0.0279	0.0182	0.1261
rs12033311	**MCH**	T	C	-0.0633453	0.00497308	6.50E-39	162.2468923	0.001	0.0229	0.966
rs12145660	**MCH**	T	C	-0.0159285	0.00190198	8.30E-17	70.13525038	-0.0038	0.0337	0.9094
rs12146644	**MCH**	G	A	-0.0171932	0.00192631	3.40E-19	79.66353165	0.0035	0.017	0.8354
rs12196049	**MCH**	C	A	-0.0210844	0.00229098	2.80E-22	84.69901926	0.0099	0.0169	0.559899
rs12232375	**MCH**	C	G	-0.071065	0.00639009	6.70E-31	123.6790255	0.0325	0.0426	0.4449
rs1229984	**MCH**	C	T	0.0517605	0.00589046	5.80E-19	77.2141664	-0.0166	0.0205	0.4184
rs12378537	**MCH**	T	C	-0.0392731	0.00348857	2.10E-30	126.7342407	0.0038	0.0188	0.8399
rs12416288	**MCH**	T	G	-0.0134573	0.00211814	3.30E-11	40.36500667	0.0089	0.0167	0.5937
rs12439534	**MCH**	A	C	-0.0138296	0.00188917	1.20E-15	53.58899853	-0.0122	0.016	0.4479
rs12447005	**MCH**	G	C	0.0177336	0.00276547	1.10E-10	41.12012225	0.0079	0.0267	0.7683
rs12479919	**MCH**	T	C	0.0151363	0.00194541	1.20E-16	60.53626882	0.0248	0.0168	0.1392
rs12494775	**MCH**	A	G	-0.0150449	0.00241395	1.40E-09	38.84369666	0.0157	0.0276	0.57
rs12573992	**MCH**	G	A	-0.0306613	0.00188871	2.00E-65	263.5415241	-0.0491	0.0168	0.00354299
rs12599260	**MCH**	A	G	-0.0202282	0.00205455	1.80E-23	96.93475835	0.0089	0.0163	0.5866
rs12601867	**MCH**	G	C	0.0118343	0.0018992	2.30E-12	38.82775043	-0.0141	0.0159	0.3756
rs12605189	**MCH**	G	A	-0.0118953	0.00199008	2.00E-10	35.72795941	0.0315	0.019	0.09749
rs12641022	**MCH**	T	C	0.0126718	0.00187324	2.10E-11	45.76023622	-0.0037	0.0161	0.8185
rs12742691	**MCH**	T	C	0.0144024	0.00204164	2.30E-12	49.76338211	-0.0036	0.0163	0.8229
rs1275189	**MCH**	C	T	-0.0135093	0.00197971	2.80E-13	46.56515173	-0.0102	0.0219	0.6418
rs1283686	**MCH**	T	C	-0.0147375	0.00190038	7.10E-15	60.14024841	0.0034	0.0163	0.8343

rs12975585	**MCH**	T	A	0.0112654	0.00193135	5.50E-11	34.02277629	-0.0045	0.0179	0.8033
rs12976094	**MCH**	A	G	0.0241328	0.00289783	2.20E-17	69.35345524	0.0085	0.0946	0.9285
rs13007705	**MCH**	T	C	0.0212547	0.00188288	6.30E-31	127.4274986	-0.0018	0.0165	0.911
rs13250272	**MCH**	G	A	-0.0275071	0.00221348	1.60E-36	154.4318563	0.003	0.0544	0.9557
rs13355364	**MCH**	T	C	-0.0120198	0.00189201	5.20E-11	40.35953107	0.0185	0.0166	0.2657
rs13414216	**MCH**	C	T	0.0107412	0.00186698	3.30E-09	33.09975345	-0.0037	0.0159	0.8155
rs1360950	**MCH**	A	G	-0.0125416	0.00214517	3.70E-08	34.1806994	0.0168	0.0185	0.3639
rs140446749	**MCH**	A	G	-0.0899988	0.00872368	8.60E-28	106.4320601	-0.2628	0.2524	0.2978
rs140522	**MCH**	C	T	0.0672086	0.00198323	1.00E-200	1148.423352	-0.0027	0.0177	0.8789
rs141382271	**MCH**	A	G	0.115409	0.00825233	2.00E-43	195.5801013	0.1046	0.1748	0.5494
rs141597584	**MCH**	A	G	0.0369497	0.00439753	1.00E-18	70.59967393	-0.0501	0.1169	0.667999
rs141844044	**MCH**	A	G	0.0313955	0.00500424	1.70E-10	39.360176	-0.2314	0.1337	0.0835391
rs141866277	**MCH**	T	A	-0.0574863	0.00614298	1.30E-22	87.57275144	0.3198	0.2673	0.2315
rs1423056	**MCH**	A	G	-0.015534	0.00210273	3.60E-16	54.57554277	0.0318	0.0242	0.188
rs142980456	**MCH**	C	G	0.0380631	0.00517876	1.20E-14	54.02008035	-0.2115	0.1389	0.1278
rs143270123	**MCH**	G	A	0.0428257	0.00704755	4.10E-10	36.92590033	-0.0929	0.1182	0.4322
rs143747543	**MCH**	A	C	-0.0306918	0.00395958	1.30E-15	60.08207995	-0.0483	0.1132	0.669501
rs143759545	**MCH**	G	C	-0.0784262	0.00542543	1.10E-50	208.9548712	-0.0026	0.0308	0.9318
rs144204502	**MCH**	T	C	0.0957638	0.00847955	1.00E-29	127.5427025	0.0511	0.1347	0.704301
rs144942726	**MCH**	T	C	-0.0382902	0.00520238	1.00E-14	54.17134399	-0.0211	0.1639	0.8976
rs144991697	**MCH**	A	G	0.102931	0.00614493	1.50E-65	280.580179	0.0707	0.0809	0.3821
rs146008976	**MCH**	T	C	-0.107606	0.0114424	1.20E-20	88.43765292	-0.3669	0.4296	0.3931
rs146220482	**MCH**	T	C	0.0588545	0.00904355	4.10E-12	42.35258714	-0.2664	0.1667	0.11
rs147707926	**MCH**	T	C	-0.0666587	0.00720904	1.10E-21	85.49826319	-0.0205	0.117	0.8606
rs148125759	**MCH**	C	T	-0.0709976	0.00836503	5.30E-17	72.03620317	-0.0173	0.3751	0.9632
rs149200430	**MCH**	T	G	-0.0575132	0.00810853	7.20E-14	50.3094179	0.4888	0.3271	0.1351
rs149290349	**MCH**	A	G	0.0454915	0.00357308	4.70E-39	162.0964554	0.0899	0.1208	0.4569
rs1533237	**MCH**	G	A	0.0223278	0.00432184	5.00E-09	26.69027085	-0.007	0.0174	0.6887
rs1533378	**MCH**	T	C	0.026688	0.00287635	4.20E-21	86.08888445	-0.0349	0.0302	0.2479
rs154275	**MCH**	T	C	0.0145877	0.00204411	9.90E-13	50.92881893	0.0013	0.0209	0.952
rs1558151	**MCH**	A	T	-0.0136927	0.00226834	1.70E-08	36.43848965	1.00E-04	0.0293	0.9976
rs1569419	**MCH**	C	T	-0.0271538	0.00221832	2.10E-35	149.8343279	-0.0161	0.0268	0.547
rs165975	**MCH**	C	T	-0.0155819	0.00220451	7.50E-14	49.95916408	-0.0122	0.0162	0.4517
rs17012334	**MCH**	A	G	-0.0335289	0.00234573	1.30E-49	204.3059668	-0.0187	0.0168	0.2656
rs17116384	**MCH**	G	A	-0.0251196	0.00201444	3.30E-38	155.4945779	0.004	0.0173	0.8179
rs17116710	**MCH**	A	G	0.0247182	0.00239292	7.90E-26	106.7027982	-0.0173	0.0198	0.3835
rs17302154	**MCH**	C	A	-0.020321	0.00278526	1.90E-13	53.23008395	-0.0451	0.0485	0.3524
rs1730872	**MCH**	G	A	0.0189767	0.00244798	1.70E-12	60.09303479	0.0442	0.0331	0.1822
rs17476364	**MCH**	C	T	0.0724656	0.00301005	1.10E-136	579.5819466	-0.1129	0.0968	0.2433
rs17501461	**MCH**	T	C	-0.0138482	0.00246579	2.10E-08	31.54081885	0.0388	0.03	0.1962
rs17699658	**MCH**	T	C	-0.0215805	0.00226559	3.20E-22	90.73165879	-0.0196	0.0384	0.6101
rs17728589	**MCH**	T	C	0.0192746	0.00352492	3.90E-08	29.89996652	0.0476	0.0913	0.6024
rs17749725	**MCH**	C	A	0.0112508	0.0018821	1.30E-09	35.73385768	0.0024	0.0176	0.8928
rs17758695	**MCH**	T	C	0.0984569	0.00555829	8.10E-79	313.7678159	-0.07	0.1222	0.5668

rs17779838	**MCH**	G	A	-0.029527	0.00475131	5.80E-09	38.61983358	0.0437	0.1602	0.785301
rs17806888	**MCH**	C	T	0.0205912	0.00288911	2.30E-13	50.79648282	-0.0496	0.0435	0.2543
rs17819562	**MCH**	G	A	-0.0439428	0.00765667	4.00E-09	32.93776126	-0.2941	0.1856	0.113
rs1800562	**MCH**	A	G	0.276093	0.00370088	1.00E-200	5565.436968	0.0282	0.1158	0.8077
rs1811069	**MCH**	G	T	-0.0340739	0.00236776	1.20E-48	207.0936649	0.0142	0.016	0.3728
rs1822569	**MCH**	G	C	-0.0135466	0.00253405	1.80E-08	28.57779646	0.0967	0.0638	0.13
rs186137462	**MCH**	A	T	-0.0662148	0.0092342	1.40E-13	51.41739548	-0.6892	0.5678	0.2248
rs1877030	**MCH**	C	T	0.0279458	0.00259079	2.00E-29	116.350205	-0.0515	0.0219	0.01883
rs190399027	**MCH**	A	G	0.12629	0.0081715	1.10E-59	238.8541768	-0.0279	0.2384	0.9068
rs1909350	**MCH**	A	G	-0.0147015	0.0021915	5.10E-12	45.0027269	-0.0142	0.0182	0.4339
rs1913467	**MCH**	A	G	-0.0334776	0.00583955	3.10E-10	32.86611368	0.3395	0.1333	0.01088
rs1935958	**MCH**	T	C	-0.018181	0.00254758	1.20E-13	50.9305488	-0.0389	0.0726	0.5921
rs199470	**MCH**	T	C	0.0185586	0.00223724	7.40E-20	68.81193262	0.1492	0.1325	0.2601
rs1998422	**MCH**	C	G	0.0266758	0.00196181	3.60E-41	184.8925879	0.0182	0.0185	0.3269
rs2036916	**MCH**	T	A	-0.012525	0.0019275	9.70E-11	42.22456454	0.016	0.0177	0.3672
rs205648	**MCH**	A	G	0.0124752	0.00256245	4.20E-08	23.70187578	0.0023	0.0222	0.9176
rs2067663	**MCH**	T	C	0.0274069	0.00226034	2.60E-37	147.0180889	-0.0178	0.0206	0.3885
rs2071243	**MCH**	T	C	-0.0164096	0.00187519	1.00E-20	76.57798186	-0.0233	0.016	0.1435
rs2072813	**MCH**	T	C	-0.0470991	0.00201928	5.90E-131	544.0397257	0.0019	0.0172	0.9103
rs2075672	**MCH**	G	A	0.0837776	0.00192536	1.00E-200	1893.347905	-0.0151	0.0209	0.4696
rs2134814	**MCH**	G	C	0.0180215	0.00189541	1.80E-23	90.40116368	-0.1486	0.0367	5.24E-05
rs2137283	**MCH**	A	C	0.049095	0.00189897	1.70E-156	668.4005597	0.0743	0.0469	0.1135
rs2140875	**MCH**	G	A	-0.0175449	0.00242024	3.50E-13	52.55129341	0.0197	0.018	0.2733
rs2153876	**MCH**	C	T	0.0315622	0.00347436	4.10E-19	82.52459202	0.0079	0.0187	0.6723
rs2160348	**MCH**	C	T	-0.023778	0.00211866	1.10E-32	125.9582432	-0.041	0.0271	0.13
rs2163951	**MCH**	C	T	-0.0236524	0.00261982	2.50E-21	81.509083	0.0242	0.0434	0.5781
rs218265	**MCH**	C	T	0.119696	0.00260846	1.00E-200	2105.66557	0.0423	0.0181	0.0191699
rs2236659	**MCH**	G	A	-0.0419829	0.00470094	1.40E-20	79.75794148	-0.043	0.0218	0.0483805
rs2236947	**MCH**	A	C	0.0104546	0.00186022	1.40E-08	31.58527826	-0.0014	0.0181	0.9369
rs2240460	**MCH**	T	C	-0.0117173	0.00181138	5.60E-11	41.84412069	-0.0012	0.0159	0.939
rs2241738	**MCH**	G	C	-0.0131554	0.00191838	4.20E-12	47.02592242	0.0378	0.0164	0.0210999
rs2253823	**MCH**	T	C	0.0161564	0.00218088	8.40E-15	54.88126814	0.0034	0.0164	0.8361
rs2288789	**MCH**	A	G	0.0183014	0.00198241	8.10E-23	85.22757873	-0.0221	0.0175	0.2059
rs2294450	**MCH**	C	T	-0.0210749	0.00215667	7.00E-23	95.49093254	0.0428	0.0169	0.0113
rs2302976	**MCH**	G	A	0.0254247	0.0023167	1.10E-29	120.4399484	0.028	0.0207	0.1775
rs2312491	**MCH**	G	A	-0.0105763	0.00191946	4.00E-09	30.36042689	0.0279	0.0168	0.0967609
rs2316124	**MCH**	A	G	-0.0142482	0.00195825	7.00E-15	52.93979053	-0.1099	0.1156	0.3415
rs2328945	**MCH**	C	T	0.0518482	0.00314556	5.00E-64	271.6876542	0.0277	0.0224	0.2168
rs233701	**MCH**	C	G	-0.0123498	0.00198059	1.10E-14	38.88026074	-0.0338	0.1173	0.772999
rs2351959	**MCH**	C	T	-0.0187875	0.00303821	2.20E-10	38.23850402	0.052	0.0871	0.5506
rs235887	**MCH**	C	T	0.0167593	0.00242449	3.50E-13	47.78256285	0.0115	0.0264	0.662599
rs2366541	**MCH**	C	T	0.0273638	0.00398997	4.00E-12	47.03401337	0.0015	0.0205	0.9414
rs2384322	**MCH**	G	C	-0.0285254	0.00231084	1.20E-38	152.3779817	-5.00E-04	0.0255	0.9859
rs2461848	**MCH**	A	G	-0.0243397	0.00351699	7.80E-13	47.89461195	-0.0913	0.1114	0.4123

rs2468024	**MCH**	A	G	0.0418637	0.00373361	1.30E-32	125.7233083	-0.015	0.0208	0.4711
rs2492301	**MCH**	C	T	-0.0196977	0.00186878	1.70E-27	111.0997716	-0.0062	0.0185	0.738401
rs2501369	**MCH**	A	G	-0.0138777	0.00188939	6.70E-14	53.9498509	-0.0064	0.0163	0.6934
rs2537594	**MCH**	A	G	0.0239235	0.00187397	8.50E-38	162.975623	-0.0221	0.0186	0.2334
rs2541639	**MCH**	A	G	0.0685349	0.00242615	6.00E-191	797.9703492	0.0117	0.0201	0.559899
rs2556097	**MCH**	T	C	0.0178645	0.00186132	2.90E-23	92.11664816	0.0199	0.0158	0.2089
rs257677	**MCH**	G	C	0.0105705	0.00187952	3.50E-09	31.62974146	-0.0103	0.0166	0.5325
rs259286	**MCH**	T	C	-0.0256297	0.00203051	9.20E-39	159.3218218	0.0118	0.0175	0.4977
rs2612696	**MCH**	A	G	-0.0150563	0.00190365	1.90E-14	62.55482257	-0.1656	0.113	0.1429
rs2661794	**MCH**	A	C	-0.0178926	0.00189836	1.20E-19	88.83584574	0.0027	0.0221	0.9037
rs2672092	**MCH**	C	T	-0.0192034	0.00223337	7.10E-22	73.93215816	0.0171	0.0169	0.3105
rs2692685	**MCH**	G	A	0.012799	0.00197341	4.80E-10	42.0645176	-0.0191	0.0174	0.2703
rs2713934	**MCH**	T	C	-0.0146054	0.00195093	9.30E-16	56.04566425	-1.00E-04	0.0226	0.9953
rs2734420	**MCH**	T	C	-0.0241398	0.00273638	1.00E-18	77.82390234	0.2318	0.1626	0.154
rs2748364	**MCH**	C	G	-0.0127284	0.00182228	1.40E-12	48.78832365	-0.0239	0.018	0.1845
rs2748427	**MCH**	G	A	-0.0336381	0.00226382	3.50E-57	220.7890921	0.0322	0.02	0.1073
rs2762497	**MCH**	T	C	0.022262	0.00390871	8.60E-09	32.43843854	0.0238	0.0373	0.5229
rs2777774	**MCH**	A	C	0.0107445	0.00204428	1.10E-08	27.62422767	-0.0189	0.0294	0.52
rs2791586	**MCH**	A	G	0.0128433	0.00244826	1.70E-08	27.51925445	0.0877	0.0628	0.1624
rs2811972	**MCH**	C	T	-0.0290367	0.00186683	1.40E-58	241.9265076	9.00E-04	0.0504	0.9862
rs282605	**MCH**	C	G	-0.0168246	0.00264684	3.20E-11	40.40476646	0.0147	0.0231	0.525
rs2834259	**MCH**	T	C	-0.0402031	0.0019815	5.90E-94	411.6512275	0.0303	0.0162	0.0619298
rs2834712	**MCH**	C	T	-0.0153723	0.00195175	1.70E-16	62.03371786	0.0243	0.016	0.1273
rs2836422	**MCH**	A	T	-0.0170984	0.00193616	1.80E-20	77.98784899	0.0237	0.0191	0.215
rs28498859	**MCH**	G	A	-0.0728374	0.00219345	1.00E-200	1102.686037	0.0207	0.0174	0.2327
rs2857078	**MCH**	C	A	-0.0288473	0.00205964	1.40E-49	196.1671085	-8.00E-04	0.0172	0.9643
rs28601761	**MCH**	G	C	-0.0224606	0.00191826	3.70E-33	137.0964626	0.0162	0.0203	0.4252
rs28621035	**MCH**	G	T	0.0179267	0.00245937	1.60E-12	53.13142243	-0.0785	0.068	0.2487
rs28638301	**MCH**	A	G	-0.023238	0.00346651	2.60E-12	44.93772309	-0.0177	0.0183	0.3345
rs2869045	**MCH**	C	T	0.0164435	0.00231312	1.20E-11	50.53481518	-0.018	0.0172	0.2955
rs28791905	**MCH**	A	G	-0.0155814	0.00229473	6.10E-11	46.10502379	-0.0169	0.0177	0.3393
rs2903578	**MCH**	C	T	-0.0180621	0.00193365	9.40E-23	87.25277207	-0.0056	0.0166	0.737199
rs2972558	**MCH**	T	C	0.0129984	0.00198916	9.00E-10	42.70107846	0.0483	0.0187	0.00980189
rs3004316	**MCH**	A	T	0.0111807	0.0020129	4.10E-08	30.85262165	-0.0084	0.0197	0.67
rs3173805	**MCH**	C	T	0.0119955	0.00188494	2.00E-10	40.49861141	-0.0029	0.0161	0.8549
rs322873	**MCH**	G	T	-0.019114	0.00186784	3.40E-27	104.7182326	-0.019	0.0187	0.3091
rs33983467	**MCH**	A	G	0.0113662	0.00207217	1.20E-09	30.08695991	-0.0265	0.0214	0.2146
rs34164109	**MCH**	T	C	0.180962	0.00206703	1.00E-200	7664.427121	0.016	0.0166	0.3365
rs34282584	**MCH**	C	A	-0.0132808	0.00203135	3.60E-12	42.74422429	-0.0018	0.0199	0.9278
rs34904684	**MCH**	G	A	0.0124058	0.00213271	1.50E-08	33.83642132	-0.0522	0.054	0.334
rs34931195	**MCH**	A	T	-0.033351	0.00201235	1.20E-64	274.6686977	-0.0104	0.016	0.516
rs35150201	**MCH**	G	T	0.0163885	0.00187287	1.20E-18	76.57052565	-0.0227	0.0168	0.176
rs35158985	**MCH**	G	A	-0.0262648	0.00202255	1.60E-43	168.6351668	-0.0048	0.0207	0.8169
rs35362007	**MCH**	A	G	0.0269759	0.00218413	6.90E-39	152.5433928	-0.0072	0.0189	0.702901

rs35388001	**MCH**	T	C	-0.0172536	0.00208299	6.50E-17	68.609391	-0.0106	0.0437	0.8081
rs35555052	**MCH**	C	G	0.0111569	0.00213718	1.40E-08	27.25231261	0.0398	0.0596	0.5048
rs35585881	**MCH**	A	G	-0.0244263	0.00336815	4.30E-16	52.59337103	0.0191	0.0259	0.4604
rs35602605	**MCH**	T	G	0.0293469	0.002409	1.10E-35	148.4052785	-0.0855	0.0617	0.1658
rs35673167	**MCH**	A	G	-0.0156014	0.00203316	2.80E-15	58.88199465	-0.0013	0.0204	0.9473
rs35936358	**MCH**	T	G	-0.0118747	0.00195797	4.50E-10	36.78169258	-0.0061	0.0185	0.739701
rs35998524	**MCH**	T	C	0.0796329	0.00319365	4.30E-148	621.7401438	0.0162	0.0708	0.8193
rs3730590	**MCH**	T	C	-0.0113761	0.00190428	1.90E-10	35.68812024	-0.0118	0.0174	0.4976
rs3740685	**MCH**	T	C	0.0177854	0.0020687	1.00E-19	73.91468476	-0.0031	0.016	0.847
rs3743879	**MCH**	G	C	0.0544251	0.0031046	3.20E-71	307.3163376	-0.0158	0.0179	0.3748
rs3756703	**MCH**	A	G	-0.0140058	0.00229335	6.20E-10	37.29697807	0.0109	0.0169	0.5189
rs3758641	**MCH**	A	G	0.0271774	0.00188395	8.10E-54	208.1016634	0.033	0.0165	0.0450796
rs3760137	**MCH**	C	T	0.0136376	0.00189303	8.20E-15	51.89904746	-0.0171	0.0179	0.337
rs3768321	**MCH**	T	G	-0.0334029	0.00234012	8.50E-51	203.7466955	-0.0294	0.0221	0.1843
rs3781058	**MCH**	C	T	0.0132569	0.00188443	9.50E-13	49.49056652	0.0347	0.0167	0.0373801
rs3807544	**MCH**	A	G	-0.0112755	0.00187876	1.30E-08	36.01865504	-0.0137	0.0158	0.3853
rs3809627	**MCH**	A	C	-0.0309755	0.00190435	8.70E-66	264.5706072	1.00E-04	0.0166	0.9961
rs3811444	**MCH**	T	C	-0.0304623	0.00196536	6.20E-59	240.2368583	-0.0028	0.0198	0.8881
rs3825762	**MCH**	C	T	-0.0198464	0.00204452	3.90E-23	94.22784039	0.0022	0.0206	0.9139
rs3891167	**MCH**	G	A	0.0148285	0.00223663	4.10E-12	43.95463067	-0.036	0.0281	0.2006
rs3909258	**MCH**	G	C	0.0415874	0.00187512	1.20E-116	491.8853533	0.0061	0.019	0.748001
rs4134058	**MCH**	C	T	0.030845	0.00186928	1.30E-65	272.2822397	-0.1129	0.0486	0.02031
rs4149056	**MCH**	C	T	-0.0254123	0.00260371	2.40E-26	95.25794516	0.0199	0.0219	0.365
rs42042	**MCH**	T	C	0.0164958	0.00186485	4.30E-19	78.24515206	0.0093	0.0202	0.6459
rs4332427	**MCH**	G	A	-0.0180688	0.00218721	4.50E-17	68.2458331	0.0171	0.0292	0.559
rs4355757	**MCH**	T	C	0.0178713	0.00201184	1.20E-19	78.90851983	0.043	0.0229	0.0605494
rs4484022	**MCH**	A	G	0.0130391	0.00214667	3.50E-10	36.89462851	-0.0508	0.0478	0.287
rs4554318	**MCH**	T	C	0.0412206	0.0018426	3.10E-119	500.4549257	-0.006	0.0196	0.758901
rs45553335	**MCH**	C	T	-0.0253003	0.00251517	2.20E-27	101.1847672	0.0324	0.0257	0.2072
rs4672884	**MCH**	A	G	0.0104384	0.00189556	3.90E-08	30.32433477	0.0105	0.0166	0.5262
rs4680338	**MCH**	G	C	-0.0114431	0.00190859	3.40E-11	35.9468287	0.0193	0.0169	0.2521
rs4696326	**MCH**	G	T	0.0108857	0.00201198	6.70E-09	29.27277455	-0.0172	0.0161	0.2857
rs4709820	**MCH**	C	T	-0.0326224	0.00184423	3.40E-73	312.8960899	-0.02	0.016	0.2134
rs4805993	**MCH**	T	A	-0.0115689	0.00192888	2.10E-09	35.97263059	0.0199	0.0211	0.3459
rs4815606	**MCH**	A	T	0.01938	0.00189289	1.50E-24	104.8226839	0.0168	0.0169	0.3189
rs4846072	**MCH**	C	T	-0.0122645	0.00189577	1.10E-11	41.8530282	0.0027	0.0163	0.8665
rs4887030	**MCH**	G	A	0.0114952	0.00188114	3.50E-11	37.34128946	-0.0031	0.0181	0.8652
rs496321	**MCH**	C	T	-0.0211208	0.00191847	1.70E-31	121.2018328	0.0051	0.0177	0.7743
rs4970485	**MCH**	T	C	0.0194667	0.00230367	6.10E-19	71.40730171	0.0194	0.0169	0.251
rs500422	**MCH**	A	C	-0.0129756	0.00187862	1.10E-15	47.70627405	-0.0149	0.0188	0.4292
rs507403	**MCH**	A	G	-0.0141365	0.00219771	7.20E-12	41.37533377	-0.0099	0.0162	0.542099
rs552565	**MCH**	C	T	0.0259027	0.00204629	8.60E-37	160.2338218	0.0022	0.016	0.889
rs55641552	**MCH**	A	G	0.0227894	0.0021286	3.70E-27	114.6241559	0.0343	0.0172	0.0458501
rs55648810	**MCH**	C	T	0.0356774	0.00295217	5.80E-39	146.0501992	-0.0845	0.059	0.1522

rs55776024	**MCH**	C	T	-0.020968	0.00281386	4.40E-13	55.5274242	0.0798	0.0426	0.0611097
rs55941903	**MCH**	C	A	0.0270557	0.00190327	5.00E-49	202.076229	-0.0385	0.0259	0.1371
rs56094641	**MCH**	G	A	-0.0127111	0.00190151	2.10E-12	44.68559316	-0.0206	0.0192	0.2832
rs56129102	**MCH**	T	C	-0.0223148	0.00210528	4.20E-29	112.3478464	0.0535	0.0218	0.01426
rs56241813	**MCH**	A	G	0.0263183	0.00404344	5.50E-12	42.36548122	0.0581	0.041	0.1565
rs56264440	**MCH**	C	A	0.0678469	0.0100079	2.10E-11	45.95921345	-0.1222	0.2334	0.6006
rs56273049	**MCH**	T	A	-0.0441348	0.00191685	3.20E-126	530.1326483	0.0152	0.0311	0.6262
rs56356382	**MCH**	C	T	-0.0470922	0.00237726	1.30E-95	392.4127209	-0.1184	0.0639	0.0638602
rs56397034	**MCH**	C	G	0.0736177	0.00190752	1.00E-200	1489.446037	-0.0037	0.023	0.8727
rs56687322	**MCH**	A	G	0.0327115	0.0039077	1.40E-16	70.0740103	0.0109	0.0298	0.7142
rs57103278	**MCH**	C	G	0.0391829	0.00375994	5.20E-27	108.5999952	-0.0326	0.0424	0.4418
rs57199709	**MCH**	G	A	-0.0418477	0.00586433	5.70E-14	50.92193155	0.1318	0.14	0.3465
rs57201028	**MCH**	T	C	0.0304198	0.00354916	1.40E-18	73.4615413	2.00E-04	0.0179	0.9919
rs5750673	**MCH**	A	G	-0.016073	0.00208042	7.60E-16	59.68845434	0.0088	0.0167	0.5999
rs5756527	**MCH**	G	T	-0.0312568	0.00186369	9.90E-67	281.2808473	-0.0111	0.0167	0.5047
rs58111256	**MCH**	T	C	0.0304458	0.00373684	8.50E-18	66.38107143	-0.0161	0.0249	0.518601
rs58123204	**MCH**	G	A	-0.0332297	0.00259485	4.20E-37	163.9935659	0.0111	0.0194	0.5681
rs58141407	**MCH**	T	C	0.0506976	0.00255958	1.00E-95	392.3158629	0.0638	0.0679	0.3479
rs58422016	**MCH**	A	G	-0.0231376	0.00209248	1.40E-29	122.2679047	-0.0501	0.0239	0.03596
rs595982	**MCH**	C	T	0.0157468	0.0019818	9.70E-16	63.13402221	0.0019	0.0169	0.9095
rs5995288	**MCH**	C	T	-0.0265503	0.00201593	1.30E-42	173.4548523	0.0027	0.0195	0.8907
rs600619	**MCH**	G	A	0.0150467	0.0020709	3.90E-14	52.79134802	0.1376	0.0515	0.00753009
rs6014993	**MCH**	G	A	0.0600645	0.0018824	1.00E-200	1018.14677	-0.0164	0.016	0.3067
rs6031307	**MCH**	A	T	0.0191378	0.00206036	8.30E-23	86.27724869	-0.0347	0.0181	0.0552701
rs6060987	**MCH**	A	T	0.0323984	0.0022818	3.60E-45	201.5999677	-0.0912	0.0521	0.0802195
rs60697121	**MCH**	C	G	-0.0300242	0.0031607	4.80E-23	90.23495782	0.0052	0.0337	0.8773
rs6073958	**MCH**	C	T	0.0241845	0.00234994	2.70E-29	105.9154126	0.0348	0.0249	0.1619
rs60757417	**MCH**	G	C	-0.0562205	0.00398112	2.10E-49	199.4239685	0.0642	0.0841	0.4456
rs60776348	**MCH**	C	T	0.0222133	0.00238789	1.70E-23	86.53584008	-0.0066	0.0608	0.9136
rs60833263	**MCH**	A	G	0.0172369	0.00188746	4.60E-21	83.39908669	0.0203	0.0184	0.2716
rs6084653	**MCH**	G	C	0.0233325	0.0019333	7.10E-33	145.6540386	-0.0469	0.0488	0.3363
rs6090040	**MCH**	C	A	0.0109607	0.00188692	1.70E-10	33.74178785	0.0234	0.017	0.1677
rs6093254	**MCH**	C	G	0.0137744	0.00191974	1.70E-13	51.48242989	0.0075	0.0163	0.6462
rs61134960	**MCH**	G	A	0.0126449	0.00224803	3.60E-09	31.63916963	-2.00E-04	0.0182	0.9931
rs61302130	**MCH**	C	G	0.0117937	0.00216189	2.20E-08	29.75989887	-0.0111	0.0176	0.5286
rs61421071	**MCH**	C	G	0.0109345	0.00192708	2.40E-08	32.19562448	-0.0285	0.0181	0.1148
rs61823972	**MCH**	C	A	0.041243	0.00188409	6.90E-118	479.1766987	0.0329	0.0232	0.1558
rs61828723	**MCH**	G	A	-0.0319662	0.00361787	5.70E-20	78.06827686	-0.1522	0.1185	0.1989
rs61874823	**MCH**	T	C	-0.0365106	0.0069104	2.60E-08	27.91451267	-0.1708	0.3094	0.581
rs61952071	**MCH**	A	C	0.0221795	0.00201959	6.90E-30	120.6078466	0.0243	0.0179	0.1733
rs61980623	**MCH**	T	C	-0.0254936	0.00410495	2.10E-10	38.56959471	0.1433	0.1276	0.2616
rs62005623	**MCH**	T	C	0.0204164	0.0021748	2.30E-24	88.12886295	0.0047	0.0187	0.8022
rs62162286	**MCH**	C	A	-0.0177161	0.0018955	2.60E-22	87.35487615	0.0164	0.0182	0.3676
rs62398471	**MCH**	A	G	-0.0132072	0.00212224	1.50E-09	38.72852894	-0.0019	0.0537	0.9725

rs62401093	**MCH**	A	T	-0.0415849	0.00316544	2.50E-41	172.5844388	-0.001	0.0817	0.9904
rs62410539	**MCH**	T	C	-0.0645803	0.00275067	1.40E-127	551.2157501	-0.0338	0.0174	0.0522504
rs638051	**MCH**	A	G	-0.013815	0.0019607	5.70E-12	49.64528002	0.001	0.0196	0.9586
rs644205	**MCH**	A	G	0.0189141	0.00236249	2.00E-17	64.09584652	-0.0038	0.0217	0.8594
rs6458869	**MCH**	A	C	0.016394	0.00189439	2.50E-18	74.89098803	0.0118	0.0226	0.600999
rs6460528	**MCH**	C	T	0.0135802	0.00187656	5.80E-12	52.37040181	0.063	0.1112	0.5709
rs646179	**MCH**	G	A	-0.0174698	0.00191728	1.00E-18	83.02392594	-0.0158	0.0165	0.3382
rs6494533	**MCH**	T	C	-0.0330057	0.00214273	1.10E-55	237.2692619	-0.0221	0.0286	0.439
rs6504074	**MCH**	T	G	-0.0186107	0.00213339	1.10E-18	76.09977813	0.0191	0.0188	0.308
rs6512645	**MCH**	A	G	-0.0206458	0.00191697	2.90E-28	115.992867	0.0166	0.0179	0.3531
rs6545005	**MCH**	T	C	-0.0134776	0.00204074	1.70E-11	43.61624316	0.0148	0.0193	0.4431
rs6549120	**MCH**	G	A	-0.0206575	0.00198967	2.50E-25	107.7933336	0.0203	0.0173	0.24
rs6573193	**MCH**	C	T	-0.0207089	0.00197352	1.40E-26	110.1106894	-0.0016	0.0165	0.9246
rs6578125	**MCH**	T	C	-0.0340581	0.0031845	2.60E-26	114.3817693	-0.0201	0.0709	0.7772
rs6580224	**MCH**	G	A	-0.0136039	0.00192414	1.10E-12	49.98641718	0.0089	0.0163	0.584501
rs6592965	**MCH**	A	G	0.0520561	0.00187948	2.30E-184	767.1253289	0.013	0.0171	0.4473
rs66824612	**MCH**	T	C	-0.0332144	0.00197726	1.40E-69	282.1783863	-0.1069	0.1165	0.3589
rs6692219	**MCH**	C	G	-0.0317114	0.00552523	1.10E-08	32.94037909	0.0083	0.0721	0.9085
rs6711700	**MCH**	A	G	0.0189128	0.00198418	2.80E-25	90.85482752	0.0253	0.0192	0.188
rs6718798	**MCH**	C	T	0.013298	0.00189866	1.70E-12	49.05426117	-0.0115	0.0216	0.5946
rs6730558	**MCH**	T	C	-0.022451	0.00192277	6.30E-35	136.3374568	-0.0128	0.0164	0.4374
rs6734238	**MCH**	G	A	-0.0181847	0.00189532	1.10E-21	92.05463797	0.0303	0.0302	0.3162
rs676800	**MCH**	A	C	-0.0171312	0.00196684	2.40E-18	75.86404416	0.0043	0.0168	0.796
rs6781977	**MCH**	A	G	0.0102155	0.00195255	1.40E-08	27.37243368	-0.0068	0.016	0.6724
rs6782400	**MCH**	A	C	-0.0582804	0.00188786	1.00E-200	953.0242729	0.0194	0.0159	0.2215
rs6802828	**MCH**	C	T	0.0125125	0.00196547	9.20E-11	40.52787436	-0.0029	0.0166	0.8592
rs6807097	**MCH**	G	A	0.0121103	0.00189014	6.80E-12	41.05067866	0.147	0.1121	0.1899
rs6810965	**MCH**	A	G	-0.0179266	0.00191107	2.40E-22	87.99158687	-0.0262	0.018	0.1467
rs68149176	**MCH**	T	C	0.0321339	0.00220282	4.70E-49	212.7978999	-0.0436	0.0177	0.0137899
rs6819291	**MCH**	C	T	0.03536	0.00309638	1.70E-32	130.4110793	0.2293	0.085	0.00696899
rs6844176	**MCH**	C	T	-0.0246438	0.00188542	6.00E-42	170.8431678	-0.0227	0.0161	0.1584
rs6857303	**MCH**	A	G	0.0228356	0.00188747	2.40E-35	146.3737579	-0.0119	0.016	0.4582
rs6867325	**MCH**	G	A	0.012519	0.00222794	1.00E-08	31.57408553	-0.0548	0.0315	0.0822602
rs6945202	**MCH**	C	T	-0.0106429	0.0018928	1.00E-09	31.61615065	-6.00E-04	0.0159	0.9709
rs6961761	**MCH**	A	C	0.0131797	0.00189649	4.50E-14	48.29569491	-0.0082	0.0274	0.764499
rs697248	**MCH**	C	G	-0.0149065	0.0020827	2.50E-12	51.22670458	0.0212	0.0172	0.2191
rs6987558	**MCH**	C	G	-0.0259687	0.0029959	5.20E-19	75.13534352	0.0193	0.0192	0.315
rs6992286	**MCH**	A	G	-0.0108514	0.00199446	5.30E-09	29.60188502	-0.018	0.0165	0.275
rs699579	**MCH**	T	C	0.0111043	0.00187814	3.00E-09	34.95625768	-0.0049	0.0205	0.8105
rs7024675	**MCH**	C	G	0.0160663	0.0021458	2.20E-15	56.05982608	0.0338	0.0214	0.1142
rs706027	**MCH**	T	A	0.0145427	0.00192144	1.00E-18	57.28420882	0.0118	0.0198	0.5516
rs7117878	**MCH**	A	C	-0.0220539	0.00201021	5.50E-30	120.3611774	-0.0162	0.0252	0.521601
rs7121112	**MCH**	C	T	-0.0260003	0.00190063	6.80E-47	187.1371632	-0.0093	0.0165	0.5733
rs7137095	**MCH**	A	C	0.0139009	0.00190483	1.10E-16	53.25640876	-0.0052	0.0169	0.757

rs71494799	**MCH**	C	T	0.0921998	0.00369022	2.40E-147	624.2433509	-0.0171	0.0249	0.4919
rs7156476	**MCH**	C	T	-0.0109919	0.00188075	2.80E-09	34.15716992	-0.0164	0.0202	0.4167
rs718515	**MCH**	A	G	-0.0593391	0.00188829	1.00E-200	987.5134227	0.0418	0.0343	0.2226
rs720483	**MCH**	C	T	0.018986	0.00201477	1.90E-24	88.80031078	-0.027	0.0168	0.1085
rs720783	**MCH**	G	C	0.0130548	0.00188854	1.80E-12	47.78444584	-0.0046	0.0189	0.8095
rs7208859	**MCH**	C	T	0.0376631	0.00266439	1.60E-50	199.8181899	0.0245	0.0212	0.248
rs72631343	**MCH**	G	C	-0.0149856	0.00279128	1.90E-09	28.82305088	-0.0018	0.0167	0.9126
rs7266642	**MCH**	G	A	0.024394	0.00194716	1.40E-39	156.9499725	-0.0385	0.0269	0.1523
rs72778450	**MCH**	A	G	0.013591	0.00254682	7.20E-09	28.4776931	-0.1144	0.0687	0.0955807
rs72796106	**MCH**	C	A	-0.0317354	0.00249272	1.40E-35	162.0837368	0.1005	0.048	0.0362301
rs72815555	**MCH**	A	G	-0.0448378	0.00368367	2.90E-36	148.158182	0.2551	0.1003	0.0109701
rs72829144	**MCH**	A	C	-0.0720549	0.00522631	5.00E-44	190.0794604	0.1062	0.1229	0.3873
rs72839066	**MCH**	G	A	0.090156	0.00487773	1.30E-80	341.6270414	0.1418	0.1346	0.2922
rs72888792	**MCH**	A	G	0.0184965	0.0026911	1.20E-12	47.24087411	0.0204	0.0369	0.5801
rs72939141	**MCH**	A	G	0.0378397	0.00611749	2.90E-10	38.26020712	-0.3701	0.2005	0.06485
rs72980278	**MCH**	T	G	-0.0674123	0.00413053	2.80E-62	266.3576729	0.1335	0.0981	0.1734
rs72990620	**MCH**	A	G	-0.0380983	0.00316775	1.50E-37	144.6464832	0.0227	0.0997	0.8196
rs73066228	**MCH**	G	A	-0.013682	0.00253986	4.50E-08	29.01870978	-0.0096	0.0336	0.776
rs7309114	**MCH**	A	G	-0.0159671	0.00243122	2.30E-11	43.13224414	-0.0255	0.0165	0.1212
rs73171144	**MCH**	A	G	0.0124779	0.00187471	5.80E-11	44.30097591	0.0063	0.0197	0.7493
rs73187263	**MCH**	T	C	0.0206122	0.00312699	2.40E-12	43.45044159	-0.0973	0.0743	0.19
rs732520	**MCH**	G	A	0.0308957	0.00204727	7.40E-55	227.7426227	0.0029	0.0271	0.9148
rs73369896	**MCH**	A	G	-0.0326637	0.00361863	6.70E-21	81.4780978	-0.0336	0.0514	0.5132
rs73382361	**MCH**	C	T	0.092077	0.0081172	2.00E-30	128.6732624	-0.2329	0.3555	0.5125
rs73560587	**MCH**	T	A	0.0143306	0.00257133	3.40E-08	31.06072569	0.0154	0.028	0.5815
rs73591936	**MCH**	A	G	-0.0374044	0.00425473	3.00E-22	77.28582694	0.0462	0.1336	0.7296
rs73660574	**MCH**	A	G	-0.0521166	0.00493948	5.20E-27	111.3238383	0.1473	0.107	0.1687
rs741951	**MCH**	A	G	0.036674	0.00263614	1.30E-45	193.5432828	0.022	0.0573	0.7009
rs74800925	**MCH**	A	T	-0.0747215	0.00937516	2.40E-16	63.52318576	-0.1642	0.0472	0.000507703
rs748716	**MCH**	A	G	-0.0114625	0.00195052	5.90E-09	34.5347539	0.0204	0.0179	0.2533
rs7498555	**MCH**	C	T	0.0150994	0.00190749	1.10E-17	62.66042331	-0.0259	0.0213	0.225
rs750714	**MCH**	A	G	0.0107925	0.00189086	1.20E-09	32.57795725	0.0135	0.0177	0.4462
rs75113100	**MCH**	A	G	0.041599	0.00783187	1.30E-09	28.21196427	-0.1105	0.1758	0.5295
rs7521458	**MCH**	C	T	0.0173706	0.00188743	1.30E-22	84.70058615	-0.0102	0.0162	0.5296
rs754205	**MCH**	C	A	0.0227874	0.00193816	1.10E-35	138.2320608	-0.015	0.0159	0.3454
rs7607996	**MCH**	C	T	-0.0116786	0.0018621	1.30E-09	39.33453778	0.0074	0.0232	0.750601
rs76177150	**MCH**	A	G	-0.0290672	0.00287068	8.60E-27	102.526307	0.0052	0.0202	0.7976
rs76201019	**MCH**	T	C	0.0421792	0.0058405	2.80E-14	52.15489843	0.0734	0.1927	0.7032
rs76234559	**MCH**	G	A	0.0390617	0.00334959	1.40E-33	135.9932841	-0.1208	0.0771	0.1169
rs762679	**MCH**	A	T	0.0185618	0.00265328	1.90E-13	48.94096122	-0.0077	0.0656	0.9071
rs7633686	**MCH**	C	G	0.0184314	0.00266325	6.70E-12	47.89511948	-0.0037	0.0164	0.8196
rs7641133	**MCH**	C	T	0.0118456	0.0020972	1.50E-08	31.903102	-0.0055	0.0218	0.8014
rs77055095	**MCH**	A	G	0.0331981	0.0022307	4.40E-54	221.4841267	0.0101	0.0169	0.5501
rs7705526	**MCH**	A	C	-0.0425713	0.00201976	3.60E-104	444.2554605	-0.0086	0.0172	0.6186

rs77115810	**MCH**	A	G	-0.0423222	0.00775802	1.60E-09	29.76001628	0.2745	0.2258	0.2242
rs7721678	**MCH**	C	T	0.0128656	0.00209841	2.60E-11	37.59048065	2.00E-04	0.0222	0.9941
rs77234976	**MCH**	G	C	-0.0178342	0.00266209	1.20E-11	44.88076812	-0.0577	0.0287	0.0442099
rs774211	**MCH**	C	T	0.0169544	0.00250683	1.60E-12	45.74183334	0.0055	0.0238	0.8176
rs7750077	**MCH**	C	T	0.00916959	0.00183918	4.30E-08	24.85706498	0.0055	0.0163	0.7349
rs77542162	**MCH**	G	A	0.0793691	0.00629774	7.00E-42	158.8297825	-0.3167	0.2464	0.1986
rs77651270	**MCH**	G	T	-0.0252067	0.00487243	4.40E-08	26.76327471	-0.0385	0.1061	0.7165
rs7765828	**MCH**	G	C	-0.0506026	0.00187442	2.70E-172	728.8032964	0.0167	0.0231	0.4709
rs7777063	**MCH**	T	G	-0.0479227	0.00298009	4.30E-56	258.5962844	-0.0128	0.0319	0.6893
rs7789162	**MCH**	C	T	0.0286476	0.00186398	2.30E-58	236.2068432	-0.0032	0.0197	0.8705
rs7793934	**MCH**	A	G	0.0105203	0.00188879	5.80E-10	31.02325647	-0.0016	0.0158	0.9209
rs78024727	**MCH**	A	G	0.116585	0.00543662	1.10E-105	459.8602604	0.222	0.325	0.494399
rs78378222	**MCH**	G	T	-0.0969038	0.00866894	1.80E-29	124.9534079	-0.2682	0.1933	0.1652
rs78406129	**MCH**	G	A	0.0400218	0.00652184	2.50E-09	37.65749597	0.1601	0.1451	0.2696
rs7867137	**MCH**	C	T	0.0148169	0.00191284	1.80E-15	60.0006415	-0.0188	0.0163	0.248
rs78744187	**MCH**	T	C	-0.0953115	0.00341225	4.10E-190	780.2026262	-0.0448	0.0496	0.3664
rs79287178	**MCH**	A	G	-0.0338274	0.00561488	1.50E-09	36.29566546	0.2156	0.1247	0.0838205
rs79780963	**MCH**	T	C	0.0247242	0.00349709	4.00E-14	49.98381049	-0.004	0.0189	0.8303
rs79785827	**MCH**	T	C	-0.0411067	0.00639445	2.50E-10	41.32542409	-0.2498	0.3936	0.5257
rs8013143	**MCH**	G	A	-0.029602	0.00208613	1.80E-50	201.3528844	0.017	0.034	0.6161
rs8018823	**MCH**	A	G	0.0249715	0.00207311	1.70E-35	145.0918612	0.0458	0.1223	0.7083
rs8032553	**MCH**	G	A	0.0106444	0.00187942	8.70E-09	32.07695746	-0.0244	0.0182	0.1817
rs8062719	**MCH**	G	A	-0.0242908	0.00194852	1.10E-39	155.4076469	-0.011	0.025	0.660601
rs806704	**MCH**	A	C	-0.0159658	0.00195268	2.10E-16	66.85250681	-0.0347	0.0226	0.1245
rs806970	**MCH**	T	C	-0.0741328	0.00383632	7.20E-90	373.4131771	-0.0496	0.0788	0.5293
rs854775	**MCH**	T	C	0.0132478	0.00192476	8.20E-13	47.37321295	-0.0028	0.0239	0.9052
rs855791	**MCH**	G	A	0.151668	0.00188394	1.00E-200	6481.152929	2.00E-04	0.0159	0.9918
rs869784	**MCH**	C	T	0.0576779	0.00197895	1.50E-195	849.4693631	0.0026	0.019	0.8912
rs875741	**MCH**	A	G	-0.0269803	0.0019031	5.60E-48	200.9873916	-0.015	0.0176	0.3951
rs8887	**MCH**	C	T	0.0707948	0.00190254	1.00E-200	1384.629593	-0.0105	0.017	0.5354
rs896850	**MCH**	G	C	-0.0108042	0.00200465	1.00E-08	29.04735531	-0.0201	0.0181	0.2673
rs900444	**MCH**	C	T	0.0135786	0.00236624	3.20E-09	32.9299363	0.0497	0.027	0.0654696
rs911000	**MCH**	C	G	0.0168308	0.00205147	1.20E-16	67.30970104	-0.0056	0.0169	0.7428
rs920112	**MCH**	A	G	0.0359834	0.00412238	1.20E-17	76.19155409	0.0083	0.0171	0.626599
rs9289629	**MCH**	G	A	-0.0299678	0.00197862	2.60E-56	229.3947202	0.0446	0.0303	0.1409
rs9319415	**MCH**	A	G	-0.0185899	0.00262668	4.70E-14	50.08852656	0.1853	0.0603	0.00213201
rs9349205	**MCH**	A	G	-0.107492	0.00208725	1.00E-200	2652.172889	0.0202	0.02	0.3125
rs9392465	**MCH**	A	C	-0.0124719	0.0019478	7.20E-11	40.99916396	-0.008	0.0159	0.612901
rs9429742	**MCH**	T	C	0.0217119	0.00214313	4.80E-28	102.6353842	-0.0787	0.0429	0.0664492
rs9457800	**MCH**	T	C	-0.0172229	0.0026576	1.50E-10	41.99830987	-0.0302	0.0591	0.609
rs9464759	**MCH**	C	T	-0.0469979	0.0034065	7.30E-45	190.343742	0.028	0.0766	0.7142
rs9487023	**MCH**	G	A	0.0783197	0.00182207	1.00E-200	1847.610404	-0.0184	0.0241	0.4457
rs9521020	**MCH**	A	C	-0.0168536	0.00194949	1.40E-19	74.73806071	0.0277	0.0159	0.0824005
rs9532563	**MCH**	C	T	-0.0265838	0.00227535	1.00E-33	136.5011106	0.0022	0.0296	0.94

rs9549317	**MCH**	G	A	0.0116799	0.00189222	6.90E-11	38.10074571	0.0044	0.0167	0.7931
rs9579583	**MCH**	C	T	0.0612603	0.00725306	1.10E-17	71.33689167	-0.2757	0.1719	0.1086
rs9811850	**MCH**	A	T	-0.0537065	0.00800502	9.30E-11	45.01189972	0.049	0.2697	0.8557
rs9832466	**MCH**	G	A	-0.0238901	0.00247686	3.60E-23	93.03181629	0.083	0.0474	0.0802694
rs9833388	**MCH**	C	G	-0.0294783	0.00224779	3.10E-41	171.9855196	-0.0935	0.1413	0.5079
rs9833415	**MCH**	A	G	-0.0164084	0.00254504	2.50E-12	41.56633449	0.0221	0.0171	0.1975
rs9856990	**MCH**	C	T	0.0243316	0.00256057	4.50E-22	90.29558467	-0.0425	0.0286	0.1373
rs9859077	**MCH**	C	G	0.0163159	0.00196878	5.40E-18	68.67934992	0.0301	0.0176	0.0869
rs9872347	**MCH**	C	T	0.0695887	0.00200065	1.00E-200	1209.856031	0.0054	0.0169	0.7504
rs9896673	**MCH**	G	C	-0.0129929	0.00195245	1.60E-11	44.28440702	0.0176	0.0168	0.2971
rs989978	**MCH**	G	A	0.0269532	0.00213686	4.20E-39	159.0988397	-0.0112	0.016	0.485
rs9963625	**MCH**	C	G	-0.0106168	0.00193544	1.90E-08	30.09028708	-8.00E-04	0.0162	0.9598
rs9972293	**MCH**	C	T	-0.0105284	0.00192862	1.30E-08	29.80093598	0.0118	0.0159	0.457999
rs9995319	**MCH**	C	G	0.0319015	0.00191045	3.20E-67	278.8363184	0.0221	0.0159	0.1665

MCH: Mean corpuscular hemoglobin. EA: effect allele. OA: other allele. GX: beta-exposure. GX(SE): standard error of GX. GY: beta-outcome. GY(SE): standard error of GY.

Table S12. Published associations of BMI on PTB

SNP	Exposure	EA	OA	GX	GX(SE)	Pval-exp	F	GY	GY(SE)	Pval-outcome
rs10063055	**BMI**	T	C	0.0136775	0.00226991	1.70E-09	36.30732798	-0.0651	0.0206	0.00157899
rs10099330	**BMI**	G	A	0.0124247	0.00198907	4.20E-10	39.01843138	0.0198	0.0159	0.2119
rs10160769	**BMI**	C	G	-0.0155862	0.00242199	1.20E-10	41.41273524	-0.0424	0.0197	0.0318302
rs10169594	**BMI**	C	T	0.0121875	0.00205506	3.00E-09	35.17048545	-0.0065	0.0246	0.792399
rs10182416	**BMI**	G	A	0.0130378	0.00197134	3.70E-11	43.74049351	-0.0265	0.0285	0.3528
rs10423928	**BMI**	A	T	-0.0340136	0.00249869	3.40E-42	185.3013402	-0.04	0.0188	0.0331002
rs10505836	**BMI**	C	A	0.0184851	0.00287086	1.20E-10	41.45889263	-0.0159	0.0166	0.3383
rs10510025	**BMI**	T	C	0.0175643	0.00229886	2.20E-14	58.37605777	-0.0232	0.016	0.1475
rs1064213	**BMI**	A	G	0.0149301	0.00197196	3.70E-14	57.32279372	-0.0011	0.0175	0.9481
rs10742752	**BMI**	C	T	0.0117979	0.00202961	6.10E-09	33.78954558	-0.0066	0.0162	0.683901
rs10756714	**BMI**	G	A	-0.0207862	0.00199408	1.90E-25	108.658365	5.00E-04	0.0162	0.9737
rs10756792	**BMI**	T	C	-0.0190582	0.00227201	4.90E-17	70.36252258	0.0078	0.0163	0.6317
rs10760277	**BMI**	T	C	0.0138734	0.00203825	1.00E-11	46.32858458	0.0048	0.0166	0.7716
rs10780248	**BMI**	A	G	-0.0121232	0.00199415	1.20E-09	36.95872767	0.026	0.0161	0.1069
rs1078141	**BMI**	T	C	0.0142239	0.00205862	4.90E-12	47.74007786	-0.0141	0.0221	0.5253
rs10799778	**BMI**	G	T	-0.0182237	0.00264876	6.00E-12	47.33539591	0.0189	0.1489	0.899
rs10809621	**BMI**	G	C	-0.0125305	0.00207043	1.40E-09	36.62805146	0.0195	0.0269	0.4678
rs10824211	**BMI**	T	C	0.0207688	0.00286631	4.30E-13	52.50185018	0.0493	0.0276	0.0739503
rs10832778	**BMI**	G	C	0.0115628	0.00203985	1.40E-08	32.13125432	-0.0151	0.0166	0.3649
rs10887578	**BMI**	C	G	0.0133212	0.00198909	2.10E-11	44.85139415	-0.0332	0.0168	0.0478597

rs10927006	**BMI**	C	T	-0.0168156	0.00281467	2.30E-09	35.69175354	-0.0443	0.0294	0.1315
rs10965698	**BMI**	T	C	-0.0112741	0.002052	3.90E-08	30.18611138	-0.0127	0.0222	0.565999
rs10989067	**BMI**	A	G	0.0169514	0.00212349	1.40E-15	63.72484994	-0.0166	0.0285	0.5605
rs11001963	**BMI**	T	C	0.0116794	0.00201921	7.30E-09	33.45616875	-0.0114	0.0165	0.4897
rs11009685	**BMI**	T	C	-0.0130834	0.00230766	1.40E-08	32.14368858	0.1131	0.0594	0.0568198
rs11012732	**BMI**	G	A	0.0216425	0.00210126	7.10E-25	106.0848541	-0.0939	0.0479	0.05
rs11079849	**BMI**	T	C	-0.020093	0.00211163	1.80E-21	90.54240777	-0.0138	0.0387	0.7224
rs11099020	**BMI**	T	C	-0.0142038	0.00206202	5.60E-12	47.44838863	-0.02	0.0188	0.2861
rs11115160	**BMI**	A	G	-0.0130768	0.00233641	2.20E-08	31.32585751	0.0215	0.0202	0.2861
rs11122450	**BMI**	G	T	-0.0116316	0.00202425	9.10E-09	33.01784614	0.0134	0.0175	0.4446
rs11134679	**BMI**	G	A	0.0182459	0.00213286	1.20E-17	73.18195369	0.0045	0.0252	0.8589
rs11150745	**BMI**	G	A	-0.0211611	0.00212961	2.90E-23	98.73575283	0.0052	0.0169	0.7586
rs111598585	**BMI**	T	C	-0.0142245	0.00243798	5.40E-09	34.04174858	0.0523	0.069	0.4485
rs11165643	**BMI**	T	C	0.0193319	0.00200337	4.90E-22	93.11611877	-0.0142	0.0177	0.4218
rs111689389	**BMI**	C	G	-0.0136706	0.00219522	4.70E-10	38.78083613	-0.03	0.0266	0.2595
rs11218510	**BMI**	A	G	-0.0144616	0.00202121	8.40E-13	51.19268769	0.014	0.0178	0.4339
rs11250094	**BMI**	C	G	-0.0202907	0.00199209	2.30E-24	103.7466941	-0.0113	0.0297	0.702999
rs1126930	**BMI**	C	G	0.0322695	0.00535892	1.70E-09	36.26002029	0.0564	0.1355	0.6772
rs113079574	**BMI**	T	C	-0.0155896	0.00251598	5.80E-10	38.39314535	0.018	0.0172	0.2955
rs113603865	**BMI**	T	C	0.0186013	0.00242689	1.80E-14	58.74684226	0.0097	0.0212	0.6461
rs113624107	**BMI**	A	G	0.0150461	0.00236876	2.10E-10	40.34631585	0.0585	0.0534	0.2737
rs11525873	**BMI**	C	T	-0.0239781	0.00333603	6.60E-13	51.66158863	0.0246	0.0169	0.1461
rs11607476	**BMI**	C	A	0.015712	0.00199179	3.10E-15	62.22629784	-0.0044	0.0212	0.8355
rs11610621	**BMI**	A	T	0.0164869	0.00278318	3.10E-09	35.0908215	-0.0325	0.0638	0.610299
rs11630647	**BMI**	A	G	-0.012674	0.00228082	2.70E-08	30.87759725	0.0269	0.0194	0.1652
rs11634851	**BMI**	G	C	0.0118671	0.00198456	2.20E-09	35.75681722	-0.0096	0.0279	0.7314
rs116374395	**BMI**	A	G	0.0318817	0.00536236	2.80E-09	35.34834831	-0.0293	0.1356	0.8288
rs11642090	**BMI**	C	T	0.0114184	0.00205925	2.90E-08	30.74613105	-0.0174	0.0198	0.3773
rs11656076	**BMI**	A	G	-0.0154285	0.00237054	7.60E-11	42.35951233	-0.0253	0.0166	0.1286
rs1167311	**BMI**	A	G	-0.0192649	0.00213109	1.60E-19	81.71994269	0.0164	0.0165	0.3204
rs11675464	**BMI**	G	A	0.0119751	0.00198558	1.60E-09	36.37321049	0.0061	0.0179	0.731601
rs11691869	**BMI**	A	C	-0.0193158	0.00205562	5.60E-21	88.29535332	-0.0021	0.0159	0.8942
rs11699828	**BMI**	A	G	-0.0335418	0.00582677	8.60E-09	33.13714566	-0.2601	0.1807	0.1502
rs11709402	**BMI**	G	A	0.0228192	0.00220796	4.90E-25	106.8111386	-7.00E-04	0.0217	0.9725
rs117118217	**BMI**	C	G	0.0448186	0.00788004	1.30E-08	32.34877553	0.0903	0.1802	0.6161
rs117342986	**BMI**	T	C	0.0365794	0.00646781	1.60E-08	31.98576676	0.1741	0.2067	0.3997
rs11757278	**BMI**	C	T	-0.0146269	0.00214834	9.90E-12	46.35500934	0.0171	0.0158	0.2804
rs11778219	**BMI**	G	A	0.015796	0.0026874	4.20E-09	34.54838433	-0.094	0.064	0.1421
rs118136827	**BMI**	T	G	-0.0132582	0.0022019	1.70E-09	36.25534707	0.0027	0.0227	0.9041
rs11919665	**BMI**	T	A	-0.0127854	0.00211292	1.40E-09	36.61513581	-0.031	0.0187	0.0973106
rs12001437	**BMI**	C	T	0.0121836	0.00205069	2.80E-09	35.29793954	-0.0169	0.016	0.2912
rs12072739	**BMI**	G	A	0.0157008	0.00236691	3.30E-11	44.00258512	-0.0059	0.0352	0.8662
rs12088284	**BMI**	T	C	0.0139317	0.00214854	8.90E-11	42.04549392	0.0157	0.0184	0.393
rs12089815	**BMI**	A	G	-0.0123121	0.00198606	5.70E-10	38.43064353	0.0052	0.0164	0.7485

rs12140153	**BMI**	T	G	-0.033075	0.0034589	1.20E-21	91.43696676	-0.1114	0.0888	0.2097
rs12149660	**BMI**	A	G	-0.0227338	0.00311586	3.00E-13	53.23365869	0.0016	0.0248	0.9477
rs12259464	**BMI**	A	G	0.0130895	0.00198686	4.50E-11	43.4019957	-0.0343	0.0312	0.2726
rs12273545	**BMI**	T	C	0.0248393	0.00428551	6.80E-09	33.5947797	-0.0232	0.0259	0.3703
rs1229984	**BMI**	C	T	0.037357	0.00599385	4.60E-10	38.84457392	-0.0166	0.0205	0.4184
rs12364470	**BMI**	G	T	0.0192705	0.0026658	4.90E-13	52.25513293	0.01	0.0585	0.8647
rs12440603	**BMI**	T	C	0.0139285	0.0020011	3.40E-12	48.44726121	1.00E-04	0.0171	0.9973
rs12459368	**BMI**	G	A	-0.0170141	0.00223235	2.50E-14	58.08867983	0.028	0.0165	0.0894499
rs12462975	**BMI**	A	G	0.0195822	0.00212071	2.60E-20	85.26258842	0.0142	0.0187	0.4464
rs12507026	**BMI**	T	A	0.0289896	0.0019931	6.30E-48	211.5555316	-0.0162	0.017	0.3408
rs12541408	**BMI**	C	T	-0.0143261	0.00212723	1.60E-11	45.3550005	-0.0093	0.0158	0.5563
rs1266874	**BMI**	G	A	0.0140966	0.0020712	1.00E-11	46.32151936	-0.0274	0.0194	0.1592
rs12681792	**BMI**	A	C	0.0148693	0.00251693	3.50E-09	34.90092125	-0.0164	0.0174	0.3434
rs12692596	**BMI**	T	C	0.0130872	0.00203738	1.30E-10	41.26173822	0.0358	0.0167	0.0324198
rs12696039	**BMI**	G	A	-0.0152802	0.0027705	3.50E-08	30.41864652	0.0328	0.0295	0.2664
rs1286058	**BMI**	A	T	0.0149126	0.00216922	6.20E-12	47.26042859	0.0378	0.027	0.1606
rs12881629	**BMI**	G	A	0.0220748	0.00358929	7.70E-10	37.8246201	0.0387	0.0906	0.6693
rs12921986	**BMI**	G	A	0.0203327	0.0036983	3.80E-08	30.22622762	-4.00E-04	0.1077	0.9972
rs12937411	**BMI**	T	C	-0.017187	0.00201383	1.40E-17	72.8371022	0.0103	0.0161	0.5199
rs1296328	**BMI**	C	A	-0.018862	0.001999	3.90E-21	89.03238564	-0.0083	0.0309	0.788301
rs12974458	**BMI**	T	C	0.0152466	0.00199717	2.30E-14	58.27926465	0.0556	0.0486	0.2526
rs13012070	**BMI**	A	G	-0.0136501	0.00234795	6.10E-09	33.7980851	-0.0061	0.0195	0.7535
rs13033310	**BMI**	A	G	0.012611	0.0022826	3.30E-08	30.52372578	0.0077	0.0171	0.6528
rs13097918	**BMI**	A	T	0.0145561	0.0024161	1.70E-09	36.29596672	0.0246	0.0288	0.393
rs13107325	**BMI**	T	C	0.0475799	0.00375479	8.50E-37	160.5734958	0.0932	0.1558	0.5499
rs13176429	**BMI**	C	T	0.0141555	0.00213122	3.10E-11	44.11557862	-0.0235	0.0189	0.2144
rs1320251	**BMI**	T	C	-0.0180321	0.00199407	1.50E-19	81.7730003	0.0044	0.0166	0.7913
rs13218383	**BMI**	G	C	-0.0144025	0.00209236	5.80E-12	47.38065581	0.0318	0.0161	0.0483404
rs1322842	**BMI**	G	A	-0.0131292	0.00203539	1.10E-10	41.6082426	-0.0066	0.0164	0.6879
rs13248187	**BMI**	C	T	0.015764	0.00224155	2.00E-12	49.45772196	1.00E-04	0.0202	0.9945
rs1327259	**BMI**	G	A	-0.0148532	0.00203363	2.80E-13	53.34507078	-0.0475	0.018	0.00816996
rs13291723	**BMI**	A	G	0.0110318	0.00199876	3.40E-08	30.46278309	-0.0011	0.0189	0.9538
rs1330199	**BMI**	T	G	-0.0117509	0.00198642	3.30E-09	34.99437329	0.0108	0.0288	0.7085
rs13420048	**BMI**	A	C	-0.0154817	0.00205272	4.60E-14	56.88214766	0.0058	0.0282	0.8373
rs13427822	**BMI**	G	A	-0.0181515	0.00224142	5.60E-16	65.58079426	0.0121	0.0343	0.7251
rs1346841	**BMI**	A	G	-0.013056	0.0020172	9.70E-11	41.89097626	-0.0046	0.0161	0.774901
rs1360201	**BMI**	T	C	0.0130075	0.00197747	4.80E-11	43.26791618	0.0042	0.0157	0.788
rs13642	**BMI**	T	A	-0.0161363	0.00205695	4.30E-15	61.54015229	0.0198	0.0166	0.2332
rs140159717	**BMI**	T	C	-0.0246882	0.00370967	2.80E-11	44.29007501	-0.0649	0.0796	0.4146
rs1438945	**BMI**	A	T	-0.0133824	0.00219791	1.10E-09	37.07202603	-0.0263	0.0354	0.4569
rs1441264	**BMI**	A	G	0.0179033	0.00205854	3.40E-18	75.63898201	0.0191	0.0159	0.2274
rs1451963	**BMI**	T	G	0.022201	0.00360585	7.40E-10	37.9077387	-0.0287	0.1218	0.814
rs1454687	**BMI**	G	C	-0.0207916	0.00197396	6.10E-26	110.9423203	-0.0118	0.0172	0.4921
rs1458156	**BMI**	T	C	0.014075	0.00197951	1.20E-12	50.55679386	-0.0058	0.016	0.7183

rs145981104	**BMI**	G	A	0.022711	0.00404552	2.00E-08	31.51533495	-0.1248	0.1162	0.2827
rs146569428	**BMI**	A	G	0.0139533	0.00248571	2.00E-08	31.51019294	0.0722	0.0367	0.04923
rs1471093	**BMI**	A	G	0.0134619	0.00203996	4.10E-11	43.54793184	0.0053	0.0204	0.7958
rs1471740	**BMI**	C	T	0.0193576	0.00225404	8.90E-18	73.75269648	-0.0257	0.1262	0.8388
rs147568678	**BMI**	C	T	-0.0133327	0.00233034	1.10E-08	32.73374933	0.0094	0.0274	0.7312
rs1477290	**BMI**	C	T	0.0337772	0.00289815	2.20E-31	135.8324986	0.01	0.0166	0.549
rs147730268	**BMI**	T	G	-0.0350799	0.00358357	1.30E-22	95.82592643	-0.0059	0.0867	0.9456
rs1503526	**BMI**	C	T	0.0154308	0.00197681	5.90E-15	60.93195936	-0.0027	0.0212	0.9006
rs156201	**BMI**	C	G	0.0131899	0.00228932	8.30E-09	33.19465191	-0.014	0.0165	0.3988
rs156914	**BMI**	A	G	0.0111552	0.00197308	1.60E-08	31.96417142	-0.0815	0.0485	0.0931901
rs1582931	**BMI**	A	G	-0.0133419	0.0019956	2.30E-11	44.69783517	-0.0016	0.016	0.9214
rs1608113	**BMI**	T	A	-0.0117766	0.00205086	9.30E-09	32.97356747	0.0245	0.0158	0.1219
rs1609010	**BMI**	G	A	0.0209773	0.0019963	7.90E-26	110.4194762	-0.0064	0.0198	0.747599
rs16916303	**BMI**	G	A	-0.0192369	0.00307931	4.20E-10	39.02666745	-0.0015	0.0181	0.9323
rs17056301	**BMI**	C	T	0.0135831	0.0022697	2.20E-09	35.81450874	0.014	0.0176	0.4263
rs17132130	**BMI**	C	G	-0.0178418	0.00238564	7.50E-14	55.93268082	-0.0109	0.0236	0.643701
rs17149254	**BMI**	C	T	-0.0213507	0.00255804	7.00E-17	69.66388522	0.1245	0.1442	0.388
rs17289010	**BMI**	G	A	-0.0134661	0.00210438	1.60E-10	40.94807188	0.004	0.0169	0.8146
rs17399739	**BMI**	G	A	0.0270713	0.00391006	4.40E-12	47.93464305	0.0324	0.03	0.2805
rs17446299	**BMI**	G	C	0.0153235	0.00266515	8.90E-09	33.05755651	-0.0332	0.0471	0.4814
rs17544384	**BMI**	C	T	0.0140931	0.00241434	5.30E-09	34.07331007	0.0046	0.0611	0.9397
rs17668356	**BMI**	G	C	-0.0230543	0.00279318	1.50E-16	68.1246291	0.0104	0.0213	0.6255
rs17770336	**BMI**	T	C	0.0242931	0.00211161	1.30E-30	132.3538484	-0.024	0.0194	0.2163
rs1778830	**BMI**	A	G	0.0140775	0.00205596	7.50E-12	46.88348297	0.0158	0.0167	0.3423
rs1788808	**BMI**	G	A	-0.020404	0.00198255	7.70E-25	105.9206042	0.0231	0.0278	0.4062
rs1793636	**BMI**	C	G	-0.0133343	0.0021408	4.70E-10	38.79594806	0.0111	0.0262	0.6711
rs1805123	**BMI**	G	T	-0.0167569	0.00229711	3.00E-13	53.21350786	0.0149	0.0404	0.7121
rs1834144	**BMI**	A	C	-0.0140112	0.00205213	8.60E-12	46.6164335	-0.0094	0.0161	0.5616
rs1860750	**BMI**	A	T	0.0117038	0.00198192	3.50E-09	34.87222521	-0.0085	0.0199	0.67
rs1861410	**BMI**	T	C	-0.0212558	0.00198918	1.20E-26	114.1838967	0.0117	0.0162	0.4689
rs1884897	**BMI**	G	A	0.020001	0.00205634	2.30E-22	94.60447727	-0.0319	0.025	0.2022
rs1919243	**BMI**	C	T	0.0116759	0.00200175	5.50E-09	34.02194803	-0.0201	0.0172	0.2413
rs1967772	**BMI**	A	G	-0.0170434	0.00220377	1.00E-14	59.81058606	0.0191	0.0442	0.666
rs2035936	**BMI**	T	G	0.0370863	0.00435778	1.70E-17	72.42600482	0.0492	0.0181	0.00652094
rs2051559	**BMI**	C	T	0.0204078	0.00291963	2.80E-12	48.85790735	-0.0373	0.0203	0.0656599
rs2075466	**BMI**	C	G	0.0132903	0.00223627	2.80E-09	35.31987932	-0.0079	0.0173	0.649101
rs2102278	**BMI**	G	A	0.0118583	0.0021139	2.00E-08	31.46835977	0.0274	0.0162	0.0900306
rs2133561	**BMI**	T	A	-0.014097	0.00204744	5.80E-12	47.40554566	0.008	0.0204	0.6945
rs213518	**BMI**	C	T	0.0157894	0.00280491	1.80E-08	31.68775782	-0.0491	0.0618	0.4275
rs2153740	**BMI**	G	A	-0.0112551	0.00199313	1.60E-08	31.8878752	0.0503	0.0255	0.0489204
rs215634	**BMI**	G	A	-0.0155223	0.00203492	2.40E-14	58.18560882	-0.0122	0.0168	0.4669
rs2172131	**BMI**	C	T	-0.0149382	0.00200386	9.00E-14	55.57249619	-0.06	0.0281	0.03249
rs217672	**BMI**	C	A	0.0170155	0.00223046	2.40E-14	58.19674167	-0.0188	0.1242	0.8799
rs2192158	**BMI**	G	A	-0.015012	0.00198302	3.70E-14	57.30876381	0.0115	0.0163	0.4811

rs2216931	**BMI**	A	C	0.0169109	0.00208587	5.20E-16	65.72900904	-0.0547	0.0189	0.00376201
rs2234458	**BMI**	T	C	-0.0203841	0.00205579	3.60E-23	98.31588701	0.029	0.016	0.0690399
rs2248551	**BMI**	A	G	0.0146523	0.00266497	3.80E-08	30.22908996	-0.0043	0.0171	0.8001
rs2253310	**BMI**	G	C	0.0173213	0.00204115	2.10E-17	72.01272453	-0.0155	0.0176	0.3801
rs2271189	**BMI**	A	G	-0.0163086	0.00201869	6.50E-16	65.26678442	0.0141	0.0168	0.4004
rs2289379	**BMI**	T	C	-0.0152765	0.00202952	5.20E-14	56.65773067	0.0069	0.0207	0.7381
rs2307111	**BMI**	C	T	-0.0280042	0.00202203	1.30E-43	191.8091274	-0.0131	0.0159	0.4106
rs2342892	**BMI**	G	T	-0.0126972	0.00197715	1.30E-10	41.24153302	0.0222	0.0163	0.172
rs2381404	**BMI**	C	T	0.0139664	0.00229974	1.30E-09	36.88158628	0.0093	0.0208	0.655901
rs2383377	**BMI**	A	G	0.0161287	0.00293564	3.90E-08	30.18500502	-0.0278	0.0261	0.2866
rs2396625	**BMI**	A	T	-0.018919	0.00201335	5.60E-21	88.29902621	-0.0184	0.0165	0.2654
rs2398861	**BMI**	G	A	0.0179932	0.00226733	2.10E-15	62.97750681	-0.0063	0.0162	0.6948
rs2425816	**BMI**	A	G	0.0122284	0.00201106	1.20E-09	36.97322507	0.0041	0.0203	0.8384
rs2433733	**BMI**	A	G	-0.0171805	0.00210907	3.80E-16	66.3571503	0.0268	0.0187	0.151
rs2439823	**BMI**	G	A	0.0191998	0.00199108	5.30E-22	92.98525948	-0.0121	0.0173	0.4837
rs2482356	**BMI**	C	T	-0.0113511	0.00199506	1.30E-08	32.37144564	0.0189	0.0158	0.2339
rs2512892	**BMI**	C	T	0.0129468	0.00199819	9.20E-11	41.98067658	-0.0308	0.0164	0.0608401
rs252761	**BMI**	T	G	-0.0115046	0.00201769	1.20E-08	32.51114623	0.0022	0.0163	0.8931
rs2568958	**BMI**	A	G	0.0222928	0.00201204	1.60E-28	122.7592246	0.022	0.027	0.4138
rs2569993	**BMI**	C	T	0.01267	0.00212202	2.40E-09	35.64941454	-0.0273	0.0165	0.0985508
rs2606228	**BMI**	C	A	-0.0138791	0.00208379	2.70E-11	44.36217477	-0.0098	0.0376	0.7942
rs2616143	**BMI**	A	G	-0.0138738	0.00212547	6.70E-11	42.60680984	-0.0377	0.0389	0.3321
rs2618039	**BMI**	T	A	0.0143963	0.0020317	1.40E-12	50.20890303	0.0118	0.016	0.4598
rs2678204	**BMI**	G	T	0.024161	0.00208158	3.80E-31	134.7229908	-0.003	0.0245	0.9024
rs2725371	**BMI**	G	A	-0.0160301	0.00215845	1.10E-13	55.15521672	0.0089	0.0279	0.7487
rs2791643	**BMI**	T	C	-0.0133837	0.00231332	7.20E-09	33.47180086	-0.0029	0.0299	0.9227
rs28350	**BMI**	G	A	-0.0180335	0.00258234	2.90E-12	48.76759304	0.0347	0.0272	0.2028
rs28366156	**BMI**	C	T	-0.0264826	0.00292978	1.60E-19	81.70513267	-0.0107	0.0581	0.8543
rs2837996	**BMI**	C	T	0.0126647	0.00207859	1.10E-09	37.12361534	-0.0735	0.0262	0.00494903
rs28404639	**BMI**	T	C	-0.0117129	0.00205533	1.20E-08	32.4760997	-0.014	0.0189	0.4574
rs28489620	**BMI**	A	G	-0.0153737	0.00220025	2.80E-12	48.82147121	-0.0272	0.0525	0.6045
rs28568418	**BMI**	A	G	-0.0182825	0.00320812	1.20E-08	32.47641426	-0.0443	0.0474	0.3503
rs2861685	**BMI**	C	T	-0.0171274	0.00199763	1.00E-17	73.51075711	0.0051	0.0163	0.752801
rs28670671	**BMI**	C	T	-0.0124638	0.00226426	3.70E-08	30.30026814	-0.0036	0.0183	0.8443
rs2870111	**BMI**	T	C	-0.0157151	0.00201912	7.10E-15	60.57705477	-0.0104	0.016	0.516699
rs2875762	**BMI**	C	G	0.015339	0.0023121	3.30E-11	44.01279867	0.011	0.0189	0.5595
rs2899644	**BMI**	T	C	0.0149659	0.0023609	2.30E-10	40.18357866	-0.023	0.0163	0.1593
rs2920503	**BMI**	T	C	-0.0140307	0.00219666	1.70E-10	40.79727084	1.00E-04	0.0161	0.9955
rs2962334	**BMI**	T	G	0.0432552	0.00703547	7.80E-10	37.79971589	0.043	0.1714	0.8021
rs317656	**BMI**	A	T	-0.0144763	0.00221303	6.10E-11	42.78964313	-0.0065	0.0174	0.707499
rs3213943	**BMI**	A	C	-0.0179406	0.00288163	4.80E-10	38.76105693	0.0203	0.0163	0.2121
rs32421	**BMI**	T	A	0.0132315	0.00237757	2.60E-08	30.9706026	0.0151	0.0182	0.4066
rs329118	**BMI**	T	C	-0.0165734	0.0020038	1.30E-16	68.4088985	0.0181	0.0163	0.2645
rs329651	**BMI**	T	G	0.0157218	0.00250202	3.30E-10	39.48399592	0.0158	0.0772	0.8374

rs34045288	**BMI**	T	C	0.0234784	0.00209371	3.50E-29	125.7482813	-0.0051	0.0165	0.755099
rs34153025	**BMI**	C	T	-0.0389151	0.00678155	9.60E-09	32.92884388	-0.1772	0.1409	0.2087
rs34234296	**BMI**	A	G	-0.014947	0.00204032	2.40E-13	53.66728355	0.0218	0.0204	0.2854
rs34481751	**BMI**	A	C	-0.0185029	0.00270404	7.80E-12	46.82216571	0.008	0.0239	0.7377
rs34517439	**BMI**	A	C	0.038848	0.00304983	3.60E-37	162.2498051	-7.00E-04	0.073	0.9923
rs34696181	**BMI**	C	T	0.0114345	0.00198283	8.10E-09	33.25534925	0.0084	0.0164	0.6077
rs347551	**BMI**	G	C	0.013865	0.00201085	5.40E-12	47.5421168	0.0389	0.0401	0.3328
rs34811474	**BMI**	A	G	-0.0285293	0.00234269	4.10E-34	148.3032614	0.0093	0.0572	0.8703
rs349071	**BMI**	A	G	-0.0133062	0.00198231	1.90E-11	45.05708256	0.007	0.016	0.662
rs35154326	**BMI**	G	A	-0.0130427	0.00223442	5.30E-09	34.07246136	0.0017	0.0164	0.9194
rs35364449	**BMI**	T	C	0.0217069	0.00318335	9.20E-12	46.49700155	0.1148	0.0707	0.1042
rs355777	**BMI**	C	G	0.0152715	0.00201314	3.30E-14	57.54578973	0.0044	0.0158	0.778999
rs35697587	**BMI**	A	G	-0.0164677	0.00198067	9.20E-17	69.12573526	0.0165	0.0172	0.3396
rs35697691	**BMI**	G	C	0.0230305	0.00352223	6.20E-11	42.75327887	0.1253	0.1105	0.2568
rs35809007	**BMI**	A	G	-0.0171009	0.00205627	9.10E-17	69.16331068	0.0058	0.0159	0.715801
rs35957544	**BMI**	T	G	-0.0196429	0.00200438	1.10E-22	96.03934907	0.0188	0.019	0.3229
rs36007635	**BMI**	A	G	-0.021045	0.00286788	2.20E-13	53.84855173	0.0209	0.0733	0.7752
rs36061954	**BMI**	T	C	0.0128445	0.00201928	2.00E-10	40.4612631	0.0101	0.0171	0.5568
rs3764625	**BMI**	G	T	-0.0117714	0.00201523	5.20E-09	34.11969289	-0.0075	0.0179	0.676
rs3784710	**BMI**	C	T	-0.0297067	0.00236042	2.50E-36	158.3902218	0.0077	0.0168	0.645601
rs3803286	**BMI**	G	A	-0.0186417	0.00209828	6.40E-19	78.93002943	-0.0054	0.0159	0.7346
rs3807566	**BMI**	T	G	-0.0120727	0.00199583	1.50E-09	36.58978373	-0.0016	0.0163	0.9198
rs3814883	**BMI**	T	C	0.0240107	0.00198424	1.00E-33	146.4263916	0.0049	0.0179	0.782699
rs3845344	**BMI**	T	C	0.0163609	0.00201927	5.40E-16	65.64833442	0.015	0.0173	0.3863
rs3851998	**BMI**	G	C	-0.0136071	0.00226874	2.00E-09	35.97160488	-0.0179	0.0409	0.6625
rs3866805	**BMI**	A	C	0.0117835	0.00206546	1.20E-08	32.54716401	-0.0388	0.0191	0.0416802
rs3897102	**BMI**	T	C	0.0120838	0.00202716	2.50E-09	35.53277442	0.006	0.017	0.724001
rs3901286	**BMI**	A	C	-0.0225553	0.00275602	2.70E-16	66.97775766	0.0239	0.0172	0.1654
rs3902951	**BMI**	G	T	0.0141019	0.00235267	2.00E-09	35.92786001	0.0132	0.0174	0.4465
rs3935190	**BMI**	A	G	-0.0144864	0.00199701	4.00E-13	52.62093785	-0.0092	0.0168	0.582801
rs394608	**BMI**	C	T	0.0186353	0.00199484	9.50E-21	87.26794696	-0.0024	0.0158	0.8801
rs396755	**BMI**	G	C	-0.0123639	0.00199937	6.30E-10	38.24042784	-0.0241	0.0165	0.1423
rs40071	**BMI**	C	T	-0.0261666	0.00258163	3.80E-24	102.7317557	0.0534	0.0161	0.000912893
rs4017425	**BMI**	T	C	-0.0125817	0.00198035	2.10E-10	40.36387602	-0.0269	0.017	0.1142
rs4055791	**BMI**	T	C	-0.0178082	0.00200801	7.40E-19	78.65139396	-0.0177	0.0194	0.3601
rs406388	**BMI**	G	C	0.015973	0.00260181	8.30E-10	37.6894625	-0.0227	0.0596	0.703701
rs41279738	**BMI**	G	T	0.0684263	0.0062225	4.00E-28	120.9245337	-0.0841	0.1082	0.4371
rs4148155	**BMI**	G	A	-0.0229681	0.00310659	1.40E-13	54.66135316	-1.00E-04	0.0179	0.9954
rs4261944	**BMI**	G	T	0.0138553	0.00205652	1.60E-11	45.39041049	0.0053	0.0198	0.7895
rs4267103	**BMI**	C	T	0.0153916	0.00254312	1.40E-09	36.62958073	-0.0349	0.0166	0.03571
rs4284600	**BMI**	C	T	0.0119673	0.00199506	2.00E-09	35.98144094	-0.0279	0.0307	0.3635
rs429343	**BMI**	G	A	-0.0173795	0.00199729	3.30E-18	75.71648043	-0.0118	0.0199	0.5524
rs429358	**BMI**	C	T	-0.0266723	0.00274373	2.40E-22	94.50097919	-0.0081	0.0259	0.7548
rs4307239	**BMI**	G	A	0.0121413	0.00198816	1.00E-09	37.29287233	-0.0452	0.0194	0.0198399

rs4419475	**BMI**	T	A	0.011487	0.0020106	1.10E-08	32.64074055	-4.00E-04	0.017	0.9823
rs4444317	**BMI**	G	A	-0.0161604	0.00242104	2.50E-11	44.5552003	0.0668	0.0558	0.2315
rs4456769	**BMI**	T	C	0.0146111	0.00210138	3.60E-12	48.34535515	-0.0029	0.0163	0.8599
rs4477562	**BMI**	T	C	0.0296118	0.00298011	2.90E-23	98.73318411	-0.019	0.0192	0.324
rs4482463	**BMI**	A	C	-0.0313175	0.00370692	3.00E-17	71.37495754	0.0236	0.017	0.1659
rs45486197	**BMI**	A	G	0.025766	0.00403737	1.70E-10	40.7281815	-0.1453	0.1003	0.1476
rs4605363	**BMI**	C	A	0.0163638	0.00207733	3.30E-15	62.05195335	-0.0176	0.016	0.2732
rs4648450	**BMI**	A	C	-0.014837	0.00198775	8.40E-14	55.71431392	-0.0206	0.0163	0.207
rs4658403	**BMI**	T	C	-0.0188877	0.00264997	1.00E-12	50.80124492	0.0227	0.018	0.2062
rs4672338	**BMI**	T	C	0.0135646	0.00208669	8.00E-11	42.25676355	0.0448	0.0196	0.0223398
rs4722398	**BMI**	T	C	0.0186993	0.00287545	7.90E-11	42.28995039	0.011	0.0323	0.734401
rs4737188	**BMI**	T	A	-0.0124447	0.00198274	3.50E-10	39.39448658	-0.025	0.0205	0.2226
rs4764949	**BMI**	G	A	-0.0183924	0.00211197	3.10E-18	75.84019394	0.0091	0.0167	0.5847
rs4790292	**BMI**	A	C	-0.0254509	0.00275603	2.60E-20	85.27790557	-0.037	0.0242	0.1269
rs4820410	**BMI**	G	A	-0.0177457	0.00208527	1.70E-17	72.420213	0.0303	0.0158	0.0560299
rs4832298	**BMI**	T	C	-0.015965	0.00212268	5.40E-14	56.56748984	0.0758	0.052	0.1448
rs4858940	**BMI**	C	T	0.0229281	0.00309976	1.40E-13	54.71143587	0.0023	0.0501	0.9632
rs4876611	**BMI**	G	A	0.0197549	0.00220502	3.30E-19	80.26435554	-0.0042	0.0177	0.8135
rs4929923	**BMI**	C	T	0.0189424	0.00206436	4.50E-20	84.19712327	-0.0265	0.0163	0.1035
rs5011579	**BMI**	G	C	0.0140268	0.00219322	1.60E-10	40.90260159	0.0161	0.0164	0.3281
rs512121	**BMI**	C	T	-0.0159359	0.00252176	2.60E-10	39.93409125	0.0133	0.0173	0.4399
rs529200	**BMI**	G	A	0.0168965	0.0019785	1.30E-17	72.93223352	-0.0089	0.0163	0.5858
rs539515	**BMI**	C	A	0.0495291	0.0024426	2.00E-91	411.1632451	-0.032	0.0191	0.0943496
rs55707359	**BMI**	G	T	0.0531292	0.00812381	6.20E-11	42.77058158	-0.1207	0.3955	0.7601
rs55714539	**BMI**	C	A	0.0175719	0.00210043	6.00E-17	69.98728376	0.0366	0.0164	0.0259699
rs55726687	**BMI**	A	G	0.02483	0.00242628	1.40E-24	104.7296664	0.0066	0.0268	0.8066
rs55769038	**BMI**	A	G	0.0160961	0.00201096	1.20E-15	64.06673274	0.0251	0.0159	0.1128
rs558887	**BMI**	G	A	-0.0129976	0.00214881	1.50E-09	36.58713643	-0.0295	0.0282	0.2955
rs559231	**BMI**	T	G	0.0134907	0.00203562	3.40E-11	43.92114657	0.027	0.0164	0.0990992
rs56038322	**BMI**	A	G	0.0139276	0.00214833	9.00E-11	42.02896568	-0.0229	0.0177	0.1945
rs56094641	**BMI**	G	A	0.0734967	0.00201412	1.00E-200	1331.567275	-0.0206	0.0192	0.2832
rs56133507	**BMI**	G	T	0.0137622	0.00247385	2.70E-08	30.94761046	-0.0869	0.0529	0.1002
rs56143236	**BMI**	T	C	0.0126667	0.00226533	2.30E-08	31.26526647	-0.0019	0.0164	0.9094
rs56161855	**BMI**	T	A	0.0224575	0.00291784	1.40E-14	59.23767261	-0.004	0.018	0.8247
rs56203622	**BMI**	C	T	0.0179769	0.00280183	1.40E-10	41.16652049	0.01	0.0167	0.5488
rs56352336	**BMI**	C	T	-0.016327	0.00275021	2.90E-09	35.24351284	0.0509	0.0718	0.478
rs56399737	**BMI**	T	C	-0.0161325	0.00199646	6.40E-16	65.29504697	0.0022	0.0209	0.9168
rs56858768	**BMI**	A	G	0.0159188	0.00217378	2.40E-13	53.62750514	0.0017	0.0496	0.9725
rs56893062	**BMI**	G	T	0.0125141	0.00215383	6.20E-09	33.75782957	-0.0082	0.0181	0.6495
rs56930105	**BMI**	T	C	0.0157557	0.00286187	3.70E-08	30.30914737	-0.0072	0.0288	0.8021
rs57636386	**BMI**	C	T	-0.0412553	0.00358326	1.10E-30	132.5564833	0.027	0.0227	0.2338
rs57989773	**BMI**	C	T	0.0133488	0.00236315	1.60E-08	31.90803001	0.0543	0.0598	0.3641
rs58862095	**BMI**	T	C	-0.0229877	0.00200786	2.40E-30	131.0757355	-0.0365	0.0481	0.4484
rs59068084	**BMI**	T	G	0.0110552	0.00201048	3.80E-08	30.23652038	0.0063	0.0179	0.725

rs59086897	**BMI**	A	T	0.033385	0.00196986	2.00E-64	287.2302365	-0.0368	0.0158	0.0203198
rs59227842	**BMI**	G	A	0.0229675	0.00215313	1.50E-26	113.785013	-0.0099	0.0212	0.6399
rs594024	**BMI**	C	T	-0.0146771	0.00199081	1.70E-13	54.35243396	-0.0987	0.1113	0.375
rs6023655	**BMI**	G	A	-0.0147346	0.00234997	3.60E-10	39.31426699	0.0046	0.0315	0.8842
rs60764613	**BMI**	T	G	0.02099	0.00282688	1.10E-13	55.13257138	-0.0426	0.0582	0.4636
rs61740466	**BMI**	A	G	-0.0135153	0.00231885	5.60E-09	33.97067572	0.0392	0.0169	0.0202698
rs61813324	**BMI**	T	C	0.0290259	0.00292038	2.80E-23	98.7849481	0.0073	0.067	0.9127
rs61828641	**BMI**	A	G	0.0224585	0.00315913	1.20E-12	50.53876385	0.0243	0.02	0.2246
rs61871615	**BMI**	T	C	-0.0267159	0.00359269	1.00E-13	55.2965767	0.1243	0.088	0.1577
rs61903695	**BMI**	G	A	0.0166235	0.00227115	2.50E-13	53.57367222	-0.0339	0.0238	0.1537
rs61992671	**BMI**	G	A	-0.0161925	0.00206924	5.10E-15	61.23563124	0.086	0.0368	0.0193602
rs62007782	**BMI**	A	G	-0.0167007	0.00224215	9.40E-14	55.48021559	-0.2538	0.1254	0.0429497
rs62020775	**BMI**	A	T	-0.0165665	0.0028635	7.20E-09	33.47073678	-0.0022	0.0207	0.9171
rs62072006	**BMI**	C	A	0.0156492	0.00282374	3.00E-08	30.7137601	-0.2926	0.1577	0.0635507
rs62107261	**BMI**	C	T	-0.0911559	0.00460882	4.60E-87	391.190402	-0.0255	0.153	0.8679
rs62176243	**BMI**	T	A	-0.0149699	0.00228878	6.10E-11	42.77872304	0.0025	0.0174	0.8855
rs62190049	**BMI**	C	G	-0.0111798	0.00203614	4.00E-08	30.14747301	-0.0119	0.0261	0.6476
rs62241847	**BMI**	G	A	-0.0124263	0.00212897	5.30E-09	34.06769127	-0.0122	0.0244	0.616401
rs62246311	**BMI**	A	G	0.0207539	0.00325487	1.80E-10	40.65652562	0.0028	0.0931	0.9759
rs62379271	**BMI**	G	T	0.0117237	0.00200557	5.00E-09	34.17054155	0.0076	0.0164	0.6434
rs62407562	**BMI**	A	T	0.0144393	0.00222463	8.50E-11	42.12838571	-0.0282	0.0241	0.2418
rs6265	**BMI**	T	C	-0.0399185	0.00252719	3.30E-56	249.5001185	0.0082	0.0164	0.6188
rs6430068	**BMI**	A	G	0.0186066	0.0031882	5.30E-09	34.05971782	0.0861	0.0808	0.2864
rs6444950	**BMI**	A	G	0.0158525	0.00232007	8.30E-12	46.68651588	-0.0189	0.0187	0.3102
rs6545714	**BMI**	A	G	-0.0205219	0.00201573	2.40E-24	103.6498154	-0.0243	0.0171	0.1535
rs6560906	**BMI**	C	T	-0.012198	0.00214136	1.20E-08	32.448603	-0.0119	0.0201	0.5524
rs6561937	**BMI**	A	T	-0.015934	0.00230346	4.60E-12	47.85049043	-0.0196	0.024	0.4145
rs6567160	**BMI**	C	T	0.0541723	0.00234213	2.30E-118	534.9715339	-0.0363	0.0192	0.0582801
rs6575340	**BMI**	A	G	0.0207333	0.00206189	8.70E-24	101.1123017	-0.0419	0.0197	0.03322
rs6597975	**BMI**	G	C	0.013681	0.00199532	7.10E-12	47.01199617	-0.0149	0.0172	0.3877
rs66679256	**BMI**	T	C	0.0148864	0.00198759	6.90E-14	56.09496487	-0.0169	0.0171	0.3223
rs6669341	**BMI**	G	A	-0.0170287	0.0019986	1.60E-17	72.59543977	-0.036	0.0353	0.3071
rs6682438	**BMI**	C	T	0.0131586	0.00210047	3.70E-10	39.24501705	-0.019	0.0198	0.3354
rs6705567	**BMI**	C	T	-0.0146168	0.00204919	9.80E-13	50.87896536	-0.088	0.1146	0.4426
rs6707827	**BMI**	G	A	0.0119437	0.00217332	3.90E-08	30.20150165	0.0365	0.017	0.0320302
rs6710091	**BMI**	G	C	-0.0116835	0.00206707	1.60E-08	31.94726436	0.0106	0.019	0.5758
rs6713781	**BMI**	C	G	-0.0135736	0.00202799	2.20E-11	44.79779036	0.0348	0.0202	0.0858203
rs6725931	**BMI**	T	C	0.0191046	0.00274403	3.30E-12	48.47259894	0.0485	0.0351	0.1674
rs6744646	**BMI**	G	A	0.0554684	0.00261213	4.50E-100	450.9203352	0.0105	0.0258	0.683299
rs6752979	**BMI**	A	G	0.0125235	0.00211721	3.30E-09	34.9882068	0.0055	0.0198	0.782699
rs67609008	**BMI**	C	T	0.0170863	0.0022021	8.60E-15	60.20327245	-0.0759	0.0444	0.0872409
rs6769617	**BMI**	T	A	-0.0135754	0.00208902	8.10E-11	42.22971903	0.0243	0.0343	0.4786
rs6774894	**BMI**	A	T	0.0133088	0.00205674	9.70E-11	41.8713657	-0.0237	0.0213	0.2648
rs6777784	**BMI**	T	G	0.0116125	0.00202753	1.00E-08	32.80310801	-0.0363	0.016	0.0230802

rs6831088	**BMI**	A	G	-0.0115222	0.0020591	2.20E-08	31.31223439	9.00E-04	0.0235	0.9682
rs6843852	**BMI**	T	C	0.0130897	0.00197607	3.50E-11	43.87860928	0.0056	0.0173	0.7473
rs6909685	**BMI**	T	C	-0.014612	0.00211352	4.70E-12	47.79744929	-0.0057	0.0168	0.7341
rs6922607	**BMI**	G	A	0.0148848	0.00251479	3.20E-09	35.03326995	0.0111	0.0245	0.651199
rs6938973	**BMI**	C	T	0.0182223	0.00201872	1.80E-19	81.48024588	-0.021	0.0163	0.1982
rs6950388	**BMI**	A	G	0.0155219	0.00244806	2.30E-10	40.20163946	0.0287	0.0171	0.0929202
rs6962980	**BMI**	C	A	-0.0159568	0.00198814	1.00E-15	64.41630283	-0.0951	0.1114	0.3933
rs698147	**BMI**	G	A	-0.0128512	0.00198514	9.60E-11	41.90860472	-0.0125	0.0157	0.4258
rs7024334	**BMI**	G	T	-0.0138177	0.00238338	6.70E-09	33.61112547	0.0302	0.0162	0.0624396
rs7027304	**BMI**	T	C	0.0145492	0.00208703	3.10E-12	48.59806324	-0.013	0.016	0.4168
rs7034554	**BMI**	G	A	-0.0129826	0.00204252	2.10E-10	40.4006949	-0.0139	0.0196	0.4794
rs7038943	**BMI**	C	T	-0.0140202	0.00208641	1.80E-11	45.15514326	-0.0086	0.0159	0.5882
rs704061	**BMI**	C	T	0.0146374	0.00198552	1.70E-13	54.34723678	0.0062	0.0158	0.6934
rs7070670	**BMI**	T	C	-0.0123274	0.00211928	6.00E-09	33.83486242	-0.0076	0.0233	0.7438
rs7081254	**BMI**	C	T	-0.0142516	0.00245216	6.20E-09	33.77751994	-0.038	0.0527	0.4715
rs7124681	**BMI**	A	C	0.0256977	0.0020063	1.50E-37	164.0570436	-0.0147	0.0171	0.3891
rs7132908	**BMI**	A	G	0.0297904	0.00203363	1.40E-48	214.5887288	0.036	0.0173	0.0371099
rs71495038	**BMI**	A	G	0.0277998	0.00370969	6.70E-14	56.15730038	0.0017	0.0236	0.9437
rs7201895	**BMI**	A	G	-0.0149781	0.00207999	6.00E-13	51.85481334	0.0035	0.0192	0.8553
rs7206608	**BMI**	G	C	0.0135122	0.00211797	1.80E-10	40.70152099	-0.0327	0.0299	0.2734
rs7218014	**BMI**	C	T	0.0189524	0.00249216	2.90E-14	57.83286441	6.00E-04	0.0174	0.9722
rs7232171	**BMI**	T	G	0.0123404	0.00200821	8.00E-10	37.7605526	-0.0196	0.0415	0.6359
rs723672	**BMI**	T	C	0.0111273	0.00200698	3.00E-08	30.73913361	-6.00E-04	0.0205	0.9785
rs7250833	**BMI**	T	C	0.0135041	0.002188	6.70E-10	38.09208748	0.0049	0.0214	0.8199
rs7259070	**BMI**	C	T	0.021869	0.00203628	6.60E-27	115.3402731	0.101	0.0486	0.0378599
rs72634826	**BMI**	A	G	-0.021277	0.00228001	1.00E-20	87.08541009	-0.098	0.0593	0.0981296
rs72649373	**BMI**	C	T	0.0178062	0.00287805	6.10E-10	38.27753327	0.096	0.0665	0.1493
rs72673947	**BMI**	G	A	0.0218864	0.00321496	9.90E-12	46.34422611	-0.0324	0.0271	0.2321
rs72892910	**BMI**	T	G	0.0387798	0.00262053	1.50E-49	218.9933745	0.0029	0.0194	0.881
rs72976986	**BMI**	A	G	-0.0232257	0.00254712	7.60E-20	83.1451517	-0.119	0.0649	0.0664997
rs73026725	**BMI**	A	C	-0.022318	0.00275052	4.90E-16	65.8383669	-0.1374	0.1516	0.3646
rs73052033	**BMI**	C	T	-0.0303929	0.00254634	7.70E-33	142.46547	0.0147	0.0465	0.751201
rs7306534	**BMI**	A	G	-0.0112754	0.00205437	4.10E-08	30.12344962	-0.0118	0.0161	0.4624
rs73124396	**BMI**	C	T	-0.0154472	0.0024548	3.10E-10	39.59728666	-0.0192	0.0183	0.2937
rs73142879	**BMI**	T	C	-0.0266936	0.00252257	3.60E-26	111.9762609	0.0268	0.0229	0.2404
rs73193736	**BMI**	G	A	-0.0177165	0.00232002	2.20E-14	58.31368913	0.034	0.0551	0.5373
rs73213484	**BMI**	T	A	-0.0225766	0.0028364	1.70E-15	63.35490459	-0.0256	0.0288	0.3736
rs7331420	**BMI**	A	G	-0.0143702	0.00220081	6.60E-11	42.63425136	-0.005	0.0217	0.8195
rs7357754	**BMI**	G	A	0.0141146	0.00198362	1.10E-12	50.63121051	-0.0202	0.0167	0.2271
rs73601548	**BMI**	T	C	0.0177354	0.00311639	1.30E-08	32.38743015	-0.0158	0.0474	0.7391
rs73985439	**BMI**	C	A	0.0136616	0.00214059	1.70E-10	40.73186203	-0.0109	0.016	0.4933
rs7442885	**BMI**	G	C	-0.0228163	0.00241251	3.20E-21	89.44381412	0.0136	0.0614	0.8241
rs745249	**BMI**	T	C	0.0176118	0.00219724	1.10E-15	64.24666881	-0.0091	0.0161	0.5705
rs74750282	**BMI**	C	T	-0.0196382	0.00352561	2.50E-08	31.02651068	0.0706	0.0854	0.4085

rs7498665	**BMI**	G	A	0.0268646	0.00201997	2.30E-40	176.876052	-0.0244	0.0213	0.2513
rs7516554	**BMI**	T	C	0.0120066	0.00201565	2.60E-09	35.48198898	-0.0339	0.0159	0.0330697
rs7519259	**BMI**	A	G	0.0140028	0.00198381	1.70E-12	49.82275507	-0.0187	0.0205	0.3609
rs754635	**BMI**	G	C	0.0219925	0.0031093	1.50E-12	50.02902801	-0.0313	0.018	0.0811596
rs75499503	**BMI**	T	C	-0.0180331	0.00241845	8.90E-14	55.59870468	-0.0046	0.0308	0.8804
rs7568228	**BMI**	C	G	-0.0119625	0.00197288	1.30E-09	36.76551713	-0.015	0.0159	0.3474
rs7571496	**BMI**	G	A	-0.0158916	0.00225454	1.80E-12	49.68410581	0.0178	0.0165	0.2792
rs76183894	**BMI**	C	T	-0.0219584	0.00364437	1.70E-09	36.30400814	-0.0188	0.0806	0.8153
rs7619139	**BMI**	A	T	0.0134537	0.00201061	2.20E-11	44.77400256	0.0037	0.0161	0.8191
rs765874	**BMI**	A	T	-0.0120592	0.00197569	1.00E-09	37.25611026	0.0399	0.0159	0.0121099
rs76702514	**BMI**	G	C	-0.0164862	0.00243278	1.20E-11	45.92335117	-0.0137	0.0203	0.5015
rs7683836	**BMI**	A	G	-0.0122469	0.00199427	8.20E-10	37.71225908	-0.003	0.0167	0.8551
rs7704382	**BMI**	G	C	0.0120126	0.00199631	1.80E-09	36.20897118	0.0242	0.0181	0.182
rs7708584	**BMI**	G	A	-0.0159321	0.0019949	1.40E-15	63.78255385	0.041	0.0357	0.2508
rs7761673	**BMI**	A	T	-0.0135915	0.0023901	1.30E-08	32.33707641	-0.0237	0.0326	0.4662
rs7762794	**BMI**	G	A	0.0149077	0.00218606	9.10E-12	46.50452912	-0.0182	0.016	0.2553
rs7774	**BMI**	A	C	0.014988	0.00215169	3.30E-12	48.52059117	0.0356	0.0171	0.0376799
rs7776021	**BMI**	A	G	0.0123701	0.00218251	1.40E-08	32.12418026	0.0217	0.0194	0.2621
rs7802342	**BMI**	G	T	0.0122748	0.00218145	1.80E-08	31.6618612	-0.015	0.0182	0.4123
rs7805441	**BMI**	T	C	0.0133747	0.00198884	1.80E-11	45.22374509	0.0093	0.0158	0.5533
rs78086698	**BMI**	C	T	0.0318357	0.0050761	3.60E-10	39.3338617	-0.0603	0.0324	0.0630406
rs784257	**BMI**	C	T	0.0179315	0.00254968	2.00E-12	49.46067104	0.1182	0.0634	0.0623706
rs78605811	**BMI**	C	A	-0.032737	0.00444673	1.80E-13	54.19938438	0.2043	0.1147	0.0747808
rs7893571	**BMI**	T	G	0.0140553	0.00210152	2.30E-11	44.73127757	0.0115	0.0242	0.636
rs7924036	**BMI**	T	G	-0.0142836	0.0019778	5.10E-13	52.15653505	0.0198	0.0169	0.2428
rs7925100	**BMI**	A	G	0.014725	0.0020221	3.30E-13	53.0277825	0.0374	0.023	0.1032
rs7944782	**BMI**	G	T	0.0157606	0.00198748	2.20E-15	62.88369859	-0.0465	0.0161	0.00378704
rs7947143	**BMI**	A	G	-0.0182782	0.00267522	8.30E-12	46.6816247	0.0018	0.0263	0.9458
rs79780963	**BMI**	T	C	0.0236803	0.00369591	1.50E-10	41.0515674	-0.004	0.0189	0.8303
rs7996639	**BMI**	A	G	0.0145559	0.00200245	3.60E-13	52.8387923	0.0072	0.0158	0.648
rs80135274	**BMI**	T	A	0.021339	0.00388113	3.80E-08	30.2294235	0.0413	0.0882	0.639501
rs8015400	**BMI**	A	C	0.0213422	0.00211709	6.70E-24	101.6243655	-0.0123	0.0184	0.504001
rs8020365	**BMI**	A	T	0.0251068	0.00239517	1.00E-25	109.877345	-0.0366	0.0563	0.5163
rs8024137	**BMI**	T	A	0.0156329	0.00276778	1.60E-08	31.90171889	-0.1473	0.0878	0.0933297
rs8025516	**BMI**	G	T	-0.0146558	0.00207578	1.70E-12	49.84877965	0.0048	0.0173	0.7802
rs8076669	**BMI**	C	T	0.0140681	0.00199601	1.80E-12	49.67565311	-0.001	0.0204	0.9596
rs8089514	**BMI**	A	T	0.012988	0.00207588	3.90E-10	39.14517043	-0.0172	0.0213	0.4201
rs8112818	**BMI**	G	A	-0.020696	0.00202712	1.80E-24	104.2346307	-0.067	0.113	0.5536
rs8132491	**BMI**	A	G	-0.015375	0.00219317	2.40E-12	49.14550176	-0.0396	0.0183	0.0306803
rs815163	**BMI**	C	T	-0.016485	0.00198582	1.00E-16	68.91222303	-6.00E-04	0.0163	0.9717
rs852042	**BMI**	G	A	-0.0131207	0.00231309	1.40E-08	32.17563057	0.0158	0.0188	0.3986
rs862320	**BMI**	T	C	-0.0231703	0.00201345	1.20E-30	132.4279734	0.0158	0.0217	0.4682
rs879620	**BMI**	T	C	0.0241136	0.00203595	2.30E-32	140.2774965	-0.0161	0.0167	0.3358
rs909892	**BMI**	A	G	-0.0184105	0.00291255	2.60E-10	39.95604607	-0.0051	0.0201	0.7979

rs923994	**BMI**	G	A	-0.0145761	0.00240219	1.30E-09	36.81849961	0.0121	0.03	0.685499
rs9267671	**BMI**	A	G	0.026149	0.00413587	2.60E-10	39.97371659	-0.0712	0.1247	0.5677
rs9291822	**BMI**	T	C	-0.014263	0.00199464	8.60E-13	51.13177087	-0.1272	0.1116	0.2546
rs9294260	**BMI**	A	G	0.014782	0.00198818	1.00E-13	55.27810063	-0.0181	0.0192	0.3461
rs9349235	**BMI**	T	C	0.0111809	0.00200924	2.60E-08	30.96620704	-0.0014	0.0158	0.9277
rs935166	**BMI**	A	G	-0.0161066	0.00197292	3.20E-16	66.6479681	0.001	0.0192	0.9573
rs9388446	**BMI**	A	T	0.0111013	0.00197919	2.00E-08	31.46087665	0.001	0.0171	0.9542
rs9463175	**BMI**	T	C	-0.0115	0.00210115	4.40E-08	29.95571454	0.0286	0.0201	0.1553
rs9478496	**BMI**	C	T	0.0181836	0.00267493	1.10E-11	46.20968556	0.0011	0.0337	0.974
rs9515446	**BMI**	G	A	0.0151051	0.00199052	3.20E-14	57.58537988	0.0135	0.0161	0.4002
rs9522180	**BMI**	T	C	-0.0141095	0.00199325	1.50E-12	50.10693291	0.0037	0.0327	0.9099
rs9571687	**BMI**	A	C	-0.0135388	0.0021094	1.40E-10	41.19462747	-0.0131	0.0166	0.4293
rs961498	**BMI**	C	G	0.011981	0.00199336	1.80E-09	36.12540925	-0.015	0.016	0.3458
rs9638713	**BMI**	G	A	-0.0361735	0.00635399	1.20E-08	32.41052951	-0.017	0.2168	0.9375
rs9673839	**BMI**	G	A	0.0130337	0.00198783	5.50E-11	42.99075552	-0.018	0.0163	0.2688
rs9674487	**BMI**	G	C	0.158445	0.0286171	3.10E-08	30.65518583	-0.5946	0.2986	0.0464505
rs9674487	**BMI**	G	C	0.158445	0.0286171	3.10E-08	30.65518583	0.0971	0.0705	0.1681
rs9830592	**BMI**	A	C	0.0154849	0.00200164	1.00E-14	59.84708274	-0.0168	0.0164	0.3059
rs9839081	**BMI**	A	G	-0.011701	0.00214045	4.60E-08	29.88366792	0.029	0.0201	0.1489
rs9843653	**BMI**	C	T	0.0294509	0.00197538	2.90E-50	222.2767075	-0.0386	0.0206	0.0611801
rs9876664	**BMI**	T	G	-0.0180478	0.00204174	9.60E-19	78.13502988	0.016	0.0196	0.4145
rs9888533	**BMI**	T	C	0.0120038	0.0020182	2.70E-09	35.37587705	-0.004	0.0174	0.8186
rs9926784	**BMI**	C	T	-0.023818	0.00254677	8.60E-21	87.46398262	4.00E-04	0.0163	0.9828
rs9951619	**BMI**	G	T	0.0144231	0.00235777	9.50E-10	37.42074866	-0.0447	0.0224	0.0462498

BMI: Body mass index. EA: effect allele. OA: other allele. GX: beta-exposure. GX(SE): standard error of GX. GY: beta-outcome. GY(SE): standard error of GY.

Table S13. Published associations of TG on PTB

SNP	Exposure	EA	OA	GX	GX(SE)	Pval-exp	F	GY	GY(SE)	Pval-outcome
rs10210970	**TG**	T	C	0.0205	0.0029	9.46E-13	49.96998295	0.0166	0.0206	0.4211
rs10211562	**TG**	T	G	-0.0131	0.002	9.71E-11	42.90225056	6.00E-04	0.0161	0.9712
rs10242866	**TG**	T	C	0.0156	0.002	2.24E-14	60.83964627	-0.0035	0.0159	0.825
rs10513686	**TG**	A	G	0.0272	0.0034	1.44E-15	63.9996279	-0.0065	0.0445	0.8836
rs10519336	**TG**	A	G	0.0135	0.0024	3.59E-08	31.64044104	0.0482	0.0241	0.0453096

rs1057510	**TG**	A	G	0.0153	0.0025	7.06E-10	37.45418224	0.0039	0.0173	0.8236
rs1064173	**TG**	A	G	-0.0227	0.0023	1.36E-22	97.40775124	0.1426	0.0192	1.03E-13
rs10752652	**TG**	C	T	0.0136	0.002	2.85E-11	46.23973116	-0.0208	0.0165	0.2093
rs10763928	**TG**	T	C	0.0173	0.0031	4.17E-08	31.14341934	-0.0112	0.0209	0.5942
rs10775406	**TG**	G	A	0.0206	0.0027	3.46E-14	58.21090984	-0.0053	0.0547	0.9228
rs10786114	**TG**	T	C	-0.0266	0.003	4.42E-19	78.61732069	-0.0312	0.0225	0.1656
rs10838681	**TG**	A	G	-0.0219	0.0022	1.72E-22	99.09239907	0.0276	0.0172	0.1089
rs10851698	**TG**	T	C	0.0146	0.0023	4.80E-10	40.29466175	-0.0248	0.0196	0.2065
rs10861662	**TG**	C	A	0.018	0.0023	1.05E-14	61.24728095	0.0047	0.0177	0.791199
rs10889333	**TG**	A	G	-0.0788	0.0022	1.00E-200	1282.93469	-0.0142	0.0218	0.515901
rs10954750	**TG**	G	C	-0.0141	0.0024	2.33E-09	34.51542432	-0.0175	0.0171	0.3067
rs11030102	**TG**	G	C	0.0165	0.0026	3.54E-10	40.27343448	0.08	0.0527	0.1289
rs11057837	**TG**	T	C	0.0218	0.0038	1.28E-08	32.91116599	-0.0227	0.0753	0.7633
rs11078597	**TG**	C	T	0.0183	0.0026	7.47E-13	49.5396528	0.0156	0.0198	0.429
rs1108627	**TG**	T	C	0.0181	0.0021	2.58E-17	74.28754994	0.0269	0.0199	0.1762
rs11118310	**TG**	T	A	0.019	0.002	9.22E-21	90.24947528	0.0066	0.0158	0.6788
rs111703587	**TG**	C	T	0.0372	0.0055	1.04E-11	45.74651088	0.1323	0.1142	0.2466
rs11228377	**TG**	C	T	-0.014	0.0021	1.20E-11	44.44418604	0.0176	0.0168	0.2943
rs11231694	**TG**	C	G	0.0301	0.0033	1.22E-19	83.19602685	-0.0064	0.0181	0.7214
rs112424890	**TG**	T	C	0.0175	0.0029	1.07E-09	36.41477044	-0.0722	0.0325	0.0264503
rs1128249	**TG**	T	G	-0.0336	0.0023	5.36E-50	213.4127478	-0.0632	0.0262	0.0159599
rs1133400	**TG**	G	A	0.0144	0.0024	9.86E-10	35.99979069	-0.0427	0.0176	0.0151098
rs113366589	**TG**	G	T	0.0249	0.004	6.99E-10	38.7503997	0.1392	0.1006	0.1668
rs114052230	**TG**	T	C	-0.0239	0.0032	1.10E-13	55.78190224	-0.1836	0.1521	0.2273
rs114165349	**TG**	C	G	0.0863	0.0077	4.09E-29	125.6138758	0.0272	0.1301	0.8345
rs11588907	**TG**	T	C	-0.0136	0.0024	2.54E-08	32.11092441	0.0111	0.0492	0.8213
rs115912456	**TG**	G	A	-0.0364	0.0058	4.97E-10	39.38621571	0.0474	0.1637	0.7723
rs11600815	**TG**	A	G	-0.0365	0.0053	5.71E-12	47.42763454	0.0248	0.0885	0.779301
rs11654777	**TG**	G	C	0.0404	0.0048	7.54E-17	70.83986591	-0.0496	0.0907	0.5841
rs11664106	**TG**	T	A	-0.0148	0.0025	1.70E-09	35.04619624	0.0386	0.0515	0.454
rs116843064	**TG**	A	G	-0.2279	0.0084	1.78E-163	736.0842974	-0.0719	0.1519	0.6359
rs11759549	**TG**	C	G	0.0393	0.0046	1.03E-17	72.99059642	-0.0258	0.0288	0.3708
rs11770163	**TG**	C	G	0.0145	0.0023	1.55E-10	39.74457043	0.001	0.0218	0.9651
rs11868959	**TG**	C	T	0.0226	0.0024	2.56E-20	88.67309555	-7.00E-04	0.0173	0.9682
rs12138136	**TG**	A	T	-0.0289	0.0042	4.56E-12	47.34723039	-0.228	0.1992	0.2525
rs12151142	**TG**	C	T	0.0177	0.0021	9.61E-17	71.04040329	-0.0167	0.0195	0.3925
rs12446515	**TG**	T	C	-0.0357	0.0022	7.81E-58	263.3228492	0.0043	0.0194	0.8231
rs12492351	**TG**	A	G	0.0144	0.0023	4.15E-10	39.19825981	-0.0064	0.0172	0.7098
rs12503461	**TG**	T	C	-0.0119	0.0021	2.47E-08	32.11092441	0.028	0.0192	0.1445
rs12513202	**TG**	T	C	0.0124	0.0022	7.97E-09	31.76841034	0.0147	0.0191	0.4416
rs1260326	**TG**	C	T	-0.0992	0.002	1.00E-200	2460.145696	0.0031	0.0159	0.8472
rs12878001	**TG**	G	T	0.0197	0.0029	2.25E-11	46.14598616	-0.0488	0.0297	0.1001
rs12928099	**TG**	A	C	-0.0307	0.0025	1.31E-33	150.7975232	0.0448	0.0531	0.3995
rs12928324	**TG**	C	A	0.0147	0.0023	2.47E-10	40.84853377	0.1088	0.1106	0.325

rs13066793	**TG**	G	A	-0.0229	0.004	1.65E-08	32.77543444	0.1021	0.0879	0.2457
rs13108218	**TG**	G	A	-0.0318	0.0021	3.90E-53	229.3047892	0.0315	0.0161	0.0504603
rs13280055	**TG**	A	G	0.0392	0.0035	1.30E-29	125.4392707	-0.034	0.0771	0.6591
rs1340819	**TG**	C	A	-0.012	0.0022	2.76E-08	29.75189313	-0.0132	0.0184	0.4736
rs138422027	**TG**	A	C	-0.0248	0.0035	2.73E-12	50.20705503	-0.0085	0.0278	0.759899
rs139624990	**TG**	T	C	0.0686	0.0125	4.18E-08	30.11796889	-0.0397	0.1485	0.7894
rs139974673	**TG**	C	T	0.1444	0.0074	7.85E-84	380.7749958	0.2565	0.2605	0.3247
rs141469619	**TG**	G	A	0.3364	0.0122	1.28E-166	760.3083986	-0.7171	0.3619	0.0474996
rs143791312	**TG**	T	C	-0.113	0.0065	2.46E-67	302.2230949	-1.00E-04	0.0303	0.9966
rs144423449	**TG**	A	G	-0.0549	0.0095	7.02E-09	33.39603852	0.2649	0.3065	0.3875
rs1454687	**TG**	G	C	-0.0126	0.0021	9.30E-10	35.99979069	-0.0118	0.0172	0.4921
rs149142833	**TG**	T	C	0.0187	0.0031	2.63E-09	36.38792579	-0.0947	0.0441	0.0316599
rs150460588	**TG**	C	T	0.0245	0.0039	2.48E-10	39.46393886	0.0289	0.0219	0.1867
rs1532085	**TG**	G	A	-0.0353	0.0021	2.67E-66	282.5584479	0.0056	0.016	0.7259
rs154254	**TG**	C	G	0.0184	0.0021	9.90E-18	76.7705287	-0.0031	0.0162	0.8461
rs1549293	**TG**	T	C	-0.0151	0.0023	4.40E-11	43.1018288	0.0174	0.0248	0.4842
rs1556494	**TG**	G	A	-0.0123	0.002	1.47E-09	37.8222801	-0.0067	0.016	0.674401
rs1672981	**TG**	C	T	0.0315	0.004	6.18E-15	62.01526444	0.0083	0.0312	0.7895
rs17052061	**TG**	G	T	-0.0312	0.0029	1.90E-26	115.7472462	-0.0485	0.044	0.27
rs17184382	**TG**	C	A	-0.018	0.0022	1.07E-15	66.94175955	0.0415	0.0278	0.1359
rs17252123	**TG**	A	G	0.029	0.0048	2.17E-09	36.50152389	0.1571	0.1204	0.1921
rs17311740	**TG**	T	C	-0.0264	0.0046	1.17E-08	32.93742665	0.0989	0.0932	0.2888
rs17326656	**TG**	T	G	0.016	0.0027	4.20E-09	35.11639391	-0.0194	0.0563	0.7307
rs174574	**TG**	C	A	-0.0465	0.0021	6.84E-110	490.3032718	-0.0456	0.0162	0.00486206
rs17496249	**TG**	G	A	-0.015	0.002	1.70E-13	56.24967296	-0.0176	0.0161	0.2744
rs177430	**TG**	T	C	0.0168	0.0023	5.03E-13	53.35318696	0.0599	0.1109	0.589
rs181193678	**TG**	T	C	-0.014	0.0024	8.90E-09	34.02757994	0.0908	0.1161	0.4342
rs1835346	**TG**	G	A	-0.0427	0.0076	1.98E-08	31.56647159	0.2887	0.1879	0.1246
rs1853413	**TG**	G	C	-0.0122	0.0021	9.96E-09	33.75037066	0.0219	0.0168	0.192
rs186696265	**TG**	T	C	-0.1023	0.0096	1.92E-26	113.5550038	0.0921	0.1905	0.628599
rs188247550	**TG**	T	C	-0.1366	0.0106	3.94E-38	166.0684542	0.1334	0.1168	0.2533
rs1883025	**TG**	T	C	-0.0176	0.0023	1.47E-14	58.55542515	-0.0181	0.0177	0.3052
rs1928496	**TG**	T	C	0.0153	0.0024	1.62E-10	40.64038871	0.0033	0.0215	0.8787
rs202186505	**TG**	C	T	-0.0189	0.0032	3.79E-09	34.88358625	-0.1303	0.1518	0.3907
rs2039098	**TG**	T	C	0.0158	0.0024	2.50E-11	43.34002579	0.0906	0.1127	0.4216
rs2067819	**TG**	A	G	-0.0228	0.0027	9.73E-17	71.30822738	0.0133	0.0361	0.7125
rs2068888	**TG**	A	G	-0.0312	0.002	1.62E-53	243.3585851	-0.0214	0.0162	0.1868
rs2070895	**TG**	A	G	0.0524	0.0023	6.53E-115	519.0442412	0.021	0.016	0.189
rs2070971	**TG**	T	G	0.0196	0.0027	2.66E-13	52.69653861	0.0275	0.0177	0.1203
rs2081194	**TG**	C	G	-0.0207	0.0024	1.70E-17	74.39019249	-0.0611	0.0509	0.2302
rs2122982	**TG**	A	G	-0.0269	0.0025	3.29E-26	115.7769269	-0.041	0.0265	0.122
rs2130382	**TG**	G	C	-0.0242	0.0023	4.68E-26	110.7063507	-0.0241	0.0219	0.2716
rs213484	**TG**	C	G	-0.0114	0.002	2.78E-08	32.4898111	-0.001	0.0171	0.9548
rs2160348	**TG**	C	T	0.0142	0.0025	1.17E-08	32.26221242	-0.041	0.0271	0.13

rs2209815	**TG**	T	G	-0.0126	0.002	6.45E-10	39.68976924	-0.0229	0.016	0.153
rs2267373	**TG**	T	C	0.0208	0.0021	3.52E-24	98.103738	-0.0183	0.0164	0.265
rs2281721	**TG**	T	C	-0.0429	0.0021	2.47E-89	417.3241042	0.019	0.0188	0.3132
rs2288912	**TG**	G	C	0.0114	0.002	2.41E-08	32.4898111	-0.0171	0.0166	0.301
rs2302883	**TG**	C	T	0.0147	0.0025	6.03E-09	34.57419898	0.0099	0.0258	0.7014
rs2303566	**TG**	A	G	-0.0183	0.0022	5.04E-17	69.19174647	-0.0052	0.0224	0.8157
rs2306363	**TG**	T	G	-0.0167	0.0024	4.26E-12	48.41812127	0.0034	0.018	0.8503
rs2540945	**TG**	G	A	-0.0177	0.0021	4.53E-17	71.04040329	4.00E-04	0.017	0.9803
rs2618567	**TG**	T	G	0.0123	0.0022	1.02E-08	31.25808272	0.0099	0.0175	0.5716
rs2678379	**TG**	G	A	0.0592	0.0024	2.17E-131	608.4409069	-0.0162	0.018	0.3672
rs2699805	**TG**	A	G	-0.0162	0.0021	6.08E-15	59.50985808	-0.0027	0.0167	0.8739
rs2792751	**TG**	C	T	0.0193	0.0023	3.53E-17	70.41357926	-0.0164	0.0186	0.3762
rs2812208	**TG**	C	G	-0.0515	0.0081	1.74E-10	40.42424294	0.0255	0.1656	0.8776
rs2823291	**TG**	C	G	0.0164	0.0026	1.49E-10	39.78675092	-0.0196	0.0214	0.3587
rs28383314	**TG**	C	T	0.0343	0.0021	4.16E-59	266.7762267	0.069	0.018	0.0001305
rs28419182	**TG**	A	G	0.0125	0.0023	4.30E-08	29.53669027	0.0075	0.016	0.6375
rs28471687	**TG**	G	A	0.028	0.0035	9.89E-16	63.9996279	-0.0059	0.0244	0.8082
rs28572074	**TG**	C	T	0.0641	0.0074	6.03E-18	75.03261707	-0.1487	0.0339	1.16E-05
rs28679685	**TG**	G	A	-0.0338	0.0025	9.86E-43	182.7893372	0.0184	0.0215	0.3929
rs28712486	**TG**	A	C	-0.0234	0.0028	2.63E-17	69.84143067	-0.0298	0.1323	0.8219
rs28752924	**TG**	C	T	0.0317	0.0024	3.14E-39	174.4590551	0.1681	0.0912	0.0652995
rs28752924	**TG**	C	T	0.0317	0.0024	3.14E-39	174.4590551	0.0226	0.0549	0.680299
rs2896906	**TG**	C	T	0.0184	0.0027	1.66E-11	46.44143094	0.026	0.028	0.3529
rs2925979	**TG**	C	T	-0.0287	0.0022	6.16E-40	170.1828948	-0.0106	0.0167	0.5266
rs293427	**TG**	T	A	0.0165	0.0021	1.45E-14	61.73433495	-3.00E-04	0.0161	0.9866
rs2939312	**TG**	A	G	0.0136	0.0021	1.31E-10	41.94079923	-0.0249	0.0169	0.1403
rs2943645	**TG**	T	C	0.0397	0.0023	5.66E-67	297.9358859	-0.0216	0.0257	0.3994
rs2954021	**TG**	G	A	-0.0833	0.002	1.00E-200	1734.712414	0.0142	0.0158	0.3698
rs2963469	**TG**	T	A	0.0128	0.0022	2.61E-09	33.85104286	8.00E-04	0.0178	0.9641
rs328	**TG**	G	C	-0.1871	0.0032	1.00E-200	3418.574851	0.006	0.0241	0.8016
rs34682685	**TG**	A	G	0.0331	0.0038	2.96E-18	75.87282756	0.0506	0.0969	0.6016
rs35225200	**TG**	C	A	0.0313	0.0043	4.84E-13	52.98454862	-0.0512	0.1384	0.7112
rs35859536	**TG**	T	C	-0.012	0.0021	1.60E-08	32.65287138	-0.0195	0.016	0.2247
rs35932591	**TG**	T	C	0.0173	0.0028	8.66E-10	38.17452295	0.0093	0.0188	0.6208
rs3735080	**TG**	T	C	0.0157	0.0024	1.45E-10	42.79315397	0.0095	0.0203	0.6387
rs374796059	**TG**	G	A	0.0239	0.0037	1.01E-10	41.72437392	0.096	0.0856	0.2618
rs37507	**TG**	A	G	-0.0114	0.002	1.89E-08	32.4898111	5.00E-04	0.0161	0.9736
rs3775228	**TG**	T	C	0.0354	0.0021	6.07E-66	284.1616132	-0.0182	0.0164	0.267
rs38205	**TG**	C	A	-0.0117	0.0021	1.77E-08	31.04063585	-0.0213	0.016	0.1816
rs3936510	**TG**	T	G	0.0431	0.0029	1.70E-49	220.8798097	-0.0476	0.0667	0.4757
rs4149056	**TG**	C	T	0.025	0.0028	4.82E-19	79.71892426	0.0199	0.0219	0.365
rs4257266	**TG**	G	A	-0.015	0.0022	4.31E-12	46.48733302	-0.0162	0.0161	0.3149
rs4410790	**TG**	C	T	0.0144	0.0021	5.10E-12	47.02013478	-8.00E-04	0.0163	0.9626
rs4622313	**TG**	T	C	-0.0159	0.0023	9.09E-12	47.78989228	-0.0389	0.1107	0.725499

rs4722551	**TG**	C	T	-0.0375	0.0031	3.87E-33	146.3310951	0.0103	0.046	0.8231
rs4731702	**TG**	T	C	-0.0296	0.002	2.51E-47	219.0387265	0.0232	0.0171	0.1748
rs4800395	**TG**	C	T	-0.014	0.0023	2.29E-09	37.05082428	-0.0221	0.1112	0.8422
rs4843756	**TG**	T	C	0.0142	0.0023	1.06E-09	38.11698065	0.0488	0.0505	0.3347
rs492443	**TG**	G	A	0.0172	0.0023	8.65E-14	55.92406048	-0.0254	0.0189	0.1795
rs4945823	**TG**	A	T	0.0183	0.0029	3.82E-10	39.82022032	0.0437	0.1393	0.753701
rs4947121	**TG**	C	T	0.0151	0.0028	4.46E-08	29.08273907	0.0039	0.054	0.9422
rs4969179	**TG**	G	T	-0.0174	0.002	1.65E-17	75.68955993	0.0119	0.0159	0.4546
rs4976033	**TG**	G	A	0.0158	0.0021	1.58E-14	56.60738063	-0.021	0.016	0.1891
rs5005705	**TG**	A	C	-0.02	0.003	4.02E-11	44.44418604	0.0069	0.0385	0.857
rs5112	**TG**	G	C	0.0683	0.0025	2.18E-166	746.3780605	0.0277	0.0501	0.580301
rs5117	**TG**	C	T	0.0892	0.0025	1.00E-200	1273.054998	-0.0079	0.0263	0.7652
rs550057	**TG**	T	C	-0.0191	0.0023	6.87E-17	68.96179186	-0.0105	0.0176	0.5495
rs553779	**TG**	G	A	-0.0189	0.0021	4.88E-19	80.99952906	0.0075	0.02	0.7069
rs55747707	**TG**	A	G	-0.1069	0.0026	1.00E-200	1690.465024	-5.00E-04	0.0254	0.9831
rs57994353	**TG**	C	T	0.0143	0.0023	1.08E-09	38.65572988	-0.0278	0.0232	0.2309
rs58542926	**TG**	T	C	-0.0934	0.0038	1.23E-134	604.1211413	0.0338	0.0298	0.2561
rs59096593	**TG**	C	T	-0.0151	0.0025	2.94E-09	36.48138789	0.0545	0.0517	0.2917
rs59104589	**TG**	T	C	-0.0115	0.0021	2.44E-08	29.98848777	0.0011	0.0159	0.9471
rs6066138	**TG**	A	G	-0.0198	0.0026	1.62E-14	57.99374566	-0.0115	0.056	0.8377
rs6073958	**TG**	C	T	0.0545	0.0027	1.43E-93	407.4393321	0.0348	0.0249	0.1619
rs61778883	**TG**	C	T	0.0176	0.0027	1.14E-10	42.49083663	0.0088	0.0182	0.6312
rs61993685	**TG**	C	T	-0.0239	0.0043	3.21E-08	30.89273548	-0.0256	0.0875	0.7698
rs62112763	**TG**	G	C	0.0145	0.002	1.08E-12	52.5621944	0.0167	0.0162	0.3008
rs62466875	**TG**	T	C	0.0421	0.007	2.40E-09	36.17142235	0.0155	0.1397	0.9115
rs62521590	**TG**	G	T	0.0313	0.0026	1.13E-32	144.9237136	-0.0748	0.1251	0.5497
rs632057	**TG**	G	T	-0.0276	0.0021	2.31E-38	172.7336896	-0.0119	0.0179	0.5048
rs6432622	**TG**	G	A	-0.0126	0.0022	9.32E-09	32.80146218	0.0134	0.0243	0.580301
rs6490029	**TG**	A	G	-0.0132	0.0023	1.42E-08	32.93742665	-0.0014	0.0168	0.9354
rs6492721	**TG**	C	T	-0.015	0.0021	2.32E-12	51.02011153	-0.0173	0.0164	0.2915
rs6542680	**TG**	T	C	0.0143	0.0025	1.23E-08	32.71820977	0.0041	0.0175	0.8137
rs6606694	**TG**	T	C	-0.0388	0.0065	2.11E-09	35.63150881	0.0172	0.0413	0.6773
rs664732	**TG**	C	G	0.0325	0.0059	3.62E-08	30.34311574	-0.0777	0.0973	0.4251
rs6658257	**TG**	G	A	0.0192	0.002	9.29E-21	92.15946417	0.0202	0.0162	0.2118
rs6730325	**TG**	A	G	-0.0135	0.0024	1.27E-08	31.64044104	-0.0681	0.0489	0.1641
rs6792725	**TG**	G	A	-0.0154	0.0022	1.43E-12	48.99971511	0.0121	0.0158	0.4435
rs684773	**TG**	C	A	0.026	0.0024	8.82E-27	117.3604288	0.0095	0.0206	0.643
rs6882076	**TG**	C	T	0.035	0.0022	2.33E-57	253.097702	-0.018	0.0202	0.3716
rs6913325	**TG**	T	G	-0.0119	0.002	4.76E-09	35.40229417	-0.0146	0.0162	0.367
rs6923071	**TG**	T	C	-0.0169	0.002	1.43E-16	71.40208486	0.0047	0.0162	0.770699
rs6938091	**TG**	G	A	-0.0153	0.0027	1.48E-08	32.11092441	-0.0101	0.029	0.727999
rs6977665	**TG**	G	A	0.0145	0.002	1.25E-12	52.5621944	0.0072	0.016	0.6534
rs7017739	**TG**	G	C	0.0212	0.0035	2.25E-09	36.68876628	0.0123	0.0703	0.8615
rs7076938	**TG**	T	C	-0.0177	0.0023	4.66E-14	59.22271805	-0.0071	0.0198	0.718701

rs707931	**TG**	G	A	0.0289	0.0047	7.36E-10	37.8091962	-0.0025	0.055	0.9634
rs7140110	**TG**	C	T	0.0245	0.0023	2.84E-27	113.4681494	0.0271	0.0196	0.1664
rs71603401	**TG**	G	A	0.0253	0.0032	1.40E-15	62.50842563	-0.0248	0.0321	0.4392
rs7225606	**TG**	G	A	-0.0154	0.0023	4.45E-11	44.83149738	-0.0148	0.048	0.7571
rs7250869	**TG**	C	T	-0.0188	0.0021	1.13E-18	80.14465875	-0.0275	0.0159	0.0841008
rs72663520	**TG**	T	C	0.0233	0.0024	2.23E-21	94.25118812	-0.0112	0.0208	0.5888
rs72691637	**TG**	A	G	-0.0147	0.0026	2.00E-08	31.96579048	-0.0391	0.021	0.0628695
rs72801474	**TG**	A	G	-0.0314	0.004	4.46E-15	61.62214172	-0.0694	0.0993	0.4849
rs72815843	**TG**	A	G	-0.0181	0.0029	3.15E-10	38.95458921	-0.0274	0.066	0.677699
rs72836561	**TG**	T	C	0.1412	0.0066	2.01E-101	457.6979817	-0.0604	0.1372	0.6599
rs7284966	**TG**	G	A	0.0153	0.0021	4.29E-13	53.08132403	0.0129	0.0162	0.4234
rs72959041	**TG**	A	G	0.0595	0.0054	2.33E-28	121.4070445	-0.0219	0.1006	0.8278
rs729761	**TG**	G	T	0.0189	0.0024	5.11E-15	62.01526444	-0.0178	0.0252	0.48
rs7298844	**TG**	G	A	0.015	0.0026	1.05E-08	33.28383015	-0.0086	0.0254	0.734401
rs73224072	**TG**	C	G	0.0174	0.003	5.59E-09	33.63980441	0.0864	0.0604	0.1527
rs73990245	**TG**	C	T	0.0182	0.0032	1.95E-08	32.34746818	0.0548	0.1528	0.719701
rs742036	**TG**	A	G	-0.0139	0.0021	2.04E-11	43.81153666	-0.022	0.0163	0.1764
rs75398587	**TG**	G	C	-0.0274	0.0045	1.57E-09	37.07435235	0.1403	0.1171	0.231
rs754600	**TG**	A	G	0.0155	0.0024	2.24E-10	41.70982694	0.0067	0.0165	0.6831
rs7571753	**TG**	C	T	-0.0128	0.0023	2.53E-08	30.97146454	0.007	0.0272	0.7959
rs759404	**TG**	T	C	0.0221	0.0038	5.34E-09	33.82321055	0.0316	0.0273	0.2472
rs76308736	**TG**	A	G	0.0334	0.0044	4.71E-14	57.62156581	0.0037	0.0576	0.9483
rs7631606	**TG**	G	T	-0.0156	0.0026	3.96E-09	35.99979069	-0.1488	0.1262	0.2385
rs7639927	**TG**	T	A	0.0121	0.0022	2.45E-08	30.24982412	0.0261	0.0219	0.2316
rs76669111	**TG**	T	G	-0.0235	0.0029	1.01E-15	65.66549217	-0.0395	0.0278	0.1553
rs76895963	**TG**	G	T	-0.0907	0.0089	2.12E-24	103.8561062	0.2973	0.152	0.0505196
rs76957426	**TG**	T	C	0.0133	0.0024	2.04E-08	30.70989089	0.0036	0.0237	0.8785
rs77009508	**TG**	G	A	0.0452	0.0044	1.13E-24	105.5283121	-0.2053	0.0993	0.03874
rs77342729	**TG**	G	A	0.0388	0.0067	7.11E-09	33.53600462	-0.0651	0.1222	0.593999
rs7861679	**TG**	T	C	0.0124	0.0021	6.69E-09	34.86601044	0.014	0.016	0.3816
rs7898735	**TG**	C	T	0.0196	0.0032	5.47E-10	37.51540688	-0.0213	0.0204	0.2952
rs79192570	**TG**	A	G	-0.0276	0.0033	6.09E-17	69.95000653	0.0754	0.087	0.3864
rs7924036	**TG**	T	G	-0.0311	0.002	4.37E-52	241.8010941	0.0198	0.0169	0.2428
rs7947951	**TG**	G	A	0.0182	0.0022	7.04E-17	68.43761862	0.001	0.0173	0.9558
rs79719909	**TG**	G	A	-0.0263	0.0026	1.26E-23	102.320411	-0.0112	0.0231	0.6281
rs80137470	**TG**	A	G	0.026	0.0042	9.15E-10	38.32177266	0.0073	0.0253	0.771999
rs8025505	**TG**	T	C	0.0187	0.0022	5.86E-17	72.24957993	-0.0319	0.0163	0.0508101
rs8049676	**TG**	G	A	-0.0157	0.0021	9.86E-14	55.89309907	-6.00E-04	0.0172	0.9715
rs8088001	**TG**	G	T	-0.0291	0.0041	1.56E-12	50.37507892	-0.0446	0.037	0.2288
rs8126001	**TG**	T	C	-0.0153	0.0021	8.99E-14	53.08132403	0.0225	0.0178	0.2079
rs852392	**TG**	A	G	0.0151	0.0027	1.29E-08	31.27691006	-0.0551	0.0305	0.0708304
rs863750	**TG**	T	C	0.024	0.002	9.55E-32	143.9991628	-0.0065	0.0158	0.6814
rs870526	**TG**	T	C	-0.0164	0.002	4.35E-16	67.23960906	0.0178	0.0159	0.2637
rs917196	**TG**	G	C	-0.0133	0.0024	3.65E-08	30.70989089	-0.066	0.0203	0.00117201

rs9297994	**TG**	A	G	-0.0232	0.0022	3.84E-25	111.205965	-0.0213	0.0206	0.3009
rs940904	**TG**	A	G	0.0154	0.0024	1.14E-10	41.17337172	-0.0286	0.0217	0.1868
rs9425589	**TG**	A	G	-0.0122	0.0022	3.96E-08	30.75188732	-0.0094	0.0245	0.702
rs943265	**TG**	C	T	0.0169	0.0026	4.06E-11	42.24975435	0.0053	0.018	0.7668
rs964184	**TG**	C	G	-0.2239	0.0027	1.00E-200	6876.669209	0.0085	0.0183	0.640801
rs9831084	**TG**	C	T	-0.0128	0.0021	2.29E-09	37.15171143	0.0013	0.0209	0.9512
rs9844972	**TG**	C	G	0.0418	0.0046	8.46E-20	82.57229874	0.155	0.1036	0.1347
rs988260	**TG**	T	G	0.0116	0.0021	1.86E-08	30.51229425	-0.0177	0.017	0.3001
rs9892862	**TG**	G	A	-0.013	0.0023	1.80E-08	31.9468842	0.0025	0.0171	0.8819
rs998584	**TG**	A	C	0.0362	0.002	4.47E-72	327.6080952	-0.0047	0.0158	0.7662

TG: Triglyceride. EA: effect allele. OA: other allele. GX: beta-exposure. GX(SE): standard error of GX. GY: beta-outcome. GY(SE): standard error of GY.

Table S14. Published associations of TC on PTB

SNP	Exposure	EA	OA	GX	GX(SE)	Pval-exp	F	GY	GY(SE)	Pval-outcome
rs10104187	**TC**	C	T	0.011671	0.00206671	2.40E-08	31.89004064	-0.0025	0.0171	0.8824
rs10128711	**TC**	C	T	0.0178721	0.00235341	5.90E-15	57.67053747	-0.0383	0.0159	0.0157398
rs10189685	**TC**	A	G	-0.0234429	0.00223653	3.30E-26	109.8680029	-0.108	0.0382	0.00471097
rs103294	**TC**	T	C	0.0209511	0.00244172	1.50E-18	73.62409254	0.0194	0.0182	0.2869
rs1041983	**TC**	T	C	-0.0121219	0.00221159	2.30E-08	30.04209224	-0.0054	0.0173	0.7538
rs1049107	**TC**	T	C	0.0261402	0.00392734	1.40E-11	44.30154034	-0.1353	0.0252	8.08E-08
rs1057868	**TC**	T	C	0.0132405	0.00228772	5.00E-09	33.49662488	0.0235	0.016	0.1432
rs10753556	**TC**	G	A	0.0214427	0.00308866	2.20E-12	48.19663835	-0.014	0.0177	0.4289
rs10794579	**TC**	C	T	0.0165896	0.00207924	2.00E-16	63.6591159	0.0044	0.0159	0.7813
rs10892881	**TC**	A	C	0.0142248	0.00238286	1.00E-09	35.63635694	0.0232	0.0274	0.3977
rs10896018	**TC**	A	G	0.012672	0.00230668	1.50E-08	30.17961149	0.0082	0.0161	0.6124
rs10903129	**TC**	G	A	0.0270006	0.00205323	8.00E-43	172.9297223	0.0179	0.0167	0.2844
rs10919615	**TC**	T	C	0.0125573	0.00237517	3.40E-08	27.95124505	0.0078	0.0214	0.7147
rs11088472	**TC**	C	A	0.0117383	0.00207955	2.20E-09	31.86175488	-0.0325	0.016	0.0419904
rs11136343	**TC**	G	A	0.0135503	0.00215717	1.70E-10	39.45727544	0.0027	0.021	0.8985
rs11220462	**TC**	A	G	0.0324924	0.00301825	1.10E-28	115.8913921	0.0212	0.0165	0.1984
rs11226108	**TC**	C	G	-0.0198579	0.00265174	1.50E-14	56.07932474	-0.0159	0.0232	0.4937
rs112849259	**TC**	T	C	0.162411	0.00644311	6.30E-147	635.3862399	-0.1038	0.1624	0.5229
rs114245489	**TC**	T	G	0.0270534	0.00435352	1.80E-10	38.61540874	-0.2529	0.1237	0.0408696
rs11591147	**TC**	T	G	-0.32722	0.00790383	1.00E-200	1713.967236	0.0891	0.1332	0.5037
rs11601507	**TC**	A	C	0.0258947	0.00414175	9.10E-10	39.08877158	-0.0302	0.0344	0.3791
rs116529593	**TC**	G	T	-0.0707131	0.0118205	4.50E-09	35.78706443	-0.0322	0.3323	0.9228

rs116863821	**TC**	G	T	0.0348798	0.00650777	3.20E-08	28.72642605	-0.0059	0.189	0.975
rs1169294	**TC**	A	G	0.0290237	0.00223126	6.10E-39	169.2011106	0.0081	0.0159	0.6109
rs117139027	**TC**	A	G	-0.0588834	0.00798919	7.40E-15	54.32231561	0.0198	0.3088	0.9489
rs11755266	**TC**	T	C	-0.0351937	0.00351775	6.20E-25	100.0916684	-0.0022	0.0326	0.9473
rs117733303	**TC**	G	A	0.0733133	0.00761546	1.20E-23	92.67685142	-0.0628	0.0254	0.0135301
rs118057319	**TC**	C	T	-0.0286981	0.00451297	9.00E-11	40.4370451	0.0566	0.0909	0.5337
rs12046278	**TC**	C	T	-0.0157335	0.00214437	1.40E-14	53.83308391	0.0038	0.0192	0.8431
rs12162782	**TC**	G	T	0.0131076	0.00216263	6.30E-10	36.73499028	-0.0054	0.0169	0.751299
rs12208357	**TC**	T	C	0.0533214	0.00406315	1.50E-40	172.2167514	-0.1	0.1001	0.3176
rs12336893	**TC**	G	T	-0.0254012	0.00453073	1.90E-09	31.43186281	0.0214	0.0444	0.6297
rs12354278	**TC**	T	A	0.0149833	0.00306928	4.00E-08	23.83087276	-0.0312	0.0525	0.5521
rs12444979	**TC**	T	C	0.0163146	0.00293854	3.90E-08	30.82390541	-0.0882	0.0715	0.2172
rs1250259	**TC**	A	T	0.0139445	0.00232312	1.20E-09	36.02969802	0.0467	0.03	0.1193
rs12575519	**TC**	G	T	-0.0156791	0.00275102	6.20E-10	32.48274935	0.0469	0.0317	0.1387
rs12666989	**TC**	C	G	0.0216093	0.00266236	1.30E-16	65.87882651	0.0535	0.0445	0.2296
rs12916	**TC**	C	T	0.0616201	0.00209452	5.11E-199	865.513389	5.00E-04	0.0158	0.9757
rs13071688	**TC**	A	G	-0.0319772	0.00374998	9.20E-18	72.71449291	0.0354	0.0868	0.683299
rs13107325	**TC**	T	C	-0.0406508	0.0039614	4.10E-24	105.3025324	0.0932	0.1558	0.5499
rs1378942	**TC**	A	C	0.015801	0.00219719	1.90E-13	51.71683455	-0.0069	0.0192	0.720701
rs138354	**TC**	C	T	-0.0123777	0.00206325	5.30E-09	35.98936746	0.0066	0.019	0.726501
rs140970775	**TC**	G	A	0.0230861	0.00401156	7.20E-10	33.11864629	-0.0169	0.1007	0.8666
rs1458038	**TC**	T	C	-0.0170727	0.00226635	7.30E-13	56.7477274	-0.0209	0.0167	0.2103
rs146534110	**TC**	T	G	0.0662846	0.00897017	5.60E-15	54.60368449	0.1116	0.2862	0.6965
rs1497406	**TC**	G	A	0.0122523	0.00206572	8.10E-11	35.17955583	-8.00E-04	0.0195	0.9692
rs1501908	**TC**	C	G	0.0399994	0.00212995	1.80E-80	352.6680795	-0.0191	0.0202	0.3452
rs1524776	**TC**	A	C	-0.0118663	0.00206522	1.10E-08	33.01383829	-0.0103	0.0161	0.521601
rs1532085	**TC**	G	A	-0.0517457	0.00211051	2.40E-137	601.1345927	0.0056	0.016	0.7259
rs1556562	**TC**	T	G	0.0203091	0.0024767	2.80E-17	67.24075236	-0.0401	0.0455	0.3782
rs1574525	**TC**	G	A	0.0131229	0.00229671	8.60E-09	32.64715435	0.0189	0.0196	0.3359
rs1678299	**TC**	C	G	0.0138176	0.00239991	1.00E-08	33.14922186	0.0199	0.0168	0.234
rs16926246	**TC**	T	C	-0.0242112	0.00305243	2.90E-15	62.91282985	-0.107	0.0887	0.2279
rs17129638	**TC**	C	T	-0.0171817	0.00289266	3.50E-09	35.28056241	0.0192	0.0184	0.2969
rs17347726	**TC**	C	G	-0.0408241	0.00323156	3.70E-37	159.5904161	0.0253	0.0378	0.5037
rs17369400	**TC**	G	A	-0.0208502	0.00336364	1.40E-10	38.42372363	0.0117	0.0717	0.8709
rs17381154	**TC**	C	T	-0.0529118	0.0063746	2.50E-17	68.89650819	0.1137	0.0599	0.0575599
rs174550	**TC**	C	T	-0.0468294	0.00215959	1.50E-112	470.2107867	0.0466	0.0162	0.00398602
rs17512204	**TC**	A	G	-0.0341377	0.00371179	7.20E-20	84.58624319	0.0581	0.0894	0.515901
rs17563605	**TC**	C	T	0.0119092	0.00231831	4.90E-08	26.38883252	-0.0457	0.0399	0.252
rs17580	**TC**	A	T	0.0328721	0.00490226	2.60E-11	44.96351276	0.188	0.1921	0.3278
rs17657174	**TC**	G	C	-0.0145135	0.00245858	2.40E-10	34.84766171	0.0294	0.0273	0.2803
rs17789218	**TC**	C	T	-0.0164185	0.00239283	2.40E-12	47.08052325	0.0541	0.0586	0.3561
rs179442	**TC**	T	C	-0.0187549	0.0021694	3.00E-19	74.73916654	0.006	0.0163	0.7117
rs1795240	**TC**	G	A	-0.0135243	0.00204787	8.60E-11	43.61369079	0.0123	0.0158	0.436
rs1800562	**TC**	A	G	-0.0489758	0.00419371	7.20E-32	136.3842763	0.0282	0.1158	0.8077

rs1800588	**TC**	T	C	0.0630452	0.00249475	9.20E-145	638.6280758	0.0204	0.016	0.2032
rs1800961	**TC**	T	C	-0.0979902	0.00605679	9.90E-63	261.7449264	-0.0871	0.0685	0.204
rs1801689	**TC**	C	A	0.0499973	0.00601111	1.20E-17	69.18019298	0.0477	0.2132	0.8228
rs1883025	**TC**	T	C	-0.0499137	0.00235612	5.30E-103	448.7895476	-0.0181	0.0177	0.3052
rs1885163	**TC**	C	T	0.0131585	0.00226905	3.30E-10	33.62967374	-0.0153	0.0224	0.4956
rs2000999	**TC**	A	G	0.0498157	0.00280238	1.60E-75	315.9922573	-0.0166	0.0166	0.3162
rs2001945	**TC**	C	G	-0.0531204	0.0020556	1.50E-155	667.7954745	0.0164	0.0159	0.3004
rs2062251	**TC**	T	G	-0.0138873	0.00227801	1.70E-10	37.1639955	-0.0479	0.0588	0.415
rs2066714	**TC**	C	T	0.0347141	0.00311278	4.40E-30	124.3692404	-0.013	0.0169	0.4402
rs2068888	**TC**	A	G	-0.0164765	0.00206489	1.50E-16	63.66989002	-0.0214	0.0162	0.1868
rs2073547	**TC**	G	A	0.0330805	0.0026417	1.40E-38	156.8105053	0.0173	0.0163	0.2894
rs2081687	**TC**	C	T	-0.0324946	0.00217104	3.90E-53	224.0189497	-0.0174	0.0205	0.3949
rs2109505	**TC**	A	T	-0.0151653	0.00268389	7.80E-09	31.92791833	0.0243	0.0178	0.1719
rs2207132	**TC**	A	G	0.0942496	0.00572493	1.20E-65	271.0295763	-0.0196	0.1036	0.8502
rs2236510	**TC**	T	C	0.0167099	0.00240931	1.50E-13	48.10169184	0.0044	0.0165	0.791099
rs2241340	**TC**	G	A	-0.0224644	0.0022099	2.00E-28	103.3338046	-0.0094	0.018	0.6009
rs2255437	**TC**	G	A	-0.0144071	0.00205869	3.50E-15	48.97441395	-0.0111	0.0198	0.5759
rs2288153	**TC**	T	C	0.0206829	0.00243681	1.50E-18	72.040637	0.0155	0.0179	0.386
rs2290771	**TC**	G	A	-0.0120058	0.00224876	5.20E-09	28.50322557	0.0233	0.021	0.2662
rs2292641	**TC**	T	C	-0.025112	0.00291475	1.80E-17	74.226332	-0.0013	0.0195	0.9474
rs2297409	**TC**	A	G	-0.0187428	0.00259414	3.10E-14	52.20115805	-0.0256	0.0167	0.1253
rs2298624	**TC**	T	C	0.0187766	0.003045	4.60E-10	38.02395887	0.0145	0.0192	0.4493
rs2305407	**TC**	A	G	-0.0128427	0.00208122	7.70E-09	38.07805093	-0.0012	0.0178	0.9485
rs2465520	**TC**	A	G	-0.0205186	0.00324524	1.30E-10	39.97604507	0.0429	0.0367	0.2431
rs2526385	**TC**	G	T	0.0164472	0.00258571	7.80E-12	40.45966973	0.0084	0.0169	0.619001
rs2539980	**TC**	C	T	0.0182257	0.00219858	6.40E-17	68.7198029	0.0037	0.0186	0.8417
rs2618566	**TC**	T	G	-0.0233795	0.00217017	9.10E-30	116.0595663	0.0107	0.0176	0.5434
rs2642438	**TC**	G	A	0.0315229	0.00224696	4.80E-47	196.8156652	-0.0112	0.0202	0.580899
rs2706379	**TC**	T	C	-0.0158262	0.0026287	5.20E-10	36.24676612	-0.0125	0.0165	0.4475
rs2731443	**TC**	T	C	-0.0200628	0.00215726	6.10E-18	86.49203638	-0.0845	0.0513	0.0991197
rs2738447	**TC**	C	A	0.0368778	0.00208735	1.30E-72	312.1314093	0.0078	0.0162	0.629901
rs2792751	**TC**	C	T	-0.0250838	0.00232267	1.60E-30	116.6298331	-0.0164	0.0186	0.3762
rs28403550	**TC**	A	C	0.0191024	0.0028111	3.40E-11	46.17653017	0.0247	0.0172	0.152
rs2862954	**TC**	C	T	0.0188099	0.00206016	2.80E-21	83.36218482	0.0041	0.0317	0.8975
rs2875973	**TC**	T	C	0.0142559	0.00212772	1.30E-11	44.89090338	-0.0273	0.016	0.0883894
rs2928619	**TC**	T	C	0.013617	0.00215685	1.90E-11	39.85850504	0.007	0.021	0.7395
rs330089	**TC**	C	T	0.0217953	0.00345275	2.00E-10	39.84680008	3.00E-04	0.0182	0.9885
rs34265667	**TC**	A	G	-0.0330855	0.00569821	1.80E-09	33.71293163	0.2087	0.1725	0.2263
rs34529039	**TC**	A	C	-0.017106	0.00298344	8.70E-09	32.87458926	0.0166	0.0598	0.781801
rs34752362	**TC**	A	G	-0.0141313	0.00206711	3.70E-13	46.73422801	-0.0134	0.0159	0.3991
rs35882350	**TC**	G	A	0.0133081	0.0023367	3.20E-08	32.43576987	-0.0213	0.0362	0.5556
rs35939242	**TC**	C	A	-0.0203203	0.00216755	1.20E-23	87.88606066	0.0307	0.036	0.3941
rs364585	**TC**	G	A	0.0131629	0.00210665	1.40E-10	39.04059293	0.0088	0.0161	0.5833
rs3732359	**TC**	A	G	-0.0210554	0.00248764	3.50E-18	71.63907113	-0.0128	0.0159	0.4186

rs3738621	**TC**	G	A	-0.0187725	0.00249513	3.70E-15	56.6051423	-0.0118	0.0187	0.5282
rs3745681	**TC**	G	A	0.0148702	0.0020897	1.60E-14	50.63650825	-0.0034	0.0159	0.8279
rs3748176	**TC**	A	G	0.0110442	0.00207575	2.70E-08	28.30847333	-0.0028	0.0159	0.8581
rs3756772	**TC**	T	C	0.0227942	0.00210051	2.50E-28	117.7598431	-0.0011	0.0171	0.9489
rs3764261	**TC**	A	C	0.0438797	0.00219496	7.70E-92	399.642894	0.0046	0.0192	0.8117
rs3808348	**TC**	T	C	-0.0172943	0.00255685	2.10E-12	45.75025215	0.0126	0.0167	0.4495
rs3829125	**TC**	G	C	-0.0192622	0.00285991	4.70E-11	45.36334379	-0.0387	0.0278	0.1639
rs4245791	**TC**	T	C	-0.0536682	0.00218127	4.20E-140	605.3593056	-0.0406	0.0563	0.4708
rs4530754	**TC**	A	G	0.0170266	0.00205867	2.80E-18	68.40382884	-0.0213	0.0168	0.206
rs4565995	**TC**	C	T	0.0211293	0.00356163	3.00E-09	35.19416239	0.0275	0.0684	0.687999
rs471705	**TC**	G	T	0.039548	0.00212996	2.90E-79	344.7499185	-0.0279	0.0183	0.1272
rs4752805	**TC**	G	A	0.0208296	0.00236394	1.10E-20	77.64025941	0.0261	0.0194	0.1779
rs4788815	**TC**	T	A	0.0200077	0.00216174	1.20E-21	85.66145223	-0.0287	0.0169	0.0895901
rs4841132	**TC**	G	A	0.0813382	0.00379118	4.60E-108	460.297188	-0.0277	0.0536	0.6056
rs4850047	**TC**	C	T	-0.0219774	0.0030195	8.20E-14	52.97617204	0.0089	0.0176	0.613901
rs4939883	**TC**	C	T	0.046089	0.00268189	1.50E-68	295.3321079	0.026	0.0194	0.1808
rs499974	**TC**	A	C	-0.0201988	0.00281867	4.10E-12	51.3523932	0.0015	0.0172	0.9318
rs505151	**TC**	A	G	-0.0678658	0.0058256	3.70E-34	135.7121213	-0.0591	0.0376	0.1163
rs507666	**TC**	A	G	0.0557048	0.00265033	2.90E-103	441.7569084	-0.0159	0.0178	0.3736
rs526936	**TC**	A	G	0.0372947	0.00204562	8.40E-75	332.3856992	-0.0078	0.0172	0.6496
rs55714927	**TC**	T	C	-0.0251049	0.00264723	6.60E-23	89.93560411	0.0022	0.0197	0.9118
rs562338	**TC**	G	A	0.0963661	0.00265637	1.00E-200	1316.041089	0.0481	0.0603	0.4253
rs56244055	**TC**	A	G	0.0170986	0.00267759	4.40E-11	40.77847374	-0.0884	0.0216	4.29E-05
rs56383182	**TC**	G	A	0.0169372	0.00213879	2.90E-17	62.71117792	-0.0147	0.017	0.3869
rs5754217	**TC**	T	G	-0.0244092	0.00261053	1.40E-21	87.42743383	0.0124	0.0159	0.4382
rs581080	**TC**	C	G	0.0251569	0.00267031	1.10E-20	88.75419697	-0.0117	0.0289	0.6854
rs58148580	**TC**	T	C	0.0197192	0.0032814	2.40E-09	36.11255801	0.0479	0.0401	0.2317
rs5888	**TC**	G	A	-0.0184861	0.00207306	2.40E-18	79.51789383	-0.0101	0.0185	0.5847
rs6013844	**TC**	C	T	0.0108021	0.00205541	2.30E-08	27.61960562	0.0014	0.0223	0.9513
rs602662	**TC**	A	G	0.0301199	0.00206386	1.80E-49	212.9828331	0.0122	0.0484	0.8003
rs6115094	**TC**	G	A	0.0127747	0.00205442	3.10E-10	38.66526292	-0.0113	0.0249	0.649901
rs6124298	**TC**	A	G	0.0180871	0.00225565	1.00E-15	64.2972606	0.002	0.0174	0.9066
rs6416553	**TC**	T	C	-0.0160858	0.00287308	1.90E-08	31.34641708	0.0255	0.02	0.2024
rs6475606	**TC**	T	C	-0.0201605	0.00205542	4.30E-25	96.20540183	-0.0431	0.0167	0.00990193
rs6511720	**TC**	T	G	-0.160111	0.00314212	1.00E-200	2596.538833	-0.0366	0.0743	0.6221
rs653178	**TC**	T	C	0.0336562	0.00205383	1.20E-64	268.5339434	-0.0582	0.0485	0.23
rs66476925	**TC**	C	G	0.0328236	0.00250256	1.10E-40	172.0289118	0.0259	0.017	0.1279
rs6741180	**TC**	A	G	-0.0125932	0.00209297	1.90E-10	36.20297089	0.028	0.0303	0.3544
rs6785233	**TC**	G	T	0.0222528	0.0038026	1.20E-08	34.2457008	0.0273	0.0415	0.5104
rs6831256	**TC**	G	A	0.017171	0.00208047	1.70E-18	68.11868842	-0.0109	0.0163	0.5043
rs7124487	**TC**	T	C	-0.0166562	0.00255073	3.80E-11	42.64036271	-0.0832	0.0701	0.2353
rs7157785	**TC**	T	G	0.0179141	0.00280582	4.00E-11	40.76321144	-0.055	0.0307	0.07322
rs7186852	**TC**	G	A	0.0119901	0.00213983	1.30E-08	31.39677805	-0.0208	0.0237	0.381
rs72631343	**TC**	G	C	-0.0277023	0.00306682	1.00E-20	81.5930371	-0.0018	0.0167	0.9126

rs72836561	**TC**	T	C	-0.0482655	0.00596592	9.10E-17	65.45107511	-0.0604	0.1372	0.6599
rs72848943	**TC**	A	G	-0.036475	0.00650866	7.00E-09	31.4054808	-0.0189	0.2494	0.9396
rs7412	**TC**	T	C	-0.372953	0.00375648	1.00E-200	9856.984289	-4.00E-04	0.0392	0.9915
rs74617384	**TC**	T	A	0.0776751	0.00381045	1.50E-94	415.5364488	0.0853	0.1087	0.4327
rs7528419	**TC**	G	A	-0.102332	0.00245684	1.00E-200	1734.871019	0.0387	0.028	0.1675
rs75865302	**TC**	T	C	0.0254753	0.00459334	6.60E-09	30.75951221	-0.1108	0.0993	0.2649
rs76272805	**TC**	A	G	-0.042564	0.00466221	2.00E-20	83.3487752	-0.0812	0.1529	0.5955
rs7640978	**TC**	T	C	-0.0321785	0.00360009	3.00E-18	79.8919261	-0.0209	0.0346	0.5466
rs7748291	**TC**	T	C	-0.0143611	0.0020555	4.80E-13	48.81333348	-4.00E-04	0.0241	0.986
rs77542162	**TC**	G	A	0.109475	0.00704362	5.40E-59	241.5661558	-0.3167	0.2464	0.1986
rs77704739	**TC**	C	T	-0.0355117	0.00517768	9.70E-12	47.04034853	-0.0157	0.1301	0.9042
rs77960347	**TC**	G	A	0.175743	0.00907837	6.90E-88	374.7471424	-0.1736	0.2667	0.515101
rs780094	**TC**	C	T	-0.0475907	0.00210004	7.80E-119	513.5551255	0.0137	0.0159	0.3902
rs78729321	**TC**	G	A	-0.0158223	0.00291936	3.00E-08	29.37392215	1.00E-04	0.0565	0.999
rs78946096	**TC**	G	A	-0.0414512	0.00445606	4.30E-21	86.53069105	0.0337	0.1005	0.737199
rs7920112	**TC**	C	T	0.0171649	0.00208423	2.40E-17	67.82491937	0.0245	0.0172	0.1538
rs799157	**TC**	C	T	-0.0283932	0.00509732	4.60E-09	31.02722266	-0.2819	0.1454	0.0524904
rs8017377	**TC**	A	G	0.0169635	0.00211712	1.30E-15	64.20043828	-0.0601	0.0306	0.0494698
rs8107974	**TC**	T	A	-0.123966	0.00391487	1.00E-200	1002.694199	0.0297	0.0277	0.2852
rs887829	**TC**	T	C	-0.0159768	0.0021976	1.60E-13	52.85429945	-0.0068	0.0231	0.7676
rs9306897	**TC**	C	T	-0.0322985	0.00215604	1.60E-53	224.4135601	0.0374	0.0235	0.1118
rs9352675	**TC**	A	G	-0.0105242	0.00206941	2.60E-08	25.86325103	0.0183	0.0164	0.2652
rs9370867	**TC**	G	A	-0.0195904	0.00206871	1.30E-23	89.67789301	0.0291	0.039	0.4562
rs9376090	**TC**	C	T	-0.0192906	0.00234592	1.80E-18	67.61812008	0.0138	0.0166	0.4067
rs9534262	**TC**	C	T	-0.0149397	0.00208978	8.70E-13	51.10702873	0.0097	0.0164	0.5543
rs9577924	**TC**	G	A	0.0251749	0.00233785	7.80E-29	115.9578726	0.0243	0.0193	0.2084
rs9635726	**TC**	T	C	-0.013976	0.00256208	2.50E-08	29.75626568	-0.0073	0.016	0.6481
rs964184	**TC**	C	G	-0.0718652	0.00301632	3.10E-136	567.6497697	0.0085	0.0183	0.640801
rs9668810	**TC**	C	T	-0.012702	0.00232657	9.90E-09	29.80643159	0.0013	0.0161	0.9333
rs970548	**TC**	C	A	0.0181143	0.00238222	6.10E-16	57.8199079	0.0461	0.0312	0.1396
rs9832727	**TC**	G	C	-0.0160494	0.00216947	1.30E-13	54.72784163	0.0295	0.0259	0.2557
rs9907571	**TC**	A	G	0.0122373	0.00222729	4.90E-08	30.18670398	0.025	0.0186	0.1797
rs9916193	**TC**	G	C	0.0149323	0.00261789	6.40E-09	32.53483745	-0.0198	0.0644	0.758799

TC: Total cholesterol. EA: effect allele. OA: other allele. GX: beta-exposure. GX(SE): standard error of GX. GY: beta-outcome. GY(SE): standard error of GY.

Table S15. Published associations of LDL-c on PTB

SNP	Exposure	EA	OA	GX	GX(SE)	Pval-exp	F	GY	GY(SE)	Pval-outcome
rs10195252	**LDL-c**	C	T	-0.0238	0.0039	3.81E-08	37.24081484	-0.0672	0.0263	0.01073
rs10490626	**LDL-c**	A	G	-0.0508	0.0069	1.70E-12	54.20311224	0.0893	0.0914	0.3286

rs10832962	**LDL-c**	T	C	0.032	0.004	6.62E-14	63.99925977	-0.0402	0.016	0.01191
rs10893499	**LDL-c**	A	G	0.0521	0.0053	3.86E-21	96.6314922	0.0231	0.0166	0.1627
rs10903129	**LDL-c**	G	A	0.0328	0.0037	3.03E-17	78.5849041	0.0179	0.0167	0.2844
rs10947332	**LDL-c**	A	G	0.0504	0.0056	6.97E-18	80.99904291	-0.022	0.0234	0.3475
rs112201728	**LDL-c**	T	C	0.0675	0.0104	8.51E-10	42.1240789	-0.097	0.1002	0.3328
rs11563251	**LDL-c**	T	C	0.0345	0.0062	4.50E-08	30.96348149	-0.0104	0.0245	0.6693
rs11591147	**LDL-c**	T	G	-0.497	0.018	8.57E-143	762.3537615	0.0891	0.1332	0.5037
rs1169288	**LDL-c**	C	A	0.0375	0.004	6.45E-21	87.88954716	-8.00E-04	0.0158	0.9593
rs12066643	**LDL-c**	T	C	-0.0389	0.0064	1.06E-08	36.94317217	-0.0142	0.0386	0.7133
rs1250229	**LDL-c**	C	T	0.0243	0.0042	3.13E-08	33.47410288	0.0439	0.0309	0.1562
rs12721109	**LDL-c**	A	G	-0.4462	0.0183	2.99E-122	594.4950116	-0.1109	0.1747	0.5256
rs12748152	**LDL-c**	T	C	0.0499	0.0066	3.21E-12	57.16210312	-0.0313	0.0719	0.663399
rs12916	**LDL-c**	C	T	0.0733	0.0038	7.79E-78	372.0793748	5.00E-04	0.0158	0.9757
rs13206249	**LDL-c**	A	G	-0.0378	0.0062	4.53E-08	37.16980253	0.0035	0.0164	0.8325
rs13277801	**LDL-c**	T	C	-0.0338	0.0038	3.99E-17	79.1154289	-0.0168	0.02	0.4002
rs1367117	**LDL-c**	A	G	0.1186	0.004	9.48E-183	879.1123371	-0.0244	0.0269	0.3635
rs1408272	**LDL-c**	G	T	-0.052	0.0083	3.68E-09	39.25051224	0.1055	0.1141	0.3549
rs1564348	**LDL-c**	C	T	0.0481	0.005	2.76E-21	92.54333005	0.034	0.0677	0.615601
rs16831243	**LDL-c**	T	C	0.0378	0.0055	9.06E-12	47.23380041	-0.0083	0.0165	0.612999
rs16891156	**LDL-c**	C	A	0.0965	0.0171	8.23E-09	31.84584701	-0.0304	0.0191	0.1121
rs17404153	**LDL-c**	T	G	-0.0336	0.0054	1.83E-09	38.71560153	0.0114	0.0216	0.5991
rs174583	**LDL-c**	T	C	-0.0522	0.0038	7.00E-41	188.6986493	0.0443	0.0161	0.00602698
rs1800961	**LDL-c**	T	C	-0.0685	0.0106	6.03E-10	41.76027265	-0.0871	0.0685	0.204
rs1801689	**LDL-c**	C	A	0.1028	0.0139	9.81E-12	54.69514949	0.0477	0.2132	0.8228
rs1883025	**LDL-c**	T	C	-0.0296	0.0044	6.14E-11	45.25567312	-0.0181	0.0177	0.3052
rs2000999	**LDL-c**	A	G	0.065	0.0046	4.22E-41	199.6668588	-0.0166	0.0166	0.3162
rs2030746	**LDL-c**	T	C	0.0214	0.0038	8.60E-09	31.71431485	0.0162	0.0159	0.3086
rs2073547	**LDL-c**	G	A	0.0485	0.0049	1.92E-21	97.96844267	0.0173	0.0163	0.2894
rs2228603	**LDL-c**	T	C	-0.104	0.0072	4.43E-44	208.639345	0.0161	0.0349	0.6447
rs2315065	**LDL-c**	A	C	0.1102	0.0158	5.23E-12	48.64476802	0.0961	0.0955	0.314
rs2328223	**LDL-c**	C	A	0.0299	0.005	5.63E-09	35.75998117	-0.0123	0.0186	0.5081
rs2390536	**LDL-c**	A	G	0.0223	0.0038	2.04E-08	34.43796748	-0.069	0.0323	0.0327801
rs2419604	**LDL-c**	G	A	-0.0302	0.004	7.49E-14	57.00184028	-0.016	0.0186	0.3883
rs247616	**LDL-c**	T	C	-0.0547	0.0041	2.57E-37	177.9925698	0.0017	0.0203	0.9322
rs2587534	**LDL-c**	A	G	0.0391	0.0037	8.06E-25	111.672193	-0.0076	0.0173	0.6617
rs2642438	**LDL-c**	G	A	0.0352	0.0042	7.32E-16	70.23951383	-0.0112	0.0202	0.580899
rs267733	**LDL-c**	G	A	-0.0331	0.0053	5.29E-09	39.00308596	0.0483	0.0346	0.163
rs2710642	**LDL-c**	A	G	0.0239	0.0038	6.09E-09	39.5570219	0.0093	0.0184	0.6134
rs2737252	**LDL-c**	A	G	-0.0314	0.0041	7.04E-14	58.65250436	0.0034	0.0177	0.8488
rs2886232	**LDL-c**	C	T	-0.0451	0.0064	3.88E-11	49.65783608	0.1215	0.0752	0.1059
rs2954029	**LDL-c**	T	A	-0.0564	0.0036	2.10E-50	245.4416063	0.0042	0.0157	0.7879
rs2965157	**LDL-c**	C	T	-0.1886	0.0112	7.29E-62	283.5582124	0.0188	0.0324	0.562999
rs314253	**LDL-c**	C	T	-0.0242	0.0038	3.44E-10	40.55630874	-0.0181	0.0159	0.2546
rs3184504	**LDL-c**	C	T	0.0268	0.0038	4.20E-12	49.73900927	-0.0543	0.0486	0.2634

rs364585	**LDL-c**	G	A	0.0249	0.0038	4.28E-10	42.93647996	0.0088	0.0161	0.5833
rs3757354	**LDL-c**	T	C	-0.0382	0.0044	2.09E-17	75.3730955	0.0199	0.0161	0.2153
rs3780181	**LDL-c**	G	A	-0.0445	0.0074	1.76E-09	36.16192423	-0.0156	0.0282	0.579301
rs4253776	**LDL-c**	G	A	0.0311	0.0059	3.35E-08	27.78508165	0.0778	0.0845	0.357
rs4530754	**LDL-c**	A	G	0.0275	0.0036	3.58E-12	58.35194887	-0.0213	0.0168	0.206
rs4722551	**LDL-c**	C	T	0.0391	0.0049	3.95E-14	63.67314954	0.0103	0.046	0.8231
rs4942486	**LDL-c**	C	T	-0.0243	0.0037	2.26E-11	43.132442	0.0083	0.0164	0.611
rs4970712	**LDL-c**	C	A	0.0339	0.0044	2.46E-13	59.35933456	-0.0243	0.0431	0.573
rs4970834	**LDL-c**	T	C	-0.1503	0.0047	1.00E-200	1022.626916	0.0564	0.0307	0.0662705
rs5763662	**LDL-c**	T	C	0.0767	0.0121	1.19E-08	40.18043657	0.0699	0.0348	0.0442497
rs579459	**LDL-c**	C	T	0.0665	0.0045	2.42E-44	218.3801871	-0.0183	0.0178	0.3019
rs6016373	**LDL-c**	G	A	-0.0349	0.0037	7.95E-19	88.96974439	-0.0124	0.0159	0.4341
rs6065311	**LDL-c**	C	T	0.0417	0.0036	1.66E-30	134.1720449	-0.0178	0.0208	0.3918
rs6504872	**LDL-c**	T	C	0.0274	0.0037	3.48E-13	54.83938976	-0.0145	0.0173	0.4022
rs6511720	**LDL-c**	T	G	-0.2209	0.0061	1.00E-200	1311.374307	-0.0366	0.0743	0.6221
rs6544713	**LDL-c**	C	T	-0.0806	0.0041	4.84E-83	386.4535914	-0.0454	0.0563	0.4204
rs6693893	**LDL-c**	C	T	-0.0767	0.0132	2.88E-08	33.76232986	-0.0264	0.1128	0.8153
rs6709904	**LDL-c**	G	A	-0.055	0.0085	4.58E-10	41.86758017	0.067	0.0381	0.0789205
rs676388	**LDL-c**	C	T	0.0265	0.0039	1.31E-11	46.16972921	0.0245	0.0485	0.612999
rs6818397	**LDL-c**	G	T	-0.0224	0.004	1.68E-08	31.3596368	0.0168	0.0159	0.2931
rs6882076	**LDL-c**	C	T	0.0456	0.0038	3.31E-31	143.9983353	-0.018	0.0202	0.3716
rs6909746	**LDL-c**	T	C	-0.0263	0.0037	7.86E-11	50.5246068	0.0796	0.0292	0.00645298
rs7254892	**LDL-c**	A	G	-0.4853	0.0119	1.00E-200	1663.107874	-4.00E-04	0.0396	0.9911
rs72902576	**LDL-c**	G	T	-0.0933	0.0133	9.58E-12	49.20955318	-0.0067	0.1141	0.9532
rs7534572	**LDL-c**	G	C	0.0407	0.0058	1.29E-11	49.240366	0.1115	0.1155	0.3347
rs7551981	**LDL-c**	T	G	0.0472	0.0038	1.36E-33	154.2807651	-0.033	0.0187	0.0779292
rs75687619	**LDL-c**	T	G	0.1735	0.0161	8.05E-24	116.1279111	-0.1043	0.1624	0.520701
rs7640978	**LDL-c**	T	C	-0.0392	0.0069	9.84E-09	32.27519756	-0.0209	0.0346	0.5466
rs7832643	**LDL-c**	T	G	0.0339	0.0038	2.67E-17	79.58421453	-0.0104	0.0194	0.592899
rs8017377	**LDL-c**	A	G	0.0303	0.0038	2.52E-15	63.57890429	-0.0601	0.0306	0.0494698
rs964184	**LDL-c**	C	G	-0.0855	0.0078	2.01E-26	NA	0.0085	0.0183	0.640801
rs9875338	**LDL-c**	A	G	-0.027	0.0037	2.21E-11	53.24993186	0.0226	0.0302	0.454099
rs9987289	**LDL-c**	G	A	0.0714	0.0066	8.53E-24	117.0315959	-0.0276	0.0536	0.6062

LDL-c: Low density lipoprotein cholesterol. EA: effect allele. OA: other allele. GX: beta-exposure. GX(SE): standard error of GX. GY: beta-outcome. GY(SE): standard error of GY.

Table S16. Published associations of HDL-c on PTB

SNP	Exposure	EA	OA	GX	GX(SE)	Pval-exp	GY	GY(SE)	pval.outcome
rs10031010	**HDL-c**	A	G	0.0138083	0.00246597	2.10E-08	0.0725	0.0207	0.000467401
rs10053349	**HDL-c**	C	T	0.0120071	0.00195336	7.90E-10	0.0175	0.0165	0.2911

rs10108282	**HDL-c**	A	T	0.0168525	0.00234262	6.30E-13	0.0183	0.0202	0.3646
rs10119644	**HDL-c**	A	T	0.013468	0.00191042	1.80E-12	-0.0025	0.016	0.8767
rs10162642	**HDL-c**	A	G	-0.0479223	0.0023419	4.60E-93	-0.0026	0.0235	0.911
rs10233430	**HDL-c**	C	T	-0.0204944	0.00192772	2.10E-26	0.0118	0.0158	0.4531
rs1045241	**HDL-c**	T	C	0.0164171	0.00215576	2.60E-14	0.0152	0.0226	0.5022
rs1047891	**HDL-c**	A	C	-0.0189073	0.00204367	2.20E-20	-0.0375	0.0219	0.0874299
rs10504477	**HDL-c**	C	T	-0.0150661	0.00193097	6.10E-15	0.0161	0.0181	0.3729
rs10513801	**HDL-c**	G	T	-0.0303325	0.00277034	6.70E-28	0.0237	0.0507	0.6404
rs1055582	**HDL-c**	T	C	0.0140251	0.00190973	2.10E-13	0.0014	0.0173	0.9357
rs10750766	**HDL-c**	A	C	-0.0185663	0.00209928	9.20E-19	-1.00E-04	0.0181	0.996
rs10774439	**HDL-c**	A	G	0.020513	0.00248318	1.40E-16	-0.018	0.0173	0.2984
rs10786114	**HDL-c**	T	C	0.0238735	0.00288231	1.20E-16	-0.0312	0.0225	0.1656
rs1083470	**HDL-c**	A	G	0.0115829	0.00195974	3.40E-09	-0.015	0.0168	0.3711
rs11009262	**HDL-c**	T	G	-0.0233289	0.00409245	1.20E-08	0.0169	0.0251	0.5022
rs11021232	**HDL-c**	C	T	-0.0167378	0.00248678	1.70E-11	-0.0305	0.0284	0.2833
rs11045171	**HDL-c**	G	A	0.0283661	0.00240558	4.30E-32	0.0078	0.0206	0.705801
rs111363680	**HDL-c**	T	C	0.0328171	0.00592877	3.10E-08	0.0012	0.1925	0.9951
rs11171710	**HDL-c**	A	G	-0.0114694	0.00192933	2.80E-09	-0.0124	0.0159	0.4368
rs112001035	**HDL-c**	A	G	-0.0466304	0.00408312	3.30E-30	0.2221	0.0913	0.01502
rs11218738	**HDL-c**	A	G	0.0234498	0.00219817	1.40E-26	0.0089	0.0289	0.757801
rs112233856	**HDL-c**	G	A	-0.0528133	0.00579493	8.00E-20	0.0175	0.1012	0.8624
rs112350227	**HDL-c**	T	C	-0.0319379	0.00582274	4.10E-08	-0.0446	0.2105	0.832
rs11239536	**HDL-c**	A	T	0.0287501	0.00223129	5.50E-38	0.0467	0.0312	0.1345
rs11254464	**HDL-c**	C	T	0.0127581	0.00193081	3.90E-11	0.0219	0.0174	0.207
rs1125873	**HDL-c**	T	A	0.0159741	0.00192237	9.60E-17	-0.0056	0.0159	0.7259
rs1132274	**HDL-c**	A	C	-0.0217809	0.00263979	1.60E-16	0.0146	0.0162	0.3688
rs113740515	**HDL-c**	A	G	0.0377752	0.00233951	1.20E-58	-0.0434	0.0163	0.00771898
rs113966472	**HDL-c**	A	G	0.0324769	0.00554864	4.80E-09	0.2396	0.1249	0.0551099
rs114165349	**HDL-c**	C	G	-0.0810512	0.00636562	3.90E-37	0.0272	0.1301	0.8345
rs115912456	**HDL-c**	G	A	0.028084	0.0047884	4.50E-09	0.0474	0.1637	0.7723
rs116006942	**HDL-c**	A	G	-0.02987	0.00403491	1.30E-13	0.2263	0.1186	0.0563002
rs11631178	**HDL-c**	C	T	0.0211366	0.0031235	1.30E-11	-0.0025	0.031	0.9351
rs11640494	**HDL-c**	A	G	-0.015349	0.00189969	6.50E-16	-0.0106	0.0289	0.7127
rs11664369	**HDL-c**	T	C	-0.0229777	0.00215861	1.80E-26	-0.0101	0.0209	0.6298
rs1168114	**HDL-c**	G	A	0.0155365	0.00199819	7.50E-15	-5.00E-04	0.0196	0.9781
rs116843064	**HDL-c**	A	G	0.206178	0.0069176	3.40E-195	-0.0719	0.1519	0.6359
rs116857878	**HDL-c**	T	C	0.0482826	0.00697677	4.50E-12	-0.0208	0.2356	0.9296
rs11688682	**HDL-c**	C	G	0.0147369	0.0022133	2.80E-11	0.0343	0.0561	0.5414
rs117230571	**HDL-c**	G	A	-0.0266417	0.00363105	2.20E-13	0.0882	0.12	0.4621
rs117291242	**HDL-c**	T	C	-0.0315912	0.00506695	4.50E-10	-0.29	0.1086	0.00755997
rs117762989	**HDL-c**	T	C	-0.0268923	0.00468314	9.30E-09	0.309	0.1893	0.1027
rs117847213	**HDL-c**	G	A	0.0295589	0.0048659	1.20E-09	-0.114	0.1571	0.4681
rs12045101	**HDL-c**	T	C	-0.0145519	0.00222909	6.70E-11	-0.0431	0.0291	0.1383
rs12046972	**HDL-c**	C	T	-0.0148213	0.00191599	1.00E-14	-0.0077	0.0172	0.652299

rs12205778	**HDL-c**	A	G	0.0162321	0.00217549	8.60E-14	-0.0332	0.0204	0.1032
rs12229011	**HDL-c**	T	C	-0.0259933	0.00322614	7.80E-16	-0.0177	0.02	0.3779
rs1225053	**HDL-c**	C	T	-0.0150376	0.00216684	3.90E-12	-0.0068	0.022	0.756901
rs1240820	**HDL-c**	A	G	0.0131835	0.00210043	3.50E-10	0.0546	0.023	0.0176901
rs12411732	**HDL-c**	A	G	-0.0305779	0.00274115	6.80E-29	0.0151	0.0192	0.4323
rs12462109	**HDL-c**	T	C	-0.0159539	0.00210812	3.80E-14	0.0179	0.0161	0.2655
rs12475332	**HDL-c**	G	T	0.012897	0.0021638	2.50E-09	-0.0079	0.0179	0.6578
rs12575456	**HDL-c**	A	G	0.0445866	0.00203342	1.40E-106	0.0385	0.017	0.02391
rs12686780	**HDL-c**	T	C	-0.0161688	0.00251034	1.20E-10	0.0163	0.0161	0.3117
rs1270076	**HDL-c**	G	A	0.014345	0.00228637	3.50E-10	0.0361	0.0345	0.2958
rs12705595	**HDL-c**	A	G	0.0110554	0.00198306	2.50E-08	-0.0091	0.0181	0.6126
rs12740374	**HDL-c**	T	G	0.0288629	0.00229106	2.20E-36	0.0388	0.028	0.1661
rs12740811	**HDL-c**	G	A	-0.0184297	0.00326047	1.60E-08	-0.0507	0.0287	0.0773499
rs12781812	**HDL-c**	T	G	0.0107203	0.00194289	3.40E-08	0.0296	0.0315	0.3471
rs1281959	**HDL-c**	G	C	0.0146574	0.00190666	1.50E-14	-0.006	0.0175	0.730601
rs12926854	**HDL-c**	G	A	0.0122457	0.00213341	9.50E-09	0.0224	0.0176	0.2017
rs12928099	**HDL-c**	A	C	0.0214263	0.00206787	3.70E-25	0.0448	0.0531	0.3995
rs12986742	**HDL-c**	C	T	-0.0106002	0.00191315	3.00E-08	0.0064	0.016	0.6885
rs12998038	**HDL-c**	T	C	0.0131918	0.0021847	1.60E-09	0.0203	0.0174	0.2439
rs13066793	**HDL-c**	G	A	0.0220769	0.00332887	3.30E-11	0.1021	0.0879	0.2457
rs13087167	**HDL-c**	C	G	0.0167458	0.00198206	2.90E-17	0.0144	0.0337	0.668499
rs13097947	**HDL-c**	C	T	0.0159634	0.00203024	3.80E-15	-0.0186	0.025	0.4556
rs13107325	**HDL-c**	T	C	-0.080346	0.00362445	7.00E-109	0.0932	0.1558	0.5499
rs13111599	**HDL-c**	G	A	0.0127921	0.00216664	3.50E-09	0.0023	0.0161	0.8862
rs13137144	**HDL-c**	A	G	0.0164073	0.00192431	1.50E-17	0.0195	0.0163	0.2322
rs13144151	**HDL-c**	G	A	0.0178695	0.00270341	3.80E-11	-0.0104	0.0176	0.5546
rs13235365	**HDL-c**	T	C	0.0258181	0.00214379	2.10E-33	0.0245	0.0207	0.2355
rs13269725	**HDL-c**	G	A	-0.0259912	0.00353253	1.90E-13	0.0023	0.0238	0.9215
rs133015	**HDL-c**	G	C	0.0205993	0.00192854	1.20E-26	0.0065	0.0173	0.7047
rs13379043	**HDL-c**	C	T	0.0198764	0.00216717	4.70E-20	0.0053	0.0175	0.763001
rs13389219	**HDL-c**	T	C	0.0277324	0.00194608	4.50E-46	-0.062	0.0262	0.0179602
rs13402475	**HDL-c**	G	C	-0.0249227	0.00249807	1.90E-23	-0.002	0.1453	0.9893
rs1349852	**HDL-c**	C	A	0.0112127	0.00192565	5.80E-09	3.00E-04	0.0178	0.9848
rs138354839	**HDL-c**	A	C	-0.060325	0.00841064	7.40E-13	0.4076	0.4757	0.3914
rs1383732	**HDL-c**	G	A	-0.0152024	0.00267698	1.40E-08	0.0074	0.0241	0.759401
rs1395221	**HDL-c**	T	G	-0.0111888	0.00195359	1.00E-08	-0.0145	0.0183	0.4286
rs140064750	**HDL-c**	C	T	-0.0440664	0.00634192	3.70E-12	0.0505	0.1561	0.746299
rs140164052	**HDL-c**	A	G	-0.0417453	0.00540785	1.20E-14	0.0146	0.1381	0.9161
rs140584594	**HDL-c**	G	A	0.031071	0.00212905	3.10E-48	-0.0279	0.1236	0.8213
rs141062196	**HDL-c**	A	G	-0.0188553	0.00241089	5.20E-15	0.063	0.0462	0.1724
rs1411432	**HDL-c**	C	A	-0.0139757	0.00245569	1.30E-08	0.0016	0.017	0.9239
rs1412234	**HDL-c**	C	T	-0.011989	0.00203583	3.90E-09	-0.0243	0.0195	0.2125
rs141440048	**HDL-c**	T	C	0.0436872	0.00756751	7.80E-09	-0.7435	0.3563	0.0369301
rs141469619	**HDL-c**	G	A	-0.203479	0.0101857	8.70E-89	-0.7171	0.3619	0.0474996

rs142288236	**HDL-c**	T	C	-0.0776969	0.00795635	1.60E-22	0.0177	0.1544	0.9089
rs1431659	**HDL-c**	G	A	0.0127918	0.00214104	2.30E-09	-0.0211	0.0174	0.224
rs144033177	**HDL-c**	C	A	-0.05557	0.0078389	1.40E-12	0.2619	0.1946	0.1785
rs144311893	**HDL-c**	T	C	0.0809615	0.0066916	1.10E-33	-0.1323	0.2431	0.5863
rs1446585	**HDL-c**	G	A	0.0167827	0.00217146	1.10E-14	0.086	0.0517	0.0965295
rs145947882	**HDL-c**	C	A	-0.164935	0.00609907	4.70E-161	-0.0327	0.1438	0.8202
rs1471251	**HDL-c**	T	A	-0.0193662	0.00195327	3.60E-23	-0.0105	0.0164	0.5201
rs147627829	**HDL-c**	A	G	-0.0535763	0.00468184	2.50E-30	-0.0388	0.1477	0.7925
rs147772065	**HDL-c**	C	G	0.0288034	0.0052496	4.10E-08	0.0348	0.1506	0.8171
rs150224153	**HDL-c**	T	C	-0.0934689	0.00575879	3.10E-59	-0.0577	0.0963	0.5487
rs150237291	**HDL-c**	C	T	0.0453058	0.00656295	5.10E-12	0.1464	0.1458	0.3152
rs150844304	**HDL-c**	C	A	-0.090543	0.00599215	1.40E-51	0.2933	0.2641	0.2667
rs150861794	**HDL-c**	T	C	-0.0446978	0.00759758	4.00E-09	-0.0728	0.2529	0.773499
rs1534696	**HDL-c**	A	C	0.0166573	0.0019113	2.90E-18	0.0451	0.0191	0.01852
rs16928809	**HDL-c**	A	G	-0.0262633	0.00329991	1.70E-15	-0.0583	0.0313	0.0628304
rs17124112	**HDL-c**	A	C	-0.020798	0.00352939	3.80E-09	0.0118	0.024	0.621999
rs17138358	**HDL-c**	C	G	-0.0272272	0.00194811	2.20E-44	-0.0058	0.0159	0.7179
rs17309930	**HDL-c**	A	C	-0.0218885	0.00235533	1.50E-20	0.0896	0.0533	0.0929394
rs17326656	**HDL-c**	T	G	-0.0223923	0.00223885	1.50E-23	-0.0194	0.0563	0.7307
rs174566	**HDL-c**	G	A	-0.0562241	0.00199551	1.20E-174	0.0457	0.0162	0.00482003
rs1760940	**HDL-c**	C	A	0.0121791	0.0022157	3.90E-08	0.0018	0.022	0.9355
rs17713879	**HDL-c**	A	G	0.0138956	0.00197564	2.00E-12	0.0116	0.0193	0.547
rs1771582	**HDL-c**	G	T	0.0129458	0.00197832	6.00E-11	-0.0158	0.0232	0.4966
rs183906992	**HDL-c**	C	T	0.0294539	0.00474735	5.50E-10	0.0211	0.0282	0.4545
rs1862205	**HDL-c**	A	G	0.0113186	0.00194381	5.80E-09	0.022	0.0287	0.4437
rs188502504	**HDL-c**	C	T	-0.0316669	0.00510538	5.60E-10	-0.0513	0.0997	0.606599
rs1955512	**HDL-c**	A	G	0.0109986	0.00197003	2.40E-08	0.0154	0.0183	0.4016
rs1970811	**HDL-c**	C	T	-0.0116112	0.00191759	1.40E-09	0.0182	0.0166	0.2734
rs201441	**HDL-c**	G	T	-0.0109672	0.0019342	1.40E-08	-0.0186	0.031	0.5477
rs2066714	**HDL-c**	C	T	0.0465396	0.00284369	3.40E-60	-0.013	0.0169	0.4402
rs2068888	**HDL-c**	A	G	0.0191803	0.0019152	1.30E-23	-0.0214	0.0162	0.1868
rs2098368	**HDL-c**	T	C	-0.0116029	0.00192757	1.80E-09	-0.0388	0.017	0.0227997
rs2098918	**HDL-c**	T	C	0.0118698	0.00191595	5.80E-10	-4.00E-04	0.0161	0.982
rs2155220	**HDL-c**	T	C	-0.0105204	0.00191315	3.80E-08	-0.0095	0.0161	0.5569
rs2159607	**HDL-c**	T	G	-0.0239454	0.00243213	7.20E-23	0.0399	0.0479	0.4048
rs2196808	**HDL-c**	C	T	0.0128638	0.00215716	2.50E-09	0.0202	0.017	0.2359
rs2236464	**HDL-c**	C	T	-0.0155836	0.00235595	3.70E-11	0.0048	0.018	0.7915
rs2237035	**HDL-c**	T	G	0.0139172	0.00195824	1.20E-12	0.0014	0.0217	0.9494
rs2247355	**HDL-c**	T	C	0.0206138	0.00245873	5.10E-17	0.0063	0.021	0.764999
rs2256609	**HDL-c**	G	A	-0.0328881	0.00244058	2.20E-41	0.0227	0.017	0.1839
rs2268840	**HDL-c**	C	T	0.0173197	0.00226775	2.20E-14	-0.0047	0.0304	0.8762
rs2281718	**HDL-c**	T	A	0.0596431	0.00195409	1.00E-200	0.106	0.1137	0.3509
rs2290866	**HDL-c**	T	C	-0.0119226	0.00218189	4.60E-08	-0.0137	0.0162	0.397
rs2297409	**HDL-c**	A	G	-0.0333565	0.00240571	1.00E-43	-0.0256	0.0167	0.1253

rs2298214	**HDL-c**	A	C	-0.01241	0.00194099	1.60E-10	-0.0174	0.0193	0.3688
rs2298624	**HDL-c**	T	C	0.0300361	0.00280916	1.10E-26	0.0145	0.0192	0.4493
rs2298632	**HDL-c**	T	C	0.0144252	0.00193744	9.70E-14	-0.0108	0.0161	0.500299
rs2302263	**HDL-c**	T	C	-0.0364373	0.00335268	1.60E-27	-0.005	0.0164	0.762
rs2307111	**HDL-c**	C	T	0.0190053	0.0019521	2.10E-22	-0.0131	0.0159	0.4106
rs2339234	**HDL-c**	A	G	-0.0118996	0.00205717	7.30E-09	0.0037	0.025	0.8829
rs235314	**HDL-c**	T	C	-0.0177425	0.00191993	2.40E-20	0.013	0.0161	0.4174
rs2362541	**HDL-c**	G	T	-0.0108666	0.00190325	1.10E-08	0.03	0.0182	0.0991608
rs2364723	**HDL-c**	C	G	0.0120956	0.00204641	3.40E-09	-0.0064	0.0161	0.691801
rs2417125	**HDL-c**	G	A	-0.0131524	0.00211658	5.20E-10	3.00E-04	0.0164	0.9837
rs2435307	**HDL-c**	T	C	0.0162767	0.00190927	1.50E-17	-0.0349	0.0167	0.0361002
rs2498786	**HDL-c**	G	C	-0.0254741	0.00196826	2.60E-38	-0.0258	0.0193	0.1812
rs2516331	**HDL-c**	A	C	0.0126835	0.00196658	1.10E-10	0.0242	0.0531	0.6484
rs2520096	**HDL-c**	G	A	0.0147205	0.00215114	7.70E-12	-0.0022	0.016	0.8915
rs254562	**HDL-c**	G	A	-0.0114752	0.00194192	3.40E-09	-0.0027	0.0176	0.8771
rs2586116	**HDL-c**	G	C	-0.0162577	0.00217179	7.10E-14	0.0129	0.0559	0.8178
rs2642438	**HDL-c**	G	A	0.0276562	0.00207854	2.10E-40	-0.0112	0.0202	0.580899
rs2645979	**HDL-c**	A	G	0.0114281	0.00198606	8.70E-09	-0.0242	0.0164	0.1399
rs267738	**HDL-c**	G	T	0.0214529	0.00229757	9.90E-21	0.0358	0.0334	0.2835
rs2723065	**HDL-c**	G	A	0.0149623	0.00196239	2.40E-14	1.00E-04	0.0168	0.9974
rs2740488	**HDL-c**	C	A	-0.0686515	0.00216181	1.00E-200	-0.0155	0.0177	0.3821
rs2750411	**HDL-c**	G	T	-0.0108405	0.0019091	1.40E-08	0.0252	0.0161	0.1164
rs2792751	**HDL-c**	C	T	-0.0360998	0.0021336	3.20E-64	-0.0164	0.0186	0.3762
rs2800710	**HDL-c**	C	T	-0.0202674	0.00190382	1.80E-26	-0.0123	0.016	0.4428
rs2804894	**HDL-c**	A	G	0.0173063	0.0021841	2.30E-15	-8.00E-04	0.0172	0.9638
rs2814982	**HDL-c**	T	C	-0.0275938	0.00314209	1.60E-18	0.0367	0.0267	0.1691
rs28362901	**HDL-c**	A	C	-0.0238891	0.00336295	1.20E-12	-0.0058	0.0168	0.729699
rs28510484	**HDL-c**	C	G	-0.0154472	0.00253932	1.20E-09	0.0212	0.0287	0.4602
rs28746806	**HDL-c**	C	A	0.0167023	0.00204471	3.10E-16	0.0436	0.0171	0.01084
rs2910949	**HDL-c**	G	T	0.0133996	0.00199892	2.00E-11	-0.0272	0.0176	0.1232
rs2925979	**HDL-c**	C	T	0.037299	0.00205659	1.60E-73	-0.0106	0.0167	0.5266
rs2943645	**HDL-c**	T	C	-0.0434706	0.00198833	5.90E-106	-0.0216	0.0257	0.3994
rs2963468	**HDL-c**	G	A	-0.0196653	0.00226256	3.60E-18	0.0062	0.0186	0.7383
rs2965169	**HDL-c**	C	A	0.0120831	0.00195448	6.30E-10	0.0073	0.0164	0.6578
rs3027167	**HDL-c**	T	C	-0.0124258	0.00205083	1.40E-09	0.0014	0.0161	0.9321
rs308	**HDL-c**	G	T	0.12651	0.00669305	1.10E-79	0.0232	0.0276	0.3991
rs3184504	**HDL-c**	C	T	0.0265387	0.00190452	3.90E-44	-0.0543	0.0486	0.2634
rs32578	**HDL-c**	A	G	0.0133235	0.00206513	1.10E-10	-0.0104	0.0171	0.5428
rs330089	**HDL-c**	C	T	0.0204692	0.00318925	1.40E-10	3.00E-04	0.0182	0.9885
rs34045894	**HDL-c**	A	G	-0.0172909	0.00262706	4.60E-11	-0.0154	0.0184	0.4007
rs34138141	**HDL-c**	T	G	-0.0175318	0.00212461	1.60E-16	0.0104	0.0172	0.545301
rs343	**HDL-c**	A	C	0.133778	0.00346421	1.00E-200	0.0209	0.0246	0.3957
rs34940374	**HDL-c**	A	G	-0.0170378	0.00247025	5.30E-12	0.0085	0.02	0.672901
rs35493868	**HDL-c**	G	C	0.0372262	0.00237772	3.00E-55	-0.0011	0.0254	0.9639

rs36057735	**HDL-c**	G	C	-0.0302136	0.00238679	1.00E-36	0.0657	0.0328	0.0451502
rs367070	**HDL-c**	G	A	0.0417949	0.00228709	1.30E-74	0.0331	0.0217	0.1262
rs367677	**HDL-c**	G	A	0.0166703	0.00224786	1.20E-13	0.2202	0.1295	0.0889201
rs3732356	**HDL-c**	T	G	-0.0299026	0.00386524	1.00E-14	0.0065	0.036	0.8577
rs3745683	**HDL-c**	A	G	-0.0546353	0.00362928	3.20E-51	-0.0137	0.019	0.4719
rs3746915	**HDL-c**	G	A	0.0108813	0.00194098	2.10E-08	-0.0113	0.0164	0.4929
rs3747973	**HDL-c**	G	A	0.0141665	0.00193802	2.70E-13	-0.0102	0.0492	0.8358
rs3768321	**HDL-c**	T	G	-0.0452296	0.00239482	1.50E-79	-0.0294	0.0221	0.1843
rs3794752	**HDL-c**	C	T	0.0128326	0.00213876	2.00E-09	-0.0164	0.0164	0.3165
rs3814883	**HDL-c**	T	C	-0.0153035	0.00189151	5.90E-16	0.0049	0.0179	0.782699
rs3924313	**HDL-c**	A	G	-0.0239168	0.00204761	1.60E-31	-0.0338	0.0262	0.1982
rs407133	**HDL-c**	C	G	-0.0105618	0.00193239	4.60E-08	-0.0019	0.016	0.9073
rs4074448	**HDL-c**	A	G	0.015131	0.00196355	1.30E-14	0.0428	0.0275	0.1204
rs41272086	**HDL-c**	A	G	-0.0567846	0.00310319	8.50E-75	-0.182	0.0796	0.02225
rs429358	**HDL-c**	C	T	-0.0756554	0.00263871	8.71E-181	-0.0081	0.0259	0.7548
rs454968	**HDL-c**	C	T	0.0110337	0.00199469	3.20E-08	-0.0376	0.0179	0.0357001
rs4599108	**HDL-c**	T	C	0.0136654	0.00192434	1.20E-12	-0.0338	0.0293	0.2481
rs460428	**HDL-c**	C	T	-0.0143462	0.00226779	2.50E-10	0.0115	0.0162	0.4789
rs4614	**HDL-c**	G	A	-0.0178217	0.00193829	3.80E-20	0.0347	0.0234	0.1377
rs4650994	**HDL-c**	A	G	-0.018056	0.00190061	2.10E-21	-0.0219	0.0158	0.1657
rs4691379	**HDL-c**	T	C	0.0118717	0.00204326	6.20E-09	-0.0078	0.0173	0.6511
rs4784709	**HDL-c**	A	T	-0.0747244	0.00477284	3.00E-55	0.0727	0.1615	0.652501
rs4803773	**HDL-c**	G	A	0.0400846	0.00199508	8.70E-90	-0.0218	0.018	0.2251
rs4855582	**HDL-c**	T	C	0.0111652	0.00192369	6.50E-09	0.0286	0.0267	0.2841
rs4871603	**HDL-c**	T	C	0.0360061	0.00199228	5.20E-73	0.0084	0.0162	0.604301
rs4871624	**HDL-c**	G	T	-0.020416	0.00211032	3.90E-22	-0.0164	0.0163	0.315
rs4875043	**HDL-c**	C	A	-0.0149745	0.0023285	1.30E-10	-0.002	0.0219	0.9281
rs4899251	**HDL-c**	T	C	-0.0232048	0.0041399	2.10E-08	-0.0302	0.0199	0.1284
rs4930352	**HDL-c**	T	G	0.0161709	0.00193852	7.30E-17	-0.0069	0.017	0.683
rs4969141	**HDL-c**	T	C	0.0296373	0.00191152	3.20E-54	0.0083	0.0159	0.604399
rs532436	**HDL-c**	A	G	0.0231547	0.00245365	3.80E-21	-0.0159	0.0178	0.3721
rs549058	**HDL-c**	T	G	0.0171095	0.00293694	5.70E-09	0.0116	0.0298	0.6975
rs554146	**HDL-c**	C	A	0.0127257	0.00211531	1.80E-09	0.0026	0.0168	0.8781
rs55781197	**HDL-c**	G	A	0.0587349	0.00295144	4.00E-88	0.0123	0.0402	0.7603
rs557933	**HDL-c**	C	A	0.0152472	0.00190783	1.30E-15	-0.007	0.0173	0.6846
rs55935382	**HDL-c**	A	C	0.01764	0.00203822	4.90E-18	-0.0182	0.0161	0.2577
rs559355	**HDL-c**	T	A	-0.0349311	0.00261254	9.00E-41	0.0012	0.0172	0.9442
rs564832	**HDL-c**	C	T	-0.011708	0.00205455	1.20E-08	-0.0097	0.035	0.7808
rs58123204	**HDL-c**	G	A	-0.0184574	0.00264401	2.90E-12	0.0111	0.0194	0.5681
rs58298943	**HDL-c**	T	C	0.0201986	0.00342748	3.80E-09	-0.0408	0.0268	0.1277
rs59104589	**HDL-c**	T	C	0.0150155	0.0019816	3.50E-14	0.0011	0.0159	0.9471
rs59781045	**HDL-c**	T	C	0.074037	0.0037879	4.50E-85	-0.063	0.0443	0.1544
rs6018652	**HDL-c**	A	G	0.0259271	0.0023605	4.60E-28	-0.0273	0.0175	0.1186
rs6059958	**HDL-c**	T	C	0.0146541	0.00255516	9.70E-09	-0.0827	0.0521	0.1121

rs6066148	**HDL-c**	C	G	0.0133124	0.00218121	1.00E-09	-0.0135	0.0575	0.8146
rs6073958	**HDL-c**	C	T	-0.0609299	0.00239207	4.10E-143	0.0348	0.0249	0.1619
rs6075860	**HDL-c**	A	G	-0.0128	0.00192763	3.10E-11	-0.0379	0.0158	0.0162499
rs6123685	**HDL-c**	A	G	0.0159783	0.0021911	3.00E-13	-0.0064	0.0206	0.7579
rs61352607	**HDL-c**	T	G	0.0306111	0.00222442	4.40E-43	-0.0581	0.027	0.0314297
rs6142206	**HDL-c**	A	G	-0.0161106	0.00193468	8.30E-17	0.036	0.0186	0.05347
rs61435086	**HDL-c**	C	T	0.089343	0.00862169	3.70E-25	0.1328	0.1825	0.467
rs61596977	**HDL-c**	T	C	-0.016225	0.00273721	3.10E-09	-0.0066	0.033	0.8417
rs61748951	**HDL-c**	A	C	-0.0361204	0.00640878	1.70E-08	-0.2309	0.1988	0.2453
rs61805075	**HDL-c**	A	G	-0.0257183	0.00202534	6.00E-37	0.0324	0.0179	0.0702102
rs61884005	**HDL-c**	G	C	0.0163406	0.00293002	2.40E-08	-0.0049	0.0251	0.8439
rs62102718	**HDL-c**	T	A	-0.0236953	0.0021125	3.40E-29	0.0073	0.0212	0.732399
rs62117487	**HDL-c**	G	A	0.0457487	0.00411744	1.10E-28	0.1198	0.1259	0.3413
rs62246443	**HDL-c**	C	T	-0.0147841	0.00250895	3.80E-09	0.0066	0.0197	0.7364
rs62271373	**HDL-c**	A	T	-0.0406667	0.00408672	2.50E-23	0.0636	0.1136	0.5758
rs62331150	**HDL-c**	T	G	-0.0129532	0.00236408	4.30E-08	-0.0015	0.017	0.9296
rs62428831	**HDL-c**	C	T	0.0178151	0.00275536	1.00E-10	-0.0905	0.0628	0.1499
rs635769	**HDL-c**	C	T	0.0197069	0.00196961	1.40E-23	-0.011	0.0179	0.539299
rs6460894	**HDL-c**	C	T	-0.0121653	0.00201452	1.60E-09	-0.0211	0.0166	0.2031
rs6469605	**HDL-c**	T	C	0.0316197	0.00191959	5.80E-61	-0.0099	0.0178	0.5768
rs6693842	**HDL-c**	C	T	0.013664	0.00199336	7.10E-12	0.0899	0.0485	0.0638396
rs6705285	**HDL-c**	T	G	0.0115225	0.00191933	1.90E-09	-0.0296	0.0184	0.1072
rs676210	**HDL-c**	A	G	0.0592694	0.00235237	4.50E-140	0.0164	0.0179	0.3606
rs6762415	**HDL-c**	G	T	-0.0108964	0.00191344	1.20E-08	0.0082	0.017	0.6311
rs6765484	**HDL-c**	T	C	0.0225207	0.00190864	3.90E-32	0.0394	0.0206	0.0557404
rs680321	**HDL-c**	C	T	0.0108517	0.00191658	1.50E-08	0.0233	0.0166	0.1617
rs6824451	**HDL-c**	A	G	-0.0200444	0.00191167	1.00E-25	-0.0012	0.0166	0.94
rs686030	**HDL-c**	A	C	0.0498241	0.00273951	6.50E-74	-0.0167	0.041	0.6848
rs6934962	**HDL-c**	T	C	0.0161905	0.00194099	7.30E-17	-9.00E-04	0.0171	0.9602
rs6939861	**HDL-c**	A	G	-0.0141972	0.00219368	9.70E-11	-0.0116	0.0182	0.523801
rs7036107	**HDL-c**	G	A	-0.0122524	0.00194377	2.90E-10	0.0191	0.0482	0.6915
rs703966	**HDL-c**	A	G	0.0156949	0.00193455	4.90E-16	0.0065	0.0164	0.691
rs7158166	**HDL-c**	C	T	0.0140932	0.00195101	5.10E-13	-0.001	0.0159	0.9495
rs71603401	**HDL-c**	G	A	-0.0154721	0.00279889	3.20E-08	-0.0248	0.0321	0.4392
rs71647892	**HDL-c**	C	T	-0.0629378	0.00871711	5.20E-13	0.7361	0.3325	0.0268201
rs7170463	**HDL-c**	G	A	0.018911	0.0020591	4.20E-20	-0.0037	0.0352	0.9169
rs7186799	**HDL-c**	C	A	-0.0220239	0.00190185	5.20E-31	-0.0079	0.0167	0.635501
rs7218647	**HDL-c**	A	G	0.0109209	0.00192614	1.40E-08	0.0134	0.0163	0.411
rs7251640	**HDL-c**	C	T	0.0141902	0.00242811	5.10E-09	0.0021	0.0203	0.9173
rs72647336	**HDL-c**	A	G	-0.0440494	0.00442834	2.60E-23	0.0875	0.1263	0.488599
rs7281183	**HDL-c**	A	G	-0.01272	0.00218963	6.30E-09	0.0078	0.0199	0.6934
rs72926946	**HDL-c**	A	C	-0.0206565	0.00208041	3.10E-23	-0.1041	0.0381	0.00628304
rs72964564	**HDL-c**	C	A	-0.0125748	0.00220598	1.20E-08	-0.1488	0.0615	0.0154999
rs73151974	**HDL-c**	T	C	-0.0161895	0.00273371	3.20E-09	0.018	0.021	0.3919

rs7316878	**HDL-c**	C	T	0.0115056	0.00201679	1.20E-08	-0.0014	0.0282	0.9604
rs73243877	**HDL-c**	G	A	-0.0253693	0.00254909	2.50E-23	-0.0307	0.0677	0.649999
rs73455693	**HDL-c**	A	G	0.0280646	0.00510538	3.90E-08	0.0908	0.0466	0.0514695
rs74500135	**HDL-c**	C	T	0.0670758	0.010029	2.30E-11	-0.0455	0.3912	0.9074
rs7488780	**HDL-c**	C	G	0.0145459	0.00237104	8.50E-10	-0.0194	0.0317	0.5403
rs75032664	**HDL-c**	G	C	-0.0551041	0.00908791	1.30E-09	-0.058	0.1597	0.7163
rs75152587	**HDL-c**	T	G	-0.0944291	0.00849948	1.10E-28	-0.0074	0.0261	0.776301
rs75479205	**HDL-c**	G	A	0.0138981	0.00243644	1.20E-08	0.0087	0.0457	0.8487
rs75609851	**HDL-c**	A	G	0.170089	0.00964674	1.40E-69	-0.2636	0.2156	0.2216
rs75662196	**HDL-c**	C	G	0.0678721	0.00587239	6.70E-31	-0.3923	0.246	0.1108
rs7583067	**HDL-c**	T	C	0.0145946	0.00223535	6.60E-11	0.0046	0.0163	0.778599
rs7622114	**HDL-c**	A	C	0.0115687	0.00193999	2.50E-09	0.0145	0.0167	0.3861
rs76247316	**HDL-c**	C	T	-0.0113866	0.0019083	2.40E-09	-0.0072	0.019	0.7052
rs76428106	**HDL-c**	C	T	-0.0624148	0.00878921	1.20E-12	-0.1408	0.2246	0.5308
rs76602912	**HDL-c**	C	T	-0.0434476	0.00626364	4.00E-12	0.1881	0.1593	0.2378
rs7665587	**HDL-c**	C	T	0.0139886	0.00193372	4.70E-13	0.0086	0.0173	0.6206
rs76962725	**HDL-c**	A	G	-0.0277897	0.00508558	4.60E-08	-0.0728	0.1692	0.666801
rs771481	**HDL-c**	A	T	0.0288148	0.0024568	9.10E-32	-0.0192	0.0171	0.2616
rs7725218	**HDL-c**	A	G	-0.0122469	0.00201433	1.20E-09	-0.0112	0.0172	0.5142
rs77320712	**HDL-c**	T	G	-0.0127407	0.0022658	1.90E-08	-0.0605	0.0593	0.3076
rs77605964	**HDL-c**	A	G	0.0170396	0.00227819	7.50E-14	-0.0111	0.0263	0.6723
rs77767539	**HDL-c**	A	G	0.0452639	0.00821382	3.60E-08	-0.2869	0.2947	0.3303
rs7794796	**HDL-c**	T	C	-0.016879	0.00203772	1.20E-16	0.0022	0.0174	0.8998
rs77960347	**HDL-c**	G	A	0.290966	0.00830188	1.00E-200	-0.1736	0.2667	0.515101
rs78058190	**HDL-c**	A	G	-0.0783702	0.00490443	1.80E-57	0.1158	0.0981	0.2378
rs7817574	**HDL-c**	C	T	0.0331985	0.00243942	3.50E-42	-0.0351	0.0192	0.0673194
rs7826177	**HDL-c**	C	T	0.0112399	0.00197917	1.40E-08	-0.0168	0.0174	0.3337
rs7853377	**HDL-c**	G	A	0.0152447	0.0023168	4.70E-11	0.0422	0.0588	0.472799
rs79153732	**HDL-c**	T	C	-0.0939759	0.00728238	4.20E-38	0.0209	0.0361	0.5623
rs7924036	**HDL-c**	T	G	0.0138679	0.00190752	3.60E-13	0.0198	0.0169	0.2428
rs79600951	**HDL-c**	G	C	-0.106795	0.00325908	1.00E-200	0.0019	0.0217	0.9312
rs79634051	**HDL-c**	C	G	0.0392148	0.00574877	9.00E-12	-0.0893	0.1357	0.5106
rs80005209	**HDL-c**	G	T	-0.144197	0.00563687	2.50E-144	-0.1308	0.1132	0.2478
rs8014289	**HDL-c**	G	A	0.0151048	0.00192712	4.60E-15	0.0103	0.0159	0.5156
rs8081548	**HDL-c**	A	T	0.01845	0.00202095	6.90E-20	0.009	0.0163	0.5811
rs8086351	**HDL-c**	G	C	0.0839587	0.0025068	1.00E-200	0.0225	0.0256	0.3799
rs830620	**HDL-c**	T	C	0.0149538	0.00193085	9.60E-15	0.006	0.0484	0.9018
rs880674	**HDL-c**	C	T	0.0149836	0.00273775	4.40E-08	0.0206	0.0172	0.2292
rs907866	**HDL-c**	A	G	-0.0183154	0.00191879	1.40E-21	0.0141	0.0169	0.4047
rs921919	**HDL-c**	A	G	-0.041665	0.00206455	1.40E-90	-0.0108	0.0168	0.5196
rs9327468	**HDL-c**	A	C	-0.0148994	0.00221907	1.90E-11	-0.0162	0.0175	0.3536
rs9347737	**HDL-c**	G	A	-0.0134784	0.00194715	4.40E-12	-0.0071	0.0213	0.7387
rs9465693	**HDL-c**	A	C	-0.0121004	0.00208231	6.20E-09	-0.0366	0.0171	0.0321203
rs9604045	**HDL-c**	T	G	0.0175151	0.00228914	2.00E-14	-0.0195	0.0247	0.4313

rs9622830	**HDL-c**	G	C	-0.0163821	0.00199965	2.60E-16	0.0066	0.016	0.6814
rs964184	**HDL-c**	C	G	0.10544	0.00279456	1.00E-200	0.0085	0.0183	0.640801
rs9647335	**HDL-c**	T	A	0.0277595	0.00242471	2.40E-30	-0.0428	0.046	0.3516
rs968050	**HDL-c**	T	C	0.0136601	0.00190807	8.10E-13	0.0316	0.0163	0.0528896
rs983663	**HDL-c**	G	A	-0.0143608	0.0022075	7.70E-11	9.00E-04	0.0162	0.9559
rs9904004	**HDL-c**	G	A	-0.0302694	0.00400552	4.10E-14	-0.2612	0.2318	0.2598
rs9916613	**HDL-c**	A	T	-0.0133119	0.00199143	2.30E-11	0.0182	0.0194	0.3462
rs9933509	**HDL-c**	C	T	-0.0140121	0.00191313	2.40E-13	-0.0109	0.0183	0.552699
rs998584	**HDL-c**	A	C	-0.0341901	0.00190975	1.10E-71	-0.0047	0.0158	0.7662
rs9987289	**HDL-c**	G	A	0.0872902	0.00329366	9.10E-155	-0.0276	0.0536	0.6062
rs9989419	**HDL-c**	G	A	0.143765	0.00192633	1.00E-200	0.0093	0.0197	0.6373

HDL-c: HDL cholesterol. EA: effect allele. OA: other allele. GX: beta-exposure. GX(SE): standard error of GX. GY: beta-outcome. GY(SE): standard error of GY.

Table S17. Published associations of Sex hormone-binding globulin levels on PTB

SNP	Exposure	EA	OA	GX	GX(SE)	Pval-exp	F	GY	GY(SE)	Pval-outcome
rs10141867	**SHBG**	A	G	0.016761	0.00224676	3.90E-16	55.65245124	-0.0146	0.0236	0.5369
rs10210970	**SHBG**	T	C	-0.0217591	0.00295905	1.80E-14	54.07232718	0.0166	0.0206	0.4211
rs1042852	**SHBG**	T	C	-0.0190768	0.00225239	2.40E-19	71.73344437	0.0133	0.0186	0.476
rs1047743	**SHBG**	G	C	0.0262627	0.00208159	4.00E-36	159.1791466	0.0068	0.016	0.670101
rs1047891	**SHBG**	A	C	0.0220665	0.00219589	5.10E-25	100.981905	-0.0375	0.0219	0.0874299
rs1047912	**SHBG**	T	C	0.0229516	0.00218222	1.20E-26	110.6182241	0.0395	0.0202	0.0509096
rs10513272	**SHBG**	T	C	-0.0184094	0.00238344	9.80E-18	59.65799227	-0.01	0.058	0.8633
rs10737153	**SHBG**	C	A	-0.032626	0.0020279	9.60E-65	258.8406046	-0.0187	0.0159	0.2405
rs10752297	**SHBG**	C	T	0.0176861	0.00204471	8.00E-21	74.81669559	-0.0117	0.0173	0.5012
rs10753556	**SHBG**	G	A	0.0219941	0.00301694	5.40E-16	53.14676772	-0.014	0.0177	0.4289
rs1075403	**SHBG**	G	T	0.0168553	0.00208512	1.60E-17	65.34444564	0.0302	0.0191	0.1129
rs10760112	**SHBG**	T	C	-0.0182743	0.00218422	1.80E-20	69.99814869	0.0105	0.016	0.5112
rs10898075	**SHBG**	T	C	-0.0171546	0.0026827	2.30E-09	40.8897811	0.0748	0.0685	0.2749
rs11010551	**SHBG**	C	T	-0.0123375	0.00206095	2.90E-08	35.83581059	-0.0467	0.0164	0.00430398
rs11021219	**SHBG**	A	G	0.0181988	0.00287479	2.90E-10	40.07477252	0.0016	0.0223	0.9412
rs11038642	**SHBG**	T	C	0.0207903	0.00288836	2.00E-11	51.81036845	0.0384	0.0231	0.0966095
rs11042751	**SHBG**	C	T	-0.0218119	0.00234698	2.10E-23	86.37061873	-0.0311	0.0305	0.3073
rs1106317	**SHBG**	G	A	-0.0160852	0.00246463	2.80E-09	42.59388772	-0.019	0.0164	0.2484
rs11075253	**SHBG**	A	C	0.0239889	0.0022068	4.20E-30	118.1659987	0.037	0.053	0.485801
rs11089620	**SHBG**	G	C	0.0229192	0.00263806	3.10E-19	75.47921649	0.0227	0.0171	0.1862
rs11110390	**SHBG**	T	C	-0.0219349	0.00212453	3.00E-26	106.5966101	0.0082	0.0291	0.7783
rs11111274	**SHBG**	A	G	-0.0252202	0.00226355	3.90E-30	124.1408402	3.00E-04	0.016	0.9859

rs111912536	**SHBG**	T	C	0.0270849	0.00337573	8.90E-17	64.37494614	-0.0612	0.0942	0.5161
rs11191580	**SHBG**	C	T	0.0268242	0.00374811	1.60E-12	51.21848321	-0.0253	0.0186	0.173
rs112035922	**SHBG**	T	C	-0.0203792	0.0023916	1.80E-18	72.60975741	-0.0095	0.0206	0.644899
rs1120608	**SHBG**	C	T	0.0246132	0.00414719	5.80E-10	35.22298099	0.0074	0.033	0.8222
rs112134815	**SHBG**	A	C	-0.0226216	0.00368141	3.10E-08	37.75861664	-0.0653	0.0938	0.4867
rs1126670	**SHBG**	A	C	-0.0331858	0.00218859	4.00E-58	229.9183183	-0.065	0.0505	0.1976
rs1128249	**SHBG**	T	G	0.039171	0.00204115	9.40E-93	368.2792817	-0.0632	0.0262	0.0159599
rs1128919	**SHBG**	A	G	0.0137703	0.00217712	1.90E-11	40.00551645	-0.0171	0.0159	0.2805
rs1132368	**SHBG**	A	G	-0.0282631	0.00515841	1.20E-08	30.01965433	0.0526	0.0536	0.3265
rs1135989	**SHBG**	A	G	-0.0194175	0.00197704	2.20E-25	96.46139264	0.0056	0.0485	0.9074
rs114165349	**SHBG**	C	G	-0.203771	0.00659885	1.00E-200	953.5556702	0.0272	0.1301	0.8345
rs115447786	**SHBG**	T	C	-0.0430159	0.0048712	7.00E-19	77.98012338	-0.0436	0.1469	0.7668
rs11550348	**SHBG**	A	G	0.0632857	0.00306245	1.40E-102	427.0423783	0.0455	0.0816	0.577
rs11556184	**SHBG**	T	C	-0.0482257	0.00405756	1.40E-33	141.2618832	0.0121	0.0214	0.5721
rs11556924	**SHBG**	T	C	0.017144	0.00208606	1.30E-17	67.54116342	0.0317	0.0372	0.3936
rs11581220	**SHBG**	T	G	0.0251411	0.00309001	5.60E-19	66.19826293	0.0278	0.0494	0.5742
rs11600570	**SHBG**	A	G	-0.016901	0.00309223	5.40E-09	29.87301365	-0.0898	0.0775	0.2469
rs11635545	**SHBG**	T	G	-0.0207172	0.00249259	2.10E-19	69.08093913	-0.0292	0.0238	0.2196
rs11636917	**SHBG**	C	T	-0.034928	0.0021139	7.30E-70	273.0086275	0.0019	0.0482	0.9682
rs11639856	**SHBG**	A	T	-0.013474	0.00254817	1.10E-08	27.95980265	0.0227	0.0272	0.4036
rs11647008	**SHBG**	C	T	-0.025954	0.002018	3.20E-41	165.4108865	0.0013	0.0275	0.9616
rs11655704	**SHBG**	C	T	0.0782784	0.00207191	1.00E-200	1427.381027	0.0126	0.0191	0.5109
rs11657929	**SHBG**	G	T	-0.0202742	0.00193696	9.40E-28	109.557966	-0.0025	0.0198	0.9013
rs11664106	**SHBG**	T	A	0.0176965	0.00212962	2.00E-16	69.0507404	0.0386	0.0515	0.454
rs11737560	**SHBG**	T	C	-0.0254556	0.00303264	5.80E-19	70.45677663	0.0117	0.0337	0.7294
rs117411982	**SHBG**	A	C	0.0570171	0.00812849	8.90E-14	49.20262912	-0.1427	0.1759	0.417
rs117572361	**SHBG**	G	A	0.0362894	0.00631868	1.40E-08	32.98410197	0.1309	0.1275	0.3043
rs11806197	**SHBG**	G	A	0.0190526	0.00253431	2.20E-12	56.5180068	0.0042	0.0201	0.8347
rs11812460	**SHBG**	A	G	0.0240074	0.00228975	4.80E-26	109.9289131	-0.0279	0.0168	0.0971091
rs11853116	**SHBG**	T	C	-0.0149796	0.00214706	6.80E-15	48.67543971	-6.00E-04	0.016	0.9689
rs11883596	**SHBG**	C	T	0.0213762	0.00330019	1.20E-10	41.95473021	0.061	0.0892	0.4937
rs11887329	**SHBG**	G	A	-0.025052	0.00235515	1.50E-28	113.147633	0.0111	0.0181	0.5373
rs11917105	**SHBG**	G	T	-0.0143788	0.0022824	1.80E-10	39.68803354	0.001	0.0161	0.9505
rs11979093	**SHBG**	A	G	-0.0195978	0.00274327	5.10E-14	51.03584933	0.0623	0.0309	0.0437895
rs1211184	**SHBG**	C	T	-0.0261095	0.00342644	1.30E-15	58.06422849	-0.0485	0.0198	0.0142801
rs1223801	**SHBG**	A	G	-0.0382092	0.00270101	4.80E-50	200.1157716	0.0145	0.0186	0.4365
rs1229502	**SHBG**	A	G	-0.0124625	0.00220108	1.90E-08	32.05800555	-0.0298	0.017	0.0788098
rs12300756	**SHBG**	T	C	-0.0165978	0.00237925	4.80E-13	48.6652222	0.0127	0.0159	0.4243
rs12414178	**SHBG**	T	C	-0.0297615	0.00235674	4.20E-38	159.4718743	0.0058	0.0173	0.737099
rs12454712	**SHBG**	C	T	0.0235395	0.00209522	1.60E-31	126.2214073	-0.0013	0.0161	0.9375
rs12569576	**SHBG**	G	A	-0.0162183	0.00200739	5.90E-17	65.27471117	-0.0078	0.0176	0.656299
rs1260326	**SHBG**	C	T	0.093048	0.00206023	1.00E-200	2039.766637	0.0031	0.0159	0.8472
rs1262217	**SHBG**	A	G	0.0176115	0.00263197	2.70E-12	44.7742827	-0.0481	0.0786	0.5407
rs12888084	**SHBG**	G	A	0.0130136	0.00202027	5.50E-13	41.49290962	0.0175	0.0207	0.3978

rs12897338	**SHBG**	T	C	-0.0240161	0.00201414	6.00E-36	142.1750763	0.0201	0.0199	0.3122
rs12915708	**SHBG**	C	G	0.0172427	0.0021821	1.30E-15	62.43944843	0.0031	0.0161	0.8495
rs1293940	**SHBG**	C	T	0.0123282	0.00201229	4.60E-11	37.53323664	-0.0047	0.0159	0.7691
rs12945299	**SHBG**	G	A	0.0537969	0.00245018	3.10E-113	482.0769119	-0.0127	0.1384	0.9269
rs12967290	**SHBG**	T	C	-0.0137722	0.00220487	7.90E-10	39.01561683	-0.0326	0.016	0.0421502
rs12983990	**SHBG**	G	A	0.0159043	0.00273091	9.10E-10	33.91658794	0.0445	0.024	0.0632907
rs13011791	**SHBG**	A	T	0.0142413	0.00251494	1.10E-09	32.06578128	-0.0438	0.0528	0.407
rs13094241	**SHBG**	G	T	0.0116941	0.00225279	2.40E-08	26.94573285	0.0055	0.0181	0.7597
rs13155267	**SHBG**	C	T	0.0141252	0.00224292	5.90E-10	39.66061614	-0.0035	0.0162	0.8295
rs1326122	**SHBG**	A	C	0.0610776	0.0070269	5.00E-22	75.54995204	0.0605	0.1641	0.7125
rs1326775	**SHBG**	T	C	-0.0173656	0.00269701	3.00E-12	41.45837594	0.0194	0.0162	0.2298
rs13289095	**SHBG**	T	G	-0.039195	0.00287357	2.20E-47	186.0439655	0.0016	0.031	0.9577
rs138373837	**SHBG**	T	C	0.0392943	0.00672672	2.00E-10	34.12323714	-0.1235	0.2214	0.576901
rs138840	**SHBG**	T	C	-0.021466	0.00327484	3.50E-12	42.96550193	-0.0103	0.0293	0.7248
rs138924453	**SHBG**	T	C	0.0749929	0.0137966	1.70E-09	29.54567815	-0.8014	0.5445	0.1411
rs1421085	**SHBG**	C	T	0.0117148	0.00205432	4.10E-08	32.51856562	-0.021	0.0191	0.2724
rs144725072	**SHBG**	A	G	-0.043009	0.00593224	2.30E-15	52.56287074	-0.0446	0.1032	0.666
rs144867634	**SHBG**	C	T	-0.0379318	0.00658994	1.40E-08	33.13155316	5.00E-04	0.1705	0.9975
rs1474419	**SHBG**	C	T	0.0166644	0.00204805	6.00E-15	66.2058042	-0.0749	0.0305	0.0140401
rs1497406	**SHBG**	G	A	0.0265373	0.00201543	3.40E-43	173.370756	-8.00E-04	0.0195	0.9692
rs150539196	**SHBG**	G	A	0.0957998	0.00514186	5.60E-86	347.1255027	0.0519	0.0337	0.1231
rs152503	**SHBG**	A	C	-0.014843	0.00249813	8.30E-10	35.30295975	0.0191	0.0162	0.2363
rs1529868	**SHBG**	T	C	0.0140188	0.0020619	1.60E-14	46.22578499	-1.00E-04	0.016	0.9974
rs1531343	**SHBG**	C	G	-0.0207639	0.00331913	9.90E-10	39.13516275	0.0032	0.0238	0.8918
rs1534696	**SHBG**	A	C	0.0125088	0.00201372	4.20E-11	38.58610511	0.0451	0.0191	0.01852
rs1547014	**SHBG**	C	T	-0.0305309	0.00215895	1.60E-48	199.9824373	0.0278	0.023	0.2278
rs1556562	**SHBG**	T	G	0.0219499	0.00241751	1.60E-20	82.437791	-0.0401	0.0455	0.3782
rs1572053	**SHBG**	G	A	0.0141236	0.00207742	2.70E-13	46.22107202	-0.0162	0.0159	0.3079
rs1573486	**SHBG**	A	G	-0.0203082	0.00216664	1.40E-20	87.85513744	0.0199	0.0159	0.2102
rs1574525	**SHBG**	G	A	0.0299925	0.00215368	5.20E-46	193.9370554	0.0189	0.0196	0.3359
rs157934	**SHBG**	C	T	0.027442	0.00218342	1.40E-39	157.9627785	0.0326	0.0161	0.0429398
rs161941	**SHBG**	T	C	-0.0119501	0.00222569	2.20E-08	28.82780279	0.0124	0.0269	0.645
rs1661714	**SHBG**	T	C	-0.0156502	0.0022018	4.20E-13	50.52215436	0.0375	0.0242	0.1209
rs16845803	**SHBG**	G	A	-0.0290544	0.00297538	2.30E-26	95.35352577	0.0238	0.0234	0.3095
rs16925010	**SHBG**	T	C	-0.0138089	0.00225324	1.90E-09	37.5578806	0.0186	0.0168	0.2695
rs17036326	**SHBG**	G	A	0.0487484	0.00307476	3.70E-59	251.3599603	0.0066	0.0388	0.8659
rs17123214	**SHBG**	T	C	-0.0386069	0.00751972	3.70E-08	26.35872106	-0.022	0.0441	0.6172
rs17206359	**SHBG**	G	A	0.0197834	0.0021425	5.10E-21	85.26249073	-0.0177	0.0166	0.2848
rs1736176	**SHBG**	A	G	0.0482439	0.00224745	6.90E-111	460.7894768	0.0065	0.0163	0.690601
rs17377148	**SHBG**	G	T	0.0474977	0.00381931	5.90E-39	154.6582717	0.069	0.0904	0.4456
rs1741344	**SHBG**	T	C	0.0117683	0.00209206	7.10E-10	31.64295274	-0.012	0.0204	0.5579
rs1755618	**SHBG**	T	G	-0.0292948	0.00289779	1.40E-26	102.1986287	0.0051	0.0247	0.8362
rs17580	**SHBG**	A	T	0.0623089	0.00479246	1.50E-43	169.0366929	0.188	0.1921	0.3278
rs17630640	**SHBG**	G	A	0.0238144	0.00297687	2.20E-18	63.99666779	-0.0168	0.0239	0.4837

rs1772183	**SHBG**	A	G	-0.029011	0.00199798	2.40E-53	210.8341403	0.0048	0.0171	0.7804
rs17789218	**SHBG**	C	T	0.0165555	0.0023425	1.20E-14	49.94858809	0.0541	0.0586	0.3561
rs17790938	**SHBG**	A	G	0.042401	0.00391065	4.50E-28	117.5579796	-0.009	0.0312	0.7728
rs17855876	**SHBG**	A	G	-0.0266222	0.00330949	1.90E-16	64.70883032	-0.0271	0.024	0.2601
rs1801689	**SHBG**	C	A	-0.0924133	0.00564719	5.30E-64	267.7946955	0.0477	0.2132	0.8228
rs1832007	**SHBG**	G	A	-0.0509179	0.00277142	1.60E-86	337.546871	-0.0378	0.0277	0.1725
rs1894644	**SHBG**	C	T	-0.0191015	0.00288898	1.00E-12	43.71632917	0.0467	0.0779	0.549
rs1902023	**SHBG**	C	A	0.0341097	0.0020239	5.70E-69	284.0373885	-6.00E-04	0.0177	0.9724
rs1920149	**SHBG**	A	G	-0.0169788	0.00201546	1.50E-16	70.96814131	0.0114	0.016	0.4758
rs1931788	**SHBG**	A	G	-0.0134537	0.00226182	3.60E-10	35.38060141	-0.0208	0.0172	0.2245
rs1975937	**SHBG**	T	A	-0.0170651	0.002664	8.00E-11	41.03430163	0.0122	0.0172	0.4785
rs1982151	**SHBG**	G	A	-0.0560448	0.00231676	2.40E-134	585.202762	0.0019	0.0181	0.9155
rs2021965	**SHBG**	A	C	-0.0126946	0.00223533	1.70E-11	32.25169625	0.0044	0.0175	0.802
rs202200760	**SHBG**	C	G	0.172316	0.00479921	1.00E-200	1289.167992	-0.2127	0.1098	0.0526502
rs2054989	**SHBG**	G	A	0.0139811	0.00233005	3.40E-11	36.00393884	-6.00E-04	0.0163	0.9719
rs2074518	**SHBG**	T	C	-0.0113521	0.0019353	2.60E-08	34.40754095	-0.0158	0.0194	0.4176
rs2075025	**SHBG**	C	T	0.0191728	0.00210162	4.70E-20	83.22628528	0.0056	0.0213	0.793999
rs2076847	**SHBG**	G	A	0.0115573	0.00193469	8.70E-09	35.68517344	-0.0371	0.016	0.0201902
rs2081687	**SHBG**	C	T	0.0238137	0.00212643	2.30E-30	125.4149725	-0.0174	0.0205	0.3949
rs2205262	**SHBG**	A	C	-0.0175324	0.00203217	1.70E-20	74.43213591	-0.0081	0.0157	0.6059
rs2227947	**SHBG**	A	G	0.0114019	0.00208682	2.30E-09	29.85260861	0.0091	0.017	0.5942
rs2229357	**SHBG**	A	G	0.0426912	0.00235392	8.30E-79	328.9206498	-0.0576	0.027	0.0329997
rs2230316	**SHBG**	A	G	0.0338916	0.00201031	3.50E-66	284.2208216	-0.0321	0.0269	0.2339
rs2232015	**SHBG**	T	A	0.0615313	0.00211316	1.00E-200	847.862117	-0.0034	0.0174	0.8443
rs2274159	**SHBG**	G	A	0.0168735	0.00201958	1.80E-18	69.80492127	0.0011	0.0158	0.9441
rs2275516	**SHBG**	T	G	-0.0195958	0.00277986	7.50E-13	49.69102555	-0.0479	0.0338	0.1568
rs2276161	**SHBG**	T	A	-0.0161495	0.00296172	7.40E-09	29.73226383	-0.0052	0.0181	0.7755
rs2280552	**SHBG**	T	C	0.0172476	0.00208379	4.30E-19	68.50894135	0.001	0.0165	0.9533
rs2282377	**SHBG**	G	C	-0.0167537	0.0029793	2.30E-09	31.62210731	-0.0185	0.0257	0.4708
rs2283371	**SHBG**	G	A	0.0205635	0.00213439	7.90E-25	92.82059079	0.0014	0.016	0.9326
rs2286973	**SHBG**	A	G	-0.0124092	0.00205266	2.10E-11	36.54696708	-0.0029	0.017	0.8671
rs2288004	**SHBG**	C	G	-0.0199226	0.00207283	3.90E-23	92.37670602	-0.015	0.0249	0.5474
rs2289850	**SHBG**	C	T	0.0255774	0.00449043	1.10E-09	32.44402607	0.0114	0.02	0.5665
rs2290545	**SHBG**	A	G	-0.0135753	0.00227879	8.70E-11	35.4885352	0.0044	0.0162	0.7861
rs2291542	**SHBG**	T	C	-0.02274	0.00215885	8.90E-25	110.9516582	-0.0024	0.0185	0.8952
rs2293476	**SHBG**	C	G	-0.0297214	0.00238067	4.00E-38	155.8611726	-0.0272	0.0218	0.2136
rs2294613	**SHBG**	T	C	0.0208506	0.00330856	2.10E-12	39.71521301	-0.0038	0.0352	0.9141
rs2298650	**SHBG**	T	G	0.0161035	0.00228536	1.70E-14	49.65112852	-0.0098	0.0159	0.539499
rs2302370	**SHBG**	C	T	-0.0154123	0.00205818	3.30E-12	56.07457786	-0.0252	0.017	0.1368
rs2305833	**SHBG**	C	G	-0.0194934	0.00204176	4.30E-23	91.15145778	-0.0067	0.0179	0.7094
rs2306374	**SHBG**	C	T	-0.0191268	0.00273298	2.00E-11	48.97895768	-0.0186	0.0277	0.501601
rs2306700	**SHBG**	T	C	0.0176883	0.00294848	1.10E-10	35.98931915	0.0092	0.0233	0.691801
rs2383208	**SHBG**	G	A	0.0151754	0.00262746	3.60E-08	33.35845135	-0.0303	0.0165	0.0666807
rs2522056	**SHBG**	A	G	0.0232787	0.00257522	6.20E-19	81.71214029	-0.0136	0.0164	0.4086

rs2529123	**SHBG**	G	A	0.0171725	0.00206382	7.00E-17	69.23428777	-0.0203	0.0205	0.3211
rs2538	**SHBG**	T	C	-0.0122141	0.00221981	1.10E-08	30.27534829	0.0035	0.0203	0.8622
rs2567426	**SHBG**	G	A	-0.019739	0.00209385	2.10E-23	88.87036704	-0.0392	0.0219	0.0732892
rs2618566	**SHBG**	T	G	-0.0189622	0.00212868	2.70E-20	79.35138268	0.0107	0.0176	0.5434
rs267733	**SHBG**	G	A	-0.0300164	0.0027355	1.30E-30	120.404185	0.0483	0.0346	0.163
rs2738755	**SHBG**	T	C	-0.0296824	0.00212657	1.30E-46	194.8213163	0.0271	0.0211	0.2008
rs2820436	**SHBG**	C	A	-0.0252458	0.00210129	5.80E-34	144.3457827	-0.0121	0.0502	0.809
rs2823662	**SHBG**	G	A	0.0103478	0.00204475	2.30E-08	25.61022718	0.0273	0.0406	0.5008
rs2836934	**SHBG**	C	A	-0.0114984	0.00205957	1.20E-08	31.16875987	-0.0277	0.0159	0.0813205
rs2862954	**SHBG**	C	T	0.0250193	0.00200372	3.00E-34	155.9100301	0.0041	0.0317	0.8975
rs28656215	**SHBG**	C	T	-0.0246866	0.00385523	1.30E-09	41.00339218	0.033	0.0292	0.2592
rs2889537	**SHBG**	A	G	0.0131045	0.00212271	5.40E-11	38.11161837	0.0207	0.0179	0.2482
rs28925904	**SHBG**	T	C	-0.0541666	0.00652533	5.10E-17	68.90583581	-0.3173	0.29	0.2739
rs28929474	**SHBG**	T	C	0.237363	0.0072706	1.00E-200	1065.818742	0.2462	0.1735	0.156
rs2893236	**SHBG**	T	C	0.0142249	0.00241628	4.60E-11	34.6578596	0.0231	0.0193	0.2317
rs2925979	**SHBG**	C	T	0.0161725	0.00219806	4.50E-15	54.13436487	-0.0106	0.0167	0.5266
rs2943641	**SHBG**	C	T	-0.0339261	0.00208577	4.50E-65	264.5652834	-0.0205	0.0257	0.4241
rs2971677	**SHBG**	A	C	-0.0226496	0.00277427	3.30E-16	66.65326597	0.0248	0.0175	0.1583
rs310786	**SHBG**	T	C	0.0174514	0.00287758	1.90E-10	36.77930731	0.0058	0.0163	0.7205
rs3110039	**SHBG**	G	T	-0.0109842	0.00205756	4.90E-08	28.49900249	-0.0063	0.016	0.6955
rs314373	**SHBG**	A	G	0.0401169	0.00257537	7.50E-61	242.6460799	0.047	0.0438	0.2833
rs329120	**SHBG**	T	C	-0.0220426	0.00204194	4.90E-28	116.5299334	0.0182	0.0163	0.2633
rs340874	**SHBG**	C	T	-0.015938	0.00201247	1.90E-17	62.72008341	0.0073	0.0162	0.653501
rs34145453	**SHBG**	G	A	0.0207587	0.00208394	1.10E-25	99.22650531	0.0235	0.0161	0.145
rs34372369	**SHBG**	A	G	0.0414611	0.0046998	3.30E-18	77.82528124	-0.0461	0.0927	0.618899
rs34481144	**SHBG**	T	C	0.0132406	0.00204576	3.20E-11	41.88936512	-0.0659	0.0487	0.1763
rs34651	**SHBG**	T	C	0.0294153	0.00368137	6.20E-19	63.84478645	0.0013	0.0234	0.9543
rs34691116	**SHBG**	T	C	0.0240702	0.00282018	9.60E-20	72.84563834	-0.0366	0.0753	0.6272
rs34930209	**SHBG**	G	A	0.0322481	0.00337937	1.70E-22	91.06145544	-0.0351	0.0254	0.167
rs35171419	**SHBG**	T	C	0.0285717	0.0051442	7.80E-09	30.8485166	0.2034	0.1795	0.2571
rs35313240	**SHBG**	T	C	0.0640052	0.0113094	2.80E-09	32.02942931	-0.6281	0.4552	0.1677
rs35350976	**SHBG**	G	A	0.019582	0.00266383	1.00E-15	54.03795354	-0.0084	0.0336	0.8034
rs35497030	**SHBG**	G	A	-0.0191423	0.00202178	9.70E-24	89.64338691	-0.0193	0.0173	0.263
rs35756394	**SHBG**	G	A	-0.0325415	0.00372071	2.50E-19	76.4929315	-0.0045	0.0244	0.8549
rs35759537	**SHBG**	T	C	0.0285333	0.00253836	1.40E-32	126.3558596	0.0326	0.0206	0.1141
rs35932591	**SHBG**	T	C	-0.0171697	0.00305472	1.50E-09	31.59223924	0.0093	0.0188	0.6208
rs35955110	**SHBG**	C	T	0.011383	0.0021568	6.70E-10	27.85425418	-0.0057	0.0164	0.7293
rs35981831	**SHBG**	G	T	-0.0429854	0.00625804	2.60E-12	47.1805596	0.1527	0.162	0.3458
rs3729931	**SHBG**	A	G	0.0208046	0.00209369	2.60E-24	98.73969817	0.0068	0.0274	0.8048
rs3733322	**SHBG**	G	T	0.0170019	0.00208141	1.50E-14	66.7232902	0.0617	0.1142	0.589
rs3741367	**SHBG**	C	T	0.0138401	0.00203244	4.20E-12	46.37039564	0.001	0.0185	0.958
rs3741378	**SHBG**	T	C	0.0315481	0.00292661	4.20E-32	116.2022468	0.0023	0.0167	0.8916
rs3743588	**SHBG**	A	G	-0.0200523	0.00225557	2.00E-21	79.03393034	0.0087	0.0168	0.6051
rs3746575	**SHBG**	G	C	0.0426511	0.00208996	2.20E-97	416.4686708	0.0032	0.0164	0.8433

rs3752293	**SHBG**	T	C	0.0172424	0.00219928	2.00E-16	61.46560979	0.0161	0.0166	0.3323
rs3754186	**SHBG**	A	T	-0.0139399	0.00200815	4.40E-14	48.18643865	0.0198	0.0163	0.2246
rs3760620	**SHBG**	C	T	-0.0195657	0.00245997	8.00E-16	63.25996848	-0.044	0.0248	0.0759504
rs3761706	**SHBG**	A	G	-0.044684	0.00411507	2.70E-28	117.9091325	-0.0379	0.0216	0.0791407
rs3779195	**SHBG**	A	T	-0.0820929	0.00258425	1.00E-200	1009.113365	0.0181	0.0178	0.3092
rs3790414	**SHBG**	A	T	-0.0243155	0.00236881	5.00E-24	105.3667939	0.0363	0.0172	0.0347096
rs3824627	**SHBG**	C	T	-0.014287	0.00207173	8.30E-12	47.55690597	-0.0123	0.0164	0.4555
rs38324	**SHBG**	T	C	-0.0176008	0.00261903	4.70E-10	45.16288555	-0.013	0.02	0.517201
rs3848375	**SHBG**	T	C	0.0204783	0.00254585	3.60E-17	64.70234051	-0.0174	0.0285	0.5408
rs3858420	**SHBG**	C	G	-0.0217872	0.0021784	9.10E-27	100.0288776	-0.0068	0.0159	0.6709
rs3912391	**SHBG**	A	G	-0.0130203	0.00200341	1.10E-13	42.23768624	5.00E-04	0.0172	0.975
rs3934712	**SHBG**	C	T	-0.0227515	0.00245386	1.00E-20	85.96433728	0.0134	0.0179	0.4548
rs4026608	**SHBG**	T	C	0.0136349	0.00205191	1.30E-12	44.15553087	0.0025	0.018	0.8891
rs41272717	**SHBG**	A	G	0.015942	0.00296444	3.50E-08	28.91998694	0.0024	0.0499	0.9614
rs41305481	**SHBG**	G	A	0.011961	0.00221838	4.40E-08	29.0710629	0.0011	0.0193	0.9559
rs4132228	**SHBG**	T	C	0.0128968	0.00221411	1.60E-08	33.92839811	-0.0218	0.0164	0.1839
rs4148646	**SHBG**	G	C	0.0127167	0.00211165	4.10E-10	36.26626167	-0.0085	0.0162	0.6008
rs4149056	**SHBG**	C	T	-0.0776477	0.0028298	1.60E-174	752.910788	0.0199	0.0219	0.365
rs41736	**SHBG**	T	C	-0.0153829	0.00202257	1.90E-15	57.8451729	0.0138	0.0158	0.3812
rs4246215	**SHBG**	T	G	-0.0229858	0.00209443	3.70E-29	120.4440525	0.0422	0.0162	0.00915693
rs4259246	**SHBG**	T	C	0.0288573	0.00214669	3.70E-44	180.705152	0.0271	0.0169	0.1091
rs4291201	**SHBG**	G	A	0.0191056	0.00220768	2.80E-21	74.89398301	-0.0109	0.0195	0.5745
rs4388292	**SHBG**	G	T	0.018337	0.00281807	1.80E-09	42.3399976	-0.0167	0.0172	0.3327
rs440837	**SHBG**	G	A	0.0457672	0.00249001	2.10E-79	337.8347479	0.04	0.0226	0.0764892
rs445	**SHBG**	T	C	-0.0284671	0.00340323	1.90E-17	69.96835514	0.0062	0.0177	0.726599
rs4568225	**SHBG**	T	C	0.0171155	0.00215964	5.70E-16	62.80789938	0.0093	0.0409	0.8206
rs459193	**SHBG**	G	A	-0.0362628	0.00230682	5.30E-59	247.1115619	-0.0128	0.0159	0.421
rs4633399	**SHBG**	C	A	0.0183833	0.00346168	3.10E-08	28.20141657	0.0151	0.017	0.3747
rs4711750	**SHBG**	A	T	-0.0220559	0.0020117	4.50E-30	120.2045615	-0.0043	0.016	0.7901
rs4765127	**SHBG**	T	G	0.0231284	0.00211913	1.60E-30	119.1170124	-0.0249	0.0218	0.253
rs4804416	**SHBG**	G	T	-0.0367496	0.00202964	1.20E-79	327.842323	0.0193	0.016	0.2281
rs4804833	**SHBG**	G	A	0.0143069	0.00210978	3.30E-12	45.98482663	-6.00E-04	0.0159	0.969
rs4806073	**SHBG**	C	T	0.0742675	0.00400946	5.20E-84	343.1023178	0.0038	0.0297	0.8989
rs4809221	**SHBG**	A	G	-0.0148579	0.00216838	7.00E-14	46.95068021	-0.0034	0.0164	0.8367
rs4841132	**SHBG**	G	A	0.06237	0.00371042	1.80E-72	282.5551035	-0.0277	0.0536	0.6056
rs4858608	**SHBG**	G	T	-0.0196274	0.0020112	1.50E-24	95.23856396	0.0059	0.0158	0.7099
rs4907236	**SHBG**	T	C	0.0138626	0.0022177	2.00E-11	39.073436	0.0152	0.0162	0.3507
rs4925809	**SHBG**	T	C	0.0246432	0.00201251	1.10E-39	149.9394529	0.0038	0.016	0.8132
rs4950979	**SHBG**	A	G	-0.0188233	0.00291714	2.00E-11	41.63655563	-0.0448	0.0382	0.2406
rs543874	**SHBG**	G	A	0.0240665	0.00246852	7.30E-23	95.04961945	-0.0316	0.0191	0.09846
rs555754	**SHBG**	A	G	0.0421637	0.00202293	1.70E-109	434.4237214	0.0185	0.0173	0.2849
rs55662831	**SHBG**	A	G	0.0971291	0.00200002	1.00E-200	2358.456467	-0.0264	0.0502	0.5986
rs55707100	**SHBG**	T	C	-0.152947	0.00637931	1.00E-143	574.8206005	0.3604	0.257	0.1608
rs55744465	**SHBG**	T	G	-0.0124679	0.00215304	2.50E-09	33.53360437	0.0144	0.016	0.367

rs55770587	**SHBG**	T	C	0.0288607	0.00502895	1.90E-08	32.93494161	0.1142	0.1261	0.3653
rs55840085	**SHBG**	A	G	0.0202749	0.00209775	5.40E-27	93.4131021	0.0068	0.0163	0.675199
rs56033865	**SHBG**	A	G	0.0189802	0.00331848	1.50E-08	32.71304507	-0.0304	0.0372	0.4139
rs56046429	**SHBG**	C	T	-0.0151357	0.00244345	1.80E-10	38.37036421	0.0233	0.0343	0.4976
rs56196860	**SHBG**	A	C	0.0594641	0.00632483	1.00E-22	88.39135647	0.17	0.1933	0.3789
rs57310685	**SHBG**	C	T	0.0119248	0.0020563	2.30E-09	33.63001562	0.0311	0.0212	0.1412
rs57400569	**SHBG**	A	G	0.0164966	0.00243935	1.40E-10	45.73392392	0.0205	0.0162	0.2072
rs5745687	**SHBG**	T	C	0.0354454	0.00407193	3.70E-20	75.77343385	-4.00E-04	0.1156	0.9971
rs57467915	**SHBG**	A	G	-0.0567963	0.00859704	1.00E-11	43.6455536	0.5603	0.1855	0.00251698
rs5751777	**SHBG**	T	C	-0.0192258	0.00204779	7.90E-22	88.14462145	0.055	0.0494	0.2653
rs5757169	**SHBG**	A	G	-0.0124503	0.00219844	2.40E-09	32.07216003	0.02	0.1201	0.8675
rs58263961	**SHBG**	T	C	0.0146473	0.00255074	6.30E-09	32.97467929	0.0054	0.0179	0.7623
rs59774409	**SHBG**	T	C	0.0516402	0.00367013	2.30E-46	197.9751807	0.021	0.0414	0.6124
rs61154548	**SHBG**	T	C	-0.0334288	0.00437791	2.60E-14	58.30499035	-0.0171	0.0314	0.5866
rs6129800	**SHBG**	A	G	0.0256138	0.00241957	6.80E-28	112.0648614	-0.024	0.0181	0.1863
rs6142206	**SHBG**	A	G	-0.0145041	0.00204237	2.80E-14	50.43250474	0.036	0.0186	0.05347
rs61754233	**SHBG**	C	G	0.0356787	0.00726105	4.10E-09	24.14443247	-0.1132	0.1757	0.5194
rs61856594	**SHBG**	G	A	0.0110038	0.00218859	4.30E-08	25.27867858	0.0216	0.0229	0.3444
rs61935492	**SHBG**	G	T	-0.0276983	0.00300482	4.80E-23	84.97029371	-0.0201	0.0378	0.5948
rs62292896	**SHBG**	A	G	-0.0162734	0.00298508	1.40E-09	29.71956593	0.0305	0.024	0.2027
rs62433130	**SHBG**	T	C	-0.0190559	0.00307156	3.80E-12	38.48918685	0.0064	0.035	0.856
rs62576339	**SHBG**	C	T	0.0298708	0.00235372	9.00E-38	161.0578603	-0.0174	0.0186	0.3485
rs62577365	**SHBG**	T	C	-0.0273521	0.00446658	6.50E-11	37.49979811	-0.1997	0.1218	0.1011
rs62580767	**SHBG**	C	T	0.0266015	0.00261273	5.20E-30	103.6623383	-0.0062	0.0741	0.9332
rs62618693	**SHBG**	T	C	0.0414741	0.00496366	9.00E-18	69.81483277	-0.1327	0.1411	0.347
rs628751	**SHBG**	A	C	0.0129359	0.00201163	4.10E-11	41.35184622	-0.0186	0.0185	0.313
rs638971	**SHBG**	A	G	0.0763495	0.0123779	3.30E-09	38.04663635	0.028	0.0214	0.1914
rs6415788	**SHBG**	T	G	-0.0137581	0.00206684	1.90E-12	44.30992552	0.0045	0.0231	0.8469
rs6424245	**SHBG**	C	A	0.0107333	0.00200757	1.10E-08	28.58399679	-0.0198	0.0157	0.2076
rs6445064	**SHBG**	G	A	0.014524	0.00212706	5.60E-10	46.62413757	-0.0076	0.0162	0.6369
rs645040	**SHBG**	T	G	-0.0655117	0.00239357	5.60E-179	749.1060164	0.0086	0.0207	0.6761
rs6479896	**SHBG**	C	T	0.131071	0.00200139	1.00E-200	4288.91646	0.0093	0.0158	0.5555
rs6486122	**SHBG**	T	C	-0.0253949	0.00217054	5.60E-36	136.8848042	-7.00E-04	0.0171	0.9692
rs6489190	**SHBG**	T	C	0.0126315	0.00247179	1.00E-08	26.11466887	0.0471	0.0179	0.00837298
rs6557781	**SHBG**	C	T	0.0151942	0.00283458	2.30E-08	28.73267448	0.0337	0.0477	0.4804
rs66720010	**SHBG**	G	A	0.0319913	0.00208358	8.10E-57	235.7443154	-0.0022	0.0201	0.914
rs67199213	**SHBG**	G	A	-0.029583	0.00228802	1.60E-43	167.1716414	0.0038	0.0162	0.813
rs6720394	**SHBG**	G	T	0.0193464	0.003182	1.40E-11	36.96560236	0.0713	0.0754	0.3442
rs6736913	**SHBG**	G	A	-0.0804034	0.00705593	1.50E-33	129.8488517	0.0379	0.179	0.8324
rs6755571	**SHBG**	A	C	-0.0301056	0.00403164	2.70E-14	55.76078712	-0.1519	0.1306	0.2447
rs6756943	**SHBG**	A	G	-0.0440705	0.00217335	1.50E-97	411.1823128	-0.0517	0.0284	0.06871
rs67611724	**SHBG**	T	C	-0.0335632	0.00278365	3.10E-35	145.3768575	-0.1375	0.0654	0.0355402
rs6797405	**SHBG**	A	G	0.0133459	0.00208442	1.90E-11	40.9942629	-0.0015	0.0159	0.9255
rs6798189	**SHBG**	A	G	0.0145595	0.00227091	5.60E-12	41.10464373	-0.1741	0.0601	0.00374197

rs6809436	**SHBG**	G	T	-0.0202434	0.00371263	3.80E-09	29.7304434	0.0932	0.0976	0.3393
rs6812193	**SHBG**	T	C	0.0303929	0.00206649	2.70E-53	216.3094428	0.0157	0.027	0.5603
rs6831256	**SHBG**	G	A	0.0471454	0.00202852	3.20E-128	540.1543452	-0.0109	0.0163	0.5043
rs6861681	**SHBG**	A	G	-0.0170834	0.00218527	3.50E-17	61.11336884	-0.0161	0.0242	0.5059
rs6939861	**SHBG**	A	G	-0.0340709	0.00231398	2.80E-51	216.793283	-0.0116	0.0182	0.523801
rs7015812	**SHBG**	G	C	0.012039	0.00203957	9.90E-10	34.84186636	-0.0025	0.0201	0.9028
rs7026694	**SHBG**	C	T	0.0207694	0.00371505	1.70E-08	31.25477573	-0.1619	0.1107	0.1435
rs7109616	**SHBG**	T	C	-0.0386818	0.00651461	3.40E-09	35.25610407	-0.0271	0.1002	0.7866
rs7134375	**SHBG**	A	C	0.0143024	0.00201827	2.50E-12	50.21773523	-0.0257	0.0489	0.599601
rs7161750	**SHBG**	C	A	-0.0593281	0.00327022	2.20E-74	329.1279404	0.0351	0.0255	0.1694
rs7175361	**SHBG**	A	G	0.0222105	0.00275146	6.40E-17	65.16105034	-0.004	0.0173	0.818
rs7220814	**SHBG**	G	A	-0.0396407	0.00404019	1.50E-24	96.26687265	-0.0069	0.0189	0.715801
rs724577	**SHBG**	C	A	-0.0189375	0.00227752	1.10E-16	69.13830135	-0.0088	0.0159	0.5797
rs72631343	**SHBG**	G	C	-0.0219298	0.00287384	7.60E-14	58.22935555	-0.0018	0.0167	0.9126
rs72663933	**SHBG**	C	T	-0.0158543	0.00297328	9.30E-09	28.43284626	-0.0478	0.0735	0.5157
rs72670345	**SHBG**	T	C	0.0386548	0.00447298	1.10E-16	74.6811094	0.0199	0.0208	0.3386
rs72683923	**SHBG**	C	T	0.0738895	0.00744304	5.60E-27	98.5513464	0.1164	0.2056	0.5712
rs727428	**SHBG**	C	T	0.236988	0.0019417	1.00E-200	14896.56935	0.0036	0.0161	0.8245
rs72743115	**SHBG**	C	A	-0.0806144	0.00509558	5.90E-61	250.2855724	-0.1828	0.1103	0.09749
rs72751639	**SHBG**	A	G	-0.0282582	0.00395963	2.40E-11	50.93045891	-0.0223	0.1136	0.8444
rs72787511	**SHBG**	C	G	0.0336959	0.00529057	1.90E-09	40.56458187	0.2409	0.1541	0.1179
rs72790275	**SHBG**	T	C	0.0169229	0.00204269	1.20E-17	68.63449823	0.0352	0.0353	0.3183
rs72832032	**SHBG**	A	G	-0.0505715	0.00648803	4.60E-15	60.75524612	-0.062	0.1652	0.707301
rs72902436	**SHBG**	A	G	0.0160479	0.00236203	2.60E-14	46.15974625	-0.0141	0.0215	0.5108
rs72906888	**SHBG**	T	G	0.0295018	0.00346752	5.00E-17	72.3863856	-0.0088	0.0291	0.7614
rs72948115	**SHBG**	T	C	-0.024587	0.00340333	2.80E-12	52.19161921	-0.0063	0.0937	0.9466
rs7310409	**SHBG**	G	A	0.040455	0.00206445	2.60E-97	384.0019993	-0.0094	0.0158	0.5513
rs7314285	**SHBG**	G	T	0.0868421	0.00396533	2.30E-115	479.6227499	-0.0254	0.0244	0.297
rs731839	**SHBG**	A	G	0.0213152	0.0021299	3.20E-28	100.1516732	-0.0242	0.0158	0.1268
rs73201506	**SHBG**	G	A	-0.0295749	0.00488458	9.70E-12	36.65978623	-0.046	0.0902	0.609799
rs738408	**SHBG**	T	C	0.05444	0.00253073	3.90E-105	462.7457286	-0.0044	0.0161	0.785199
rs7412	**SHBG**	T	C	0.026561	0.00370536	1.10E-12	51.38375499	-4.00E-04	0.0392	0.9915
rs7429135	**SHBG**	G	T	0.0218192	0.00273761	2.50E-17	63.52319031	-0.0234	0.0183	0.2009
rs74406200	**SHBG**	T	C	0.0145327	0.00257769	2.20E-09	31.78550061	-0.0431	0.0748	0.5644
rs7445090	**SHBG**	T	C	0.0135097	0.00216658	2.00E-12	38.88120919	-0.0072	0.0162	0.6562
rs7451021	**SHBG**	C	T	-0.0255358	0.00218684	2.40E-32	136.3523824	-0.0205	0.0192	0.2873
rs7515341	**SHBG**	C	G	-0.0244627	0.00421438	1.50E-08	33.69296311	0.1484	0.1039	0.1533
rs7520897	**SHBG**	A	G	-0.0130375	0.00203145	2.10E-10	41.18832967	-0.0144	0.02	0.473301
rs756637	**SHBG**	G	A	0.0208923	0.0022596	3.60E-20	85.48839963	0.3204	0.2382	0.1786
rs76074266	**SHBG**	G	C	0.0265438	0.00490921	2.60E-08	29.23484427	-0.2041	0.1202	0.0896293
rs7644763	**SHBG**	T	C	-0.026145	0.00361544	6.50E-14	52.29411137	0.0896	0.0573	0.1183
rs76610881	**SHBG**	G	A	0.0423756	0.00322	1.00E-42	173.1879972	0.0419	0.0737	0.5692
rs7694379	**SHBG**	A	G	-0.0438188	0.00202181	2.80E-114	469.7189613	-0.0159	0.0165	0.3327
rs7697204	**SHBG**	T	C	-0.0314324	0.00229025	2.10E-45	188.35932	3.00E-04	0.021	0.9887

rs7726159	**SHBG**	A	C	-0.0116575	0.00214004	1.00E-09	29.67323382	-0.0094	0.0173	0.587899
rs7756992	**SHBG**	G	A	-0.0183914	0.00227663	3.70E-16	65.25931092	0.0082	0.0159	0.6036
rs77738818	**SHBG**	A	G	-0.0417608	0.00654888	2.80E-09	40.66318259	-0.0301	0.1272	0.8131
rs7786120	**SHBG**	C	T	0.0175343	0.00209564	9.10E-18	70.00697613	-4.00E-04	0.0159	0.9806
rs7789908	**SHBG**	G	T	-0.0190334	0.00247279	9.50E-16	59.24559838	0.0509	0.0381	0.1816
rs78081080	**SHBG**	T	C	-0.102974	0.00365807	1.50E-178	792.408189	0.0611	0.0814	0.453
rs7850488	**SHBG**	C	G	-0.0134535	0.00216438	1.30E-08	38.63681818	-0.0103	0.0161	0.5219
rs7860634	**SHBG**	A	G	0.0225208	0.0020954	1.30E-29	115.5131856	-0.0314	0.0174	0.0714908
rs7903146	**SHBG**	T	C	-0.0228374	0.00219782	3.20E-29	107.9709413	-0.0051	0.0333	0.8782
rs79784917	**SHBG**	T	G	0.0218681	0.00308804	5.50E-15	50.14805348	0.0332	0.0436	0.4464
rs8017377	**SHBG**	A	G	-0.036066	0.00206993	6.70E-69	303.5864972	-0.0601	0.0306	0.0494698
rs8023580	**SHBG**	C	T	0.0825808	0.00224914	1.00E-200	1348.102783	-0.009	0.0166	0.5886
rs802658	**SHBG**	C	T	0.0141566	0.00233145	2.80E-10	36.86918371	-0.007	0.0178	0.6957
rs803736	**SHBG**	A	G	-0.0175202	0.00287972	4.60E-11	37.01479991	-0.0199	0.0218	0.3621
rs8066822	**SHBG**	C	T	0.044438	0.00721406	9.00E-10	37.94436781	-0.384	0.2508	0.1257
rs8095050	**SHBG**	A	G	0.0170916	0.00204839	3.20E-17	69.62063162	0.0196	0.0241	0.4163
rs811970	**SHBG**	G	A	0.0138261	0.00236004	7.40E-10	34.32088452	0.0066	0.034	0.8469
rs849134	**SHBG**	G	A	0.0251594	0.00200381	1.60E-37	157.6468484	-0.0369	0.0189	0.0503802
rs870526	**SHBG**	T	C	-0.013369	0.00199675	7.70E-13	44.82788743	0.0178	0.0159	0.2637
rs892161	**SHBG**	G	A	-0.0123828	0.00209702	1.30E-11	34.86826872	0.0038	0.0161	0.8149
rs9379084	**SHBG**	A	G	-0.0361618	0.00320919	4.40E-33	126.9717285	0.0184	0.0192	0.3382
rs9427104	**SHBG**	T	C	0.0293486	0.00199433	4.00E-56	216.5601513	-1.00E-04	0.0159	0.9946
rs9491675	**SHBG**	A	G	-0.0159832	0.00286398	4.50E-09	31.14478365	0.0182	0.018	0.3121
rs9556403	**SHBG**	G	A	0.0226244	0.00211001	9.40E-31	114.9695751	0.0095	0.0159	0.5501
rs9617090	**SHBG**	T	C	0.0136095	0.00205458	2.40E-12	43.87691047	0.0325	0.0191	0.0885503
rs9634098	**SHBG**	C	T	0.059323	0.00533505	5.20E-31	123.6422886	0.2995	0.1759	0.0885992
rs9644032	**SHBG**	G	T	-0.0181446	0.00208483	3.70E-20	75.74453677	0.0186	0.0183	0.3103
rs9757252	**SHBG**	C	T	-0.0123564	0.0020627	1.70E-08	35.88472255	-0.0105	0.0159	0.5086
rs976002	**SHBG**	G	A	0.0385776	0.00235537	2.60E-65	268.2563094	-0.0191	0.0262	0.4663
rs9847154	**SHBG**	C	T	0.0110714	0.00202431	1.10E-08	29.91223436	-0.0036	0.0477	0.9399
rs988911	**SHBG**	A	G	0.0222509	0.00293187	3.80E-16	57.59748985	0.0201	0.0175	0.2516
rs9894648	**SHBG**	C	T	0.0247604	0.00199432	1.20E-35	154.1428682	0.022	0.0158	0.1653
rs9944207	**SHBG**	T	A	0.0128803	0.00231854	2.30E-08	30.86175247	0.0045	0.0161	0.7796
rs997633	**SHBG**	T	A	-0.0133894	0.00274879	4.00E-08	23.7266771	-0.0362	0.0237	0.1262

SHBG: Sex hormone-binding globulin levels. EA: effect allele. OA: other allele. GX: beta-exposure. GX(SE): standard error of GX. GY: beta-outcome. GY(SE): standard error of GY.

Table S18. Published associations of CRP on PTB

SNP	Exposure	EA	EA	GX	GX(SE)	Pval-exp	F	GY	GY(SE)	Pval-outcome
rs10151525	**CRP**	G	A	0.0227179	0.00433548	2.90E-08	27.45743758	0.2532	0.1222	0.0383098
rs10153578	**CRP**	G	A	-0.0231334	0.00239789	4.60E-24	93.07186867	-0.053	0.0256	0.0381496
rs1047891	**CRP**	A	C	-0.0128442	0.00219165	3.30E-09	34.34549116	-0.0375	0.0219	0.0874299
rs10504360	**CRP**	G	A	0.0176739	0.00343036	1.40E-08	26.54504096	-0.0284	0.0316	0.3691
rs10513689	**CRP**	A	G	0.0181696	0.00303612	3.10E-09	35.81383923	-0.007	0.0445	0.8758
rs1064725	**CRP**	G	T	0.0571412	0.00548574	4.10E-27	108.4991373	-0.1134	0.1237	0.359
rs10745954	**CRP**	G	A	-0.0240121	0.00198309	1.70E-37	146.6133272	-0.0293	0.0171	0.0871104
rs1076540	**CRP**	T	C	-0.0140993	0.00235139	4.10E-11	35.95371579	-0.0053	0.0338	0.8748
rs10899123	**CRP**	G	C	-0.0261841	0.00360963	1.60E-12	52.61964806	0.0215	0.0182	0.2385
rs10936873	**CRP**	G	A	0.0243546	0.00334431	6.70E-13	53.03309332	-0.0026	0.0687	0.9701
rs11054397	**CRP**	A	G	-0.0141846	0.00202097	8.00E-14	49.26204826	0.0232	0.0253	0.358
rs11075253	**CRP**	A	C	0.0141943	0.00218798	4.30E-10	42.08615751	0.037	0.053	0.485801
rs111300036	**CRP**	T	C	-0.020216	0.00363166	1.20E-08	30.98689815	0.0817	0.1139	0.473
rs11177742	**CRP**	A	C	-0.0157224	0.00249761	1.80E-10	39.62656662	-0.0229	0.0205	0.2642
rs114165349	**CRP**	C	G	-0.0954527	0.00642636	1.10E-52	220.6197284	0.0272	0.1301	0.8345
rs11465932	**CRP**	A	G	-0.0108781	0.00201001	2.50E-08	29.2892108	-0.0244	0.0175	0.1633
rs115125308	**CRP**	A	G	-0.0302178	0.00472192	6.70E-11	40.95306845	-0.0178	0.0286	0.5321
rs11568544	**CRP**	G	A	0.0139816	0.00207778	1.80E-13	45.28064822	0.024	0.0288	0.4036
rs11635067	**CRP**	C	G	0.0118013	0.00211645	6.80E-09	31.0915009	-0.0199	0.0207	0.3361
rs11640223	**CRP**	T	G	0.0232677	0.00244329	1.50E-22	90.68905748	-0.0281	0.0588	0.6326
rs11665829	**CRP**	A	G	-0.038733	0.00204706	5.00E-82	358.0132825	0.0513	0.0275	0.0621799
rs11670056	**CRP**	T	C	-0.0209533	0.00374742	1.90E-08	31.26353868	-0.1688	0.1247	0.1758
rs11681133	**CRP**	T	C	-0.0152505	0.0024092	1.00E-10	40.07010388	0.0065	0.0429	0.8805
rs116961756	**CRP**	A	T	-0.0553142	0.00930616	1.10E-09	35.32891121	-0.005	0.3279	0.9878
rs11727676	**CRP**	C	T	0.022814	0.00340455	8.10E-12	44.90363025	-0.0298	0.0851	0.7262
rs11755724	**CRP**	G	A	0.0155458	0.00207531	2.30E-15	56.11231762	-0.0221	0.049	0.652299
rs117973083	**CRP**	T	C	-0.0373686	0.00641552	8.80E-12	33.92719388	0.1904	0.1644	0.2466
rs11806	**CRP**	G	A	-0.0201718	0.00211806	7.30E-23	90.7007341	-0.0092	0.0167	0.5833
rs11812460	**CRP**	A	G	-0.0167565	0.00229297	3.40E-15	53.40327465	-0.0279	0.0168	0.0971091
rs11850396	**CRP**	A	G	-0.0198384	0.00208338	3.00E-23	90.67223398	0.0164	0.0225	0.4659
rs11928797	**CRP**	A	C	-0.0203794	0.00311985	1.90E-11	42.66908907	0.0798	0.0681	0.2413
rs12030843	**CRP**	G	C	-0.0194567	0.00266474	1.30E-13	53.31221111	-0.0194	0.0172	0.2588
rs1205	**CRP**	T	C	-0.191535	0.00206171	1.00E-200	8630.563442	-0.0111	0.0168	0.5093
rs12141189	**CRP**	C	T	-0.0262725	0.00226879	1.40E-32	134.0948859	0.0178	0.0212	0.4009
rs12162782	**CRP**	G	T	-0.0110367	0.00210635	4.60E-08	27.45461851	-0.0054	0.0169	0.751299
rs12239046	**CRP**	C	T	0.0409496	0.00201073	1.10E-93	414.7532756	-0.002	0.0161	0.9007
rs12304084	**CRP**	C	T	-0.0547532	0.00526747	4.20E-26	108.0470423	-0.1316	0.164	0.4222
rs123698	**CRP**	C	G	-0.0159651	0.00202721	8.80E-15	62.02172181	-0.0066	0.0516	0.8989
rs12464660	**CRP**	T	C	0.0123776	0.00209507	2.40E-10	34.90388936	-5.00E-04	0.0252	0.9842
rs12472324	**CRP**	T	C	0.0242633	0.00328288	3.60E-15	54.62453234	-0.0902	0.0794	0.256
rs1248860	**CRP**	A	G	-0.0106363	0.00199899	4.30E-08	28.31117703	0.0189	0.0164	0.2498
rs12595858	**CRP**	C	T	0.0205412	0.00213273	1.40E-24	92.76366063	0.0077	0.0168	0.647099

rs1260326	**CRP**	C	T	-0.0892001	0.00205587	1.00E-200	1882.510452	0.0031	0.0159	0.8472
rs12619647	**CRP**	G	T	0.0222982	0.00216119	1.30E-25	106.4514855	0.0054	0.0165	0.741401
rs12654264	**CRP**	T	A	0.0158099	0.00206368	1.60E-14	58.69100517	2.00E-04	0.0158	0.9909
rs12740374	**CRP**	T	G	0.0174772	0.00233778	3.70E-14	55.89004336	0.0388	0.028	0.1661
rs13009757	**CRP**	T	C	0.029379	0.00459216	9.10E-10	40.92964783	-0.1648	0.1594	0.3012
rs13107325	**CRP**	T	C	-0.0262409	0.00385493	6.20E-12	46.33641949	0.0932	0.1558	0.5499
rs13223343	**CRP**	C	A	-0.0180408	0.00237453	1.20E-14	57.72371507	0.0144	0.0193	0.4548
rs13596	**CRP**	C	T	-0.0109749	0.00203506	3.90E-09	29.08336939	0.0134	0.0225	0.5505
rs1364344	**CRP**	C	T	0.0144396	0.00249629	1.60E-08	33.45940884	0.0137	0.0197	0.4872
rs1408272	**CRP**	G	T	0.0433011	0.00400361	5.50E-28	116.9748076	0.1055	0.1141	0.3549
rs1412444	**CRP**	T	C	0.0286123	0.00211067	1.50E-44	183.7650332	0.0228	0.0185	0.2179
rs142296998	**CRP**	A	G	0.12499	0.0130424	2.20E-23	91.84034725	-0.028	0.1762	0.8738
rs143674704	**CRP**	G	A	-0.0706291	0.00760496	4.70E-21	86.25245959	0.0613	0.1242	0.6214
rs1441170	**CRP**	A	G	-0.0223416	0.00199297	9.50E-31	125.6680955	0.0244	0.0291	0.4005
rs147211387	**CRP**	A	G	-0.0760658	0.00831838	6.30E-22	83.61792278	0.6411	0.3179	0.0437301
rs1483840	**CRP**	G	A	-0.0117687	0.00200924	1.60E-09	34.30768118	0.0382	0.0227	0.09192
rs1545536	**CRP**	T	C	-0.0240438	0.00242618	3.20E-27	98.21056036	-0.0029	0.0166	0.8633
rs165316	**CRP**	G	A	-0.0408439	0.00244217	1.10E-63	279.7052619	0.0173	0.0193	0.3713
rs1688601	**CRP**	A	C	0.0182024	0.00349965	4.30E-08	27.05241831	-0.0094	0.0351	0.788099
rs17050272	**CRP**	A	G	0.0112196	0.00202451	3.00E-09	30.71234001	-0.023	0.0158	0.1454
rs17138478	**CRP**	A	C	0.0356665	0.00298968	5.00E-35	142.3211967	0.0112	0.0195	0.5673
rs17283010	**CRP**	A	G	0.0120485	0.00206969	1.40E-10	33.88858016	0.0273	0.0213	0.1993
rs17369400	**CRP**	G	A	-0.0201688	0.00327306	1.80E-09	37.97083937	0.0117	0.0717	0.8709
rs174550	**CRP**	C	T	-0.015091	0.00210393	4.50E-13	51.44835316	0.0466	0.0162	0.00398602
rs17616063	**CRP**	G	A	-0.143806	0.00381409	1.00E-200	1421.576225	0.1465	0.0724	0.0429903
rs17652767	**CRP**	A	G	-0.030889	0.00325867	7.90E-23	89.85141992	0.012	0.0222	0.589599
rs17737062	**CRP**	G	A	-0.0117196	0.00225982	3.60E-08	26.89526913	-0.001	0.0198	0.9579
rs17790938	**CRP**	A	G	-0.022208	0.00388601	7.70E-09	32.65946618	-0.009	0.0312	0.7728
rs1795064	**CRP**	T	C	-0.0151454	0.00209845	8.50E-13	52.09094742	0.0114	0.0165	0.4911
rs1800437	**CRP**	C	G	0.0208395	0.00250974	1.10E-16	68.94696232	-0.0402	0.0188	0.0321203
rs1800693	**CRP**	C	T	-0.0221841	0.00207884	1.00E-27	113.8779161	-0.0444	0.0199	0.0253198
rs1800961	**CRP**	T	C	-0.118508	0.00589837	1.70E-92	403.67265	-0.0871	0.0685	0.204
rs1800973	**CRP**	A	C	0.0294635	0.0041656	9.80E-13	50.02781723	0.0968	0.111	0.3828
rs1801689	**CRP**	C	A	0.0689104	0.00584723	2.40E-33	138.8887917	0.0477	0.2132	0.8228
rs1805096	**CRP**	A	G	-0.138652	0.0020134	1.00E-200	4742.312408	0.0085	0.0216	0.6946
rs1862069	**CRP**	A	G	0.0213149	0.00199847	1.40E-29	113.7546987	-0.0014	0.0168	0.933
rs1871137	**CRP**	T	C	-0.0202659	0.00248725	8.90E-18	66.38820459	-0.0112	0.0203	0.581599
rs187429064	**CRP**	G	A	0.0534695	0.00910805	9.70E-10	34.46350516	0.1367	0.1169	0.2422
rs1888162	**CRP**	T	C	0.0171398	0.00207332	6.50E-16	68.34029322	-0.0096	0.0183	0.601999
rs1990279	**CRP**	G	A	-0.0169733	0.00217864	1.60E-17	60.69593986	-0.0257	0.0186	0.1675
rs2068397	**CRP**	A	C	0.0153125	0.00210498	1.00E-12	52.9168846	-0.0349	0.016	0.0294802
rs2070971	**CRP**	T	G	0.01606	0.00292674	1.40E-09	30.11069693	0.0275	0.0177	0.1203
rs2074572	**CRP**	T	C	0.0130771	0.00208925	1.80E-10	39.17780317	0.0026	0.0165	0.8746
rs2112161	**CRP**	A	G	0.0204571	0.00222521	2.80E-21	84.5170135	0.0159	0.0199	0.4234

rs2161375	**CRP**	T	C	-0.0193371	0.00200116	5.70E-24	93.37208818	0.019	0.016	0.2345
rs2189965	**CRP**	T	C	0.0137384	0.00248355	9.10E-09	30.60021728	0.03	0.0471	0.525
rs2205262	**CRP**	A	C	0.0238549	0.00202049	2.80E-35	139.3926267	-0.0081	0.0157	0.6059
rs2207132	**CRP**	A	G	0.0652441	0.00557498	6.30E-34	136.96004	-0.0196	0.1036	0.8502
rs2214244	**CRP**	C	T	-0.0124785	0.0020213	6.70E-10	38.11195498	0.0216	0.0174	0.2148
rs2231156	**CRP**	A	C	-0.0179082	0.00337686	6.30E-09	28.12391473	0.0076	0.0186	0.6821
rs2242262	**CRP**	T	G	-0.030457	0.00278435	3.40E-29	119.6532794	0.011	0.0168	0.5132
rs2247935	**CRP**	G	A	-0.0128912	0.0020005	2.20E-10	41.5248042	-0.0106	0.0166	0.5208
rs2250010	**CRP**	T	C	0.0205034	0.00252619	2.20E-18	65.87456458	0.007	0.0164	0.667899
rs2272157	**CRP**	T	C	-0.0147653	0.00234424	7.30E-11	39.67148145	0.0129	0.0197	0.511
rs2276824	**CRP**	G	C	-0.0101489	0.00201651	1.10E-08	25.33000051	-0.0105	0.0159	0.512
rs2283371	**CRP**	G	A	0.0306191	0.00212281	1.20E-52	208.0465914	0.0014	0.016	0.9326
rs2286028	**CRP**	C	G	-0.0171733	0.00253035	3.70E-12	46.06216332	3.00E-04	0.0279	0.9909
rs2289850	**CRP**	C	T	-0.0316695	0.00446377	9.60E-14	50.33578054	0.0114	0.02	0.5665
rs2290911	**CRP**	G	A	0.0141	0.00211823	8.20E-12	44.30880204	0.0118	0.0188	0.531401
rs2293476	**CRP**	C	G	0.0269932	0.00232068	3.00E-31	135.2932839	-0.0272	0.0218	0.2136
rs2302503	**CRP**	A	G	-0.0127798	0.00200701	8.10E-11	40.54591018	0.0024	0.0209	0.9101
rs2303998	**CRP**	A	G	-0.0573169	0.00739349	5.40E-16	60.09860887	0.0177	0.1317	0.8932
rs2333138	**CRP**	A	G	0.0120427	0.00222551	4.30E-09	29.28105007	-0.0061	0.0255	0.8121
rs2393791	**CRP**	T	C	0.164308	0.00204574	1.00E-200	6450.814551	-0.009	0.0158	0.569101
rs2426776	**CRP**	A	G	0.0136001	0.00199736	2.40E-12	46.36278532	-0.0163	0.0158	0.3002
rs2472297	**CRP**	T	C	-0.015013	0.00227679	4.60E-13	43.47974925	0.0562	0.0553	0.3092
rs2522056	**CRP**	A	G	-0.0164047	0.00256136	7.90E-11	41.01978233	-0.0136	0.0164	0.4086
rs2665404	**CRP**	T	C	0.0252982	0.0020047	3.30E-39	159.2496457	-0.0205	0.0164	0.2102
rs2710804	**CRP**	C	T	0.0272609	0.00206143	3.70E-41	174.8804279	-0.0085	0.0199	0.6693
rs2790117	**CRP**	T	C	0.0151284	0.00218928	2.20E-12	47.75088121	0.0228	0.0171	0.1825
rs2823662	**CRP**	G	A	-0.0116861	0.00202666	1.70E-09	33.24875723	0.0273	0.0406	0.5008
rs28361325	**CRP**	T	C	-0.0122972	0.002061	1.00E-09	35.60036915	0.0127	0.0167	0.4447
rs2836878	**CRP**	A	G	-0.036318	0.0022682	4.50E-63	256.3770633	0.0467	0.021	0.02646
rs28404841	**CRP**	T	C	0.0208446	0.00283724	2.00E-14	53.97504279	0.0249	0.0207	0.2293
rs2857211	**CRP**	G	A	-0.0187599	0.00319517	2.80E-09	34.47236745	0.0068	0.0181	0.7072
rs28638160	**CRP**	A	C	0.0190939	0.00233669	3.50E-17	66.77057244	0.0045	0.029	0.8758
rs28710284	**CRP**	C	G	0.0211006	0.00264245	6.30E-17	63.76378147	-0.0182	0.0167	0.276
rs28929474	**CRP**	T	C	-0.0991607	0.00722408	9.10E-49	188.41353	0.2462	0.1735	0.156
rs2965206	**CRP**	C	T	0.0117087	0.00221445	1.10E-09	27.95655231	0.0131	0.0177	0.4578
rs3024544	**CRP**	T	C	-0.0166708	0.00286526	2.80E-09	33.85188219	5.00E-04	0.0781	0.9949
rs303429	**CRP**	T	C	-0.0167189	0.00205319	6.30E-17	66.30635152	-0.0252	0.0159	0.1131
rs3064	**CRP**	C	G	0.0147736	0.00213291	1.50E-12	47.97617541	0.0223	0.0172	0.1933
rs3094519	**CRP**	A	G	0.0136938	0.00231144	8.00E-09	35.09786703	-0.0501	0.0591	0.3968
rs324137	**CRP**	C	A	-0.0101079	0.00204282	1.90E-08	24.48272099	0.0174	0.0158	0.271
rs339969	**CRP**	A	C	0.0313271	0.00205322	4.20E-56	232.7916652	-0.0184	0.0252	0.4667
rs34048412	**CRP**	A	G	-0.0314762	0.00400263	1.60E-15	61.84031772	0.059	0.0791	0.4558
rs34162105	**CRP**	C	T	-0.0156412	0.00252193	7.40E-09	38.46556313	-0.0243	0.0258	0.3465
rs34298354	**CRP**	T	C	-0.0271167	0.00297562	3.90E-20	83.04562287	0.0172	0.0244	0.4814

rs34575488	**CRP**	G	A	0.0145787	0.00220864	4.70E-10	43.5698178	-0.0137	0.0374	0.7154
rs34982954	**CRP**	C	T	-0.0106498	0.0020011	3.00E-08	28.32326606	-0.0203	0.0196	0.2991
rs35256099	**CRP**	A	G	-0.0382519	0.00556902	5.80E-11	47.17875547	0.0508	0.2396	0.8321
rs35489971	**CRP**	G	A	0.0354693	0.00257525	1.70E-44	189.6987441	0.0268	0.0176	0.1277
rs35507645	**CRP**	G	A	0.011756	0.00200183	3.30E-10	34.4875847	-0.0153	0.0163	0.3472
rs35551980	**CRP**	C	T	-0.0110421	0.00211859	4.20E-08	27.16486355	-0.0261	0.0184	0.1552
rs35629860	**CRP**	A	G	-0.0152875	0.00233095	2.70E-11	43.01352517	-0.0217	0.0264	0.4106
rs35816944	**CRP**	A	G	0.0833559	0.0126756	1.90E-10	43.24477585	0.3144	0.2962	0.2884
rs372883	**CRP**	C	T	-0.010583	0.00200848	3.00E-08	27.76390703	-0.0156	0.0159	0.3266
rs3738725	**CRP**	C	T	0.0125982	0.00194711	1.70E-10	41.86335542	-0.0317	0.0209	0.1293
rs3741380	**CRP**	A	G	0.0139659	0.00203546	3.30E-11	47.07721076	-0.0131	0.016	0.4134
rs3756772	**CRP**	T	C	0.0205953	0.00204487	1.50E-27	101.4385073	-0.0011	0.0171	0.9489
rs3805433	**CRP**	G	C	0.014529	0.00224417	2.30E-11	41.9138886	0.0021	0.0166	0.9007
rs3808348	**CRP**	T	C	-0.0243231	0.00248658	1.30E-22	95.68216429	0.0126	0.0167	0.4495
rs3812316	**CRP**	G	C	-0.0433117	0.0029967	6.30E-51	208.892062	0.0027	0.0262	0.9189
rs3818717	**CRP**	C	T	-0.0126196	0.00207794	8.20E-11	36.88274062	1.00E-04	0.026	0.9977
rs3849967	**CRP**	T	C	0.010799	0.00202855	1.80E-08	28.3395964	-0.0172	0.0228	0.450199
rs3924871	**CRP**	A	G	-0.0117617	0.0021084	3.30E-08	31.11947006	0.0031	0.0174	0.859
rs41275126	**CRP**	C	T	-0.0224874	0.00301949	3.00E-14	55.46376039	0.0101	0.0287	0.723901
rs4245184	**CRP**	A	G	0.0131571	0.00203482	1.20E-10	41.80867152	-0.0175	0.0189	0.3555
rs4266763	**CRP**	G	A	0.0235328	0.00204861	1.70E-31	131.9552407	-0.0351	0.0166	0.0346003
rs429358	**CRP**	C	T	-0.29028	0.00276149	1.00E-200	11049.5678	-0.0081	0.0259	0.7548
rs4406875	**CRP**	G	A	-0.0128081	0.00198306	2.10E-10	41.71533374	-0.0107	0.0196	0.5857
rs4410790	**CRP**	C	T	-0.0133521	0.00207264	3.00E-11	41.50012587	-8.00E-04	0.0163	0.9626
rs4559074	**CRP**	A	G	-0.0267811	0.00470726	4.80E-08	32.36819345	0.029	0.0647	0.654
rs45604939	**CRP**	G	A	0.0208449	0.00398731	1.60E-08	27.32987468	0.0363	0.0947	0.701699
rs4622954	**CRP**	G	T	0.0136188	0.00201796	1.10E-10	45.54603645	-0.017	0.0214	0.4273
rs4635400	**CRP**	A	G	0.0159698	0.00208609	4.30E-16	58.60448908	0.0132	0.0194	0.498
rs4654887	**CRP**	T	C	-0.0111999	0.00198414	3.50E-08	31.86263416	0.0027	0.018	0.8796
rs4717903	**CRP**	C	T	-0.0233997	0.00228252	5.20E-27	105.0967794	-8.00E-04	0.032	0.9799
rs4790814	**CRP**	A	G	-0.0174211	0.00200147	1.50E-18	75.76192305	0.0146	0.0177	0.4087
rs4809221	**CRP**	A	G	0.0183209	0.00215307	5.20E-17	72.40612757	-0.0034	0.0164	0.8367
rs481206	**CRP**	T	C	0.0225275	0.00210488	2.60E-27	114.5432036	0.0175	0.0175	0.3152
rs4938933	**CRP**	T	C	0.0196003	0.00203604	1.40E-23	92.67249117	-0.0098	0.0176	0.578
rs4988483	**CRP**	A	C	0.0297553	0.0044046	3.90E-12	45.63664712	0.1355	0.1536	0.3775
rs505922	**CRP**	C	T	0.0250649	0.00215207	4.30E-32	135.6491816	0.0125	0.0159	0.4297
rs55707100	**CRP**	T	C	0.0956034	0.00636482	2.90E-53	225.6173195	0.3604	0.257	0.1608
rs557302	**CRP**	G	A	-0.0150617	0.0019987	1.10E-15	56.78724155	-0.0018	0.0158	0.9072
rs55855238	**CRP**	C	T	0.0281928	0.0021022	8.60E-46	179.856596	0.024	0.0201	0.2319
rs56047269	**CRP**	T	C	0.0169782	0.00222848	4.10E-15	58.04486707	0.0104	0.0223	0.639501
rs56077916	**CRP**	T	C	0.0151981	0.00205074	1.10E-13	54.92314561	0.0026	0.0267	0.9237
rs572144	**CRP**	C	T	-0.0173964	0.00194572	2.30E-21	79.93851864	-0.0139	0.0166	0.4012
rs57405871	**CRP**	T	C	-0.0195274	0.00338531	2.00E-09	33.27284745	0.0485	0.089	0.5857
rs5751777	**CRP**	T	C	0.0102887	0.00203375	2.20E-08	25.59315863	0.055	0.0494	0.2653

rs57561814	**CRP**	C	G	-0.051114	0.00416129	3.50E-37	150.8765635	0.0359	0.017	0.0351301
rs57634646	**CRP**	A	G	-0.0140445	0.0024386	3.00E-10	33.16877544	0.0013	0.0202	0.9467
rs58542926	**CRP**	T	C	0.0451298	0.00384964	3.60E-34	137.4309642	0.0338	0.0298	0.2561
rs58759531	**CRP**	T	C	0.035021	0.00618534	2.20E-09	32.05737396	0.0483	0.1685	0.774499
rs6038557	**CRP**	A	G	-0.0117239	0.00207399	9.00E-09	31.95427047	-0.0179	0.0224	0.4244
rs6059969	**CRP**	C	T	-0.0160515	0.00262049	3.10E-11	37.52012226	-0.0385	0.0214	0.0715995
rs6082354	**CRP**	C	A	0.0119407	0.00212466	3.60E-08	31.58484159	0.0343	0.0159	0.0311501
rs61587156	**CRP**	G	T	0.0157887	0.00257001	9.30E-11	37.7416716	0.0126	0.0465	0.786001
rs61812598	**CRP**	A	G	-0.102589	0.00197174	1.00E-200	2707.075144	-0.0152	0.0162	0.3488
rs62079155	**CRP**	G	C	0.0441351	0.00323591	1.50E-45	186.0258826	0.0105	0.0271	0.6995
rs62513181	**CRP**	G	A	0.022699	0.00407108	3.30E-08	31.08795742	0.0131	0.0322	0.6851
rs62618693	**CRP**	T	C	-0.0284966	0.00493614	2.90E-09	33.32799345	-0.1327	0.1411	0.347
rs62621197	**CRP**	T	C	-0.034886	0.00539597	3.00E-10	41.79855922	0.0874	0.1643	0.5948
rs6426749	**CRP**	C	G	-0.0151812	0.0025405	2.60E-09	35.70851726	-0.0162	0.0196	0.4085
rs645040	**CRP**	T	G	-0.0373037	0.00238376	5.10E-57	244.893228	0.0086	0.0207	0.6761
rs6486122	**CRP**	T	C	0.026533	0.00215829	2.30E-36	151.1301555	-7.00E-04	0.0171	0.9692
rs6519133	**CRP**	C	T	-0.0384938	0.00205784	5.30E-84	349.9100157	0.0127	0.016	0.4269
rs6588142	**CRP**	T	C	0.017251	0.00195701	2.50E-20	77.7034806	-0.0278	0.0207	0.178
rs661561	**CRP**	C	A	-0.0153027	0.00209461	3.90E-14	53.37375963	0.0272	0.0219	0.2134
rs66550728	**CRP**	G	A	0.0224656	0.0028011	6.30E-17	64.32456072	0.0164	0.0241	0.4956
rs6661360	**CRP**	G	A	0.0228562	0.00320354	4.50E-12	50.90327975	-0.0094	0.0163	0.5653
rs6672627	**CRP**	A	C	-0.0569087	0.00268997	1.90E-103	447.5694848	-0.0088	0.0234	0.706999
rs673254	**CRP**	T	C	0.0124618	0.00194173	4.20E-11	41.18906039	0.0037	0.048	0.9383
rs6734238	**CRP**	G	A	0.0484067	0.00202696	4.50E-137	570.3200093	0.0303	0.0302	0.3162
rs6736913	**CRP**	G	A	-0.0413678	0.00703747	5.70E-10	34.55331738	0.0379	0.179	0.8324
rs6756943	**CRP**	A	G	-0.0114193	0.00216809	9.40E-09	27.74101612	-0.0517	0.0284	0.06871
rs6788650	**CRP**	C	T	0.0190512	0.00209529	3.70E-20	82.67123093	0.0021	0.0322	0.9477
rs6792995	**CRP**	T	C	0.0189937	0.0026966	8.40E-13	49.61169899	0.005	0.0567	0.9295
rs6831256	**CRP**	G	A	-0.0165651	0.00202474	3.20E-18	66.93412811	-0.0109	0.0163	0.5043
rs6841889	**CRP**	T	C	0.0131239	0.00218088	6.00E-09	36.21264921	0.004	0.0216	0.8515
rs6884621	**CRP**	A	G	-0.0138017	0.00213732	6.40E-10	41.69885011	-0.0038	0.0164	0.8166
rs6952262	**CRP**	G	C	-0.0137038	0.00249384	4.70E-08	30.19554429	0.009	0.0162	0.579399
rs6988835	**CRP**	G	A	-0.0193797	0.00332083	1.80E-10	34.05640676	0.0044	0.0206	0.832
rs6989383	**CRP**	T	C	0.0191104	0.00200663	2.20E-23	90.6990974	-0.0388	0.0159	0.01467
rs7002992	**CRP**	C	T	0.0144083	0.00207297	8.10E-12	48.31004734	0.0227	0.0182	0.2128
rs702225	**CRP**	C	T	0.0145527	0.00221619	6.90E-12	43.11924853	0.0182	0.0333	0.584501
rs704017	**CRP**	G	A	-0.0198134	0.00202144	5.20E-24	96.07144354	0.0032	0.0166	0.8461
rs7102941	**CRP**	C	T	-0.0217298	0.00307524	9.40E-14	49.92883589	-0.0276	0.0502	0.5824
rs7237147	**CRP**	T	C	-0.0190512	0.00204465	3.10E-20	86.81699129	-0.0015	0.016	0.9251
rs7241918	**CRP**	T	G	0.0147571	0.00263282	9.30E-09	31.4164939	0.0247	0.0255	0.3334
rs7261425	**CRP**	G	C	0.0117857	0.00223646	9.10E-09	27.7706795	-0.003	0.0268	0.9123
rs7451021	**CRP**	C	T	0.0307309	0.00217408	3.00E-49	199.800941	-0.0205	0.0192	0.2873
rs7479832	**CRP**	G	A	-0.0129716	0.00232771	1.60E-09	31.05470181	0.0094	0.054	0.8623
rs7486351	**CRP**	A	T	0.043084	0.00574851	6.70E-16	56.17194529	0.0764	0.1305	0.5582

rs74900413	**CRP**	A	G	0.040009	0.00807429	3.10E-08	24.55300871	0.3731	0.2223	0.0933706
rs7543562	**CRP**	G	A	-0.0152234	0.00200287	8.90E-16	57.77178769	-0.0088	0.0159	0.5802
rs7546398	**CRP**	C	T	-0.0115698	0.00196032	3.70E-09	34.83339253	-0.0135	0.0185	0.4655
rs76075198	**CRP**	T	C	0.0491246	0.00642555	3.10E-16	58.44878014	-0.0803	0.2192	0.7141
rs762360	**CRP**	T	C	-0.0179388	0.00219189	1.70E-16	66.98032915	9.00E-04	0.0161	0.9557
rs7625680	**CRP**	A	G	0.0134758	0.00207614	6.60E-11	42.13023232	-0.0192	0.0165	0.2432
rs7644234	**CRP**	T	G	0.0163222	0.00206672	1.80E-15	62.37235151	0.0165	0.016	0.3009
rs76826401	**CRP**	C	G	0.0307094	0.00533607	8.60E-09	33.1205575	-0.1795	0.1886	0.3413
rs77027760	**CRP**	A	G	-0.018951	0.00259613	5.00E-14	53.28554399	0.0078	0.0305	0.7988
rs774211	**CRP**	C	T	-0.0189771	0.00266049	8.30E-14	50.87851683	0.0055	0.0238	0.8176
rs77522	**CRP**	C	G	-0.0150047	0.00216238	1.20E-13	48.14914878	-0.1051	0.0528	0.0463799
rs7759938	**CRP**	T	C	-0.0149993	0.00213924	4.60E-14	49.16103007	-0.005	0.0174	0.774399
rs77704739	**CRP**	C	T	-0.0542063	0.00504218	8.00E-28	115.5741877	-0.0157	0.1301	0.9042
rs77960347	**CRP**	G	A	0.0454695	0.0088446	2.00E-08	26.42907577	-0.1736	0.2667	0.515101
rs78248443	**CRP**	T	C	0.0450846	0.0046003	2.00E-23	96.04663308	0.1728	0.0938	0.0654998
rs796749	**CRP**	T	C	0.0247667	0.00246024	1.40E-25	101.339638	0.0297	0.0185	0.1074
rs799474	**CRP**	G	C	-0.0119545	0.00202585	3.90E-09	34.82140363	-0.0213	0.0161	0.1864
rs80097744	**CRP**	T	C	0.0336468	0.00269902	1.40E-38	155.4079888	0.056	0.0653	0.3906
rs80121667	**CRP**	T	C	-0.0315849	0.00591945	3.70E-08	28.47044868	0.0029	0.0907	0.9744
rs8017377	**CRP**	A	G	-0.0152228	0.00206019	2.60E-13	54.59747343	-0.0601	0.0306	0.0494698
rs872359	**CRP**	A	G	0.0123903	0.00244075	7.70E-09	25.77003805	0.022	0.0248	0.3755
rs9385391	**CRP**	A	G	-0.0152153	0.00207952	6.70E-14	53.53438877	-0.0368	0.0295	0.2115
rs9580199	**CRP**	T	C	0.013271	0.00212213	2.50E-10	39.10761586	0.0233	0.0161	0.1486
rs9891115	**CRP**	A	G	-0.0138254	0.00227953	3.30E-09	36.78432562	-0.0276	0.0165	0.0940892
rs9915486	**CRP**	C	T	-0.0149203	0.00209437	9.30E-14	50.75119894	0.0208	0.017	0.2211
rs9987289	**CRP**	G	A	0.112232	0.0036867	1.00E-200	926.7355405	-0.0276	0.0536	0.6062
rs999885	**CRP**	A	G	0.011256	0.00202383	6.30E-09	30.93272085	0.0235	0.0194	0.2263

CRP: C-reactive protein levels. EA: effect allele. OA: other allele. GX: beta-exposure. GX(SE): standard error of GX. GY: beta-outcome. GY(SE): standard error of GY.
