# Supplementary material for: Assessing Causality Between Endocrine, Nutritional, and Metabolic Disease and Pulmonary Tuberculosis: A Mendelian Randomization Study
Source: Health Sci Rep. 2025 May 29;8(6):e70875. doi: 10.1002/hsr2.70875 (PMC12122389; doi:10.1002/hsr2.70875)
Supplement: Supplementary file 2 — S2_File: Removing SNPs for being palindromic with intermediate allele frequencies or for incompatible alleles before MR analyses. [file HSR2-8-e70875-s003.pdf]

SNPs needed to be deleted before MR analyses

Removing the following SNPs for being palindromic with intermediate allele frequencies or for incompatible alleles		
**Exposure**	**palindromic with intermediate allele frequencies**	**incompatible alleles**
**ENMD**	2 (rs7754251, rs9275577)	
**T2DM**	3 (rs13234269, rs2058913, rs6494307)	
**HT**	1 (rs385863)	
**Obesity**	1 (rs1040070)	
**HC**	8 (rs10830963, rs11187141, rs13089972, rs17712208, rs2732469, rs66593272, rs75431224, rs857721)	
**FBG**	NA	
**OGTT**	NA	
**HbA1c**	8 (rs10830963, rs11187141, rs13089972, rs17712208, rs2732469, rs66593272, rs75431224, rs857721)	
**BGL**	2 (rs2302593, rs3750952)	
**ABL**	4 (rs10740118, rs2001945, rs2389858, rs6968865)	
**MCH**	19 (rs10793565, rs10801682, rs11159493, rs1134634, rs12601867, rs1558151, rs233701, rs257677, rs2748364, rs2836422, rs3909258, rs4680338, rs4805993, rs4815606, rs61421071, rs6987558, rs720783, rs7765828, rs9995319)	
**BMI**	21 (rs10887578, rs11250094, rs11634851, rs12507026, rs1454687, rs1860750, rs2396625, rs347551, rs355777, rs396755, rs4419475,	1 (rs9674487)
**TG**	8 (rs11118310, rs1454687, rs154254, rs213484, rs2288912, rs5112, rs62112763, rs7639927)	1 (rs28752924)
**TC**	1 (rs2001945)	
**LDL-c**	2 (rs2954029, rs964184)	
**HDL-c**	6 (rs10119644, rs1125873, rs1281959, rs133015, rs2498786, rs407133)	
**SHBG**	5 (rs2288004, rs2305833, rs3746575, rs3754186, rs4711750)	
**CRP**	3 (rs12654264, rs2276824, rs799474)	

ENMD, endocrine, nutritional and metabolic disease; T2DM, type 2 diabetes mellitus; HT, Hyperthyroidism; HC, High cholesterol; FBG, Fasting blood glucose; OGTT, Oral Two-hour glucose;HbA1c, hemoglobin Alc; BGL, Blood glucose levels; ABL, Albumin level; MCH, Mean corpuscular hemoglobin; BMI, Body mass index; TG, Triglyceride; TC, Total cholesterol; LDL-c, Low density lipoprotein cholesterol; HDL-c, HDL cholesterol; SHBG, Sex hormone-binding globulin levels; CRP, C-reactive protein levels
