# Supplementary material for: Assessing Causality Between Endocrine, Nutritional, and Metabolic Disease and Pulmonary Tuberculosis: A Mendelian Randomization Study
Source: Health Sci Rep. 2025 May 29;8(6):e70875. doi: 10.1002/hsr2.70875 (PMC12122389; doi:10.1002/hsr2.70875)
Supplement: Supplementary file 3 — S3_File: MR‐PRESSO analyses for the association between ENMD‐related exposure and PTB. [file HSR2-8-e70875-s001.pdf]

Table S1 MR-PRESSO analysis for the association between ENMD related exposure and PTB									
**Exposure**	**MR analysis**	**Causal Estimate**	**Sd**	**T**	***P*-value**	**RSSobs**	**Global Test *P* value**	**Distortion Test *P* value**	**Remove SNP**
**ENMD**	MR-PRESSO	0.39624715	0.0974166	4.067555	0.000722551	54.889	0.0104	<2e-04	2(rs2395490/rs7996350)
	Outlier-corrected MR-PRESSO	0.09248308	0.091001	1.016287	0.324615282				
**T2DM**	MR-PRESSO	0.06913612	0.0340456	2.030694	0.04461295	183.63	<2e-04	0.3784	rs1063355
	Outlier-corrected MR-PRESSO	0.05102922	0.0312929	1.630696	0.10573876				
**HT**	MR-PRESSO	0.048394462	0.0612285	0.790391	0.4496233	35.3113	8.00E-04	0.0118	rs28375776
	Outlier-corrected MR-PRESSO	-0.001905523	0.0457406	-0.04166	0.9677912				
**Obesity**	MR-PRESSO	-0.04622549	0.0775225	-0.59629	0.5830901	19.838	0.036	0.5204	rs6752378
	Outlier-corrected MR-PRESSO	-0.1064279	0.0609327	-1.74665	0.1790304				
**HC**	MR-PRESSO	-0.8863311	0.3298788	-2.68684	0.008657514	96.9496	0.2588		
	Outlier-corrected MR-PRESSO	No outliers							
**FBG**	MR-PRESSO	-0.007170833	0.0741224	-0.09674	0.9260809	6.50301	0.595		
	Outlier-corrected MR-PRESSO	No outliers							
**OGTT**	MR-PRESSO	-0.1117551	0.1120926	-0.99699	0.3369663	27.4342	0.0454	0.763	rs11708067
	Outlier-corrected MR-PRESSO	-0.1423761	0.0883067	-1.61229	0.1328716				
**HbA1c**	MR-PRESSO	0.0907461	0.0879723	1.03153	0.3114443	54.6783	0.0034	0.6666	rs2968478
	Outlier-corrected MR-PRESSO	0.1332223	0.0803356	1.658321	0.1092704				
**BGL**	MR-PRESSO	-0.07901385	0.079367	-0.99555	0.3216342	133.262	0.0934		
	Outlier-corrected MR-PRESSO	No outliers							
**ABL**	MR-PRESSO	0.0393431	0.0640504	0.614252	0.5396836	227.505	0.3832		
	Outlier-corrected MR-PRESSO								
**MCH**	MR-PRESSO	-0.007021368	0.0353172	-0.19881	0.8425048	512.226	0.0072	0.9192	rs2036916
	Outlier-corrected MR-PRESSO	-0.004896567	0.0347898	-0.14075	0.8881345				
**BMI**	MR-PRESSO	-0.2418558	0.0597967	-4.04464	6.24E-05	468.065	0.0478		
	Outlier-corrected MR-PRESSO	No outliers							
**TG**	MR-PRESSO	-0.0376209	0.0497504	-0.75619	0.4503249	309.671	6.00E-04	0.296	3(rs1064173/rs2678379/rs2812208)
	Outlier-corrected MR-PRESSO	-0.02025954	0.042606	-0.47551	0.6348913				
**TC**	MR-PRESSO	-0.06506101	0.0547634	-1.18804	0.236341	229.042	0.0218	0.452	2(rs1049107/rs562338)
	Outlier-corrected MR-PRESSO	-0.0436858	0.0494497	-0.88344	0.3781581				
**LDL-c**	MR-PRESSO	-0.1041732	0.0481834	-2.16201	0.03376429	93.2088	0.1294		
	Outlier-corrected MR-PRESSO	No outliers							
**HDL-c**	MR-PRESSO	0.0342189	0.0469803	0.728368	0.4669314	364.326	0.0282		
	Outlier-corrected MR-PRESSO	No outliers							
**SHBG**	MR-PRESSO	-0.003785383	0.0349557	-0.10829	0.9138256	376.814	0.2652		
	Outlier-corrected MR-PRESSO	No outliers							
**CRP**	MR-PRESSO	0.001630575	0.0375486	0.043426	0.9653982	236.778	0.5948		
	Outlier-corrected MR-PRESSO	No outliers							

ENDM, endocrine, nutritional and metabolic disease; T2DM, type 2 diabetes mellitus; HT Hyperthyroidism; HC, High cholesterol; FBG, Fasting blood glucose; OGTT, Oral Two-hour glucose; HbA1c, hemoglobin Alc; BGL, Blood glucose levels; ABL, Albumin level; MCH, Mean corpuscular hemoglobin; BMI, Body mass index; TG, Triglyceride; TC, Total cholesterol; LDL-c, Low density lipoprotein cholesterol; HDL-c, HDL cholesterol; SHBG, Sex hormone-binding globulin levels; CRP, C-reactive protein levels
