# Supplementary material for: Assessing Causality Between Endocrine, Nutritional, and Metabolic Disease and Pulmonary Tuberculosis: A Mendelian Randomization Study
Source: Health Sci Rep. 2025 May 29;8(6):e70875. doi: 10.1002/hsr2.70875 (PMC12122389; doi:10.1002/hsr2.70875)
Supplement: Supplementary file 4 — S4_File: Four types of causal effect graphs of SNPs associated with ENMD on PTB: a, forest plot for the causal effects of ENMD on PTB; b, scatter plot for the causal effect of ENMD on PTB; c, funnel plot to assess heterogeneity; d, forest plot for leave‐one‐out analysis. [file HSR2-8-e70875-s002.pdf]

# Figure S1: ENMD associated-SNPS with risk of PTB

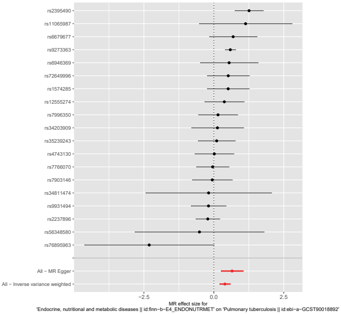


A. Forest plot of causal effect of ENMD on PTB

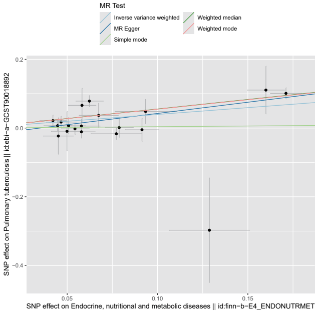


B. Scatter plot of causal effect of ENMD on PTB

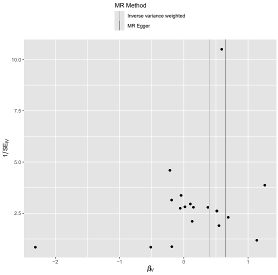


C. Funnel plot of causal effect of ENMD on PTB

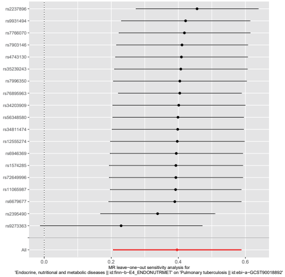


D. Leave-one-out analysis of ENMD on PTB

Figure S2: T2DM associated-SNPS with risk of PTB

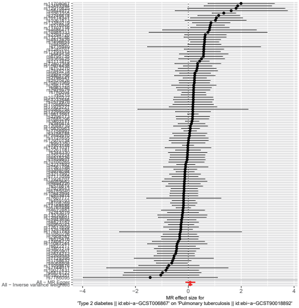


A. Forest plot of causal effect of T2DM on PTB

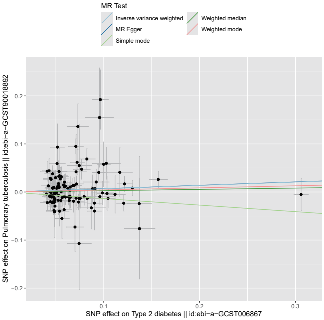


B. Scatter plot of causal effect of T2DM on PTB

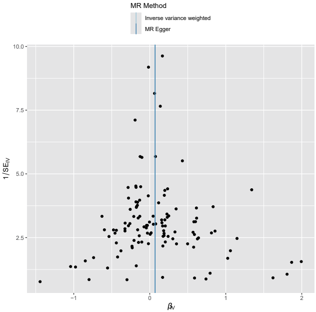


C. Funnel plot of causal effect of T2DM on PTB

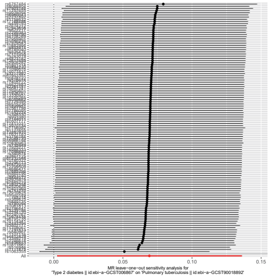


D. Leave-one-out analysis of T2DM on PTB

# Figure S3: Hyperthyroidism associated-SNPS with risk of PTB

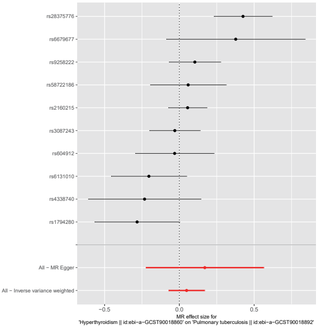


A. Forest plot of causal effect of Hyperthyroidism on PTB

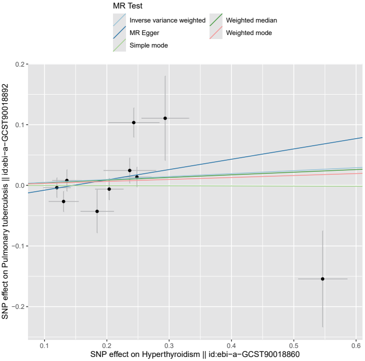


B. Scatter plot of causal effect of Hyperthyroidismon PTB

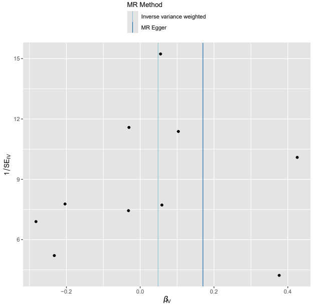


C. Funnel plot of causal effect of Hyperthyroidism on PTB

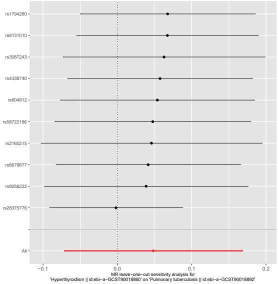


D. Leave-one-out analysis of Hyperthyroidism on PTB

# Figure S4: Obesity associated-SNPS with risk of PTB

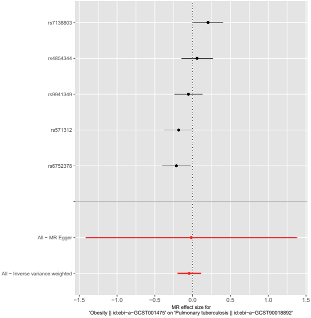


A. Forest plot of causal effect of Obesity on PTB

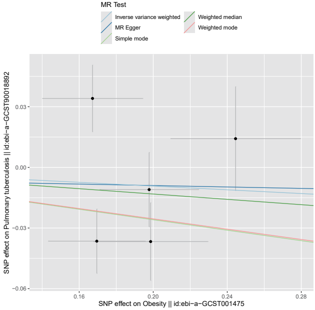


B. Scatter plot of causal effect of Obesity on PTB

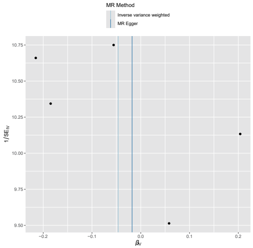


C. Funnel plot of causal effect of Obesity on PTB

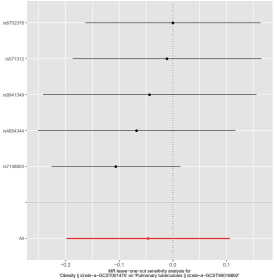


D. Leave-one-out analysis of Obesity on PTB

# Figure S5: High cholesterol associated-SNPS with risk of PTB

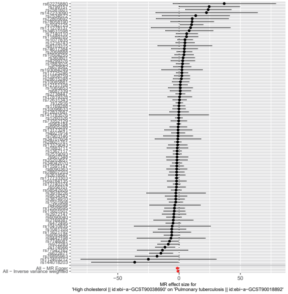


A. Forest plot of causal effect of HC on PTB

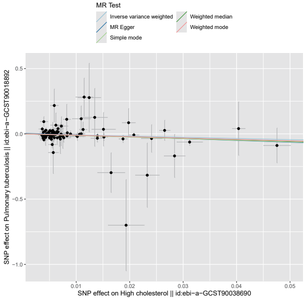


B. Scatter plot of causal effect of HC on PTB

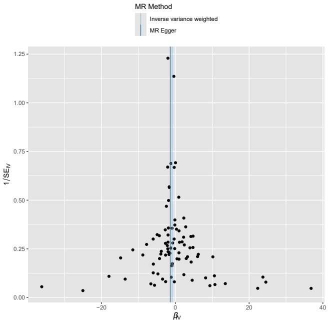


C. Funnel plot of causal effect of HC on PTB

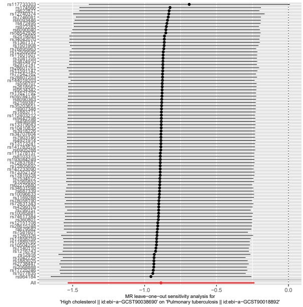


D. Leave-one-out analysis of HC on PTB

# Figure S6: Fasting blood glucose associated-SNPS with risk of PTB

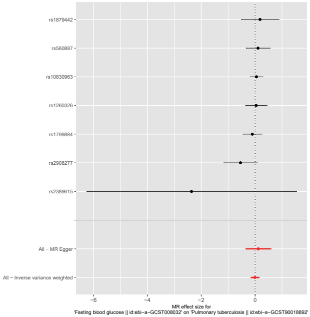


A. Forest plot of causal effect of FBG on PTB

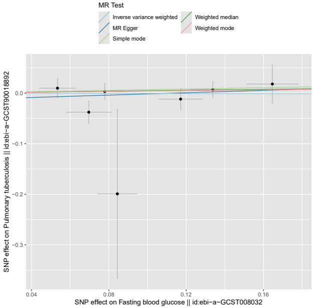


B. Scatter plot of causal effect of FBG on PTB

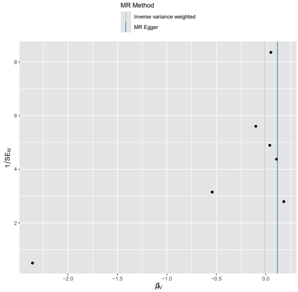


C. Funnel plot of causal effect of FBG on PTB

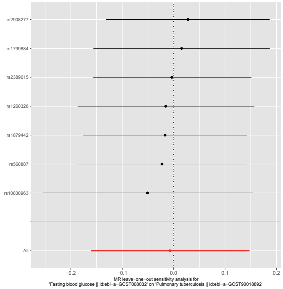


D. Leave-one-out analysis of FBG on PTB

# Figure S7: OGTT associated-SNPS with risk of PTB

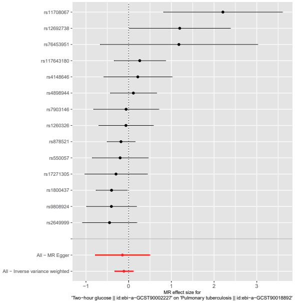


A. Forest plot of causal effect of OGTT on PTB

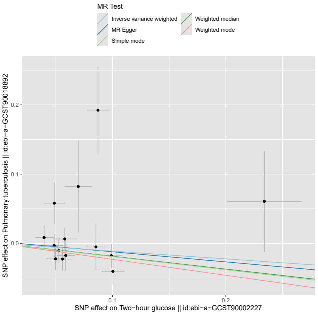


B. Scatter plot of causal effect of OGTT on PTB

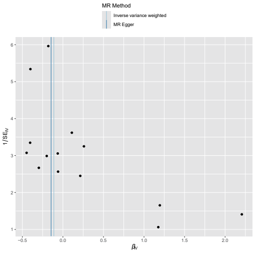


C. Funnel plot of causal effect of OGTT on PTB

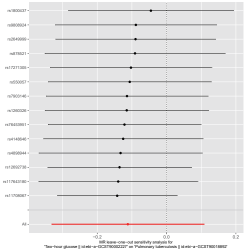


D. Leave-one-out analysis of OGTT on PTB

# Figure S8: HbA1c associated-SNPS with risk of PTB

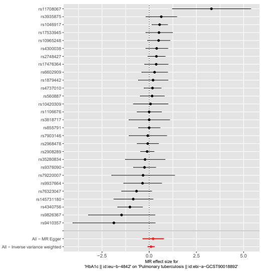


A. Forest plot of causal effect of HbA1c on PTB

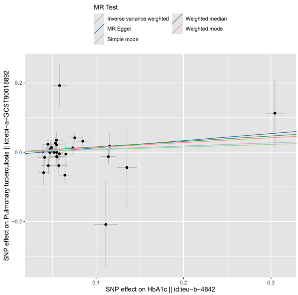


B. Scatter plot of causal effect of HbA1c on PTB

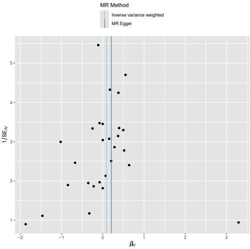


C. Funnel plot of causal effect of HbA1c on PTB

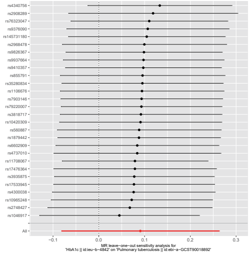


D. Leave-one-out analysis of HbA1c on PTB

# Figure S9: Blood glucose level associated-SNPS with risk of PTB

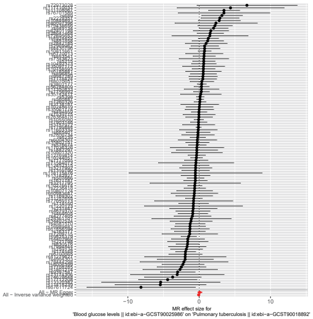


A. Forest plot of causal effect of BGL on PTB

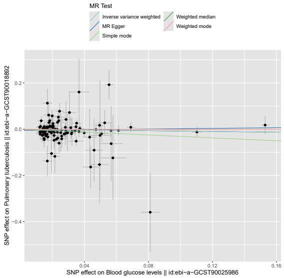


B. Scatter plot of causal effect of BGL on PTB

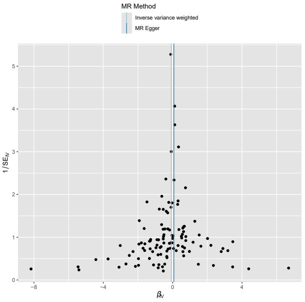


C. Funnel plot of causal effect of BGL on PTB

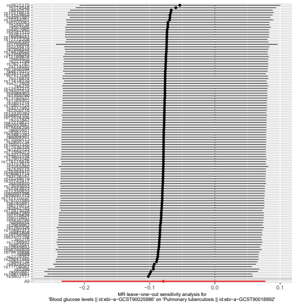


D. Leave-one-out analysis of BGL on PTB

Figure S10: Albumin level associated-SNPS with risk of  PTB

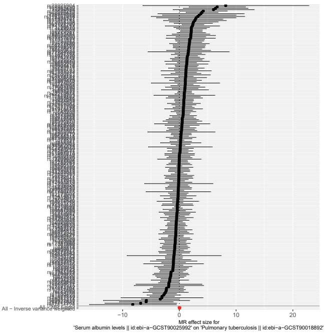


A. Forest plot of causal effect of  ABL on PTB

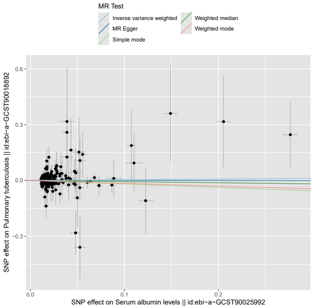


B. Scatter plot of causal effect of  ABL on PTB

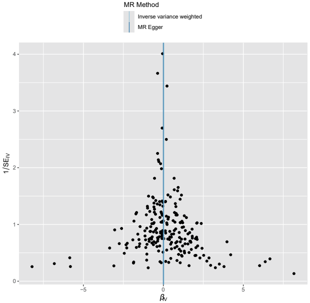


C. Funnel plot of causal effect of  ABL  on PTB

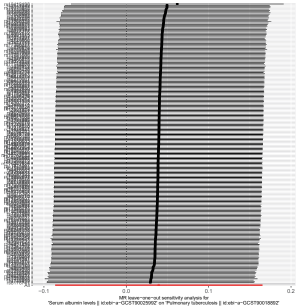


D. Leave-one-out analysis of  ABL on PTB

# Figure S11: Mean corpuscular hemoglobin associated-SNPS with risk of PTB

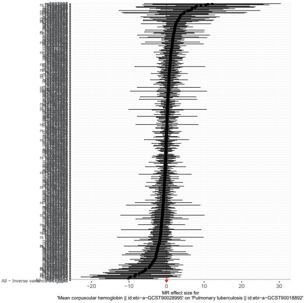


A. Forest plot of causal effect of MCH on PTB

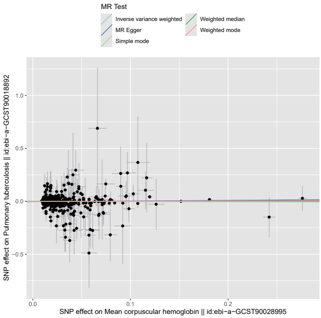


B. Scatter plot of causal effect of MCH on PTB

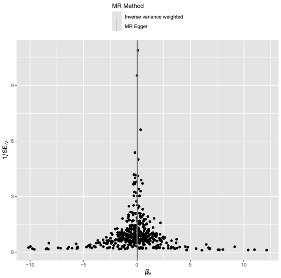


C. Funnel plot of causal effect of MCH on PTB

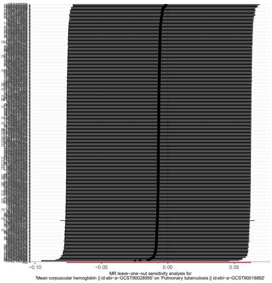


D. Leave-one-out analysis of MCH on PTB

# Figure S12: BMI associated-SNPS with risk of PTB

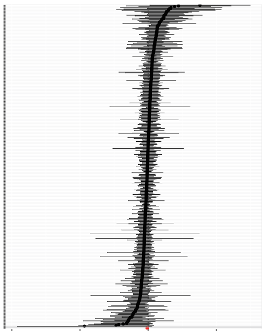

A. Forest plot of causal effect of BMI on PTB

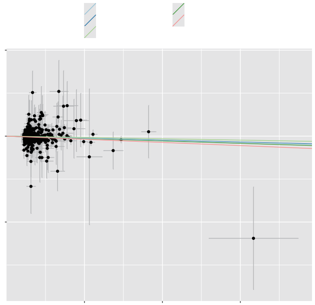

B. Scatter plot of causal effect of BMI on PTB

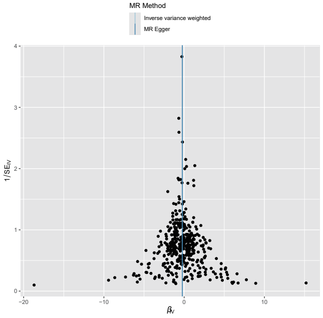


C. Funnel plot of causal effect of BMI on PTB

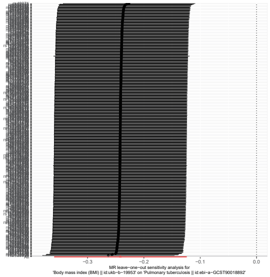


D. Leave-one-out analysis of BMI on PTB

# Figure S13: TG associated-SNPS with risk of PTB

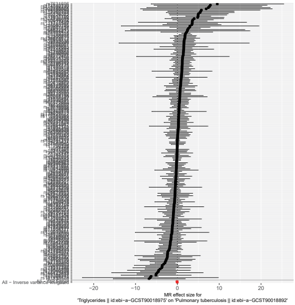


A. Forest plot of causal effect of TG on PTB

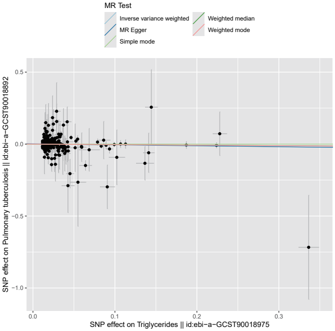


B. Scatter plot of causal effect of TG on PTB

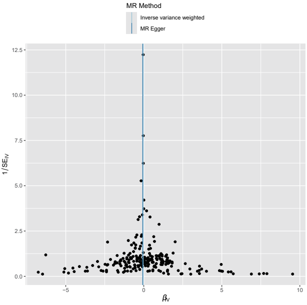


C. Funnel plot of causal effect of TG on PTB

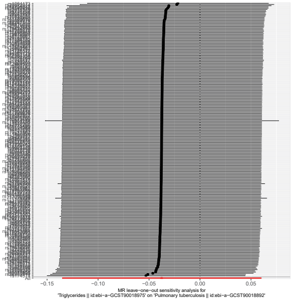


D. Leave-one-out analysis of TG on PTB

# Figure S14: TC associated-SNPS with risk of PTB

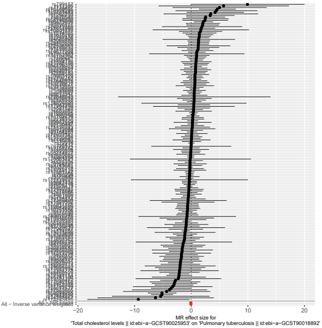


A. Forest plot of causal effect of TC on PTB

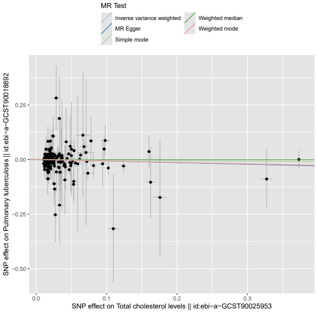


B. Scatter plot of causal effect of TC on PTB

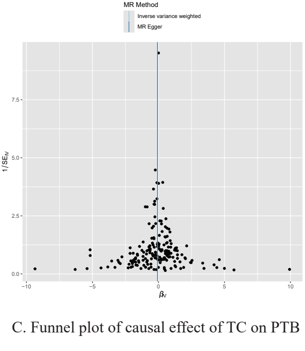


C. Funnel plot of causal effect of TC on PTB

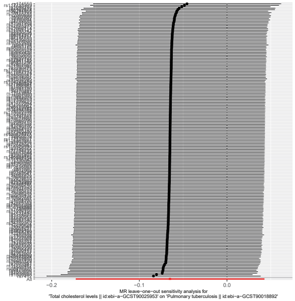


D. Leave-one-out analysis of TC on PTB

# Figure S15: LDL-c associated-SNPS with risk of PTB

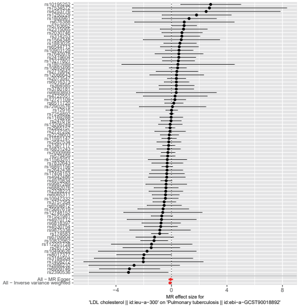


A. Forest plot of causal effect of LDL-c on PTB

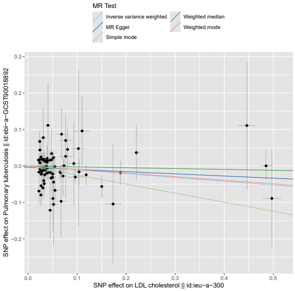


B. Scatter plot of causal effect of LDL-c on PTB

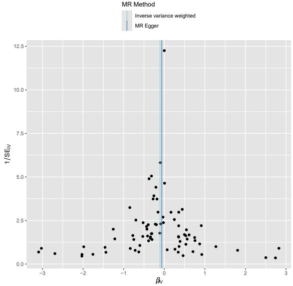


C. Funnel plot of causal effect of LDL-c on PTB

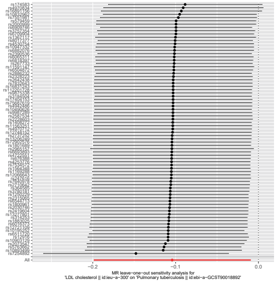


D. Leave-one-out analysis of LDL-c on PTB

Figure S16: HDL-c associated-SNPS with risk of PTB

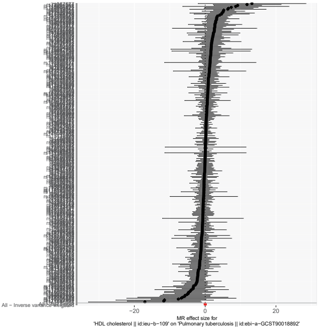


A. Forest plot of causal effect of HDL-c on PTB

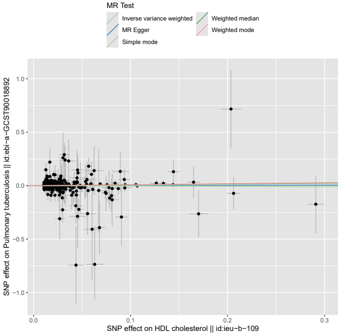


B. Scatter plot of causal effect of HDL-c on PTB

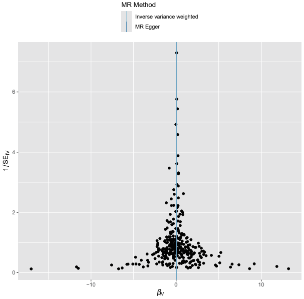


C. Funnel plot of causal effect of HDL-c on PTB

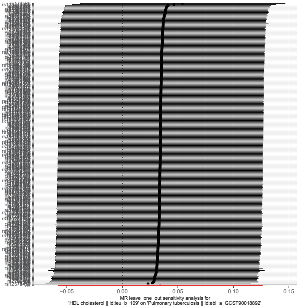


D. Leave-one-out analysis of HDL-c on PTB

# Figure S17: SHBG associated-SNPS with risk of PTB

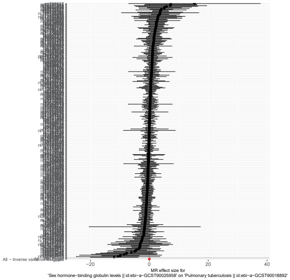


A. Forest plot of causal effect of SHBG on PTB

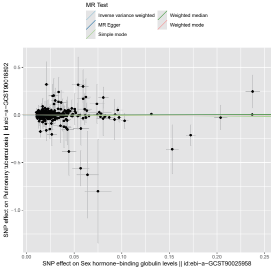


B. Scatter plot of causal effect of SHBG on PTB

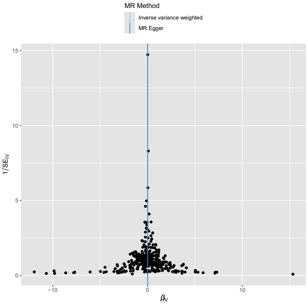


C. Funnel plot of causal effect of SHBG on PTB

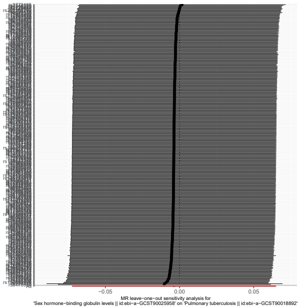


D. Leave-one-out analysis of SHBG on PTB

# Figure S18: CRP associated-SNPS with risk of PTB

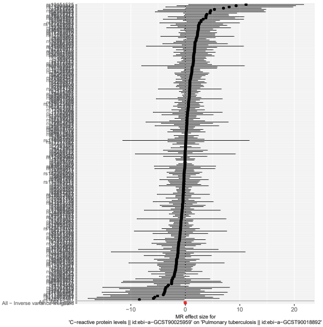


A. Forest plot of causal effect of CRP on PTB

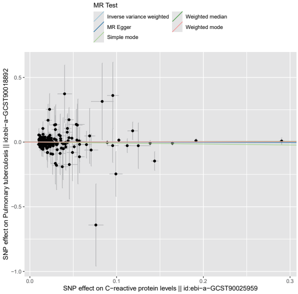


B. Scatter plot of causal effect of CRP on PTB

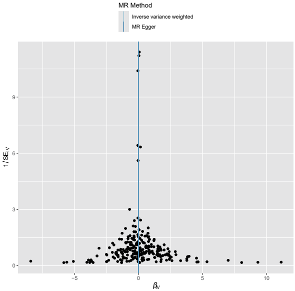


C. Funnel plot of causal effect of CRP on PTB

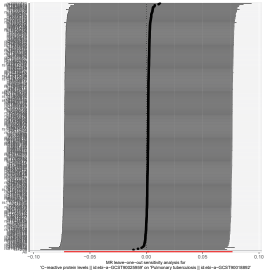


D. Leave-one-out analysis of CRP on PTB
